# General Principles for the Design of Visible‐Light‐Responsive Photoswitches: Tetra‐*ortho*‐Chloro‐Azobenzenes

**DOI:** 10.1002/anie.202008700

**Published:** 2020-09-23

**Authors:** Lucien N. Lameijer, Simon Budzak, Nadja A. Simeth, Mickel J. Hansen, Ben L. Feringa, Denis Jacquemin, Wiktor Szymanski

**Affiliations:** ^1^ Medical Imaging Center University Medical Center Groningen University of Groningen Hanzeplein 1 9713GZ Groningen The Netherlands; ^2^ Stratingh Institute for Chemistry University of Groningen Nijenborgh 4 9747AF Groningen The Netherlands; ^3^ Department of Chemistry Faculty of Natural Sciences Matej Bel University Tajovkého 40 97401 Banska Bystrica Slovakia; ^4^ CEISAM Lab UMR 6230 Université de Nantes CNRS 44000 Nantes France

**Keywords:** azobenzene, photochromism, photoswitches, TD-DFT, visible light

## Abstract

Molecular photoswitches enable reversible external control of biological systems, nanomachines, and smart materials. Their development is driven by the need for low energy (green‐red‐NIR) light switching, to allow non‐invasive operation with deep tissue penetration. The lack of clear design principles for the adaptation and optimization of such systems limits further applications. Here we provide a design rulebook for tetra‐ortho‐chloroazobenzenes, an emerging class of visible‐light‐responsive photochromes, by elucidating the role that substituents play in defining their key characteristics: absorption spectra, band overlap, photoswitching efficiencies, and half‐lives of the unstable cis isomers. This is achieved through joint photochemical and theoretical analyses of a representative library of molecules featuring substituents of varying electronic nature. A set of guidelines is presented that enables tuning of properties to the desired application through informed photochrome engineering.

## Graphical abstract

Molecular photoswitches that respond to visible light irradiation enable external, non‐invasive control over biological systems, materials, and molecular machines. Here we present a systematic spectroscopic and theoretical investigation into the photochemistry of tetra‐*ortho*‐chloro‐azobenzenes and outline the design principles that allow the optimization of those emerging photochromes on a molecular level.

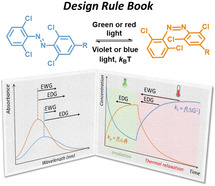

## Introduction

Molecular photoswitches form the basis of light‐responsive systems that are designed to enable reversible control of function with high spatiotemporal resolution.^[1]^ They have found application in remotely manipulating biological systems,[^2^, ^3^] smart materials,[^4^, ^5^] and molecular machines.[^6^, ^7^] In particular, their potential in biomedical context, along the principles of photopharmacology,[^8^, ^9^, ^10^, ^11^] evoked considerable interest in recent years. The available panel of molecular photoswitches features many established architectures that mainly rely on double bond isomerisation (azobenzenes,^[12]^ azoheteroarenes,^[13]^ stilbenes,^[14]^ hemithioindigos^[15]^), electrocyclisation (diarylethenes^[16]^), or mixed mechanisms (spiropyrans^[17]^). Furthermore, various novel designs^[18]^ have appeared during the last decade, including donor–acceptor Stenhouse adducts (DASAs),[^19^, ^20^] hydrazone^[21]^‐ and acylhydrazone^[22]^‐based switches, BF_2_‐coordinated azo compounds,^[23]^ diazocines,^[24]^ indigos,[^15^, ^25^] and iminothioindoxyls.^[26]^ The development of new molecular photoswitches is largely driven by the challenge of enabling the use of visible, and red or even near‐IR (NIR) light for operation in both directions.[^27^, ^28^, ^29^] This is relevant especially in biological applications, where red/NIR light enables deep (1 cm) tissue penetration without the toxic effects induced by higher energy light.^[30]^

The successful application of the new visible‐light‐responsive photoswitches depends on establishing their design principles, based on the understanding of the interplay between the nature of the substituents and the key photochemical properties. This understanding is enabled through synthesis, spectroscopic studies, and theoretical investigations.[^31^, ^32^, ^33^] It ultimately allows both the tuning of these properties, and the effective choice of substituents determining the function of the photoresponsive unit in a biological system, material, or a molecular machine.

**Figure 1:**
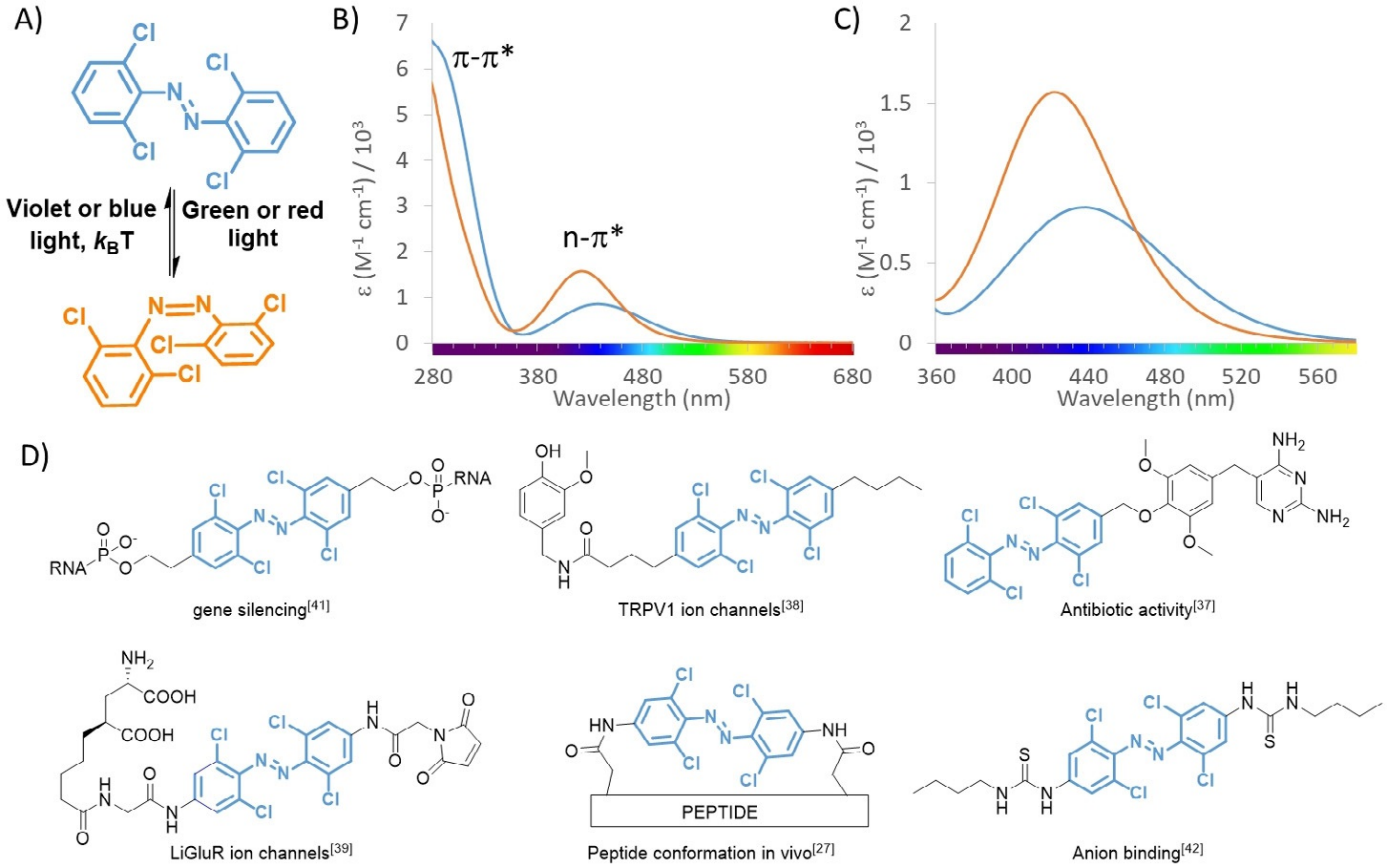
Photochromism and applications of tetra‐*ortho*‐chloro‐azobenzenes. A) The *trans*‐isomer can be switched to the *cis* isomer using green or red light. The metastable *cis* isomer can be switched back using violet or blue light. B) The spectra of both isomers feature the high energy absorption band in the UV region, associated with the symmetry‐allowed π–π* transition, and a low energy band in the visible region, associated with the weakly allowed n–π* transition. C) The operation of tetra‐*ortho*‐chloro‐azobenzenes with visible light is enabled due to the separation of n–π* bands. D) Examples of application of tetra‐*ortho*‐chloro‐azobenzenes for visible‐light regulation of processes in biology and supramolecular chemistry.

Here we present a systematic spectroscopic and theoretical investigation into the photochemistry of tetra‐*ortho*‐chloro‐azobenzenes, with the aim to provide a guide for their design. Tetra‐*ortho*‐substituted azobenzenes emerged as privileged light‐responsive molecular photoswitches, with good absorption band separation and half‐lives of the metastable *cis* isomer in the range that enables multiple applications.[^27^, ^28^, ^29^, ^34^, ^35^, ^36^] Among them, azobenzenes with all four *ortho* positions substituted with chlorine atoms (Figure 1 A), have already enabled using green and even red light to control peptide conformation,^[27]^ antibiotic potency,^[37]^ ion channel activity,[^38^, ^39^, ^40^] and the function of nucleic acids^[41]^ and ion receptors[^42^, ^43^] for controlling the transport through biological membranes^[44]^ (Figure 1 D). However, while for normal azobenzenes several relationships between structure and photochemical properties have been defined, only little systematic information is available for the tetra‐*ortho*‐substituted systems, making their design largely a trial‐and‐error endeavor. The main difference between those switches and classical azobenzenes comes from the fact that, for their operation in both directions, visible‐light absorption bands are used, which correspond to weakly allowed transitions of n‐π* character (Figure 1 B,C). This presents a challenge for informed design of photoresponsive units for applications and necessitates the systematic study on parameters that govern the key properties, such as band separation, switching efficiency, photostationary state distributions and thermal stability of the metastable isomer.

## Results and Discussion

A library of ten compounds with different groups in one of the *para* positions was designed, spanning the range of Hammet *σ*_para_ constants from the most electron‐donating (−NMe_2_, *σ*_para_=−0.83) to the most electron‐withdrawing (−NO_2_, *σ*_para_=+0.78). We focused on the *para*‐substituents, since *meta* ones show less pronounced resonance effects, and all *ortho* positions are occupied in the studied molecules. Furthermore, in all the applied molecules (Figure 1 D), *para*‐substituents are used.

**Figure 2:**
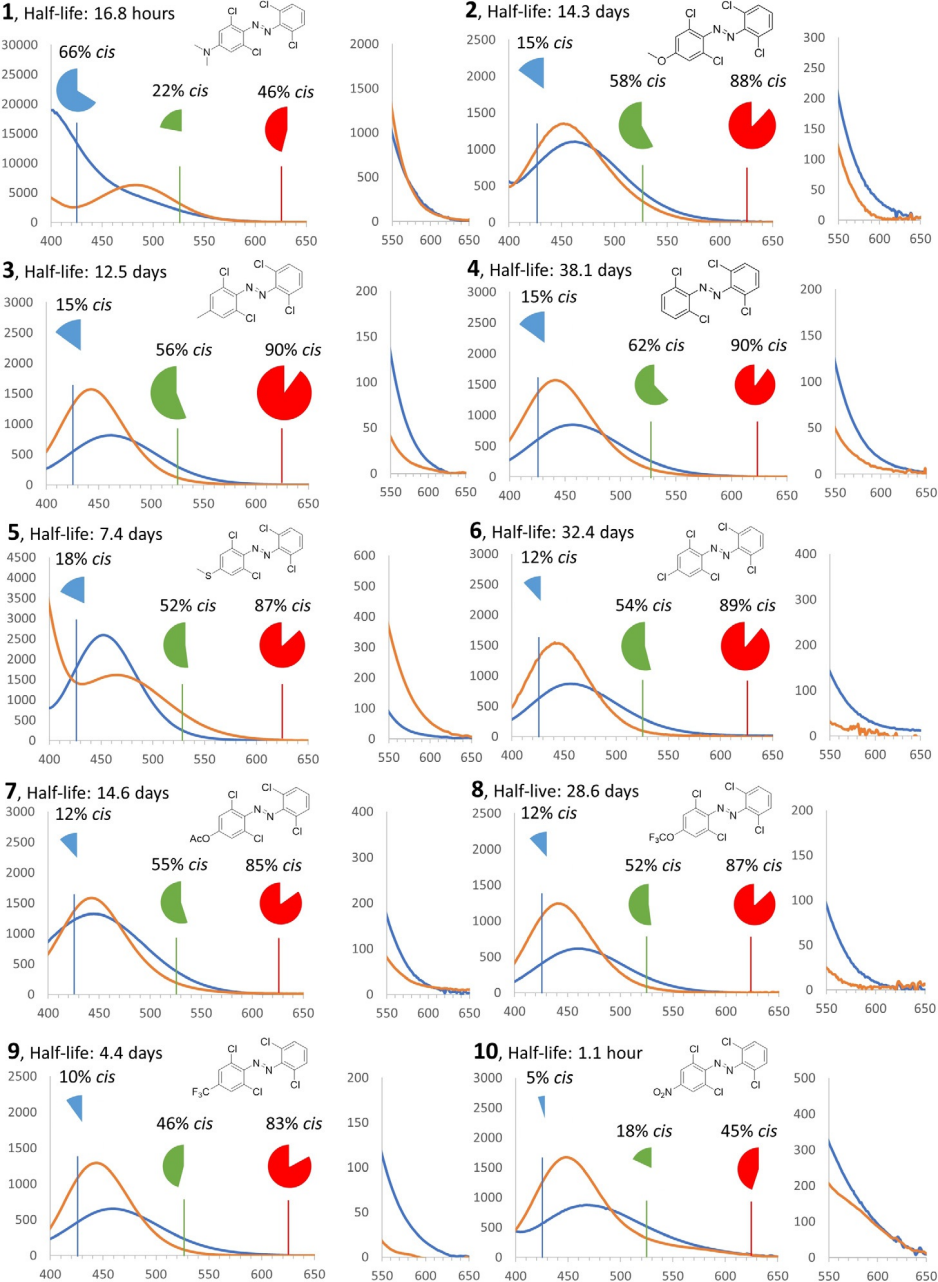
Visible‐light band separation in compounds **1**–**10** for the *trans* isomers (blue spectra) and *cis* isomers (orange spectra) in DMSO. The *x*‐axis depicts the wavelength *λ* (nm) and the *y*‐axis depicts the molar attenuation coefficient *ϵ* (M^−1^ cm^−1^). On the right side of each spectrum, a panel is provided showing the band separation in more detail in the 550–650 nm range. The spectra of pure *cis* isomers were calculated by irradiation of the sample in [D_6_]DMSO with *λ*_exc_=526 nm (FWHM=90 nm) until reaching PSS. ^1^H NMR spectra of aliquots (0.6 mL) were then taken to determine the cis/trans ratio, followed by calculation of the cis spectra based upon the molar extinction coefficients of the trans‐species (see the Supporting Information for full spectra). The pie charts show the content of *cis* isomer at PSS that can be achieved under irradiation with *λ*_exc_=426 nm (blue chart), *λ*_exc_=526 nm (green chart) or 625 nm (red chart) LEDs, as determined by NMR spectroscopy in [D_6_]DMSO (see the Supporting Information).

The synthesis of tetra‐*ortho*‐substituted azobenzenes is known to be challenging due to the highly sterically congested nature of the central N=N double bond that is surrounded with four large chlorine substituents. This limits the use of classical methods for azobenzene synthesis, such as the Baeyer–Mills reaction,^[45]^ diazonium coupling^[27]^ or oxidative coupling of anilines,^[46]^ and has inspired the development of methods better suited for these targets: late‐stage C−H chlorination[^38^, ^47^, ^48^] and the reaction of diazonium salts with lithiated aromatic compounds, reported recently by our group.^[49]^ Here, we use the latter method to prepare a versatile library of tetra‐*ortho*‐chloro‐azobenzenes (Figure 2, Table 1), additionally highlighting the robustness of this method. Furthermore, this substrate scope was acquired without the use of transition metals, instead using a Smiles rearrangement to synthesize compound **1** (see the Supporting Information).

**Table 1:** Properties of compounds **1**–**10** (in DMSO) relevant for their photochromism.^[a]^

	R	*σ*_p_ ^[54]^	*S*_0_‐*‐S*_1_ *λ*_max_ (*ϵ*)		PSD 426 nm	PSD 526 nm	PSD 625 nm	*φ* ^*t*→c^	*φ* ^*c*→t^	*φ* ^*c*→t^ × *ϵ* ^*c*,426^	*φ* ^*t*→c^ × *ϵ* ^*t*,526^	*φ* ^*t*→c^ × *ϵ* ^*t*,625^	half‐life at 25 °C	thermal *cis* to *trans* isomerization			
			*trans*	*cis*	[% *cis*]	[% *cis*]	[% *cis*]	[%]	[%]				[days]	Δ*G* ^calc^ [kJ mol^−1^]	Δ*G* ^exp^ [kJ mol^−1^]	Δ*H* ^exp^ [kJ mol^−1^]	Δ*S* ^exp^ [J mol^−1^]
1	NMe_2_	−0.83	–	483 (6301)	66	22	46	34	nd^[b]^	nd^[b]^	683	10	0.70	69.1	101.6	119.5	60.0
2	OMe	−0.27	463 (1100)	452 (1352)	15	58	88	63	51	506	264	8	14.3	72.4	108.7	107.6	−3.9
3	Me	−0.17	461 (814)	443 (1572)	15	56	90	30	72	963	87	<1	12.5	72.0	108.4	96.0	−41.7
4	H	0.0	457 (847)	441 (1569)	15	62	90	51	48	660	113	3	38.1	71.4	111.1	110.3	−2.9
5	SMe	0.0	465 (1616)	452 (2595)	18	52	87	21	37	663	155	4	7.4	70.0	107.1	103.6	−11.6
6	Cl	0.23	456 (866)	441 (1546)	12	54	89	43	nd^[c]^	nd^[c]^	104	6	32.4	72.2	110.7	113.5	9.2
7	OAc	0.31	444 (1324)	442 (1583)	12	55	85	57	73	1010	212	5	14.6	72.5	108.8	99.5	−31.0
8	OCF_3_	0.35	461 (618)	441 (1216)	12	52	87	41	nd^[c]^	nd^[c]^	87	2	28.6	72.4	110.4	111.0	1.9
9	CF_3_	0.54	459 (652)	444 (1290)	10	46	83	20	47	511	47	<1	4.4	73.1	105.8	118.1	41.5
10	NO_2_	0.78	467 (873)	448 (1673)	5	18	45	18	30	384	89	6	0.046	67.9	94.5	100.0	18.5

[a] Position of absorption maxima (*λ*_max_), molar attenuation coefficients (*ϵ)*, photostationary state distributions (PSD) determined under blue (*λ*_exc_=426 nm), green (*λ*_exc_=526 nm), and red (*λ*_exc_=625 nm) light irradiation, quantum yields for forward switching determined at *λ*=532 nm irradiation (*φ* ^*t*→c^), and for reverse switching (*φ* ^*c*→t^) determined at *λ*=445 nm irradiation, photoswitching cross‐sections (*φ*×*ϵ*) at blue (*λ*_exc_=426 nm), green (*λ*_exc_=526 nm) and red (*λ*_exc_=625 nm) light, experimentally determined half‐life of the metastable *cis* isomer at 25 °C, and the calculated and measured activation barrier parameters for the thermal *cis*‐to‐*trans* isomerization. [b] Quantum yield for the reverse switching was not determined for compound **1** owing to the presence of the overlapping, bathochromically shifted π–π* band at the *λ*=445 nm part of the spectrum. [c] Quantum yield values could not be determined for compounds **6** and **8** owing to lack of clear convergence of obtained data to a convincing fit.

For the photochemical evaluation, we have chosen DMSO as the solvent, because it facilitates the solubility needed throughout the analytical methods used (UV/Vis spectrophotometry, NMR spectroscopy) and, with its intermediate polarity, it approximates well both organic and aqueous systems well. Even more importantly, in photopharmacology it is often used as a solvent for stock solutions, which after irradiation are diluted into aqueous media for biological evaluation.[^37^, ^50^, ^51^, ^52^] Hence, it is often the photochemistry in DMSO that determines the properties of molecules in final applications.

The photochemistry of tetra‐*ortho*‐substituted azobenzenes in the visible range of the electromagnetic spectrum is related to the presence of *S*_0_–*S*_1_ absorption bands that are traditionally associated with n–π* transitions.^[53]^ The installation of *ortho* substituents induces a significant distortion of the geometry, which in turn, allows for the separation of the *S*_0_–*S*_1_ absorption bands of the two isomers and thus enables their selective excitation with light of specific wavelengths.^[29]^ This selective addressing is crucial, because the ratio of molecular attenuation coefficients *ϵ* of the two forms at the irradiation wavelength is one of the two key factors (the other being the ratio of quantum yields *φ* for the photoisomerisation in both directions) determining the photostationary state distribution (PSD) of isomers under irradiation at that wavelength.

The spectra of compounds **1**–**10** are presented in Figure 2 and their properties are summarized in Table 1. In almost all cases, we have observed n‐π* absorption bands for the *trans* isomer in the *λ*=450–465 nm region. Only for compound **1**, which features a very strong electron‐donating −NMe_2_ group, we did not observe a well‐resolved band in this region, probably due to the overlap with a very strong π–π* band. The position of the bands was well reproduced theoretically (Table 1).

**Figure 3:**
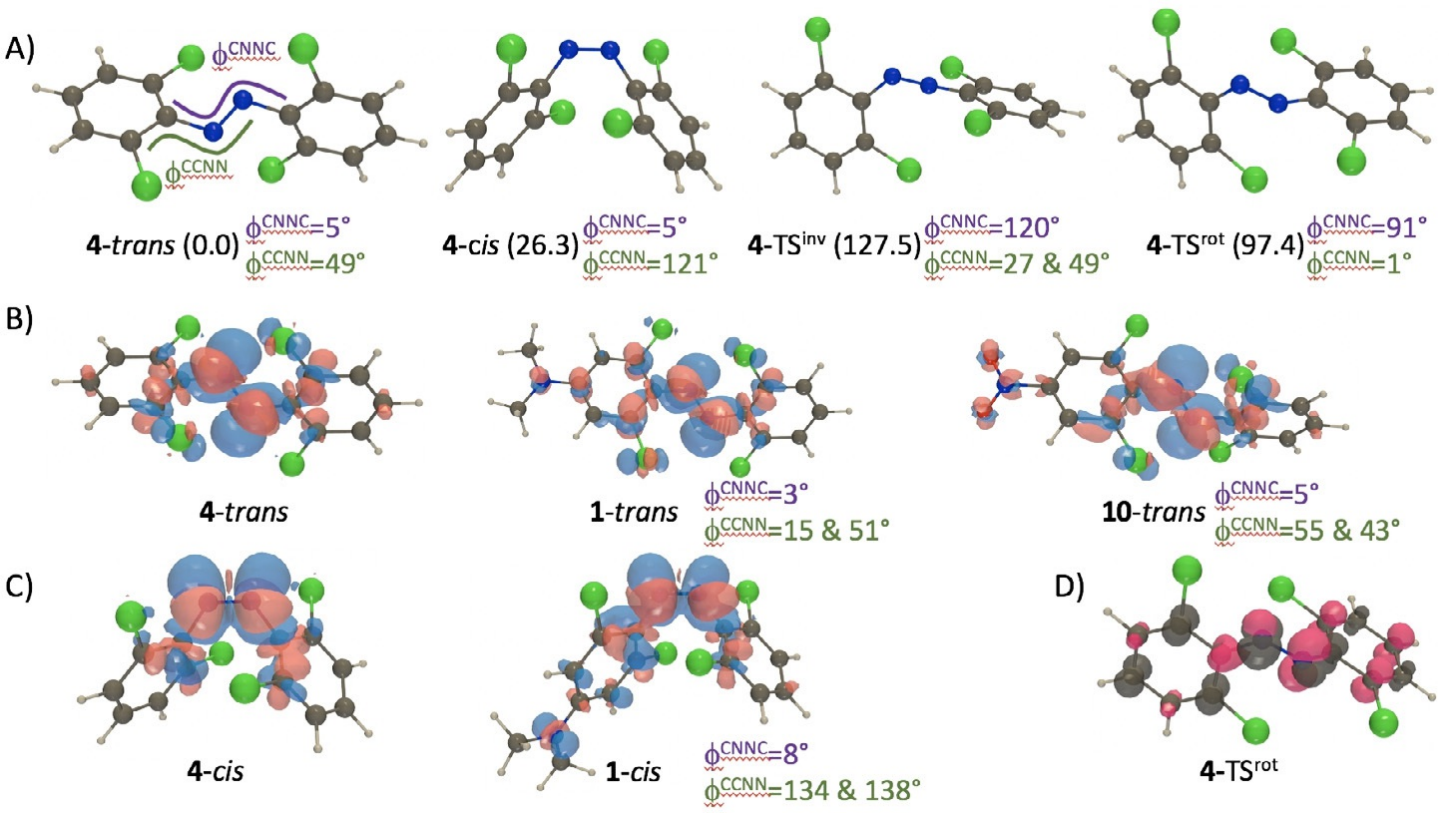
A) Representation of the DFT optimal geometries for the two stable isomers as well as inversion and rotation transition states, together with relative free energies in parenthesis (in kJ mol^−1^) and key dihedral angles for **4**; B) electron density difference plot for the lowest transitions in three selected *trans* compounds. The blue and red lobes indicate regions of decrease and increase of density upon excitation, respectively. Representation threshold 15×10^−4^ au; C) same for the *cis* isomers; D) spin density difference for the rotation transition state of **4** as given by BS‐DFT. Representation threshold 10×10^−3^ au.

To shed more light onto the experimental results, we have performed theoretical calculations on all *trans* isomers (see the Supporting Information for details). It should be noted that all compounds *trans*‐**1**–**10** strongly depart from the planarity of standard azobenzenes, with a 48.8° twisting of the aromatic rings with respect to the diazo bond in **4** (Figure 3 A). In the Supporting Information, Table S1, we provide the transition energies determined with TD‐DFT and with additional CC2 corrections for all structures (see the Supporting Information for technical details). For compound **4**, the best estimate for the *S*_0_–*S*_1_ excitation is 466 nm, in obvious agreement with the experimental value 457 nm (see Table 1). As can be seen in Figure 3 B, this transition has an n–π* topology, mainly localized on the diazo bond, though it is slightly dipole‐allowed due to the above‐mentioned non‐planarity (*f*=0.05). According to theory, this transition is separated by more than 1 eV from the following excitation (*S*_0_–*S*_2_, *f*=0.03), and even by 1.5 eV from the intense *S*_0_–*S*_4_ absorption (*f*=0.41). Amongst all studied compounds, the most red‐shifted n‐π* transition should occur in the NMe_2_‐bearing compound **1** (497 nm, *f*=0.11) according to theory, but in that case it is likely buried under the very probable *S*_0_–*S*_2_ excitation (*f*=0.86), that is much closer‐lying than in compound **4**. The second most red‐shifted n–π* transition returned by theory is obtained for the NO_2_‐substituted compound **10** (482 nm, *f*=0.09), which fits the experimental ordering (see Table 1). As can be seen in Figure 3 B, the addition of strong donating or accepting groups does not fundamentally change the nature of the transition, although one notice small red lobes (accepting character) on the nitro group of compound **10**. For **1**, in contrast, it is mostly the planarization on one side of the compound that accounts for the improved delocalization and the observed red‐shift, rather than the direct donating nature of the amino moiety.

Upon irradiation with green and red light, we consistently observe the emergence of a hypsochromically shifted band, mostly in the *λ*=440–455 nm region, which corresponds to the *cis* isomer. The calculations indicate that the unsubstituted *cis*‐**4** is 26.3 kJ mol^−1^ less stable than its *trans* counterpart, with a geometry rather typical for these structure (Figure 3 A). The lowest excited state conserves its n‐π* character (Figure 3 C), and the CC2‐corrected vertical excitation energy of 434 nm (*f*=0.03), is again close to the experimental value (441 nm, see Table 1). The most red‐shifted transition computed in the *cis* series is obtained for **1** (461 nm, *f*=0.15), which is again consistent with experimental data. As can be seen in Figure 3 C, the geometry of *cis*‐**1** resembles closely the one of *cis*‐**4** but the lone pair of the amino group now recovers its clear donating character (blue lobe) explaining the red‐shift. Data for the other compounds can be found in the Supporting Information.

Photostationary state distributions (PSDs) that can be achieved under irradiation with visible light are of crucial importance for applications, especially in biological context where the two isomers are expected to have different potency in, for example, binding to the cellular target.^[11]^ Only in very rare cases[^55^, ^56^] it is possible to design molecules in which the stable isomer is almost inactive at a given concentration, while the irradiation leads to the metastable isomer which is orders of magnitude more potent, thereby making the application virtually independent from PSD. In the majority of cases,[^9^, ^57^] the difference in potency is much less pronounced, requiring high efficiency for switching in both directions.

In series **1**–**10**, we observe (Figure 2, green pie charts) that under irradiation with green light (*λ*=526 nm) for switching in the forward (*trans* to *cis*) direction, a photostationary state with the distribution containing 46–62 % *cis* isomer can be attained. The only exceptions were compound **1** (likely due to the band overlap) and compound **10**, which is possibly due to short half‐life of the *cis* isomer, whose back‐isomerization competes with the photochemical transformation towards this isomer. However, as the use of red light is of much more biological relevance, we also evaluated the PSDs under *λ*=625 nm irradiation (Figure 2, red pie charts). To our delight, we observed distributions mostly exceeding 80 % *cis*, which is also consistent with negligible absorptivity of this isomer at wavelengths corresponding to red light. Again, lower values observed for compounds **1** and **10** can be explained by the substantial band overlap in this spectral region (Figure 2). Altogether, the limitation that remains to be solved for tetra‐*ortho*‐chloro‐azobenzenes, similarly to almost all available molecular photoswitches, is the overall low red‐light absorptivity of the *trans* form, which is one to two orders of magnitude lower than that of wavelengths corresponding to green light, leading to prolonged irradiation times^[37]^ and sometimes compromising the PSD in cases where fast thermal back‐isomerization of the metastable state is a competing process (for example, in the case of compound **10**).

In this context, the quantum yield of the forward isomerization becomes important, potentially determining the usefulness of red‐light operation of a photoswitch in a biological context. While in the studied series of molecules no general trends can be observed (*φ* ^t→c^=38±16 %) (Table 1), we note that quantum yields observed for compounds with strong electron‐donating substituents (compounds **1** and **2**, *φ* ^t→c^=34–63 %) are somewhat higher than for those with electron‐withdrawing groups (**9**,**10**, *φ* ^t→c^=18–20 %), although additional studies are still needed to confirm this trend. In a broader context of photochemical process efficiency, we also analysed the photoswitching cross section (Table 1) under green light irradiation (that is, the product of the quantum yield *φ* and molar attenuation coefficient *ϵ* at *λ*_exc_=526 nm, the maximum emission of the green LED used here). In general, values in the useful order of magnitude (10^2^–10^3^) were found, again with the strong EDG‐substituted compounds **1** and **2** showing the highest efficiency. The same trends are observed for irradiation with red light (*λ*_exc_=625 nm, Table 1), albeit with cross sections in the 10^0^–10^1^ order of magnitude.

The reverse (*cis* to *trans*) switching was studied by irradiation with blue light (*λ*_exc_=426 nm, Table 1). We were delighted to see that for most of the compounds it was possible to recover >80 % of the *trans* isomer (Figure 2, blue pie charts). This highlights the good dynamic range that can be achieved with tetra‐*ortho*‐chloro‐azobenzenes **2**–**9**, which can be switched between containing 82–90 % *trans* isomer under blue light irradiation and 83–90 % *cis* isomer under red light irradiation. Compound **10** features the best PSD under blue light (95 % *trans*), but its forward switching is less pronounced (see above). Strikingly, due to the overlap with a strong π–π* band in the blue region of the spectrum, the behavior of compound **1** is essentially inverted, as it can be most efficiently switched in the forward direction with blue light (66 % *cis* isomer) and in the reverse direction with green light (78 % *trans* isomer). Thanks to quantum yields exceeding 30 % and strong absorptivities of all the studied compounds at *λ*=426 nm, the reverse switching is an efficient process, with cross sections in the 10^3^–10^4^ order of magnitude.

The main motivation behind the introduction of tetra‐*ortho*‐substituted azobenzenes has been the possibility to achieve visible‐light‐switching without compromising the half‐life or the metastable isomer, which was the typical drawback of the more established azobenzene architectures substituted with both an electron‐withdrawing and electron‐donating substituent in the *para* positions (push–pull systems).^[29]^ Indeed, our data (Table 1) for the tetra‐*ortho*‐chloro‐azobenzenes confirm that for most of the studied *para*‐substituents (compounds **2**–**9**), the half‐life of the *cis* isomer is between 4 to 38 d, which for all practical purposes translates to bistable systems in biological applications, meaning that the thermal *cis*‐*trans* isomerisation can often be neglected for *σ*_p_ between −0.27 and 0.54. However, compounds with strongly electron‐donating and ‐withdrawing groups (such as compounds **1** and **10**) feature much lower stability of the *cis* isomer, an effect especially pronounced for compound **10**, where a half‐life of about 1 h was measured.

The thermal back‐isomerization in an azobenzene can typically take place through an inversion or a rotation mechanism, and both have been found here through theoretical investigation (Figure 3 A; Supporting Information, Table S1). For all investigated compound, the latter mechanism yields a more stabilized transition state and rotation is therefore the most favored pathway. We note that this mechanism comes with a rupture of the π bond, and we therefore used broken‐symmetry DFT to investigate it, which lead to the expected spin distribution (Figure 3 D). The theoretical back‐isomerization barriers are listed in Table 1 and it can be seen that they are significantly smaller than their experimental counterpart, but that the trends are nicely reproduced. Indeed, excluding the compound substituted with a CF_3_ group, we obtain a determination coefficient, *R* ^*2*^, between experiment [Δ*G* ^exp^] and theory [Δ*G* ^calc^] of 0.82. Compound **9** proved to be most difficult for theoretical assessment. At this stage, it might be interesting to take a specific look at azobenzenes **1** and **10**, as they are substituted with the prototypical strong donor (NMe_2_) and acceptor (NO_2_) groups. As might be appreciated, both groups experimentally show quite small and similar *t*_1/2_. However, the underlying reasons are different. Indeed, in compound **1**, the *cis* form is essentially non‐stabilized, with a relative free energy of 31.4 kJ mol^−1^ as compared to the *trans* form, which is much higher than in the non‐substituted case (26.3 kJ mol^−1^). The *cis*‐to‐*trans* barrier is small owing to this lack of stabilization (Supporting Information, Table S1). In contrast, for compound **10**, the relative free energy of *cis* as compared to the *trans* structure, 26.3 kJ mol^−1^, is essentially unchanged from the unsubstituted case, but the rotational transition state is itself much more stabilized (Supporting Information, Table S1). For this reason, a balance needs to be found between the relative stabilities of the two isomers and the stabilization of the rotational TS itself.

**Figure 4:**
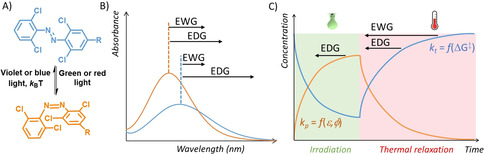
Design guidelines for tetra‐*ortho*‐chloro‐azobenzenes. Influence of the properties of substituents in the para position (black arrows) of the azobenzene (A) on the position of the bands of both isomers in the visible range of the spectrum (B) and on the kinetics of photoisomerisation (*k*_p_, which is a function of molar attenuation coefficient *ϵ* and quantum yield *φ*) and thermal reisomerisation *k*_t_ (C).

Altogether, the data presented herein enables the formulation of certain general rules for the design of tetra‐*ortho*‐chloro‐azobenzene photoswitches for specific applications (Figure 4). In situations where long half‐lives of the *cis* isomer are required, for example, when the effects of both isomers of a photopharmacological agent on a cell line for longer time are studied, the use of substituents from the middle of the Hammet *σ*_p_ scale is recommended (such as those in compounds **4** and **6**), as it provides the metastable state that persists for multiple weeks, similarly to those observed for hemiindigo photoswitches that also respond to red‐light irradiation.[^58^, ^59^] Conversely, when life‐times on the scale of hours are desired, as in the case of photoswitchable antibiotics that are activated prior to administration and then should spontaneously lose their activity,^[60]^ the strongly electron‐withdrawing groups (for example, compound **10**) are favored.

In photopharmacology, a few azobenzene‐based bioactive molecules have been described in which the *cis* isomer shows potency several orders of magnitude higher than *trans*.[^55^, ^56^] In such cases, the photostationary state distribution that one can achieve is of less importance, as even low concertation of the metastable state will result in localized activation. Here, the use of tetra‐*ortho*‐chloro‐azobenzenes with strong electron‐donating groups (for example, compound **1**) is recommended, as it offers the most efficient activation with visible light. However, such systems are so far scarce, and most often, the difference in potency of the photoisomers is limited, which requires that high photostationary states are achieved. Especially in these cases, the intermediate substituents (such as compounds **3**–**8**) should be considered.

## Conclusion

We present herein a systematic analysis of the photochromism of tetra‐*ortho*‐chloro‐azobenzenes, an emerging class of visible‐light operated photoswitches with great potential for use in biological and material sciences. Their versatility, underlined by the possibility to tune their photochemical properties towards the desired application, renders them a highly useful tool in a still limited repertoire of molecular photoswitches that respond to low energy green and red light. Detailed understanding of the influence that substituents play on key photochemical properties and thermal isomerization barriers, as presented here, will enable successful design of functional, photoresponsive systems. In a long‐term perspective, these insights provide a major step towards using light for the efficient regulation of biological processes with outstanding spatiotemporal precision.

## Conflict of interest

The authors declare no conflict of interest.

## Supporting information

As a service to our authors and readers, this journal provides supporting information supplied by the authors. Such materials are peer reviewed and may be re‐organized for online delivery, but are not copy‐edited or typeset. Technical support issues arising from supporting information (other than missing files) should be addressed to the authors.

Supplementary
