# Supplementary material for: General Principles for the Design of Visible‐Light‐Responsive Photoswitches: Tetra‐ortho‐Chloro‐Azobenzenes
Source: Angew Chem Int Ed Engl. 2020 Sep 23;59(48):21663–70. doi: 10.1002/anie.202008700 (PMC7756550; doi:10.1002/anie.202008700)
Supplement: Supplementary file 1 — Supplementary [file ANIE-59-21663-s001.pdf]

Angewandte Chemie
Eine Zeitschrift der Gesellschaft Deutscher Chemiker

Supporting Information

General Principles for the Design of Visible-Light-Responsive Photoswitches: Tetra-*ortho*-Chloro-Azobenzenes

Lucien N. Lameijer, Simon Budzak, Nadja A. Simeth, Mickel J. Hansen, Ben L. Feringa, Denis Jacquemin, and Wiktor Szymanski**

anie_202008700_sm_miscellaneous_information.pdf

Contents

Synthesis **3**

Photochemistry **9**

NMR experiments 9

Calculation of the UV/vis-spectra of the *cis*-species 10

^{1}H NMR irradiation experiments: PSS 526 nm (low concentration) & 426 nm, 526 nm & 652 nm (high concentration) 10

Thermal decay and Eyring plots 1 - 10 ..... **35**

Thermal decay plots for compound 1 36

Eyring plot for compound 1 38

Thermal decay plots for compound 2 39

Eyring plot for compound 2 41

Thermal decay plots for compound 3 42

Eyring plot for compound 3 44

Thermal decay plots for compound 4 44

Eyring plot for compound 4 47

Thermal decay plots for compound 5 47

Eyring plot for compound 5 50

Thermal decay plots for compound 6 50

Eyring plot for compound 6 53

Thermal decay plots for compound 7 54

Eyring plot for compound 7 56

Thermal decay plots for compound 8 57

Eyring plot for compound 8 59

Thermal decay plots for compound 9 60

Eyring plot for compound 9 63

Thermal decay plots for compound 10 63

Eyring plot for compound 10 66

Quantum Yield calculation **66**

Emission spectra 420, 445 & 535 LEDs & transmission spectrum band pass filter MV532/20 ..... **143**

NMR spectra (^{1}H NMR, ^{13}C APT, ^{19}F NMR) and FT-IR spectra 1 – 10 ..... **145**

Theoretical calculations **170**

References **173**

Synthesis

General. Reagents were purchased from Sigma-Aldrich, Boom & Combi-Blocks and used without further purification. Dry solvents were collected from a Pure Solve MD5 solvent dispenser from Demaco or by drying them for 48 h over fresh 3 Å molecular sieves. Flash chromatography was performed on silica gel (Screening devices B.V.) with a particle size of 40−64 μM and a pore size of 60 Å or on Buchi FlashPure silica columns (4 - 25 g, 40−63 μM, 60 Å) using a Buchi Reveleris® X2 system. TLC analysis was conducted on TLC aluminum foils with a silica gel matrix (Supelco, silica gel 60, 56524) with detection by UV absorption (254 nm), by spraying with a solution of $NH_4Mo_7O_{24}\cdot4H_2O$ (25 g · L^{-1}), $NH_4CeSO_4\cdot H_2O$ (10 g · L^{-1}), 10% H_2SO_4 in H_2O or $KMNO_4$ (7.5 g · L^{-1}), K_2CO_3 (50 g · L^{-1}) in aqueous NaOH (0.016M) followed by charring with a heat gun. Infrared spectra were recorded on a PerkinElmer UATR (Single Reflection Diamond) Spectrum Two device (4000−700 cm^{-1}; resolution 4 cm^{-1}). ^{1}H NMR and ^{13}C NMR were recorded in [D$_6$]DMSO, [D$_6$]Acetone and $CDCl_3$ with the chemical shift (δ) relative to the solvent peak on an Agilent Technologies 400-MR (400/54 Premium Shielded) spectrometer (400 MHz). All spectra were measured at room temperature (22–24 °C). High Resolution Mass Spectra were recorded on a Thermofisher LTQ Orbitrap XL with eluent MeOH (0.1% TFA) and flow rate of 0.15 mL · min^{-1} in positive (ACPI/ESI) mode. Melting point ranges were determined on a Stuart analogue capillary melting point SMP11 apparatus. All azobenzenes were acquired as mixtures of cis/trans, with only the *trans*-species reported. All *trans*-species were acquired by briefly (~10 s) heating the samples using a heat-gun in [D$_6$]DMSO, with the exception of **7**, which was heated for 5 seconds.

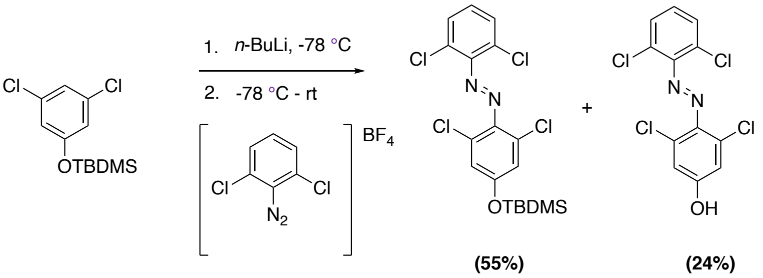

(55%) **(24%)**

2,2',4-(*O*-tert-butyldimethylsilyl)-6,6'-tetra-chloroazobenzene & 2,2',4-(hydroxyl)-6,6'-tetra-chloroazobenzene. 3-chloro-4-(*O*-tert-butyldimethylsilyl)-5-chlorobenzene[1] (1.00 g, 3.61 mmol) was dissolved under a dinitrogen atmosphere in dry THF (6 mL) in a flame-dried Schlenk-flask. The mixture was cooled to -78 °C and a solution of *n*-BuLi (2.3 mL, 3.7 mmol, 1.6 M in hexanes) was added dropwise over 5 minutes. This mixture was allowed to stir for 1 h, after which 2,6-dichlorobenzenediazonium tetrafluoroborate[2] (942 mg, 3.61 mmol) was added in one portion from which the color of the reaction changed to dark red. After vigorous stirring at -78 °C for 15 min, the suspension was slowly allowed to reach rt over 45 minutes and quenched upon the addition of aqueous $NaHCO_3$ (3 mL). The mixture was then diluted with EtOAc (100 mL) and transferred to a separatory funnel. After washing with brine (3x), the organic layer was dried (Na_2SO_4) and concentrated *in vacuo*. Purification of the residue by column chromatography (0 to 2% EtOAc in pentane) afforded 2,2',4-(*O*-*tert*-butyldimethylsilyl)-6,6'-tetra-chloroazobenzene (885 mg, 1.97 mmol, 55%) and 2,2',4-(hydroxyl)-6,6'-tetra-chloroazobenzene (296 mg, 0.88 mmol, 24%) both as red solids. R_f (-OTBDMS) = 0.56 (2% EtOAc in pentane); IR (neat) 2955, 2928, 2858, 1585, 1433; Mp = 74 – 75 °C; ^{1}H NMR (400 MHz, [D$_6$]DMSO) δ 7.66 (d, *J* = 8.4 Hz, 2H), 7.46 (t, *J* = 8.1 Hz, 1H), 7.06 (s, 2H), 3.34 (s, 6H), 0.84 (s, 9H); ^{13}C NMR (101 MHz, [D$_6$]DMSO) δ 159.9, 147.4, 137.9, 130.2, 129.7, 129.6, 125.6, 117.0, 25.8, -3.2; HRMS (ESI) m/z $[M+H]^+$ calcd for $C_{18}H_{20}Cl_4N_2OSiH$ 449.0172, found 449.0161.

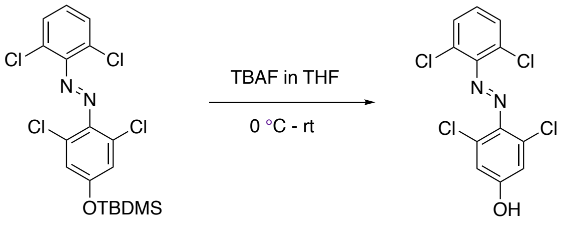

(89%)

2,2',4-(Hydroxy)-6,6'-tetra-chloroazobenzene. To a cooled (0 °C) solution of 2,2',4-(*O*-tert-butyldimethylsilyl)-6,6'-tetra-chloroazobenzene (212 mg, 0.470 mmol) in dry THF under dinitrogen was added a solution of TBAF in THF (600 μL, 0.600 mmol, 1M). The solution was allowed to stir at rt for 3 h after it was quenched upon the addition of MeOH (3 mL), diluted with EtOAc (50 mL) and transferred to a separatory funnel. After washing with 1 M HCl (1x) and brine (1x), the organic layer was dried (Na_2SO_4) and concentrated *in vacuo*. Purification of the residue by column chromatography (0 to 20% EtOAc in pentane) afforded the product as a dark red solid (142 mg, 0.420 mmol, 89%). R_f = 0.43 (20% EtOAc in pentane); IR (neat) 3644, 3330, 3140, 3089, 1567; Mp = 149 – 151 °C ^{1}H NMR (400 MHz, [D$_6$]DMSO) δ 11.18 (s, 1H), 7.65 (d, *J* = 8.0 Hz, 2H), 7.44 (t, *J* = 7.7 Hz, 1H), 7.05 (s, 2H); ^{13}C NMR (101 MHz, [D$_6$]DMSO) δ 121.6, 109.2, 99.7, 92.0, 91.3, 87.4, 78.8; HRMS (ESI) m/z $[M+H]^+$ calcd for $C_{12}H_7Cl_4N_2O$ 334.9307, found 334.9300.

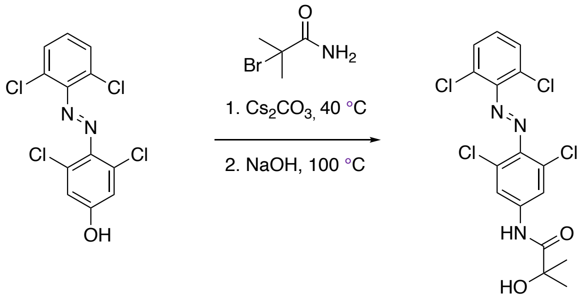

(78%)

4-(2-hydroxy-2-methylpropanamide)-2,2’,6,6’-tetra-chloroazobenzene. Adapted from a literature procedure.[3] 2,2’(4-hydroxy)-6,6’-*tetra*-chloroazobenzene (196 mg, 0.590 mmol) was dissolved in dry DMF (5.9 mL) and to this solution were added Cs_2CO_3 (616 mg, 1.89 mmol) and 2-bromo-2-methyl-propanamide (294 mg, 1.77 mmol) under a dinitrogen atmosphere. The reaction was allowed to stir for 48 h at 40 °C after which it was cooled down to rt, diluted with EtOAc (50 mL) and transferred to a separatory funnel. After washing with water (2x) and brine (1x), the organic layer was dried (Na_2SO_4) and concentrated *in vacuo*. The crude 4-(2-amino-2-methylpropanoate)-2,2’,6,6’-tetra-chloroazobenzene was then redissolved in dry DMF (2 mL), followed by addition of NaOH (192 mg, 4.80 mmol) and heating at 100 °C for 1 h under a dinitrogen atmosphere. The mixture was then allowed to cool to rt, diluted with EtOAc (50 mL) and transferred to a separatory funnel. After washing with water (1x), 1 M aqueous HCl (1x) and brine (1x), the organic layer was dried (Na_2SO_4) and then concentrated *in vacuo*. Purification of the crude product by silica column chromatography (0 to 15% EtOAc in pentane) afforded the title compound as a red solid (194 mg, 0.46 mmol, 78% over two steps): R_f = 0.32 (20% EtOAc in pentane); IR (neat) 3345, 2984, 1664, 1571, 1514; Mp = 152 – 153 °C; ^{1}H NMR (400 MHz, $CDCl_3$) δ 9.07 (s, 1H), 7.82 (s, 2H), 7.43 (d, *J* = 8.1 Hz, 2H), 7.22 (t, *J* = 8.1 Hz, 1H), 2.07 (s, 1H), 1.54 (s, 6H); ^{13}C NMR (101 MHz, $CDCl_3$) δ 174.6, 148.2, 143.1, 139.0, 129.5, 129.2, 127.2, 120.0, 28.1; HRMS (ESI) m/z $[M+H]^+$ calcd for $C_{16}H_{14}Cl_4N_3O_2$ 419.9835, found 419.9828.

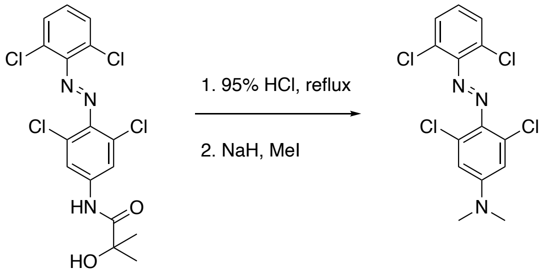

1
(49%)

4-(*N,N*-dimethylamino)-2,2’,6,6’-tetra-chloroazobenzene, 1. 4-(2-hydroxy-2-methylpropanamide)-2,2’,6,6’-tetra-chloroazobenzene (174 mg, 0.41 mmol) was dissolved in 1,4-dioxane (2.5 mL) and to this solution was added concentrated (95 %) HCl (2.5 mL). The resulting mixture was heated at 95 °C for 1.5 h after which it was cooled down to rt and concentrated *in vacuo.* The residue was then redissolved in EtOAc (50 mL) and transferred to a separatory funnel. After washing with 1M NaOH (2x) and brine (1x), the organic layer was dried (Na_2SO_4) and then concentrated *in vacuo*. The residue was redissolved in dry THF (5 mL) and cooled to 0 °C, followed by the addition of 60% NaH in mineral oil (117 mg, 2.92 mmol). After stirring for 10 minutes, MeI (140 µL, 2.76 mmol) was added dropwise and the mixture was allowed to stir 4.5 h and warm up to rt. The reaction was then quenched upon the addition of MeOH (2 mL), diluted with EtOAc (50 mL) and transferred to a separatory funnel. After washing with 1 M NaOH (2x) and brine (1x), the layers were separated, the organic layer was dried (Na_2SO_4) and concentrated *in vacuo*. Purification of the crude by silica column chromatography (0 to 5% EtOAc in pentane) afforded the title compound as a red solid (71 mg, 0.20 mmol, 49% over two steps): R_f = 0.68 (20% EtOAc in pentane); IR (neat) 2927, 2814, 1509, 1430, 1062; Mp = 159 – 161 °C; ^{1}H NMR (400 MHz, [D$_6$]DMSO) δ 7.60 (d, *J* = 8.1 Hz, 1H), 7.37 (dd, *J* = 8.5, 7.7 Hz, 1H), 6.88 (s, 1H), 3.07 (s, 3H); ^{13}C NMR (101 MHz, [D$_6$]DMSO) δ 151.5, 148.0, 133.4, 131.4, 129.4, 129.2, 125.6, 112.1, 40.2, 39.9, 39.8; HRMS (ESI) m/z $[M+H]^+$ calcd for $C_{14}H_{12}Cl_4N_3$ 361.9780, found 361.9773.

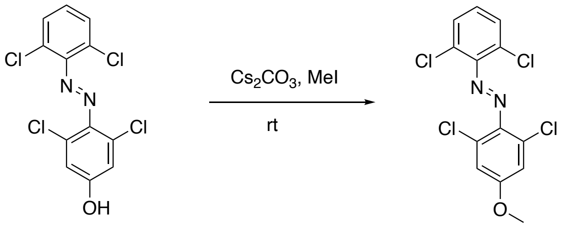

2
(94%)

2,2’,4-(*O*-methyl)-6,6’-tetra-chloroazobenzene, 2. To a solution of 2,2’,4-(hydroxy)-6,6’-tetra-chloroazobenzene (105 mg, 0.310 mmol) in dry acetone (3 mL) were added dry Cs_2CO_3 (125 mg, 0.380 mmol) and MeI (20.0 µL, 0.320 mmol) under a dinitrogen atmosphere. The mixture was allowed to stir at rt overnight after which it was filtered over a glass filter and concentrated *in vacuo*. The residue was dissolved in EtOAc (50 mL) and washed with 1 M HCl (1x), aqueous $NaHCO_3$ (1x) and brine (1x), and the organic layer was dried (Na_2SO_4) and concentrated *in vacuo*, affording the title compound as a red solid (102 mg, 0.290 mmol, 94%): R_f = 0.30 (2% EtOAc in pentane); IR (neat) 3104, 2925, 2854, 1587; Mp = 74 – 76 °C; ^{1}H NMR (400 MHz, [D$_6$]DMSO) δ 7.66 (d, *J* = 7.7 Hz, 2H), 7.46 (t, *J* = 7.2 Hz, 1H), 7.32 (s, 2H), 3.89 (s, 3H); ^{13}C NMR (101 MHz, [D$_6$]DMSO) δ 160.6, 147.2, 139.3, 130.5, 129.8, 129.0, 125.6, 115.9, 56.7; HRMS (ESI) m/z [M+H]$^+$ calcd for $C_{13}H_9Cl_4N_2$ 348.9464, found 348.9460.

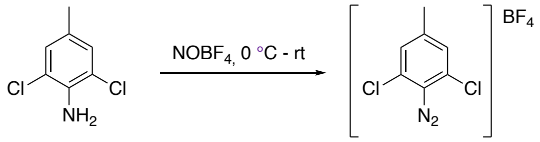

(89%)

2,6-dichloro-4-methylbenzenediazonium tetrafluoroborate. 2,6-dichloro-4-methylaniline (516 mg, 2.93 mmol) was dissolved in dry Et_2O (4.0 mL) and cooled to 0 °C. To this solution was added, in portions, $NOBF_4$ (95%, 360 mg, 2.93 mmol). After stirring for 1.5 h the suspension, was filtered over a glass filter and washed carefully with a minimal amount of cold Et_2O (2x). After drying for 1 h, the product was afforded as a white powder (716 mg, 2.60 mmol, 89%). IR (neat): 3136, 3070, 2264, 1557, 1021; ^{1}H NMR (400 MHz, [D$_6$]Acetone) δ 8.10 (s, 1H), 2.73 (d, *J* = 0.7 Hz, 4H); ^{19}F NMR (376 MHz, [D$_6$]Acetone) δ -151.80.

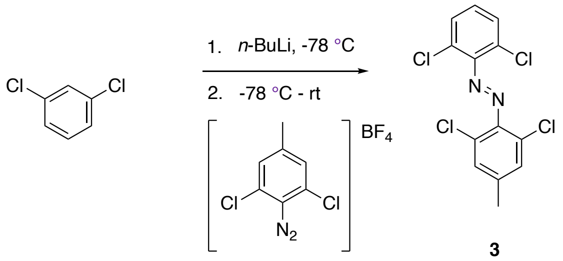

3
(90%)

2,2’,4-(methyl)-6,6’-tetra-chloroazobenzene, 3. 1,3-dichlorobenzene (300 µL, 2.63 mmol) was dissolved under a dinitrogen atmosphere in dry THF (5.0 mL) in a flame-dried Schlenk-flask. The mixture was cooled to -78 °C and a solution of *n*-BuLi (1.64 mL, 2.63 mmol, 1.6 M in hexanes) was added dropwise over 5 minutes. This mixture was allowed to stir for 1 h, after which 2,6-dichloro-4-methylbenzenediazonium tetrafluoroborate (711 mg, 2.59 mmol) was added in one portion from which the color of the reaction turned dark red. After vigorous stirring at -78 °C for 15 min, the suspension was slowly allowed to reach rt, followed by stirring for another 45 minutes after which it was quenched upon the addition of aqueous $NaHCO_3$ (3 mL). The mixture was then diluted with EtOAc (100 mL) and transferred to a separatory funnel. After washing with brine (3 x), the layers were separated, and the organic layer was dried (Na_2SO_4) and concentrated *in vacuo*. Purification of the residue by column chromatography (0 to 3% EtOAc in pentane) afforded the title compound as a dark red powder (779 mg, 2.33 mmol, 90%). R_f = 0.71 (2% EtOAc in pentane); Mp = 115 – 118 °C; ^{1}H NMR (400 MHz, [D$_6$]DMSO) δ 7.68 (d, *J* = 8.4 Hz, 2H), 7.55 (s, 2H), 7.49 (t, *J* = 7.7 Hz, 1H), 2.38 (s, 3H). All other analytical data were in agreement with data reported previously.[4]

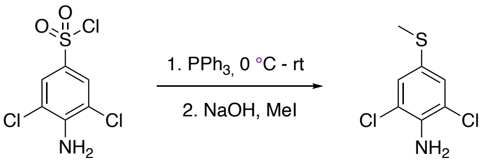

(30%)

2,6-dichloro-4-(methylthio)aniline. To the ice-cooled solution of triphenylphosphine (10.4 g, 39.7 mmol) in dry toluene was added *p*-amino-3,5-dichlorobenzenesulfonyl chloride (4.00 g, 13.2 mmol). The dark yellow solution was allowed to stir for 1 h after which it was quenched upon the addition of ~20 mL water. The mixture was transferred to a separatory funnel and the aqueous layer was discarded, while the organic layer was extracted with 1M NaOH (3x). The alkaline solution was then washed with EtOAc (2x), acidified with 1M HCl and extracted with DCM (3x). The organic layer was dried (Na_2SO_4) and concentrated *in vacuo,* affording *p*-amino-3,5-dichlorobenzenethiol as a light yellow solid (1.02 g, 5.26 mmol). Without further purification, this solid was then dissolved in a mixture of MeOH/H_2O (10 mL, 1:1) to which NaOH (254 mg, 6.35 mmol) and MeI (395 µL, 6.34 mmol) were added. After stirring overnight at rt, the mixture was filtered over a glass filter and concentrated *in vacuo*. The residue was redissolved in EtOAc (50 mL) and washed with aqueous $NaHCO_3$ (1x), water (1x) and brine (1x). The organic layer was dried (Na_2SO_4) and concentrated *in vacuo*, affording the title compound as a beige solid (830 mg, 3.90 mmol, 30% over two steps): IR (neat) 3426, 3302, 2916, 1615, 1468; Mp = 58 – 60 °C; ^{1}H NMR (400 MHz, $CDCl_3$) δ 7.19 (s, 2H), 4.42 (s, 2H), 2.42 (s, 3H); HRMS (ESI) m/z [M+H]$^+$ calcd for $C_7H_8Cl_2NS$ 208.9783, found 208.9635.

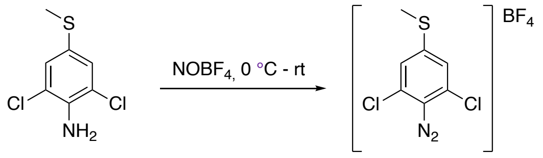

(66%)

2,6-dichloro-4-(methylthio)benzenediazonium tetrafluoroborate. To the ice-cooled solution of 2,6-dichloro-4-(methylthio)aniline (513 mg, 2.47 mmol) in Et_2O (2.0 mL), was added, in portions, $NOBF_4$ (95%, 289 mg, 2.47 mmol). After stirring for 1 h the suspension was filtered over a glass filter and carefully washed with cold Et_2O (3x). After drying under a high vacuum for 1 h, the title compound was afforded as a yellow powder (503 mg, 1.64 mmol, 66%). ^1H NMR (400 MHz, [D6]Acetone) δ 8.04 (s, 2H), 2.91 (s, 3H); ^{19}F NMR (376 MHz, [D6]Acetone) δ -151.80.

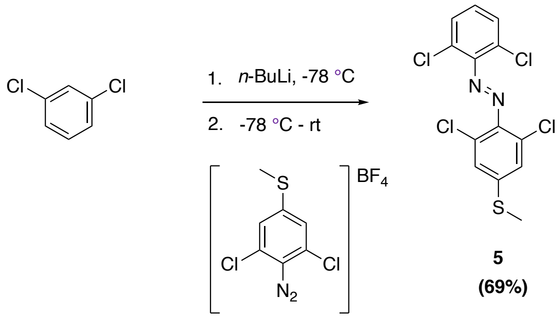

5
(69%)

4-(methylthio)-2,2',6,6'-tetra-chloroazobenzene, 5. 1,3-dichlorobenzene (380 mg, 1.24 mmol) was dissolved under a dinitrogen atmosphere in dry THF (2.5 mL) in a flame-dried Schlenk-flask. The mixture was cooled to -78 °C and a solution of *n*-BuLi (775 µL mL, 1.24 mmol, 1.6 M in hexanes) was added dropwise over 5 minutes. This mixture was allowed to stir for 1 h, after which 2,6-dichloro-4-(methylthio)benzenediazonium tetrafluoroborate (380 mg, 1.24 mmol) was added in one portion, upon which the color of the reaction slowly changed to red. After vigorous stirring at -70 °C for 20 min, the suspension was slowly allowed to reach rt over 40 minutes and quenched by the addition of aqueous $NaHCO_3$ (3 mL). The mixture was then diluted with EtOAc (100 mL) and transferred to a separatory funnel. After washing with brine (3x), the organic layer was dried (Na_2SO_4) and concentrated *in vacuo*. Purification of the residue by column chromatography (0 to 2% EtOAc in pentane) afforded **5** as a purple solid (313 mg, 0.860 mmol, 69%). R_f = 0.24 (2% EtOAc in pentane); IR (neat) 3069, 2928, 1568, 1432; Mp = 106 – 108 °C ^1H NMR (400 MHz, [D6]DMSO) δ 7.69 (s, 2H), 7.57 (d, *J* = 1.3 Hz, 2H), 7.49 (t, *J* = 8.1 Hz, 1H), 2.61 (s, 3H); ^{13}C NMR (101 MHz, [D6]DMSO) δ 147.0, 144.6, 142.3, 127.7, 125.8, 14.3; HRMS (ESI) m/z $[M+H]^+$ calcd for $C_{13}H_9Cl_4N_2S$ 364.9235, found 364.9230.

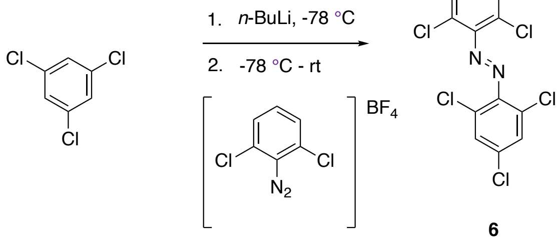

6
(26%)

2,2',4,6,6'-penta-chloroazobenzene, 6. 1,3,5-trichlorobenzene (689 mg, 3.80 mmol) was dissolved under a dinitrogen atmosphere in dry THF (6 mL) in a flame-dried Schlenk-flask. The mixture was cooled to -78 °C and a solution of *n*-BuLi (2.4 mL, 3.8 mmol, 1.6M in hexanes) was added dropwise over 5 minutes. This mixture was allowed to stir for 1 h, after which 2,6-dichlorobenzenediazonium tetrafluoroborate (990 mg, 3.80 mmol) was added in one portion, upon which the color of the reaction changed to dark red. After vigorous stirring at -78 °C for 15 min, the suspension was slowly allowed to reach rt over 45 minutes and quenched by the addition of aqueous $NaHCO_3$ (3 mL). The mixture was then diluted with EtOAc (100 mL) and transferred to a separatory funnel. After washing with brine (3x), the organic layer was dried (Na_2SO_4) and concentrated *in vacuo*. Purification of the residue by column chromatography (0 to 2% EtOAc in pentane) afforded the product as a red solid (353 mg, 1.00 mmol, 26%): R_f = 0.23 (5% EtOAc in pentane); IR (neat) 3082, 2926, 2853, 1562; Mp = 113 – 115 °C; ^1H NMR (400 MHz, [D6]DMSO) δ 7.94 (s, 2H), 7.70 (d, *J* = 8.3 Hz, 2H), 7.52 (dd, *J* = 8.6, 7.7 Hz, 1H); ^{13}C NMR (101 MHz, [D6]DMSO) δ 146.5, 145.4, 134.7, 131.5, 130.0, 129.7, 127.1, 126.1; HRMS (ESI) m/z $[M+H]^+$ calcd for $C_{12}H_6Cl_5N_2$ 352.8968, found 352.8961.

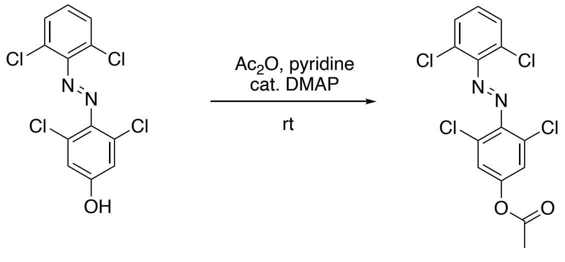

7
(100%)

2,2',4-(*O*-acetyl)-6,6'-tetra-chloroazobenzene. 2,2',4-(hydroxy)-6,6'-tetra-chloroazobenzene (119 mg, 0.350 mmol) was dissolved in dry pyridine (3.5 mL) under a dinitrogen atmosphere, and to this mixture were added Ac_2O (400 µL, 4.23 mmol) and a catalytic amount of *N,N*-dimethylaminopyridine. The mixture was then allowed to stir overnight at rt, after which it was diluted with EtOAc (50 mL) and transferred to a separatory funnel. After washing with 1M HCl (1x), aqueous $NaHCO_3$ (1x) and brine, the organic layer was dried (Na_2SO_4) and concentrated *in vacuo* affording the title compound as a red solid (132 mg, 0.350 mmol, quant.): R_f = 0.43 (5% EtOAc in pentane); IR (neat) 3086, 2924, 2856, 1763, 1567; Mp = 120 – 121 °C; ^{1}H NMR (400 MHz, [D$_6$]DMSO) δ 7.71 (s, 2H), 7.68 (s, 2H), 7.53 (t, *J* = 8.2 Hz, 1H), 2.32 (s, 3H); ^{13}C NMR (101 MHz, [D$_6$]DMSO) δ 168.72, 150.9, 146.6, 144.1, 131.3, 130.0, 126.8, 126.0, 124.0, 20.8; HRMS (ESI) m/z [M+H]$^+$ calcd for $C_{14}H_9Cl_4N_2O_2$ 376.9413, found 376.9408.

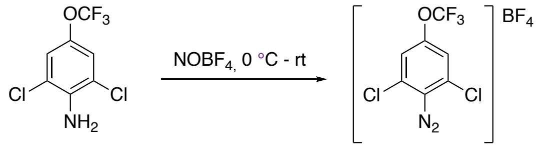

(54%)

2,6-dichloro-4-(trifluoromethoxy)benzenediazonium tetrafluoroborate. To an ice-cooled solution of 2,6-dichloro-4-(trifluoromethoxy)aniline (789 mg, 3.21 mmol) in Et_2O (3.0 mL), was added in portions, $NOBF_4$ (95%, 395 mg, 3.21 mmol). After stirring for 1 h, the suspension was filtered over a glass filter and carefully washed with cold Et_2O (3x). After drying under a high vacuum for 1 h, the title compound was afforded as a white powder (599 mg, 1.74 mmol, 54%). ^{1}H NMR (400 MHz, [D$_6$]Acetone) δ 8.27 (s, 2H); ^{19}F NMR (376 MHz, [D$_6$]Acetone) δ -58.40, -151.79.

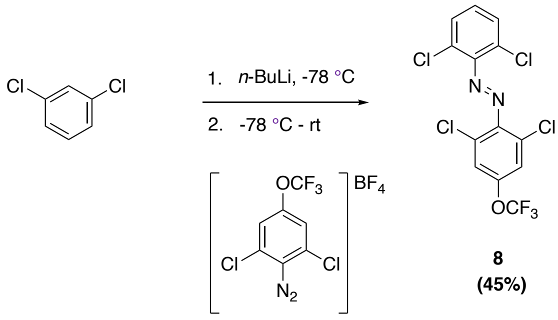

8
(45%)

2,2',4-(trifluoromethoxy)-6,6'-tetra-chloroazobenzene, 8. 1,3-dichlorobenzene (150 µL, 1.32 mmol) was dissolved under a dinitrogen atmosphere in dry THF (4.0 mL) in a flame-dried Schlenk-flask. The mixture was cooled to -78 °C and a solution of *n*-BuLi (850 µL, 1.36 mmol, 1.6M in hexanes) was added dropwise over 5 minutes. This mixture was allowed to stir for 1 h, after which 2,6-dichloro-4-(trifluoromethoxy)benzenediazonium tetrafluoroborate (454 mg, 1.32 mmol) was added in one portion, upon which the color of the reaction quickly changed to light red. After vigorous stirring at -78 °C for 5 min, the suspension was allowed to reach rt and quenched by the addition of aqueous $NaHCO_3$ (3 mL). The mixture was then diluted with EtOAc (100 mL) and transferred to a separatory funnel. After washing with brine (2x), the organic layer was dried (Na_2SO_4) and concentrated *in vacuo*. Purification of the residue by column chromatography (0 to 3% EtOAc in pentane) afforded the product as a red oil (244 mg, 0.600 mmol, 45%): R_f = 0.55 (4% EtOAc in pentane); IR (neat) 3086, 1588, 1563, 1434; Mp = 71 – 73 °C; ^{1}H NMR (400 MHz, [D$_6$]DMSO) δ 7.92 (s, 2H), 7.72 (dd, *J* = 8.2, 1.6 Hz, 2H), 7.53 (td, *J* = 8.2, 1.6 Hz, 1H); ^{13}C NMR (101 MHz, [D$_6$]DMSO) δ 148.4, 146.8, 146.2, 132.1, 130.5, 127.7, 126.6, 123.6; HRMS (ESI) m/z [M+H]$^+$ calcd for $C_{13}H_6Cl_4N_2O$ 402.9181, found 402.9178.

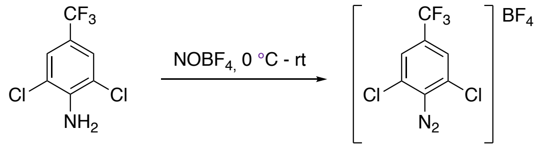

(70%)

2,6-dichloro-4-(trifluoromethyl)benzenediazonium tetrafluoroborate. To an ice-cooled solution of 2,6-dichloro-4-(trifluoromethyl)aniline (1.05 g, 4.57 mmol) in Et_2O (6.0 mL), was added, in portions, $NOBF_4$ (95%, 576 mg, 4.68 mmol). After stirring for 1 h, the suspension was filtered over a glass filter and carefully washed with cold Et_2O (2x). After drying under a high vacuum for 1 h, the title compound was afforded as a white powder (1.05 g, 3.19 mmol, 70%). ^1H NMR (400 MHz, [D$_6$]Acetone) δ 8.69 (s, 1H); ^{19}F NMR (376 MHz, [D$_6$]Acetone) δ -64.68, -151.74.

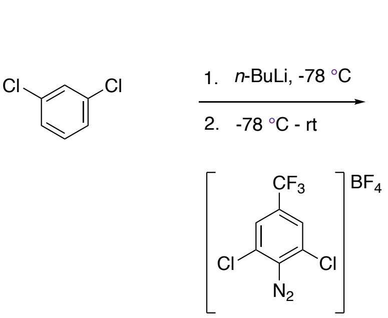

9
(80%)

2,2',4-(trifluoromethyl)-6,6'-tetra-chloroazobenzene, 9. 1,3-dichlorobenzene (200 µL, 1.75 mmol) was dissolved under a dinitrogen atmosphere in dry THF (8.0 mL) in a flame-dried Schlenk-flask. The mixture was cooled to -78 °C and a solution of *n*-BuLi (1.1 mL, 1.76 mmol, 1.6M in hexanes) was added dropwise over 5 minutes. This mixture was allowed to stir for 1 h, after which 2,6-dichloro-4-(trifluoromethyl)benzenediazonium tetrafluoroborate (579 mg, 1.76 mmol) was added in one portion, upon which the color of the reaction quickly changed to red. After vigorous stirring at -78 °C for 15 min, the suspension was allowed to reach rt and quenched upon the addition of aqueous $NaHCO_3$ (3 mL). The mixture was then diluted with EtOAc (100 mL) and transferred to a separatory funnel. After washing with brine (3x), the organic layer was dried (Na_2SO_4) and concentrated *in vacuo*. Purification of the residue by column chromatography (100% pentane) afforded the product as a red solid (548 mg, 1.41 mmol, 80%): IR (neat) 3082, 1565, 1434, 1390, 1306; Mp = 115 – 116 °C; ^1H NMR (400 MHz, , [D$_6$]DMSO) δ 8.21 (s, 1H), 7.75 (dd, *J* = 8.1, 1.4 Hz, 1H), 7.57 (ddd, *J* = 8.8, 7.6, 1.3 Hz, 1H); ^{13}C NMR (101 MHz, , [D$_6$]DMSO) δ 146.1, 134.9, 132.0, 130.7, 130.2, 127.1, 126.5; ^{19}F NMR (376 MHz, [D$_6$]DMSO) δ -61.28; HRMS (ESI) m/z $[M+H]^+$ calcd for $C_{13}H_6Cl_4F_3N_2$ 386.9232, found 386.9226.

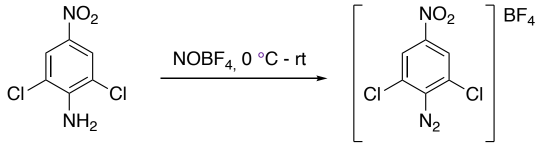

(60%)

2,6-dichloro-4-(nitro)benzenediazonium tetrafluoroborate. To an ice-cooled solution of 2,6-dichloro-*p*-nitroaniline (338 mg, 2.60 mmol) in Et_2O (5.0 mL), was added in portions, $NOBF_4$ (95%, 314 mg, 2.68 mmol). After stirring for 1 h, the suspension was filtered over a glass filter and carefully washed with cold Et_2O (3x). After drying under a high vacuum for 1 h, the title compound was afforded as a white powder (435 mg, 1.55 mmol, 60%). ^1H NMR (400 MHz, [D$_6$]Acetone) δ 8.04 (s, 2H), 2.91 (s, 3H); ^{19}F NMR (376 MHz, [D$_6$]Acetone) δ -151.80.

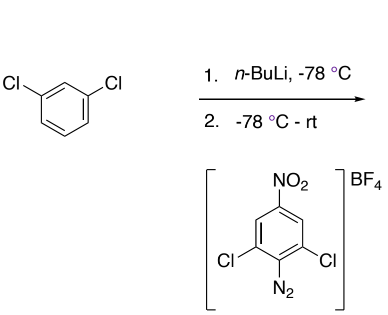

10
(56%)

2,2',4-(nitro)-6,6'-tetra-chloroazobenzene, 10. 1,3-dichlorobenzene (94.0 µL, 1.29 mmol) was dissolved under a dinitrogen atmosphere in dry THF (5.0 mL) in a flame-dried Schlenk-flask. The mixture was cooled to -78 °C and a solution of *n*-BuLi (505 µL, 0.808 mmol, 1.6M in hexanes) was added dropwise over 5 minutes. This mixture was allowed to stir for 1 h, after which 2,6-dichloro-4-(trifluoromethyl)benzenediazonium tetrafluoroborate (579 mg, 1.76 mmol) was added in

one portion, upon which the color of the reaction quickly changed to red. After vigorous stirring at -78 °C for 20 min, the suspension was allowed to reach rt and quenched by the addition of aqueous $NaHCO_3$ (3 mL). The mixture was then diluted with EtOAc (100 mL) and transferred to a separatory funnel. After washing with brine (2x), the layers were separated, and the organic layer was dried (Na_2SO_4) and concentrated *in vacuo*. Purification of the residue by column chromatography (0 to 10% EtOAc in pentane) afforded the product as a pale red solid (165 mg, 0.450 mmol, 56%): R_f = 0.76 (5% EtOAc in pentane); IR (neat) 3091, 2867, 1529, 1436, 1339; Mp = 140 – 142 °C; ^{1}H NMR (400 MHz, [D$_6$]DMSO) δ 8.57 (s, 2H), 7.76 (d, *J* = 8.0 Hz, 2H), 7.63 – 7.55 (m, 1H); ^{13}C NMR (101 MHz, [D$_6$]DMSO) δ 151.0, 147.3, 145.9, 132.3, 130.3, 126.7, 126.2, 125.2; HRMS (ACPI) m/z $[M+H]^+$ calcd for $C_{12}H_6Cl_4N_3O_2$ 363.9209, found 363.9192.

Photochemistry

For a typical experiment, a stirred solution (2 mL) of a compound (c = 200 μM) in DMSO was irradiated from the side in a fluorescence quartz cuvette (width = 1.0 cm) using a custom-built (Prizmatix/Mountain Photonics) multi-wavelength fiber coupled LED-system (FC6-LED-WL) using the following LEDs: 365A, 390B, 420Z, 445B, 535R, 630CA. The FWHM was ≤ 20 nm with the exception of the 535R LED (FWHM = 90 nm) and 630CA (FWHM = 21 nm). A lower FWHM for the 535R LED was achieved by connecting the LED through a polymer optic fiber to two collimators (2 x PRI FCM1-0.6) with a filter holder (PRI Filter holder) containing a 1" band pass filter (Chroma Narrow Green MV532/20). All LEDs were connected through a 7 to 1 fiber bundle attached to a 3 mm liquid light guide (LLG-3) and a liquid light guide adapter (LLG-AC). The adapter was placed in a Thorlabs SMR1 lens mount, which was adjusted to height using Thorlabs TR20/30 optical posts, AS6M4M adapters and a PJ302/M Offset Mounting Post Joist. The LEDs were controlled automatically via the built-in USB-controller using FC-LED-Ctrl 3.0 & Pulover's MacroCreator 5.05. Power measurements were carried out using a Thorlabs PM160T directly after the cell holder. For all kinetic experiments the temperature was maintained at 293K using a Quantum Northwest TC1 temperature controller. Electronic absorption spectra were measured using an Agilent 8453 at intervals of 0.5 s. Raw data was processed using Agilent UV-Vis ChemStation B.02.01 SP1, Spectragryph 1.2 and Origin 2018.

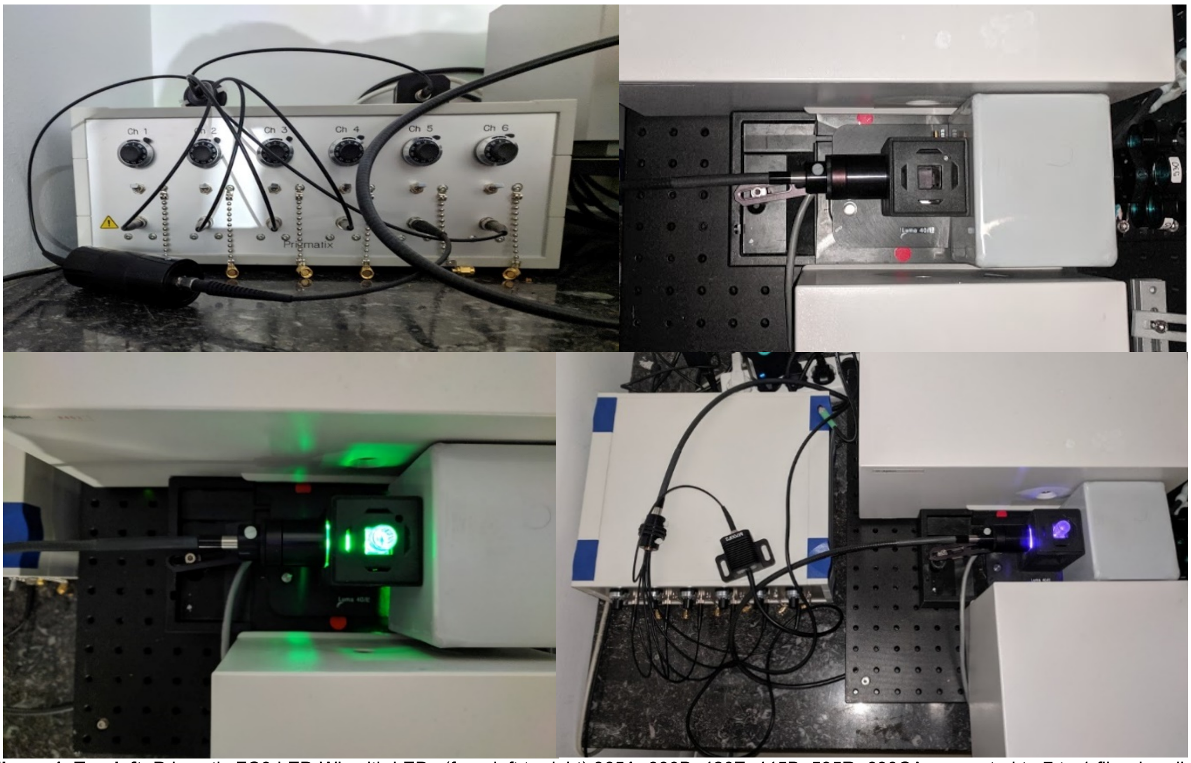

Figure 1. Top left: Prizmatix FC6-LED-WL with LEDs (from left to right) 365A, 390B, 420Z, 445B, 535R, 630CA connected to 7 to 1 fiber bundle. The 535R LED is connected through 2 FCM1-0.6 collimators and a filter holder containing a Chroma Narrow GreenMV532/20 band pass filter. The 7th leg of the fiber bundle is connected to an external Thorlabs LED with SMA connector. **Top right**: Fiber bundle connected to liquid light guide (LLG-3) and a LLG-AC liquid light guide adapter. **Bottom left**: Irradiation of fluorescence cuvette with 535 nm LED–without filter-. **Bottom right**: Irradiation of fluorescence cuvette with 420 nm LED.

NMR experiments

For a typical experiment, 1.0 – 4.0 mg of compound was dissolved in 0.6 mL [D$_6$]DMSO and irradiated at rt with either a Sahlmann cooled 3 x Roithner SMB-1N 430h (426 nm, FWHM = 16 nm, power output = 600 mW), a Sahlmann cooled 3 x LXML PM01 0100 (526 nm, FWHM = 35.1 nm, power output = 810 mW) or with a Sahlmann cooled 3 x Roithner VL-400-Emitter (652 nm, FWHM = 26.4 nm, power output = 1200 mW) for 1 and 4 h respectively until reaching PSS. ^{1}HNMR were then taken immediately after irradiation from which cis/trans-ratios could be determined.

Calculation of the UV/vis-spectra of the *cis*-species

In a typical experiment, a solution of compound **1** - **10** was prepared by preparing a solution of 200 to 300 µM in [D$_6$]DMSO (V = 3.0 mL) in 3 mL fluorescence quartz cuvette. While stirring, this sample was irradiated using the 535R LED from the custom-built irradiation setup without a band pass filter until a photostationary state (PSS) was reached. Electronic absorbance spectra were recorded during this irradiation. After reaching the PSS, an aliquot (0.3 mL) of the sample was taken and measured by NMR (^{1}H NMR), from which the *cis/trans* ratio was determined. The absorbance of the *cis*-species throughout the spectrum was then calculated using the following formula:

$$A_{cis} = \frac{A_{PSS} - \left(A_{trans} * \left(\% \frac{trans}{100}\right)\right)}{\left(\% \frac{cis}{100}\right)}$$

Molar extinction coefficients for both species (*cis* and *trans*) were then calculated using the Lambert-Beer Law, A = εcl, in which ε is the molar extinction coefficient (M^{-1} cm^{-1}), c is the concentration (M) and l is the path-length (cm).

^{1}H NMR irradiation experiments: PSS 526 nm (low concentration) & 426 nm, 526 nm & 652 nm (high concentration)

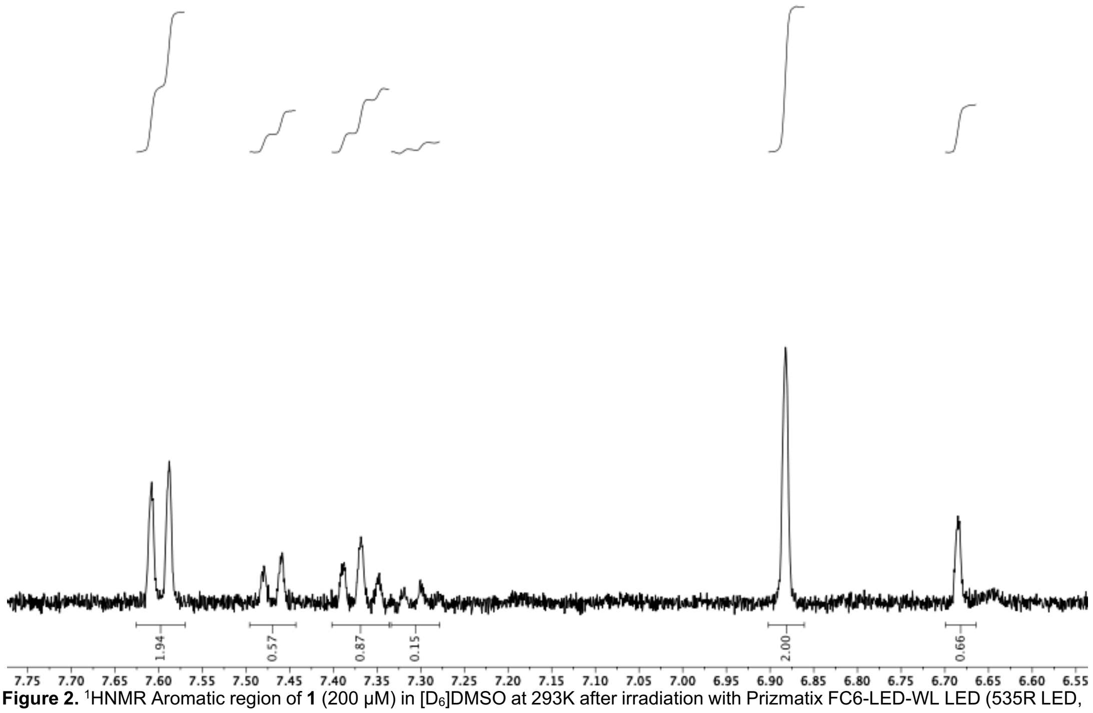

Figure 2. ^{1}HNMR Aromatic region of **1** (200 µM) in [D$_6$]DMSO at 293K after irradiation with Prizmatix FC6-LED-WL LED (535R LED, FWHM = 90 nm) until reaching the photostationary state. Data was processed with MestreNova 12.

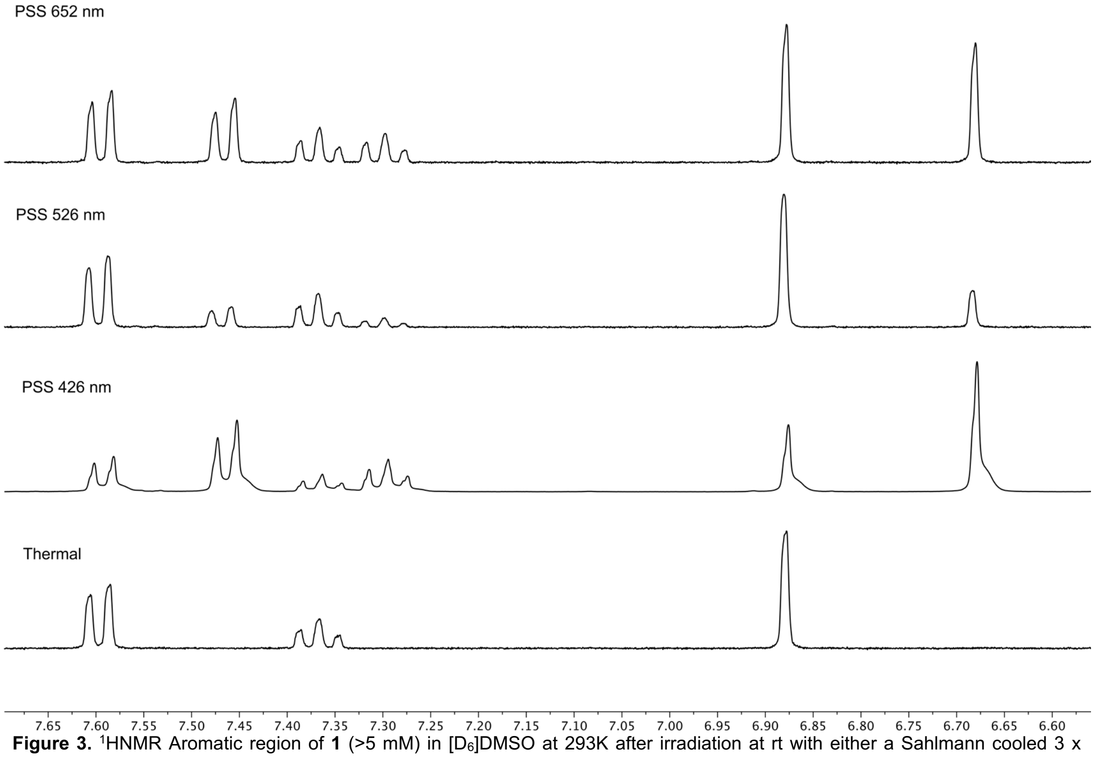

Figure 3. ^{1}HNMR Aromatic region of **1** (>5 mM) in [D$_6$]DMSO at 293K after irradiation at rt with either a Sahlmann cooled 3 x Roithner VL-400-Emitter (652 nm, FWHM = 26.4 nm, power output = 1200 mW), a Sahlmann cooled 3 x LXML PM01 0100 (526 nm, FWHM = 35.1 nm, power output = 810 mW), or a Sahlmann cooled 3 x Roithner SMB-1N 430h (426 nm, FWHM = 16 nm, power output = 600 mW).

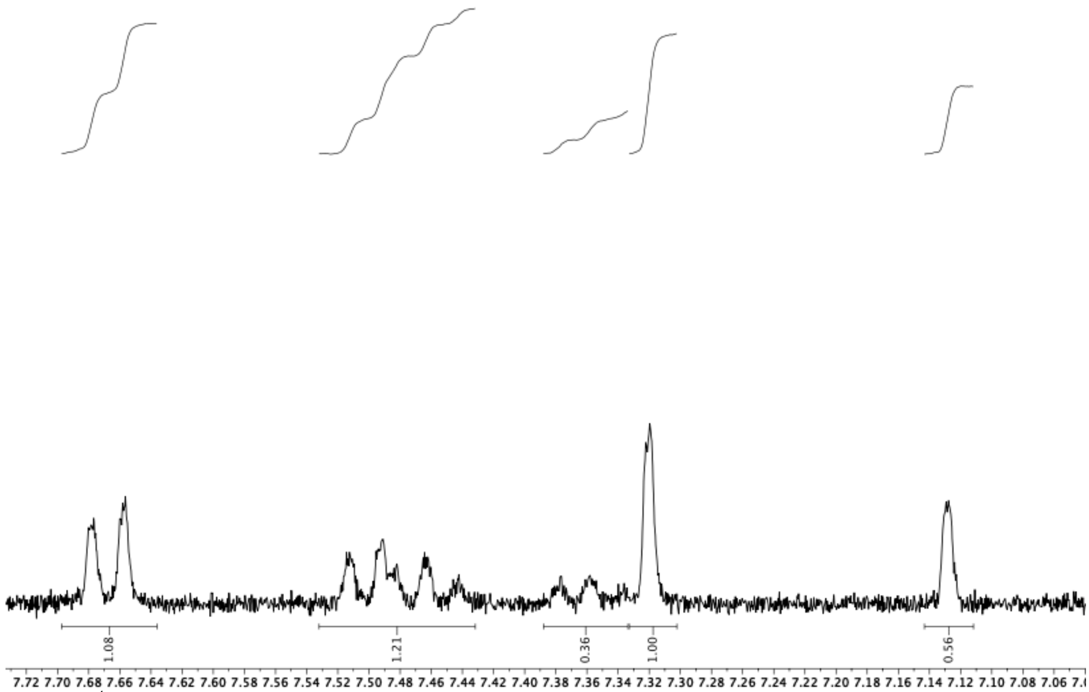

Figure 4. ^{1}HNMR Aromatic region of **2** (300 µM) in [D$_6$]DMSO at 293K after irradiation with Prizmatix FC6-LED-WL LED (535R LED, FWHM = 90 nm) until reaching the photostationary state. Data was processed with MestreNova 12.

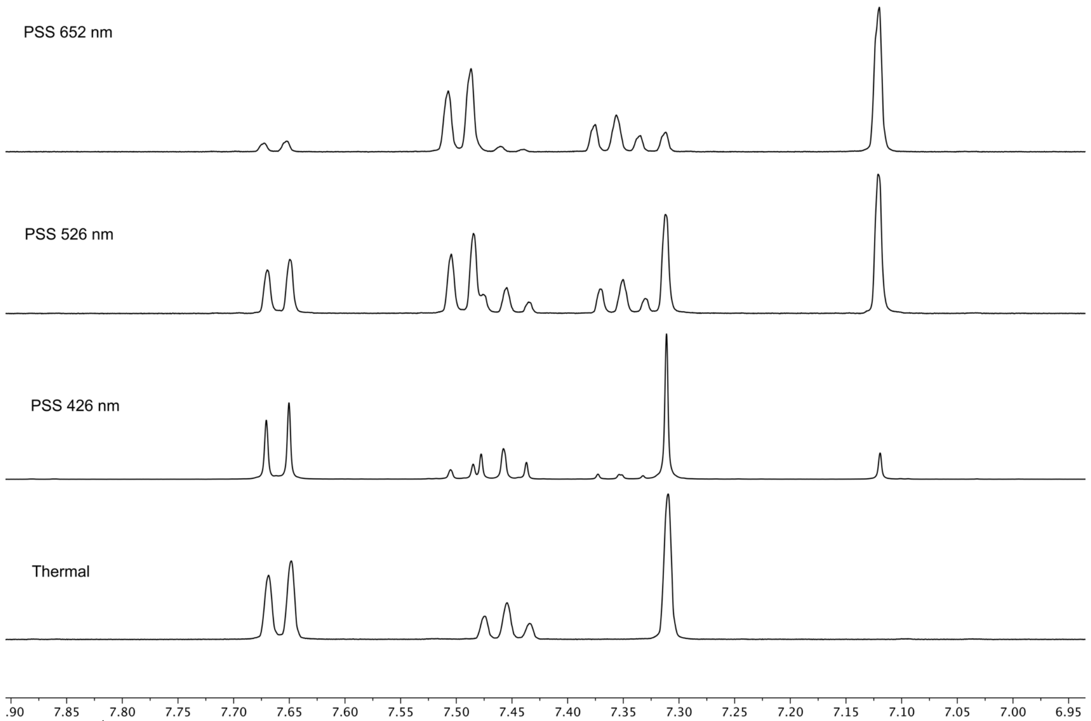

Figure 5. ^{1}HNMR Aromatic region of **2** (>5 mM) in [D$_6$]DMSO at 293K after irradiation at rt with either a Sahlmann cooled 3 x Roithner VL-400-Emitter (652 nm, FWHM = 26.4 nm, power output = 1200 mW), a Sahlmann cooled 3 x LXML PM01 0100 (526 nm, FWHM = 35.1 nm, power output = 810 mW), or a Sahlmann cooled 3 x Roithner SMB-1N 430h (426 nm, FWHM = 16 nm, power output = 600 mW).

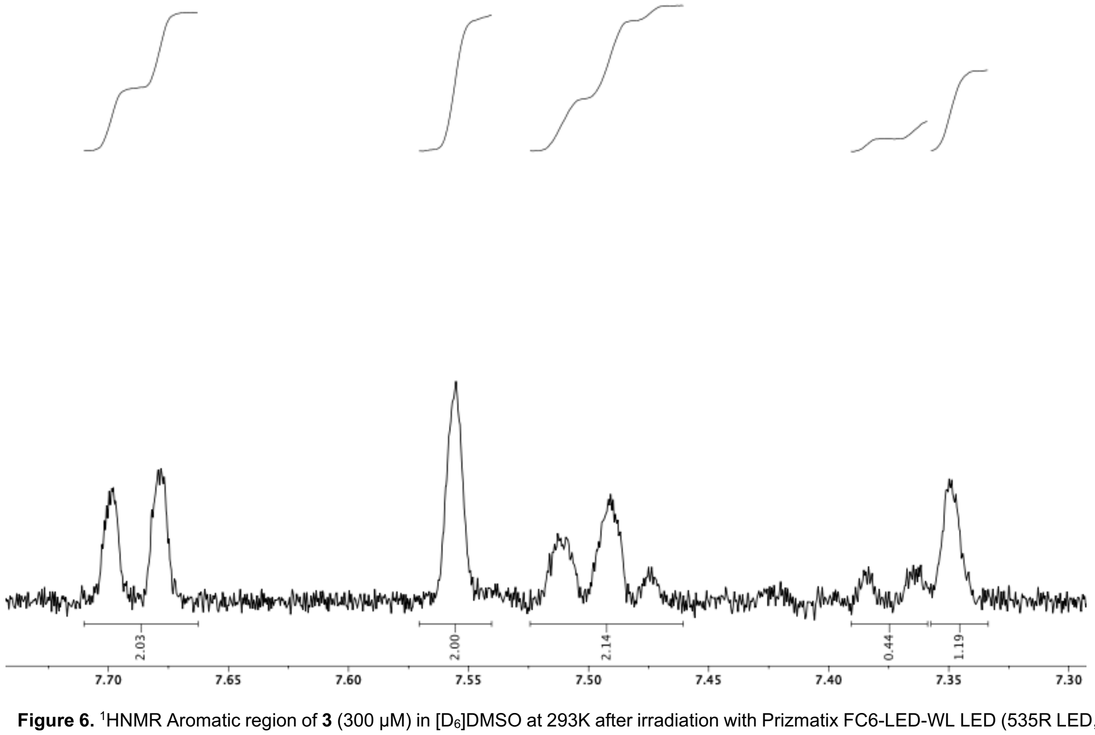

Figure 6. ^{1}HNMR Aromatic region of **3** (300 µM) in [D$_6$]DMSO at 293K after irradiation with Prizmatix FC6-LED-WL LED (535R LED, FWHM = 90 nm) until reaching the photostationary state. Data was processed with MestreNova 12.

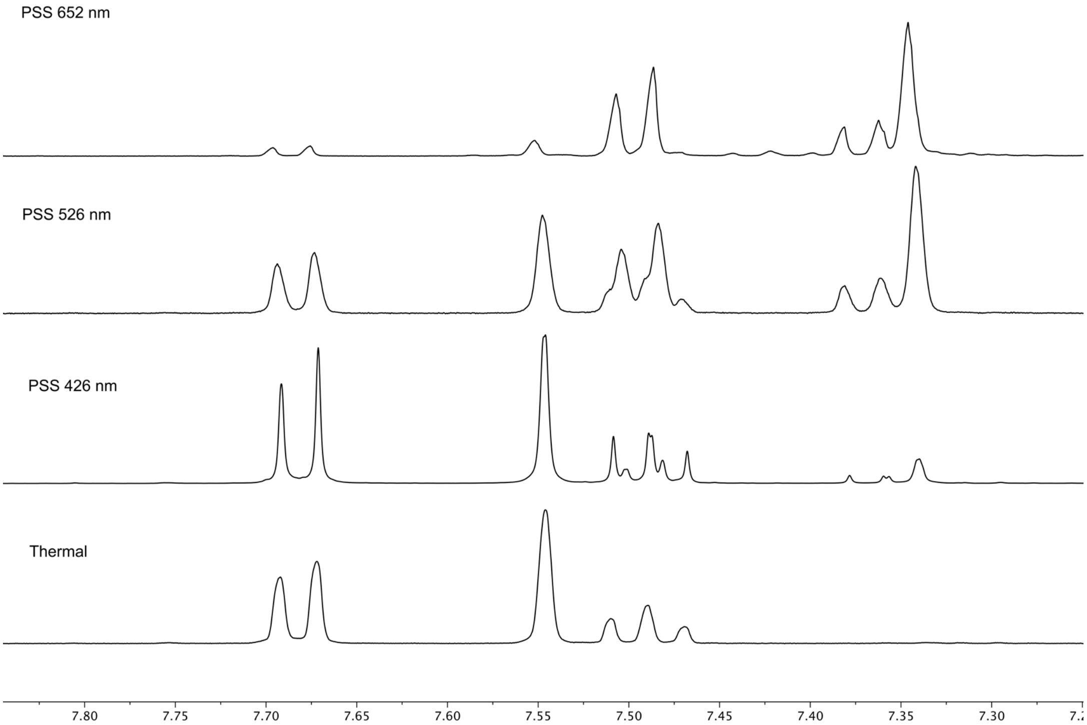

Figure 7. ^{1}HNMR Aromatic region of **3** (>5 mM) in [D$_6$]DMSO at 293K after irradiation at rt with either a Sahlmann cooled 3 x Roithner VL-400-Emitter (652 nm, FWHM = 26.4 nm, power output = 1200 mW), a Sahlmann cooled 3 x LXML PM01 0100 (526 nm, FWHM = 35.1 nm, power output = 810 mW), or a Sahlmann cooled 3 x Roithner SMB-1N 430h (426 nm, FWHM = 16 nm, power output = 600 mW).

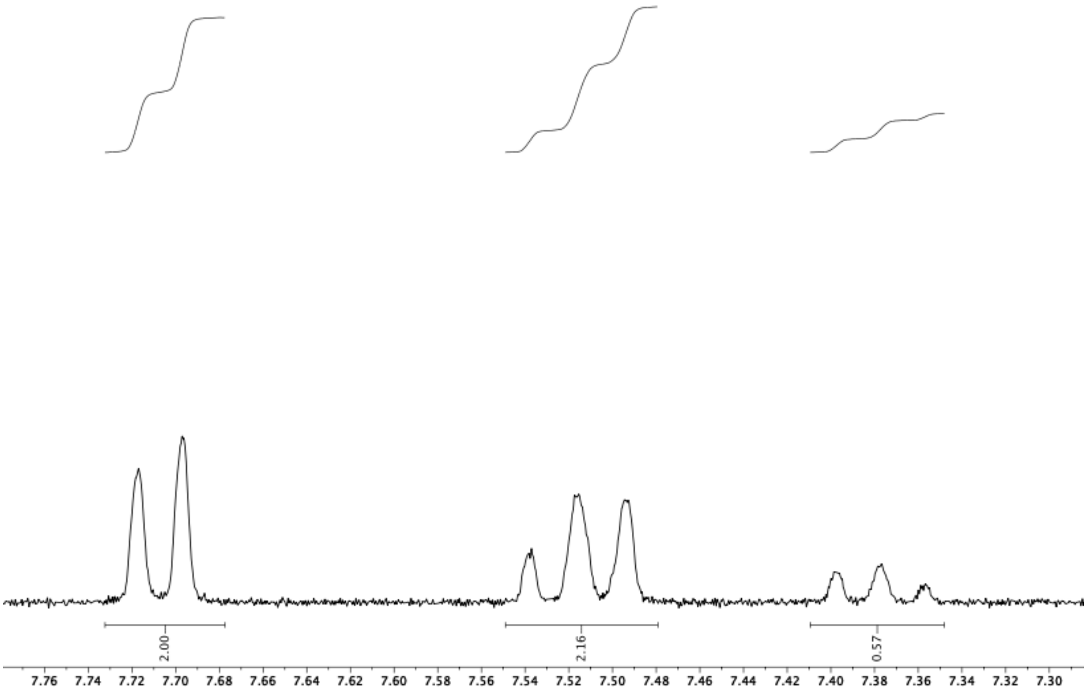

Figure 8. ^{1}HNMR Aromatic region of **4** (300 μM) in [D$_6$]DMSO at 293K after irradiation with Prizmatix FC6-LED-WL LED (535R LED, FWHM = 90 nm) until reaching the photostationary state. Data was processed with MestreNova 12.

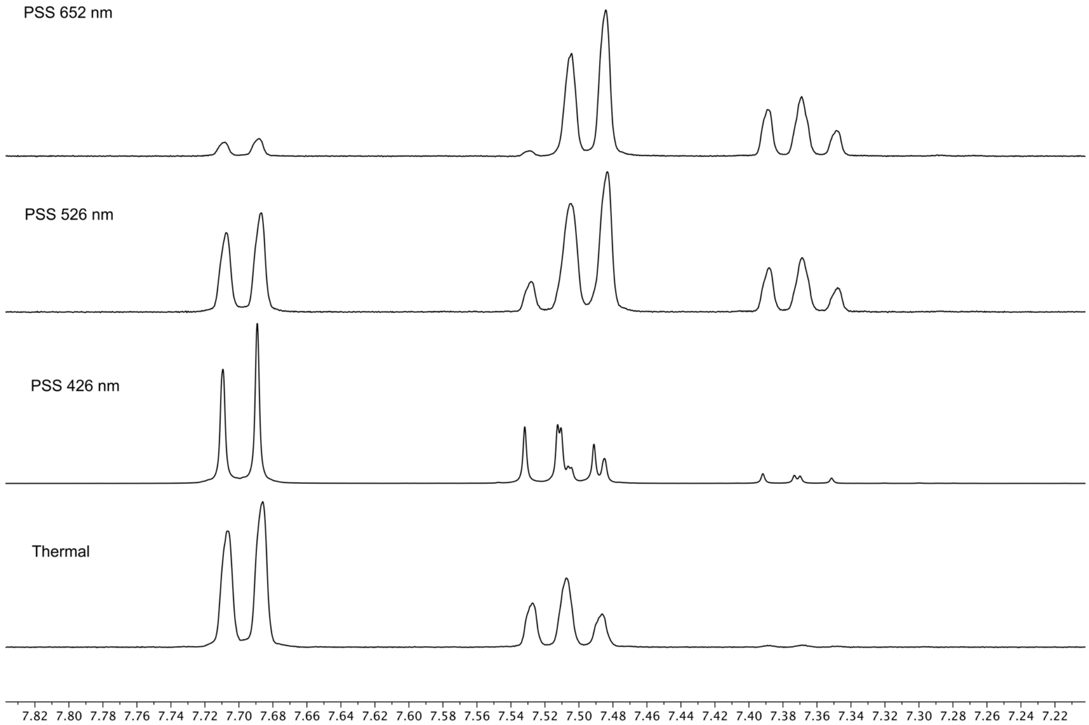

Figure 9. ^{1}HNMR Aromatic region of **4** (>5 mM) in [D_6]DMSO at 293K after irradiation at rt with either a Sahlmann cooled 3 x Roithner VL-400-Emitter (652 nm, FWHM = 26.4 nm, power output = 1200 mW), a Sahlmann cooled 3 x LXML PM01 0100 (526 nm, FWHM = 35.1 nm, power output = 810 mW), or a Sahlmann cooled 3 x Roithner SMB-1N 430h (426 nm, FWHM = 16 nm, power output = 600 mW).

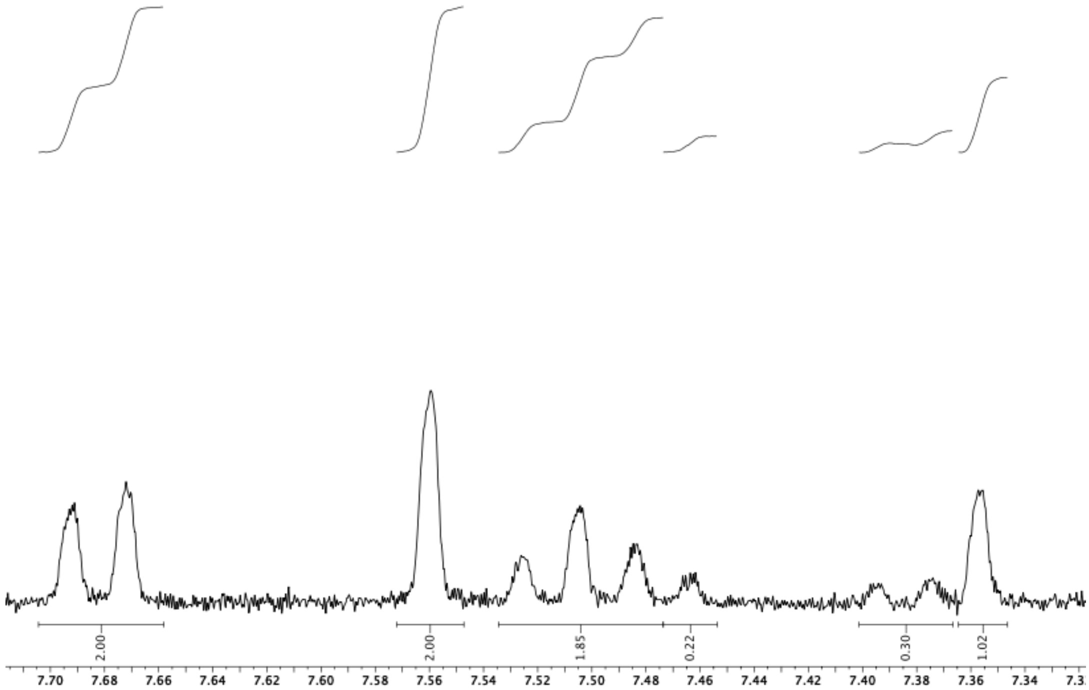

Figure 10. ^{1}HNMR Aromatic region of **5** (240 µM) in [D_6]DMSO at 293K after irradiation with Prizmatix FC6-LED-WL LED (535R LED, FWHM = 90 nm) until reaching the photostationary state. Data was processed with MestreNova 12.

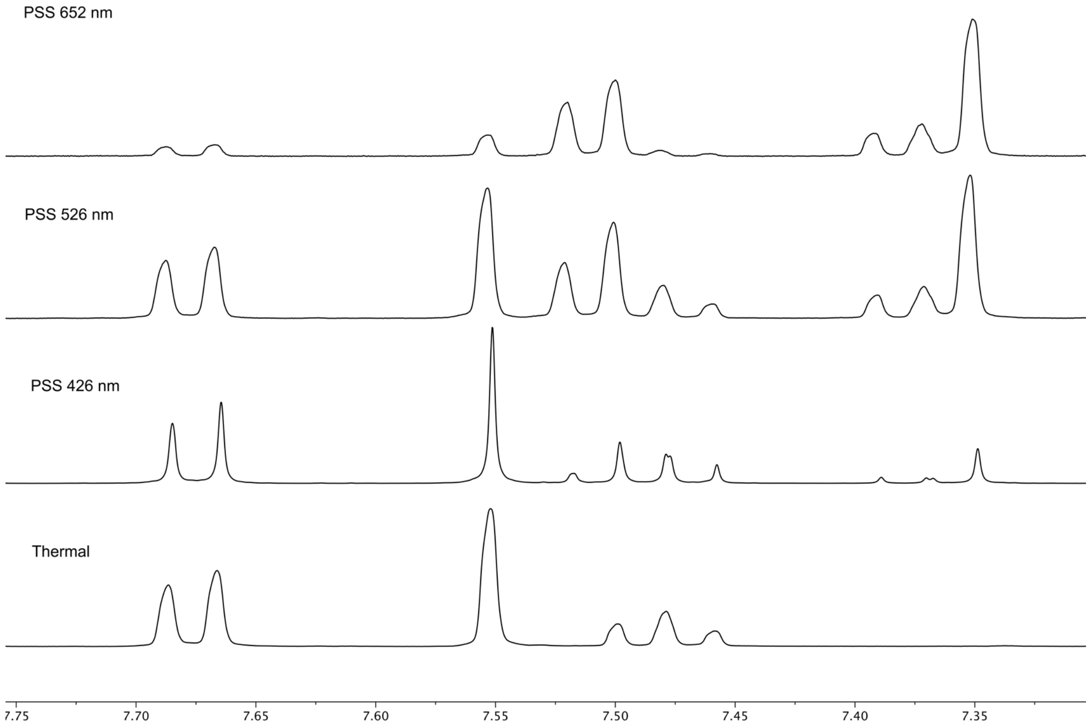

Figure 11. ^{1}HNMR Aromatic region of **5** (>5 mM) in [D$_6$]DMSO at 293K after irradiation at rt with either a Sahlmann cooled 3 x Roithner VL-400-Emitter (652 nm, FWHM = 26.4 nm, power output = 1200 mW), a Sahlmann cooled 3 x LXML PM01 0100 (526 nm, FWHM = 35.1 nm, power output = 810 mW), or a Sahlmann cooled 3 x Roithner SMB-1N 430h (426 nm, FWHM = 16 nm, power output = 600 mW).

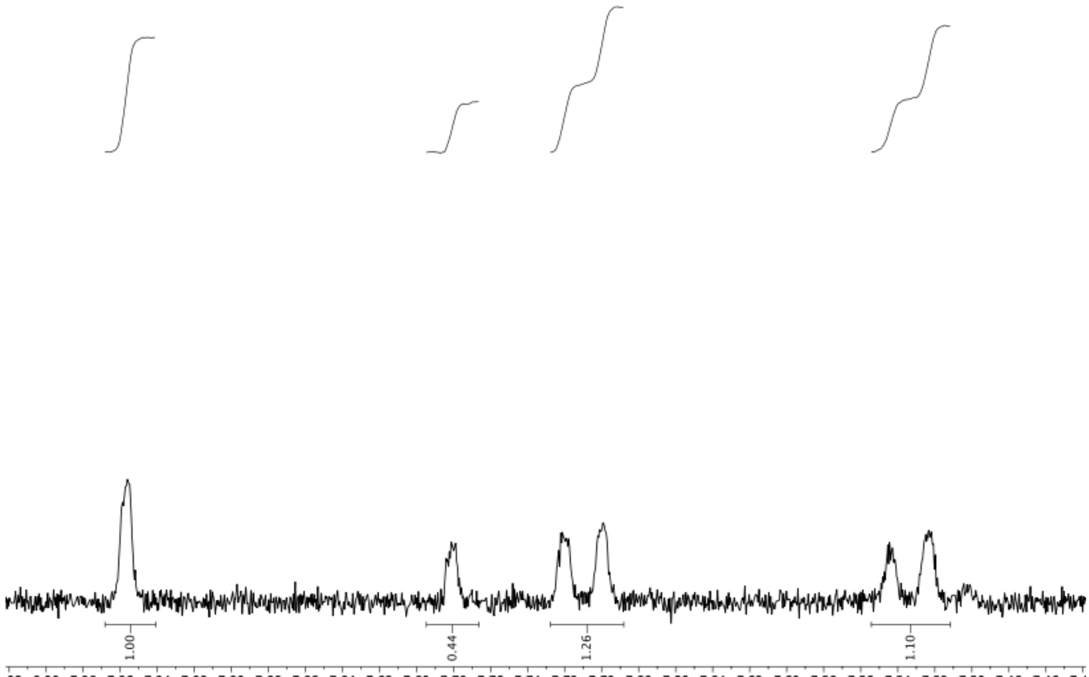

Figure 12. ^{1}HNMR Aromatic region of **6** (200 µM) in [D$_6$]DMSO at 293K after irradiation with Prizmatix FC6-LED-WL LED (535R LED, FWHM = 90 nm) until reaching the photostationary state. Data was processed with MestreNova 12.

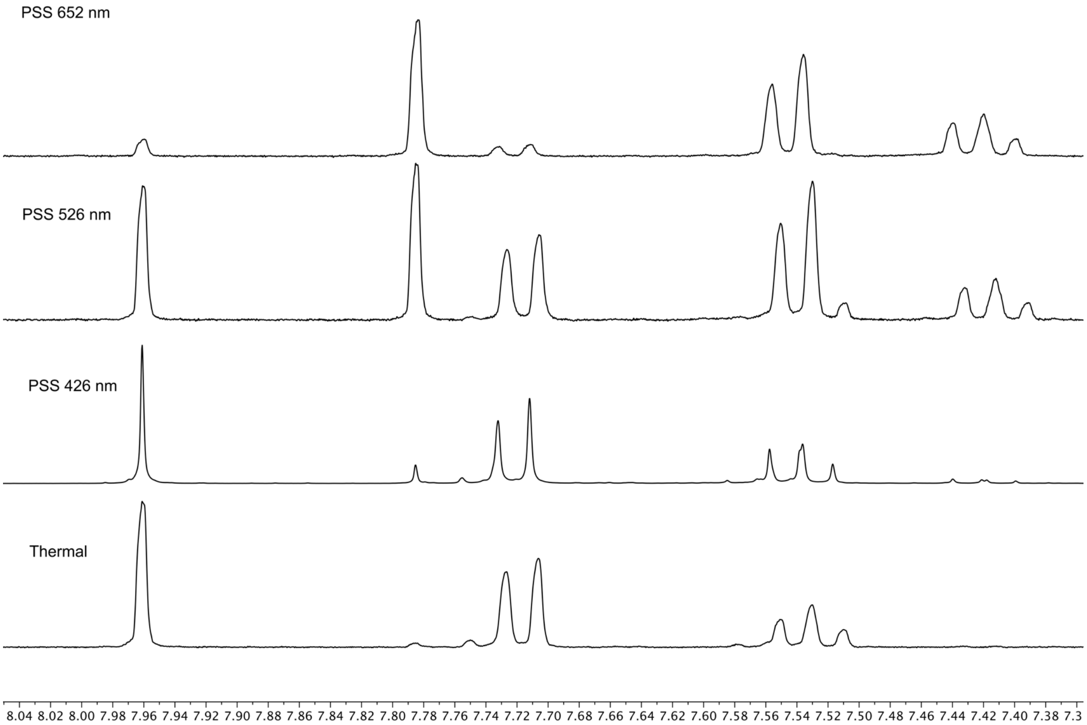

Figure 13. ^{1}HNMR Aromatic region of **6** (>5 mM) in [D_6]DMSO at 293K after irradiation at rt with either a Sahlmann cooled 3 x Roithner VL-400-Emitter (652 nm, FWHM = 26.4 nm, power output = 1200 mW), a Sahlmann cooled 3 x LXML PM01 0100 (526 nm, FWHM = 35.1 nm, power output = 810 mW), or a Sahlmann cooled 3 x Roithner SMB-1N 430h (426 nm, FWHM = 16 nm, power output = 600 mW).

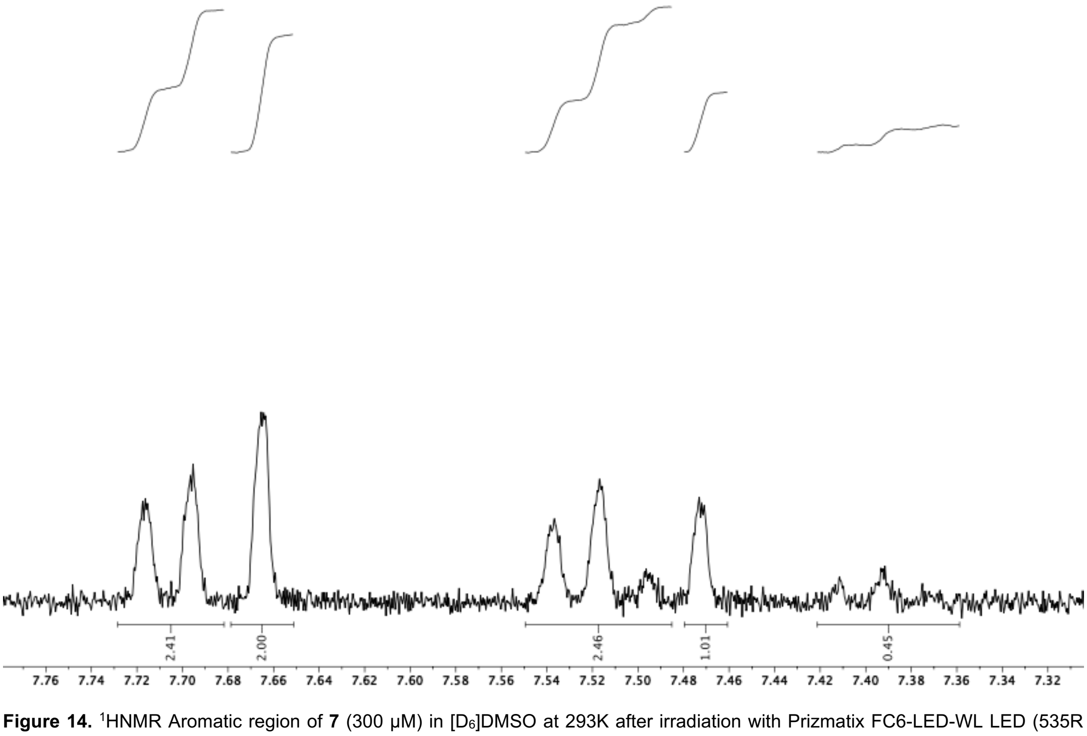

Figure 14. ^{1}HNMR Aromatic region of **7** (300 μM) in [D_6]DMSO at 293K after irradiation with Prizmatix FC6-LED-WL LED (535R LED, FWHM = 90 nm) until reaching the photostationary state. Data was processed with MestreNova 12.

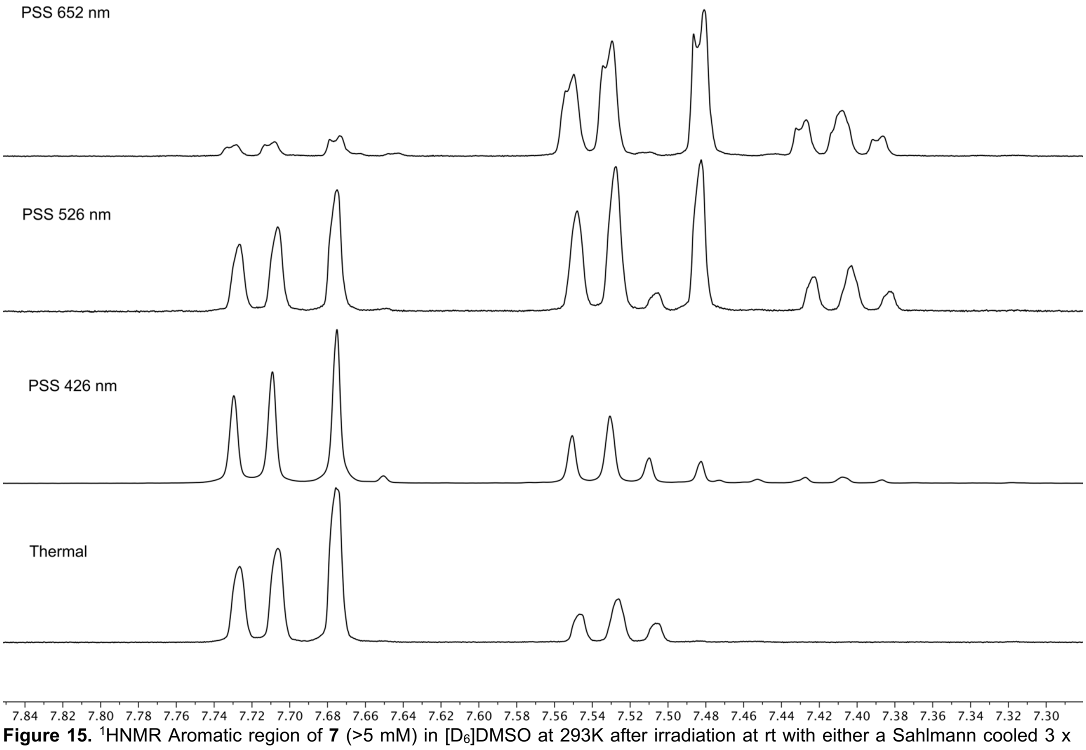

Figure 15. ^{1}HNMR Aromatic region of **7** (>5 mM) in [D$_6$]DMSO at 293K after irradiation at rt with either a Sahlmann cooled 3 x Roithner VL-400-Emitter (652 nm, FWHM = 26.4 nm, power output = 1200 mW), a Sahlmann cooled 3 x LXML PM01 0100 (526 nm, FWHM = 35.1 nm, power output = 810 mW), or a Sahlmann cooled 3 x Roithner SMB-1N 430h (426 nm, FWHM = 16 nm, power output = 600 mW).

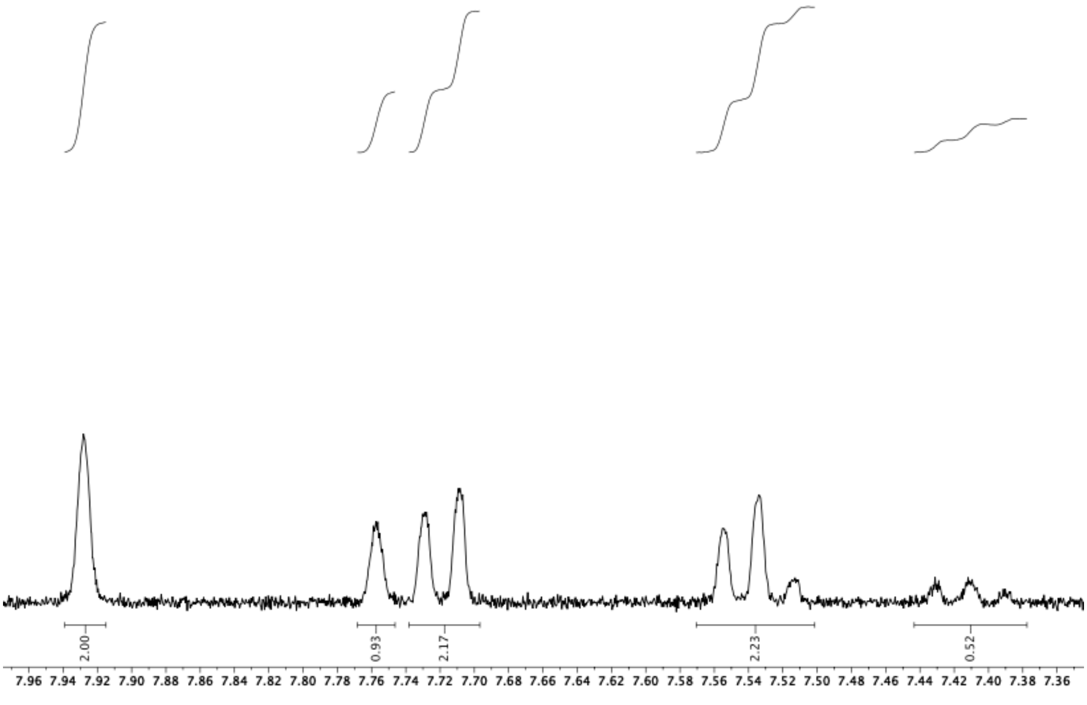

Figure 16. ^{1}HNMR Aromatic region of **8** (300 µM) in [D$_6$]DMSO at 293K after irradiation with Prizmatix FC6-LED-WL LED (535R LED, FWHM = 90 nm) until reaching the photostationary state. Data was processed with MestreNova 12.

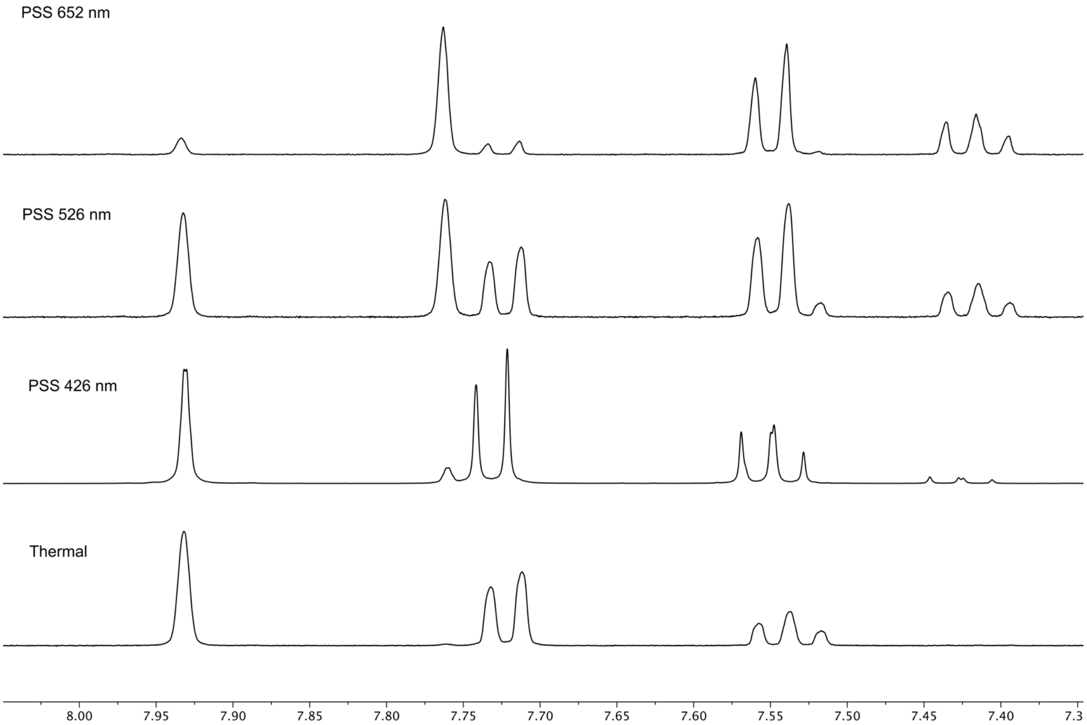

Figure 17. ^{1}HNMR Aromatic region of **8** (>5 mM) in [D_6]DMSO at 293K after irradiation at rt with either a Sahlmann cooled 3 x Roithner VL-400-Emitter (652 nm, FWHM = 26.4 nm, power output = 1200 mW), a Sahlmann cooled 3 x LXML PM01 0100 (526 nm, FWHM = 35.1 nm, power output = 810 mW), or a Sahlmann cooled 3 x Roithner SMB-1N 430h (426 nm, FWHM = 16 nm, power output = 600 mW).

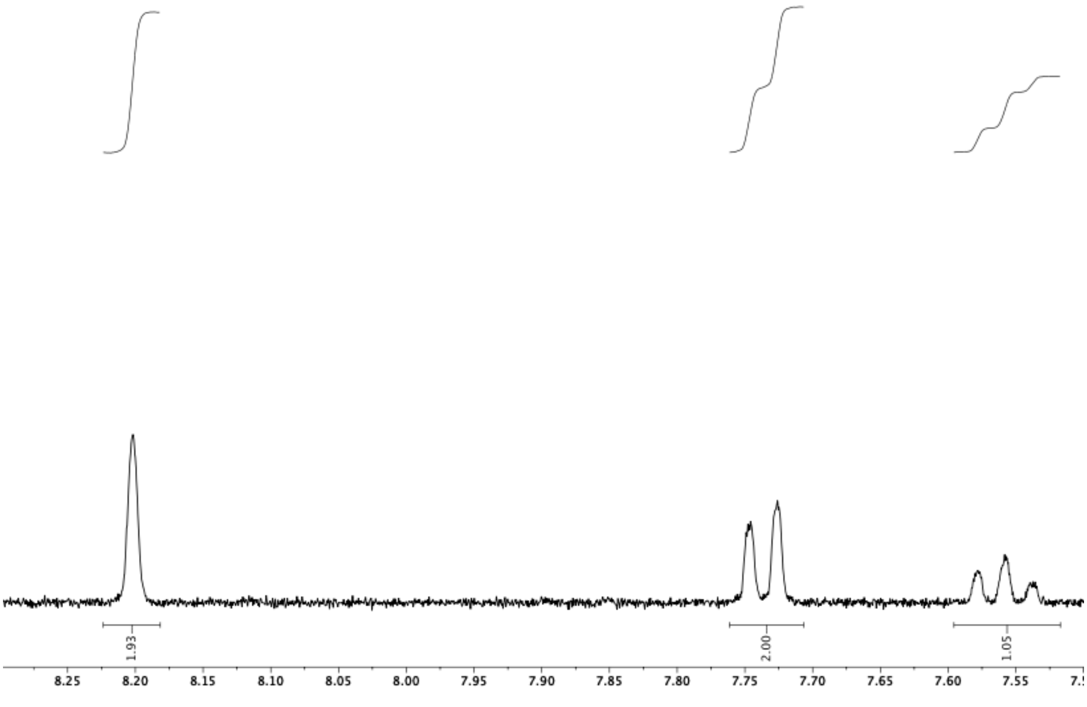

Figure 18. ^{1}HNMR Aromatic region of **9** (261 µM) in [D_6]DMSO at 293K after irradiation with Prizmatix FC6-LED-WL LED (535R LED, FWHM = 90 nm) until reaching the photostationary state. Data was processed with MestreNova 12.

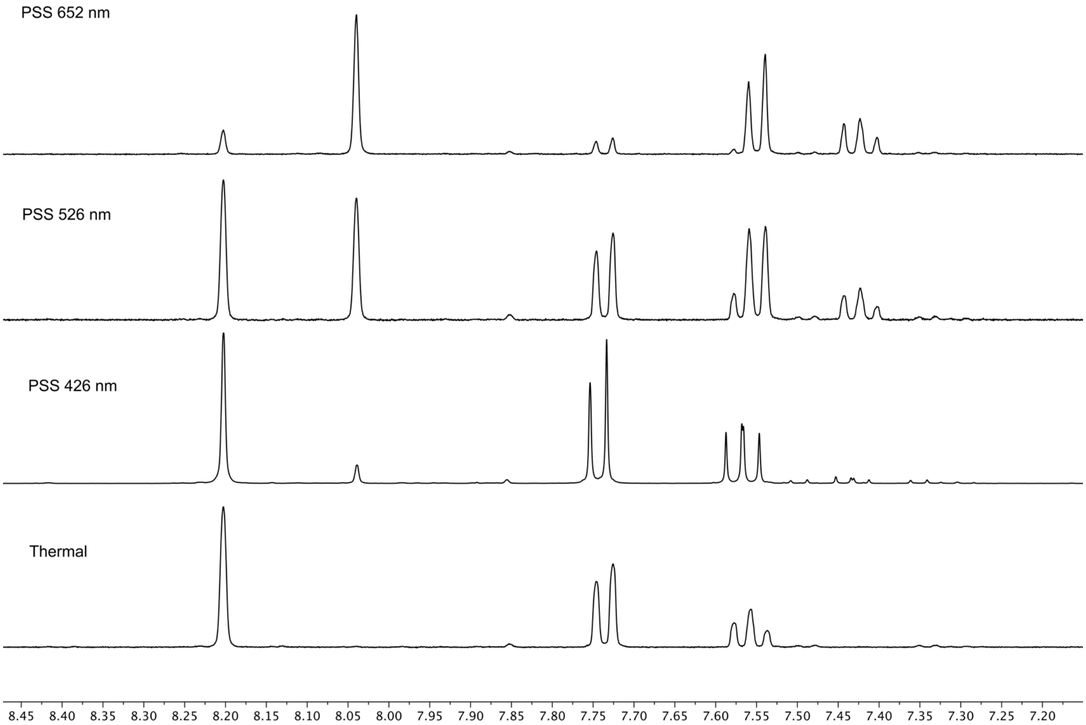

Figure 19. ^{1}HNMR Aromatic region of **9** (>5 mM) in [D$_6$]DMSO at 293K after irradiation at rt with either a Sahlmann cooled 3 x Roithner VL-400-Emitter (652 nm, FWHM = 26.4 nm, power output = 1200 mW), a Sahlmann cooled 3 x LXML PM01 0100 (526 nm, FWHM = 35.1 nm, power output = 810 mW), or a Sahlmann cooled 3 x Roithner SMB-1N 430h (426 nm, FWHM = 16 nm, power output = 600 mW).

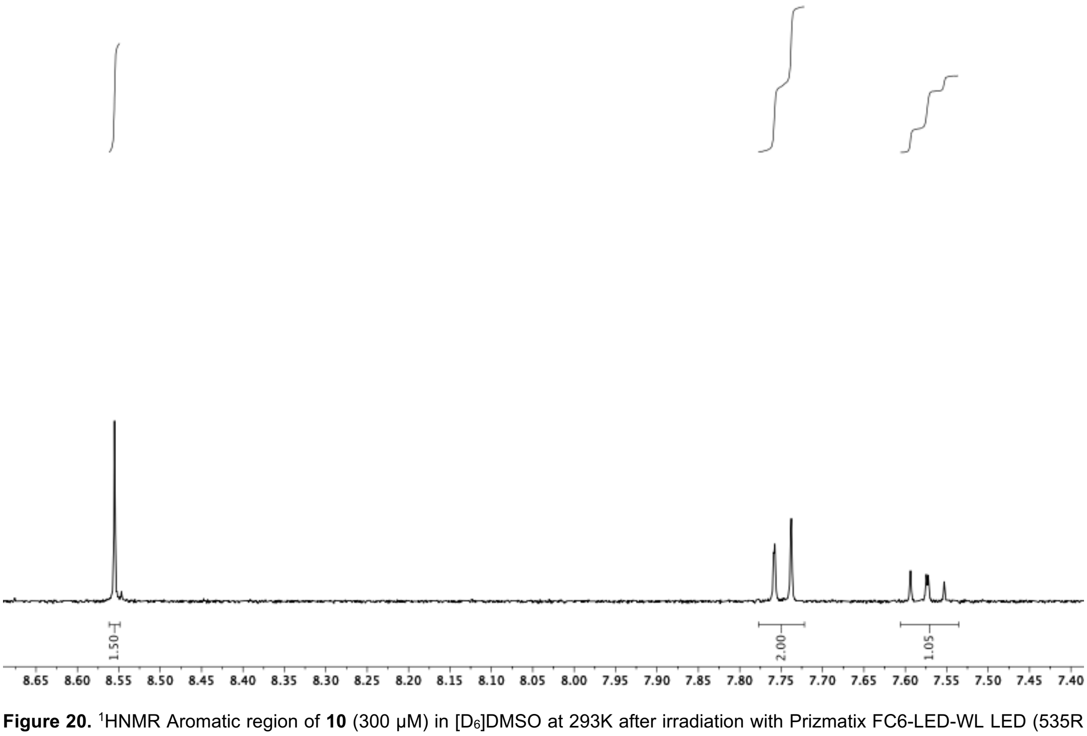

Figure 20. ^{1}HNMR Aromatic region of **10** (300 µM) in [D$_6$]DMSO at 293K after irradiation with Prizmatix FC6-LED-WL LED (535R LED, FWHM = 90 nm) until reaching the photostationary state. Data was processed with MestreNova 12.

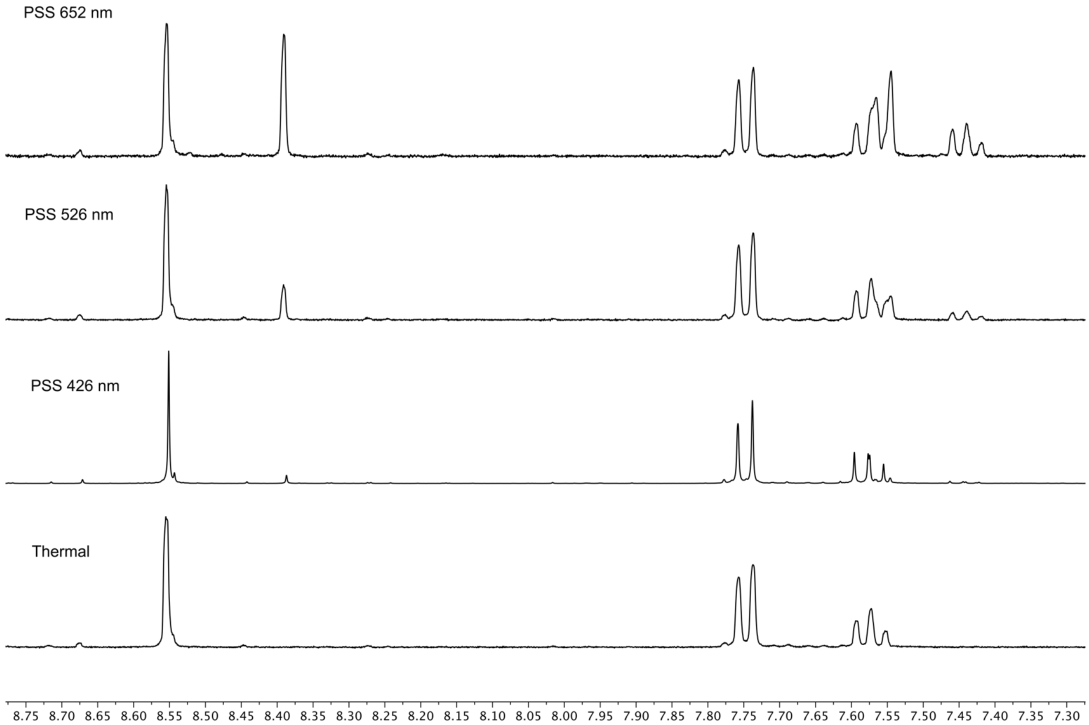

Figure 21. ^{1}HNMR Aromatic region of **10** (>5 mM) in [D6]DMSO at 293K after irradiation at rt with either a Sahlmann cooled 3 x Roithner VL-400-Emitter (652 nm, FWHM = 26.4 nm, power output = 1200 mW), a Sahlmann cooled 3 x LXML PM01 0100 (526 nm, FWHM = 35.1 nm, power output = 810 mW), or a Sahlmann cooled 3 x Roithner SMB-1N 430h (426 nm, FWHM = 16 nm, power output = 600 mW).

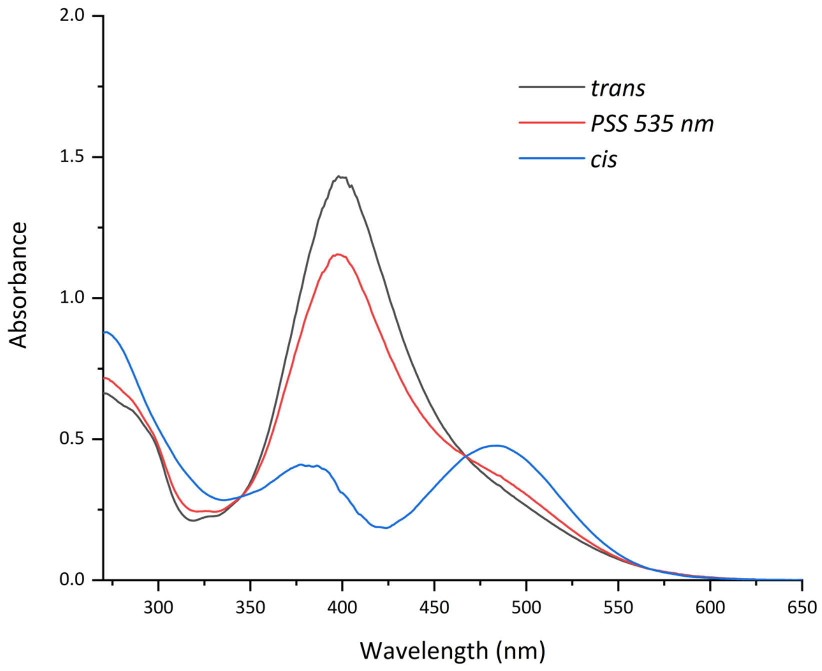

Figure 22. Electronic absorption spectrum of **1** (7.55 · 10^{-5} M in DMSO) before and after irradiation with Prizmatix FC6-LED-WL LED (535R LED, FWHM = 90 nm) until reaching the photostationary state. *Cis* spectra were approximated using the calculated epsilon for the *cis*-species multiplied by the concentration and the pathlength.

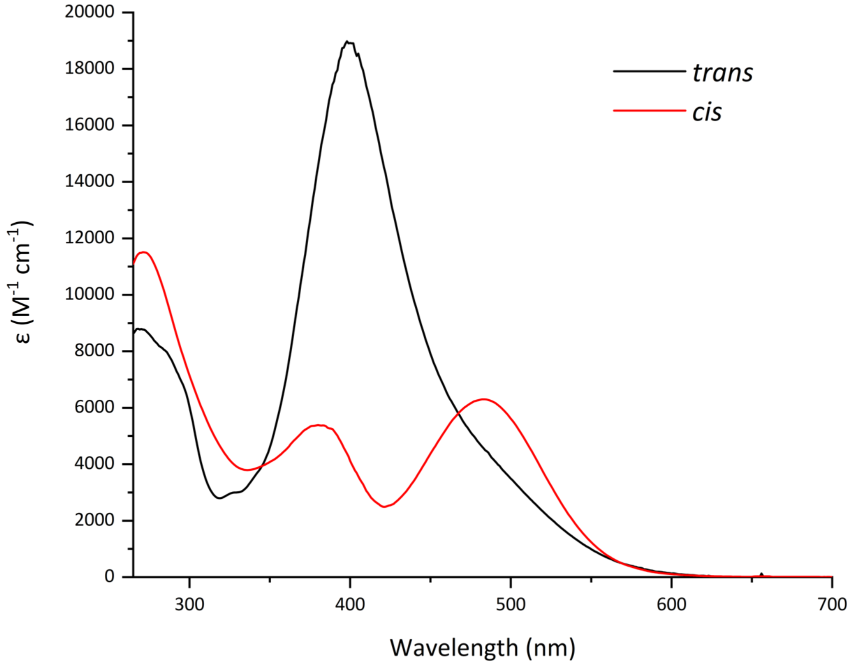

Figure 23. Molar extinction coefficient for *trans*-**1** and *cis*-**1** in DMSO.

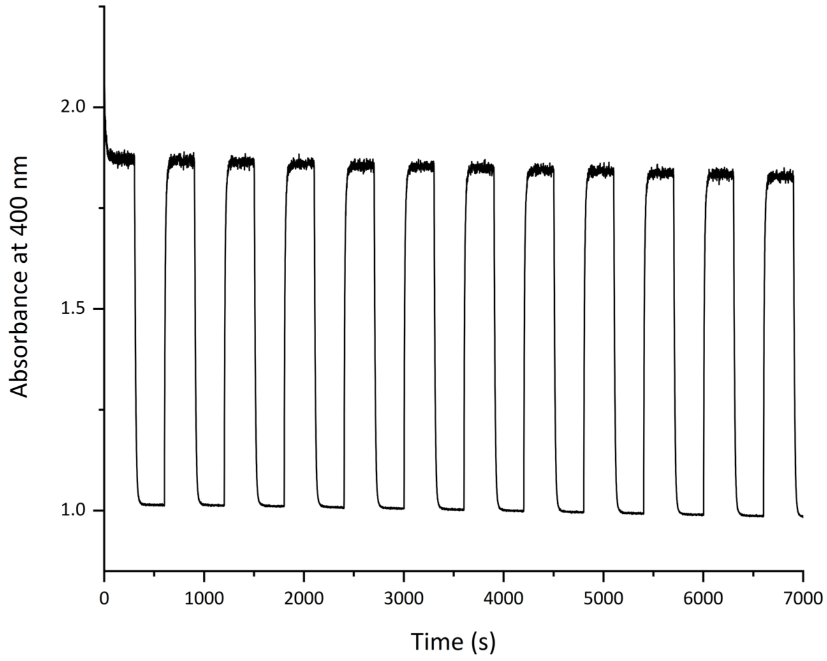

Figure 24. Absorbance at 400 nm of compound **1** ($1.07 \cdot 10^{-4}$ M in DMSO) upon alternating (*trans* = 300 seconds, *cis* = 300 seconds) irradiation with 535 nm and 420 nm light.

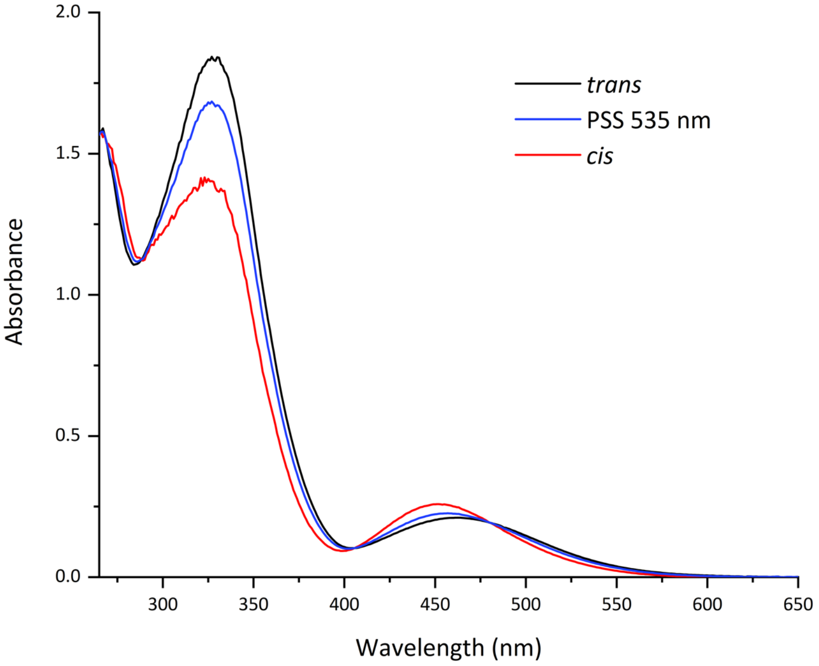

Figure 25. Electronic absorption spectrum of **2** (1.91 · 10^{-4} M in DMSO) before and after irradiation with Prizmatix FC6-LED-WL LED (535R LED, FWHM = 90 nm) until reaching the photostationary state. Cis spectra were approximated using the calculated epsilon for the *cis*-species multiplied by the concentration and the pathlength.

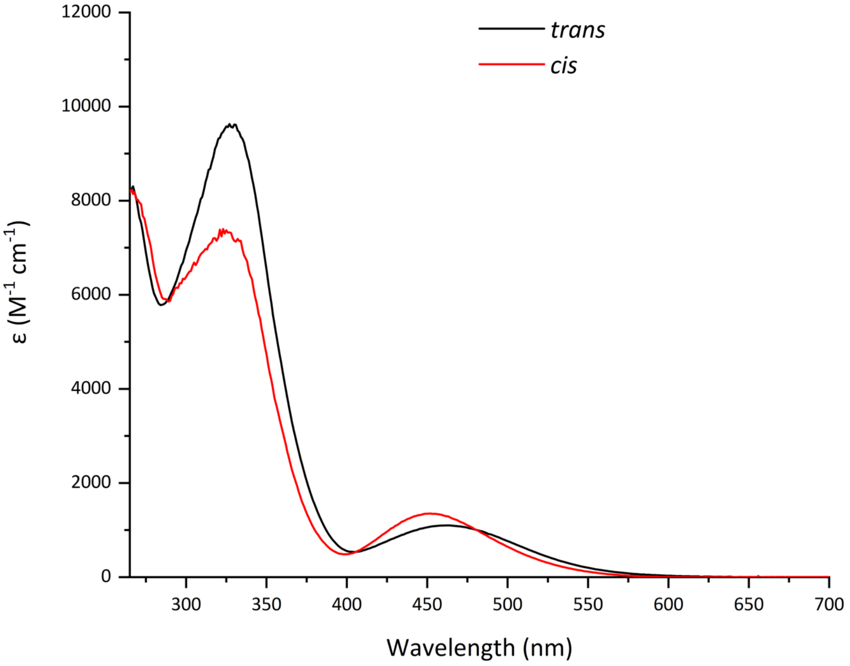

Figure 26. Molar extinction coefficient for *trans*-**2** and *cis*-**2** in DMSO.

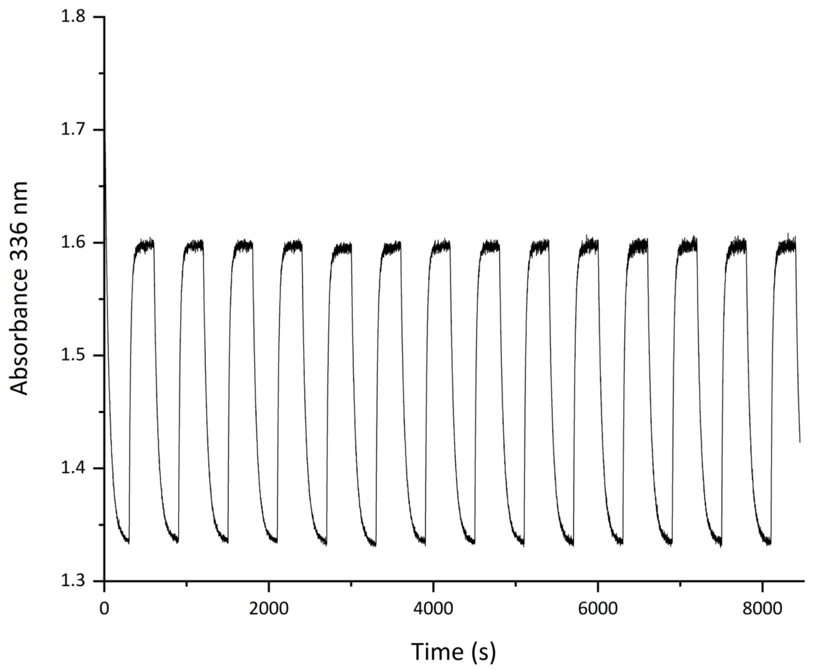

Figure 27. Absorbance at 336 nm of compound **2** (1.91 · 10^{-4} M in DMSO) upon alternating (*trans* = 300 seconds, *cis* = 300 seconds) irradiation with 535 nm and 420 nm light.

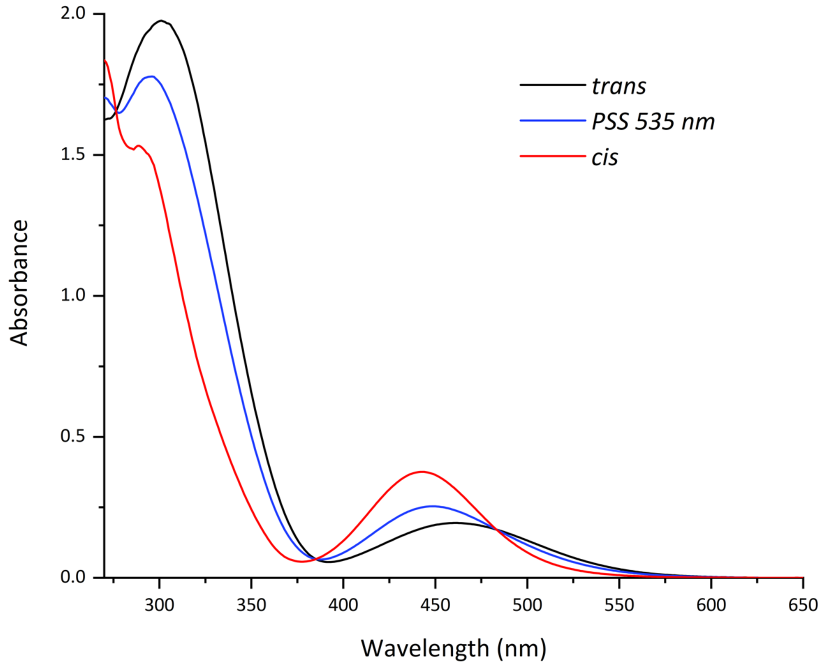

Figure 28. Electronic absorption spectrum of **3** (2.39 · 10^{-4} M in DMSO) before and after irradiation with Prizmatix FC6-LED-WL LED (535R LED, FWHM = 90 nm) until reaching the photostationary state. *Cis* spectra were approximated using the calculated epsilon for the *cis*-species multiplied by the concentration and the pathlength.

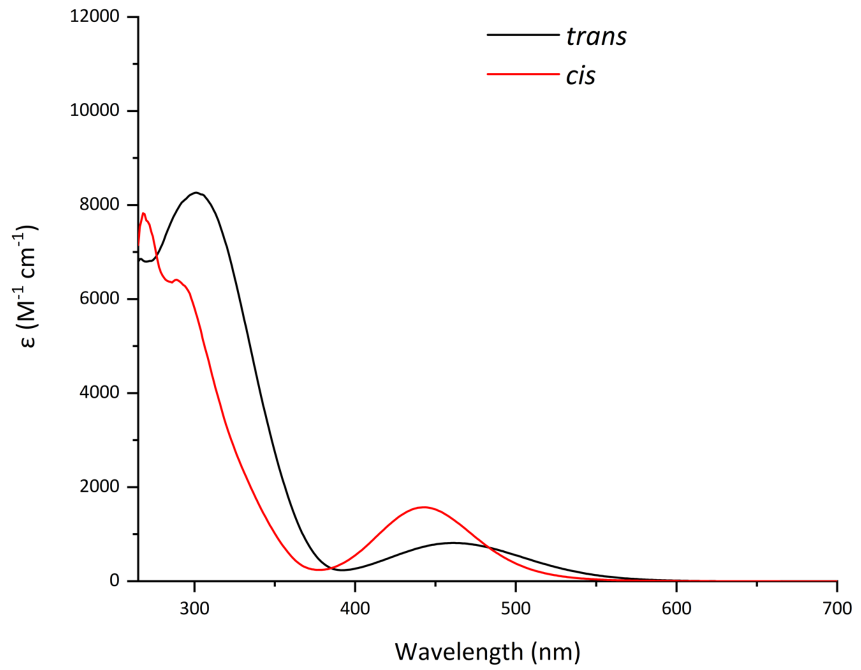

Figure 29. Molar extinction coefficient for *trans*-**3** and *cis*-**3** in DMSO.

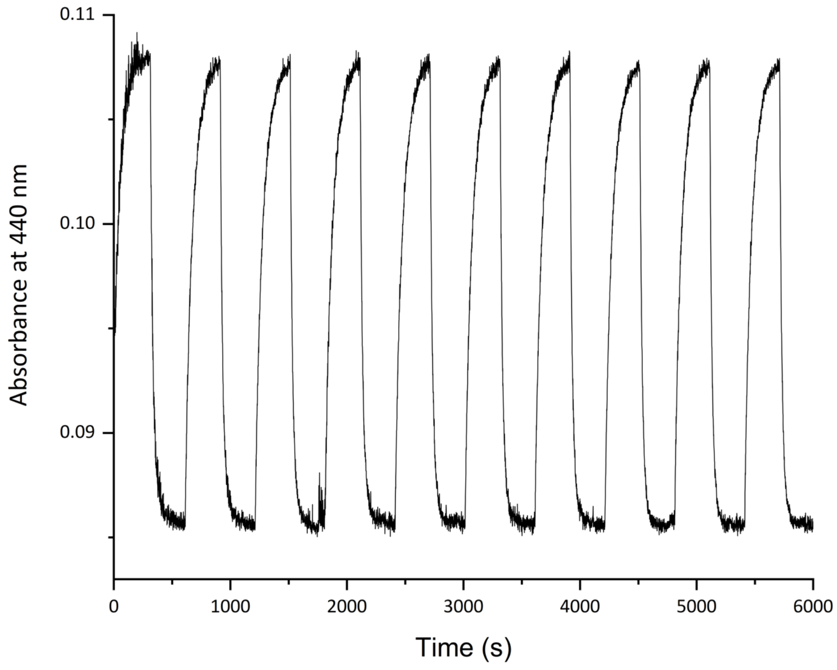

Figure 30. Absorbance at 440 nm of compound **3** ($1.34 \cdot 10^{-4}$ M in DMSO) upon alternating (*trans* = 300 seconds, *cis* = 300 seconds) irradiation with 535 nm and 420 nm light.

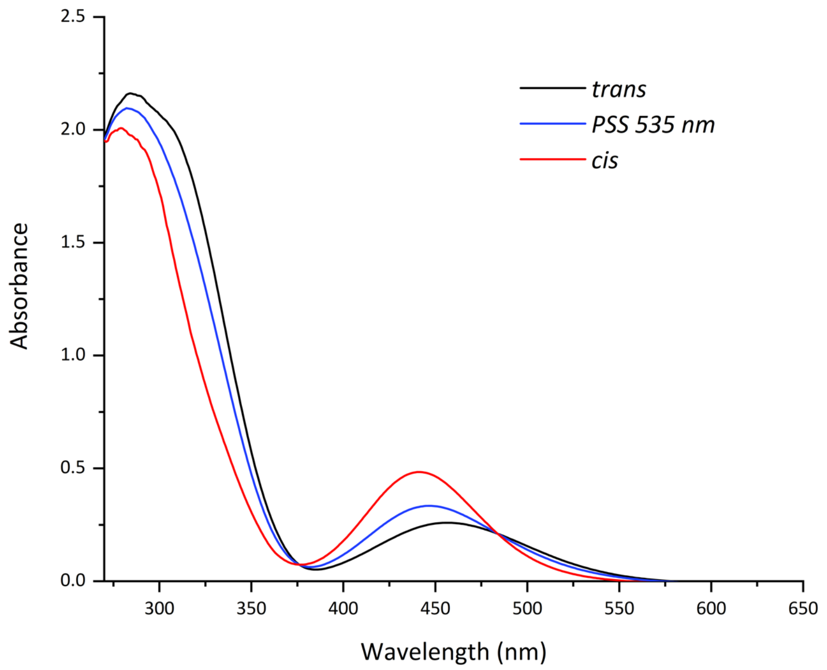

Figure 31. Electronic absorption spectrum of **4** ($3.06 \cdot 10^{-4}$ M in DMSO) before and after irradiation with Prizmatix FC6-LED-WL LED (535R LED, FWHM = 90 nm) until reaching the photostationary state. *Cis* spectra were approximated using the calculated epsilon for the *cis*-species multiplied by the concentration and the pathlength.

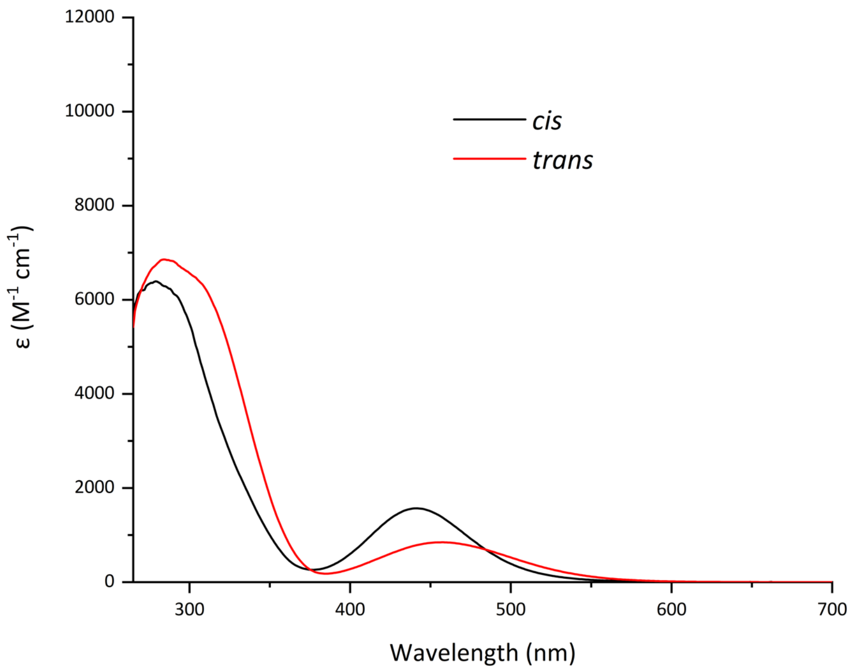

Figure 32. Molar extinction coefficient for *trans*-**4** and *cis*-**4** in DMSO.

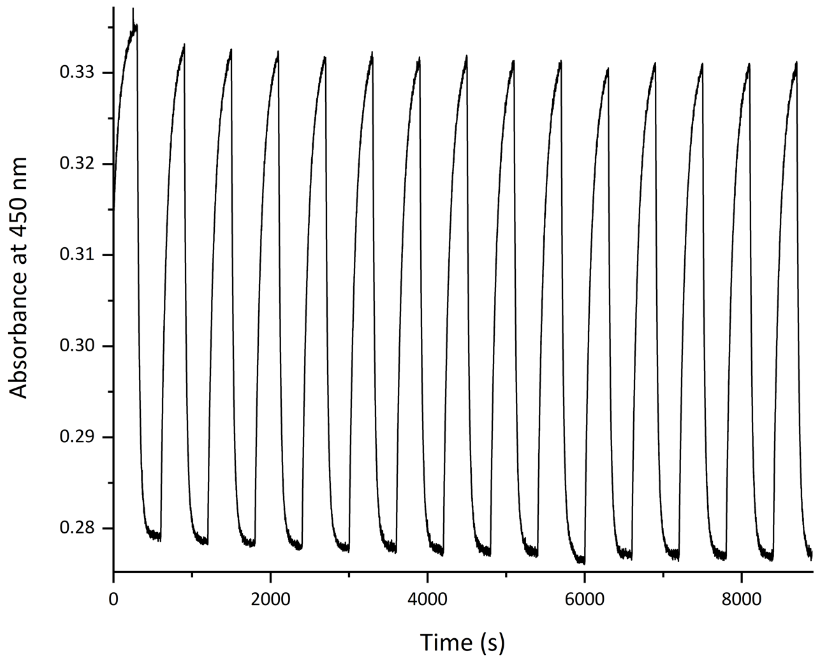

Figure 33. Absorbance at 450 nm of compound **4** (3.76 · 10^{-4} M in DMSO) upon alternating (*trans* = 300 seconds, *cis* = 300 seconds) irradiation with 535 nm and 420 nm light.

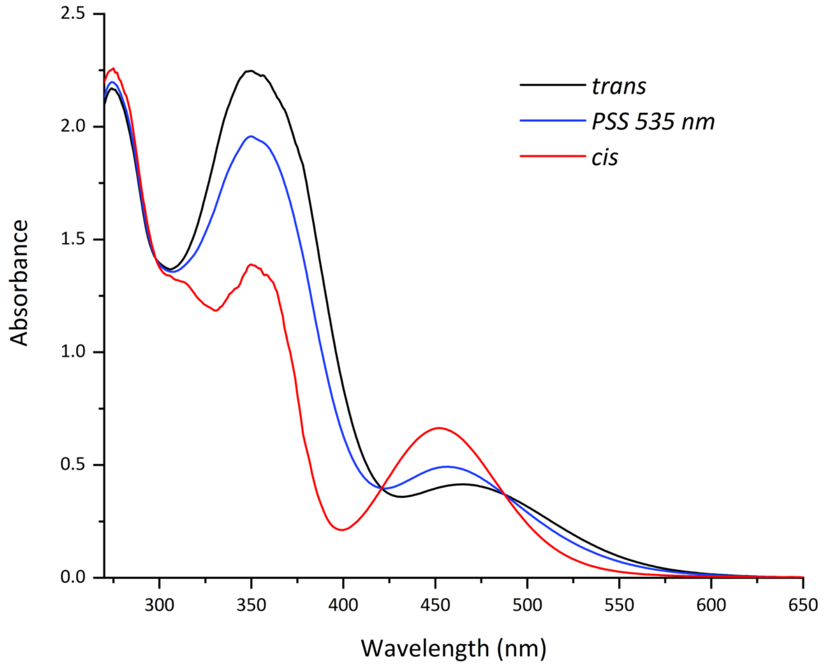

Figure 34. Electronic absorption spectrum of **5** (2.52 · 10^{-4} M in DMSO) before and after irradiation with Prizmatix FC6-LED-WL LED (535R LED, FWHM = 90 nm) until reaching the photostationary state. *Cis* spectra were approximated using the calculated epsilon for the *cis*-species multiplied by the concentration and the pathlength.

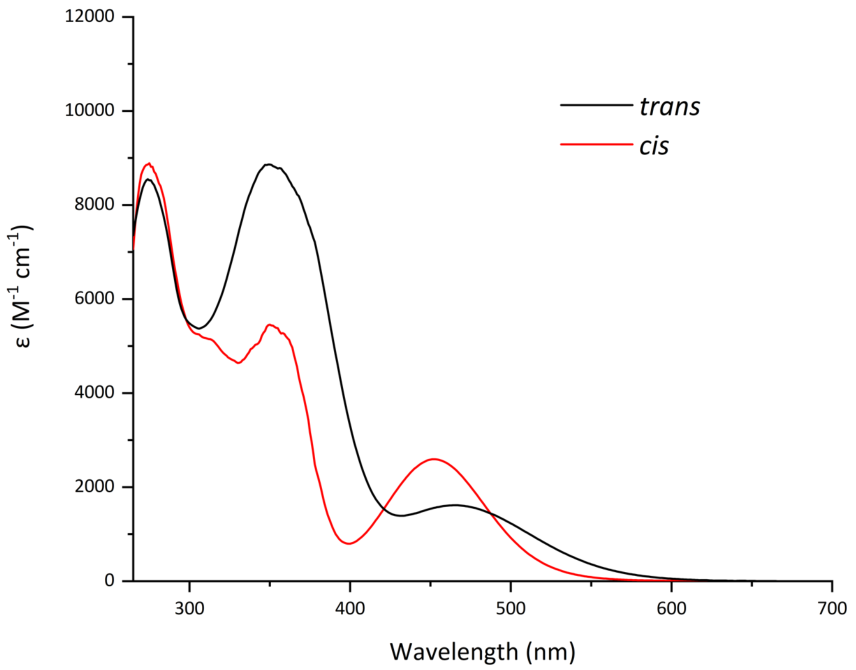

Figure 35. Molar extinction coefficient for *trans*-**5** and *cis*-**5** in DMSO.

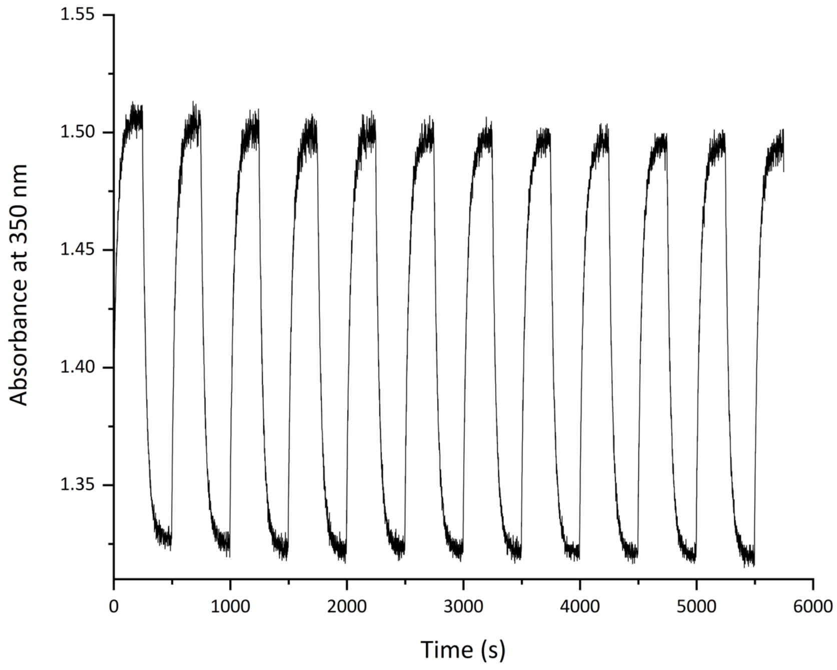

Figure 36. Absorbance at 350 nm of compound **5** ($1.47 \cdot 10^{-4}$ M in DMSO) upon alternating (*trans* = 250 seconds, *cis* = 250 seconds) irradiation with 535 nm or 390 nm light.

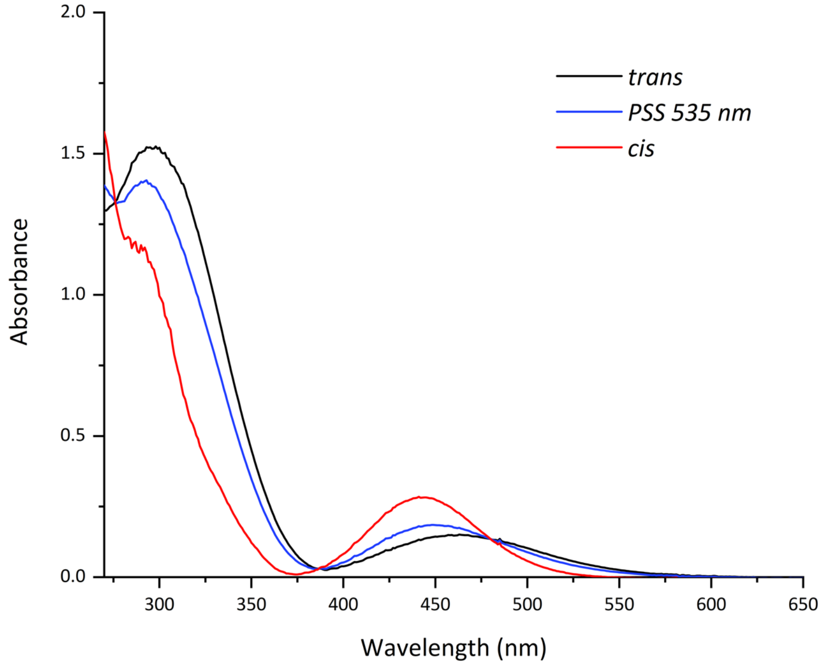

Figure 37. Electronic absorption spectrum of **6** ($1.68 \cdot 10^{-4}$ M in DMSO) before and after irradiation with Prizmatix FC6-LED-WL LED (535R LED, FWHM = 90 nm) until reaching the photostationary state. *Cis* spectra were approximated using the calculated epsilon for the *cis*-species multiplied by the concentration and the pathlength.

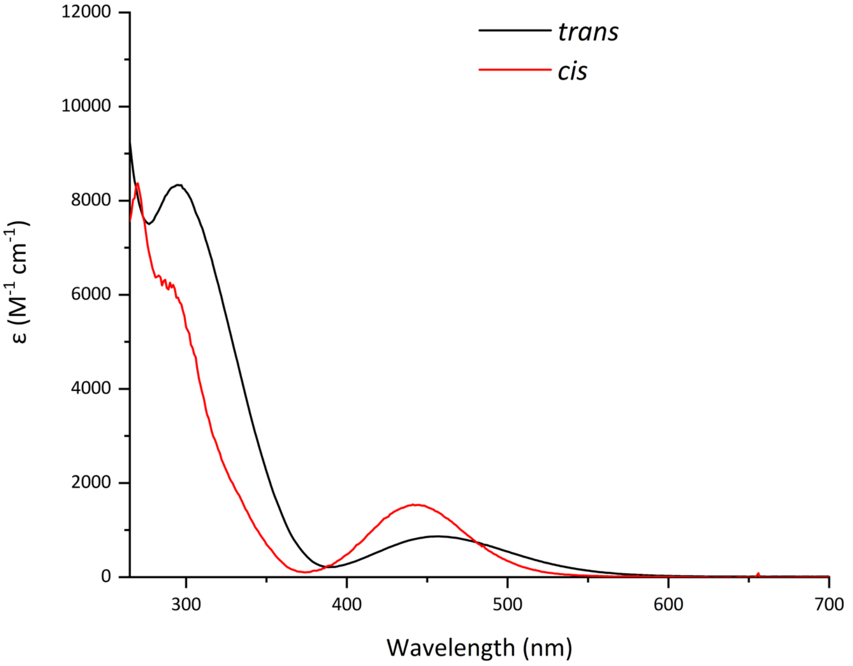

Figure 38. Molar extinction coefficient for *trans*-**6** and *cis*-**6** in DMSO.

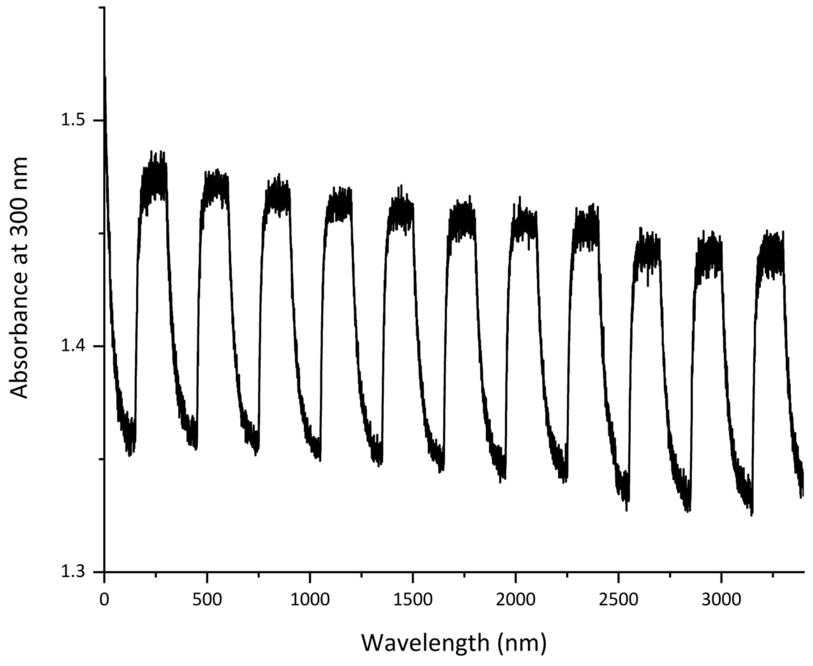

Figure 39. Absorbance at 300 nm of compound **6** (1.85 · 10^{-4} M in DMSO) upon alternating (*trans* = 300 seconds, *cis* = 300 seconds) irradiation with 535 nm and 420 nm light.

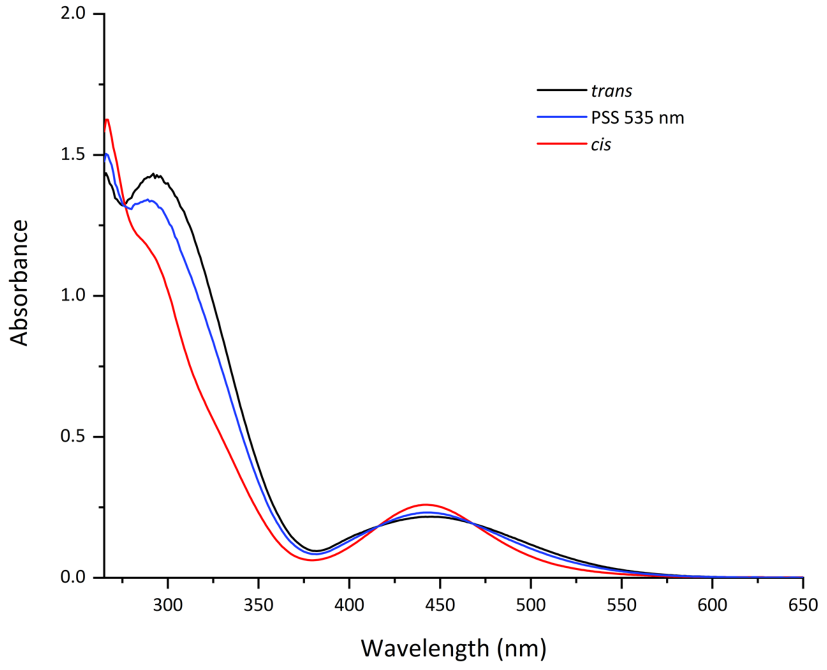

Figure 40. Electronic absorption spectrum of **7** (1.64 · 10^{-4} M in DMSO) before and after irradiation with Prizmatix FC6-LED-WL LED (535R LED, FWHM = 90 nm) until reaching the photostationary state. *Cis* spectra were approximated using the calculated epsilon for the *cis*-species multiplied by the concentration and the pathlength.

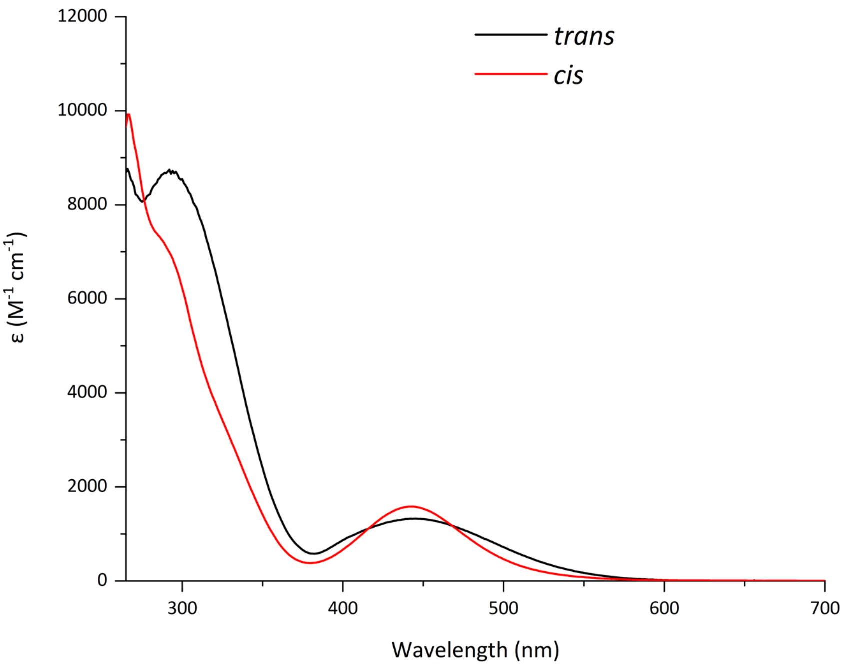

Figure 41. Molar extinction coefficient for *trans*-**7** and *cis*-**7** in DMSO.

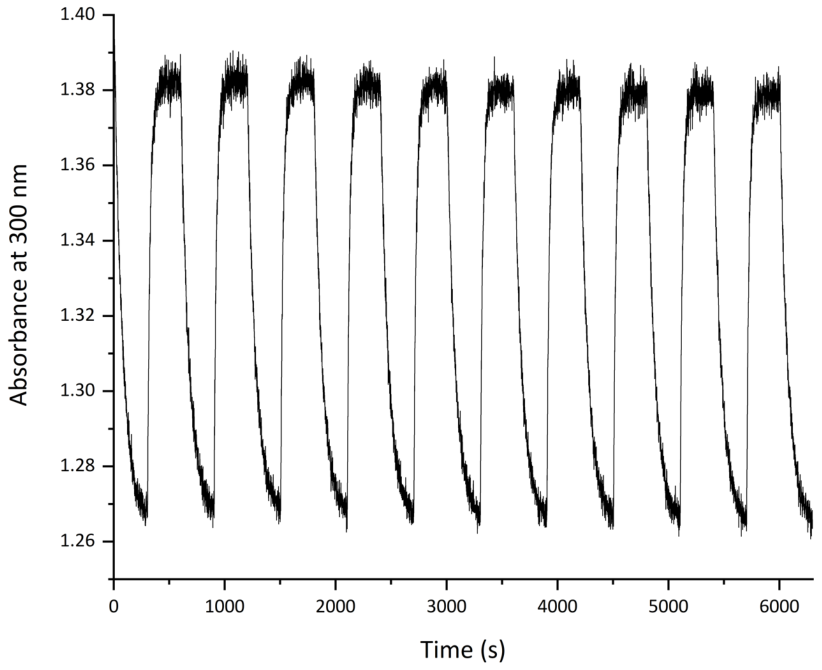

Figure 42. Absorbance at 300 nm of compound **7** (1.64 · 10^{-4} M in DMSO) upon alternating (*trans* = 300 seconds, *cis* = 300 seconds) irradiation with 535 nm and 420 nm light.

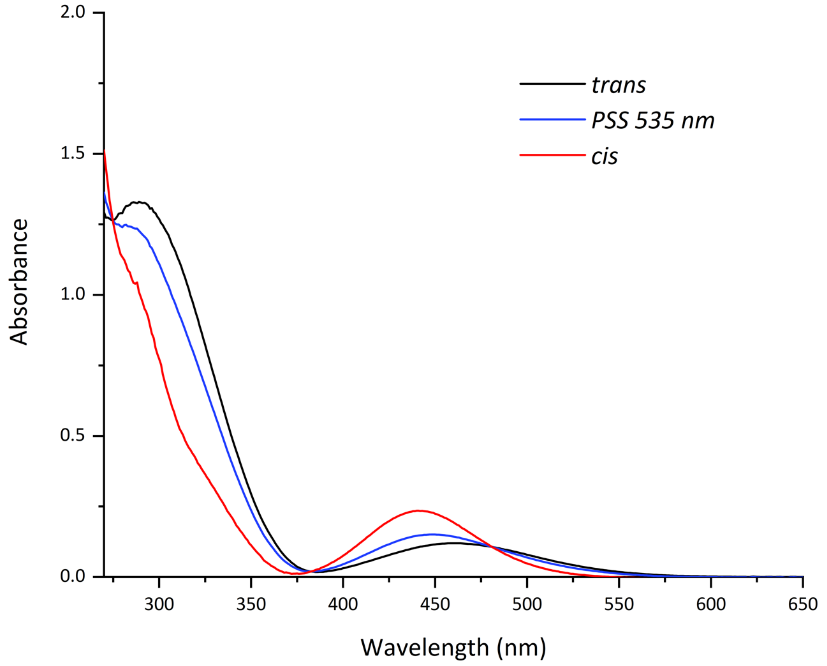

Figure 43. Electronic absorption spectrum of **8** ($1.93 \cdot 10^{-4}$ M in DMSO) before and after irradiation with Prizmatix FC6-LED-WL LED (535R LED, FWHM = 90 nm) until reaching the photostationary state. *Cis* spectra were approximated using the calculated epsilon for the *cis*-species multiplied by the concentration and the pathlength.

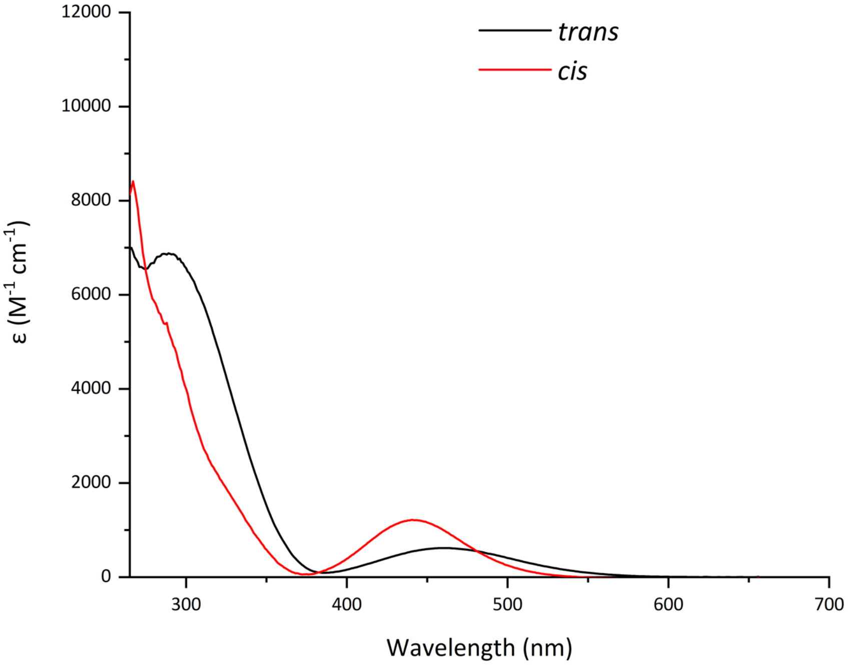

Figure 44. Molar extinction coefficient for *trans*-**8** and *cis*-**8** in DMSO.

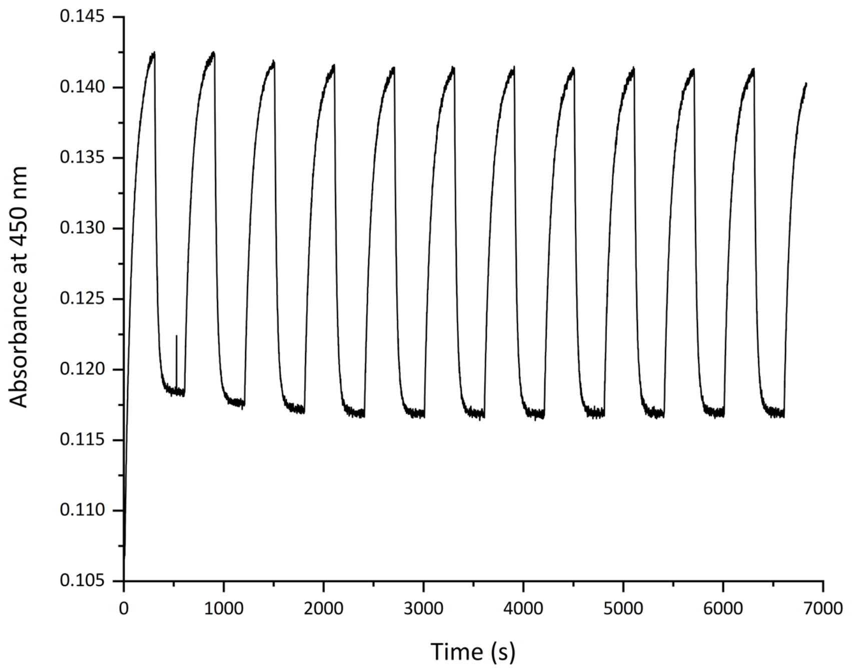

Figure 45. Absorbance at 450 nm of compound **8** (1.78 · 10^{-4} M in DMSO) upon alternating (*trans* = 300 seconds, *cis* = 300 seconds) irradiation with 535 nm and 420 nm light.

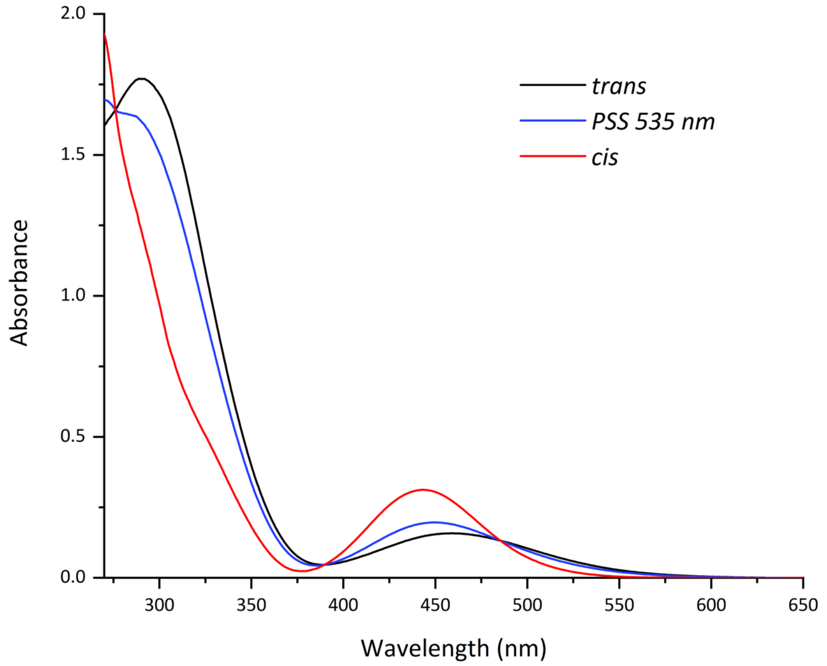

Figure 46. Electronic absorption spectrum of **9** (2.42 · 10^{-4} M in DMSO) before and after irradiation with Prizmatix FC6-LED-WL LED (535R LED, FWHM = 90 nm) until reaching the photostationary state. *Cis* spectra were approximated using the calculated epsilon for the *cis*-species multiplied by the concentration and the pathlength.

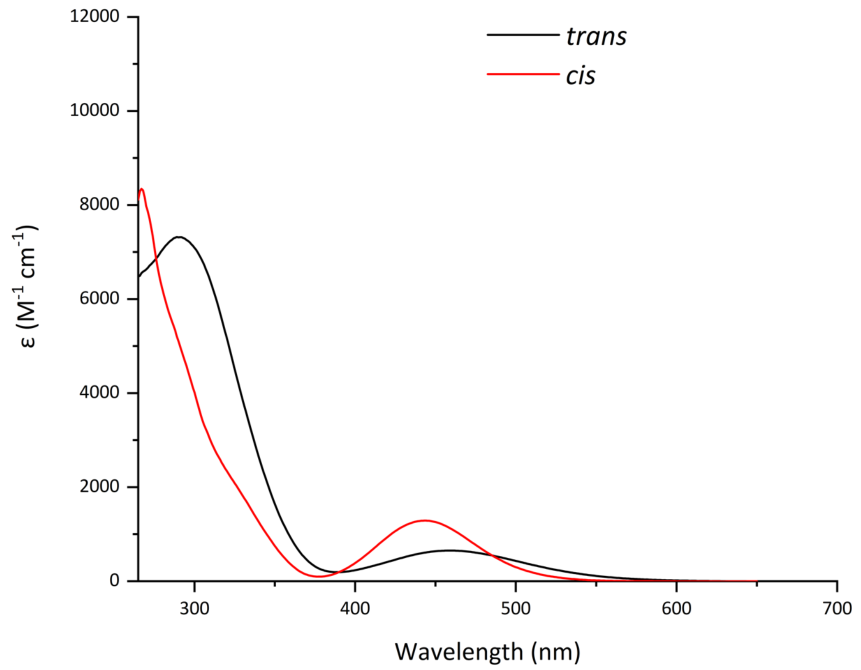

Figure 47. Molar extinction coefficient for *trans*-**9** and *cis*-**9** in DMSO.

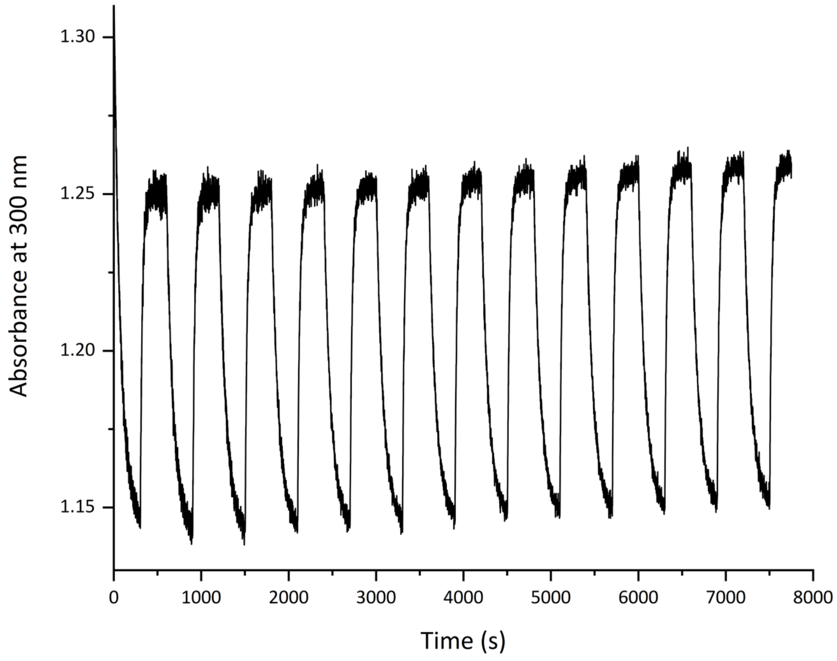

Figure 48. Absorbance at 300 nm of compound **9** ($1.85 \cdot 10^{-4}$ M in DMSO) upon alternating (*trans* = 300 seconds, *cis* = 300 seconds) irradiation with 535 nm and 420 nm light.

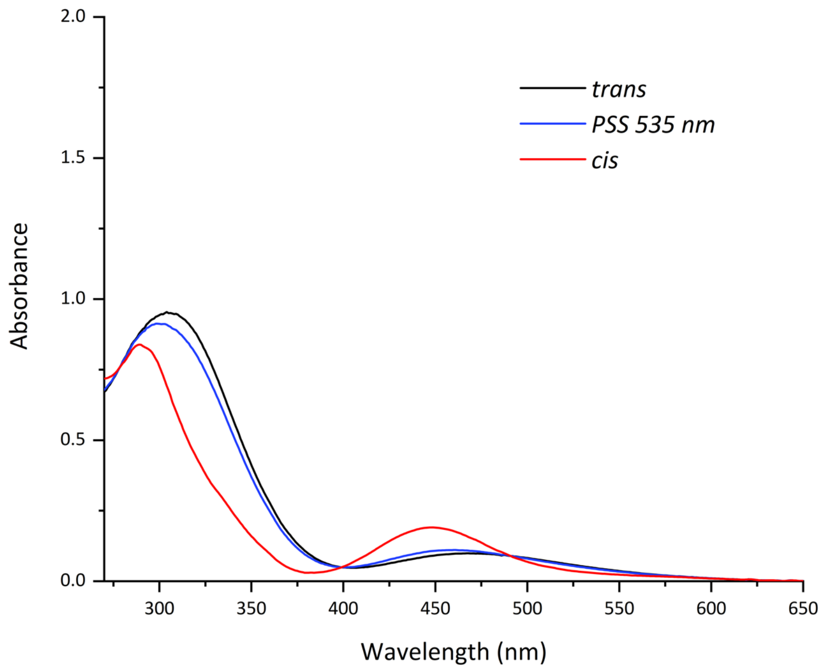

Figure 49. Electronic absorption spectrum of **10** ($1.13 \cdot 10^{-4}$ M in DMSO) before and after irradiation with Prizmatix FC6-LED-WL LED (535R LED, FWHM = 90 nm) until reaching the photostationary state. *Cis* spectra were approximated using the calculated epsilon for the *cis*-species multiplied by the concentration and the pathlength.

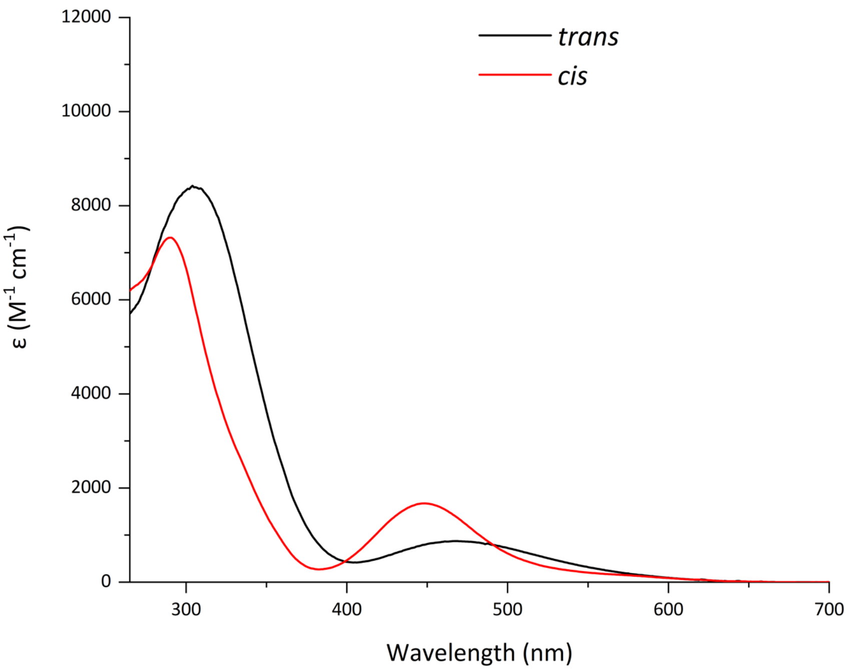

Figure 50. Molar extinction coefficient for *trans*-**10** and *cis*-**10** in DMSO.

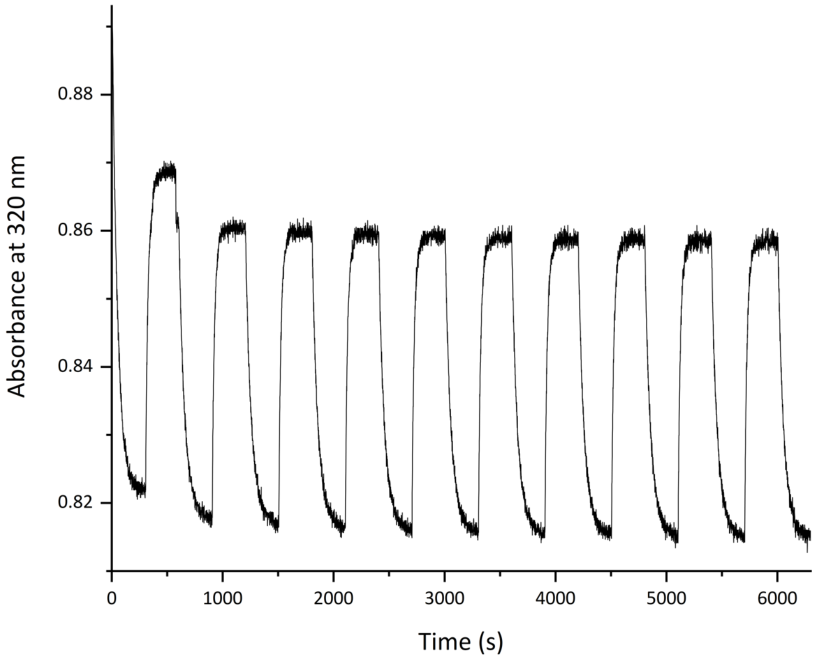

Figure 51. Absorbance at 320 nm of compound **10** ($1.13 \cdot 10^{-4}$ M in DMSO) upon alternating (*trans* = 300 seconds, *cis* = 300 seconds) irradiation with 535 nm and 420 nm light.

Thermal decay and Eyring plots 1 - 10

In a typical experiment, 1.0 mg of compound **1** – **10** was dissolved in 0.6 mL [D_6]DMSO and irradiated for 1 h in a custom built NMR tube holder with a Sahlmann cooled 3 x LXML PM01 0100 (526 nm, FWHM = 35.1 nm, power output = 810 mW) until reaching the photostationary state. The sample was then transferred to a Varian NMR 200, after which spectra were recorded every 240 seconds while maintaining a preset temperature until full *cis* to *trans*-isomerization was acquired. Obtained spectra were then processed in Mestrenova 14.1.2, followed by data processing in OriginPro2018. Kinetic parameters were acquired by plotting the integration area of an NMR signal of either the *cis* or *trans* species *vs* time in seconds, followed by line-fitting using the 'ExpGro1' or 'ExpDec1' function in OriginPro2018. The slope in the thermal decay graphs equal k^{-1}. By repeating this process for each temperature an Eyring plot was acquired:

Slope = $\frac{-\Delta H^{\ddagger}}{R}$

y–intercept = $\frac{-\Delta S^{\ddagger}}{R}$ + ln $\frac{k_B}{h}$

Gibbs energy of activation = $\Delta G^{\ddagger} = \Delta H^{\ddagger} - T\Delta S^{\ddagger}$.

Boltzmann constant = k_B = $1.381 \cdot 10^{-23}$ J · K^{-1}
Planck constant = h = $6.626070 \cdot 10^{-34}$ J · s
Gas constant = R = 8.31446261815324 J · K^{-1} · mol^{-1}

Thermal decay plots for compound 1

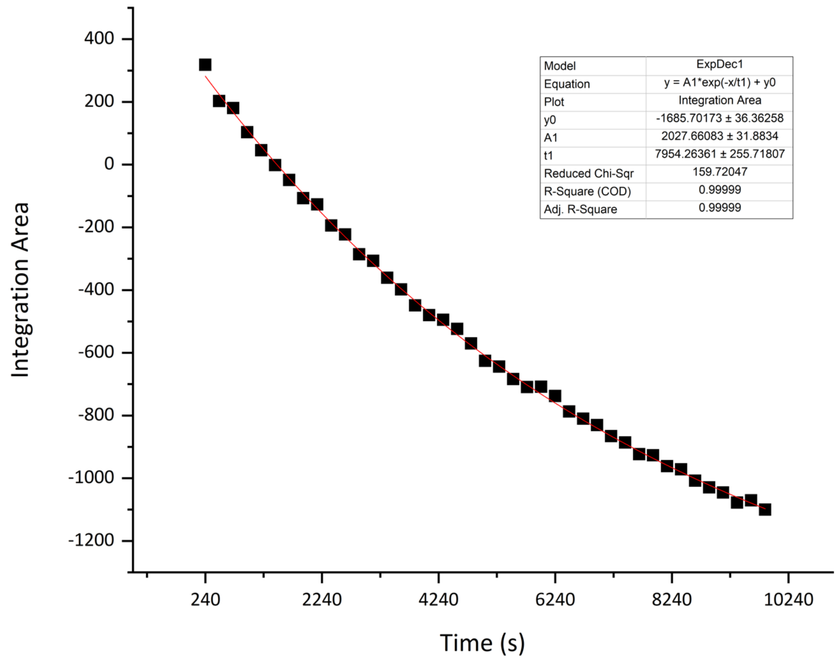

Figure 52. Thermal decay of **1** in DMSO after reaching the photo stationary state (λ_{ex} = 526 nm) at 40 °C. ^{1}HNMR spectra were collected every 240 seconds until (almost) full conversion to the trans isomer was observed.

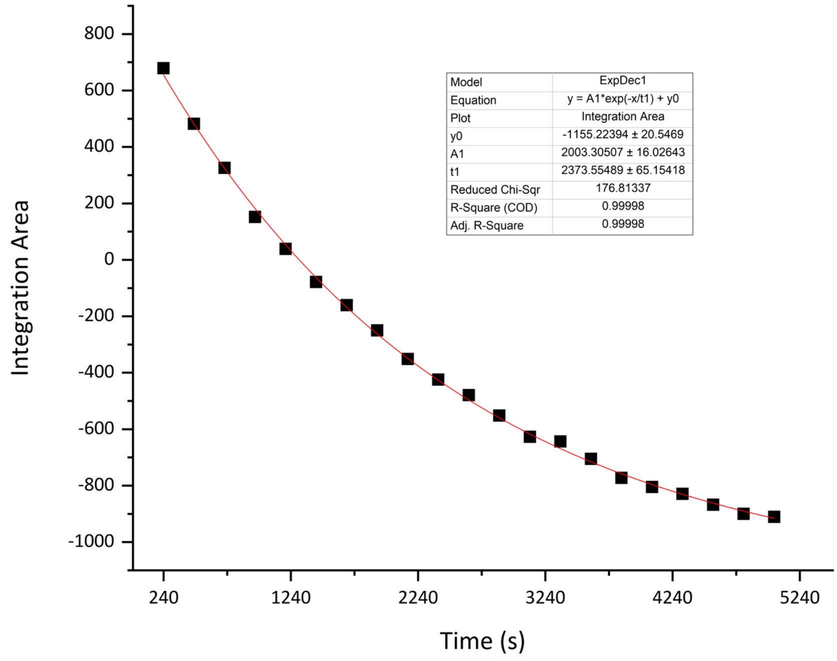

Figure 53. Thermal decay of **1** in DMSO after reaching the photo stationary state (λ_{ex} = 526 nm) at 50 °C. ^{1}HNMR spectra were collected every 240 seconds until (almost) full conversion to the trans isomer was observed.

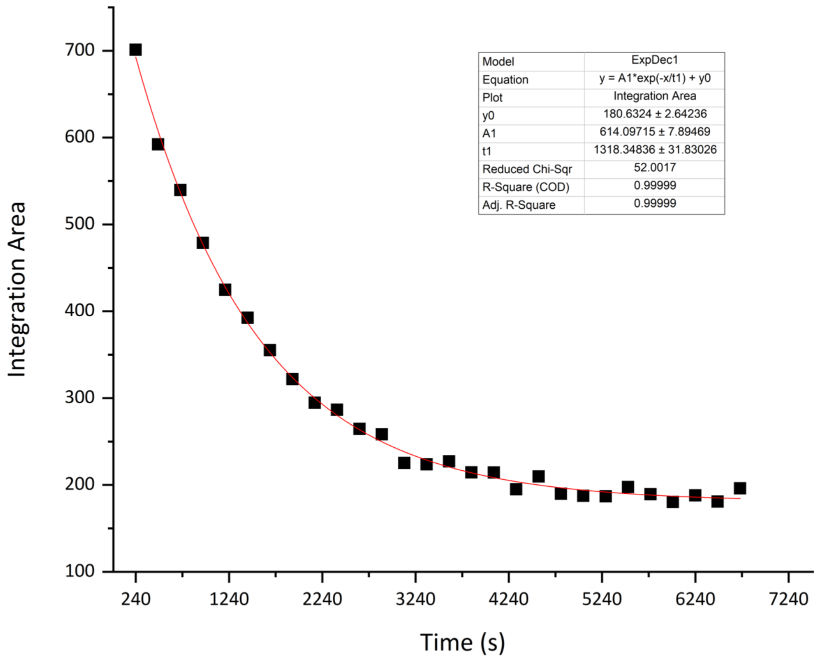

Figure 54. Thermal decay of **1** in DMSO after reaching the photo stationary state (λ_{ex} = 526 nm) at 55 °C. ^{1}HNMR spectra were collected every 240 seconds until (almost) full conversion to the trans isomer was observed.

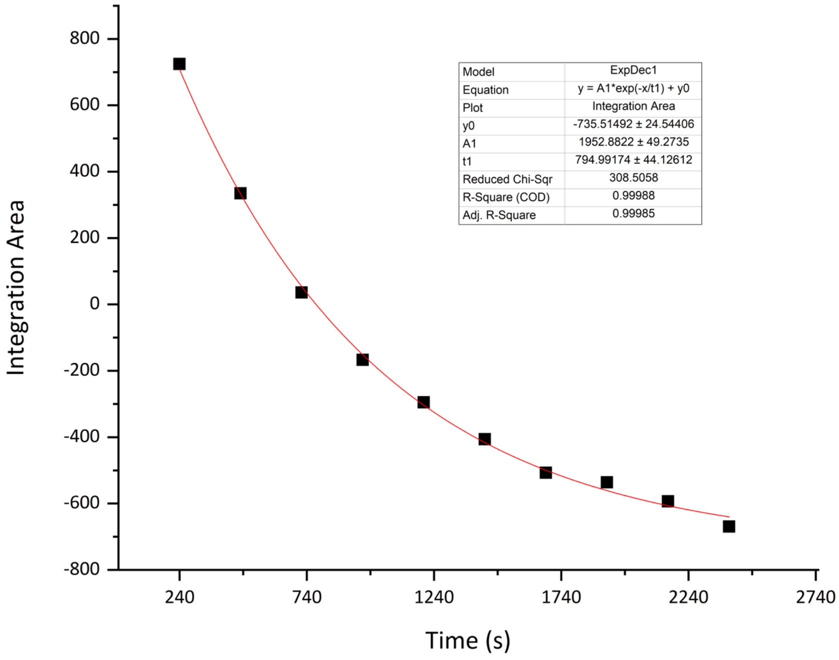

Figure 55. Thermal decay of **1** in DMSO after reaching the photo stationary state (λ_{ex} = 526 nm) at 60 °C. ^{1}HNMR spectra were collected every 240 seconds until (almost) full conversion to the trans isomer was observed.

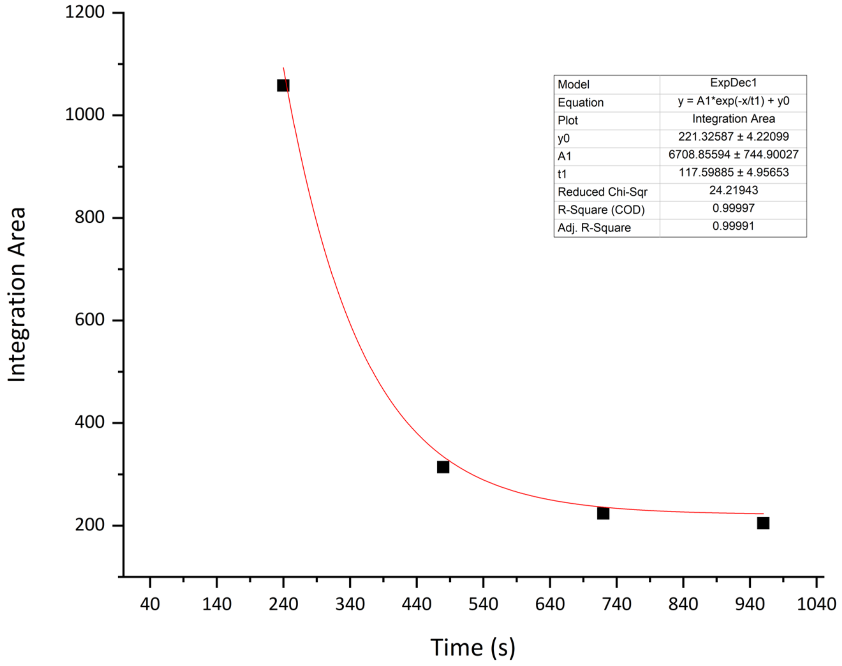

Figure 56. Thermal decay of **1** in DMSO after reaching the photo stationary state (λ_{ex} = 526 nm) at 70 °C. ^{1}HNMR spectra were collected every 240 seconds until (almost) full conversion to the trans isomer was observed.

Eyring plot for compound 1

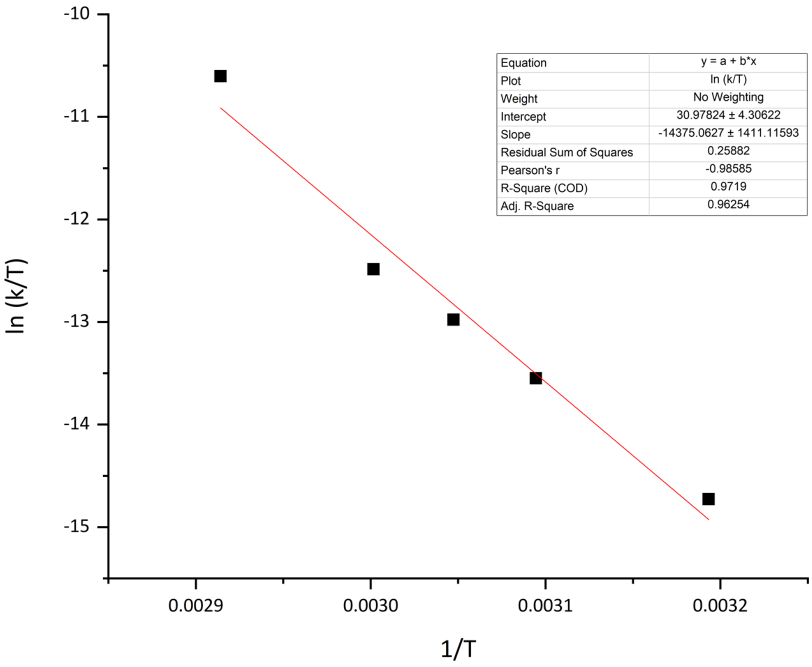

Figure 57. Eyring plot of ln (k/T) vs 1/T for compound **1**. Adjusted R^2 = 0.96.

Thermal decay plots for compound 2

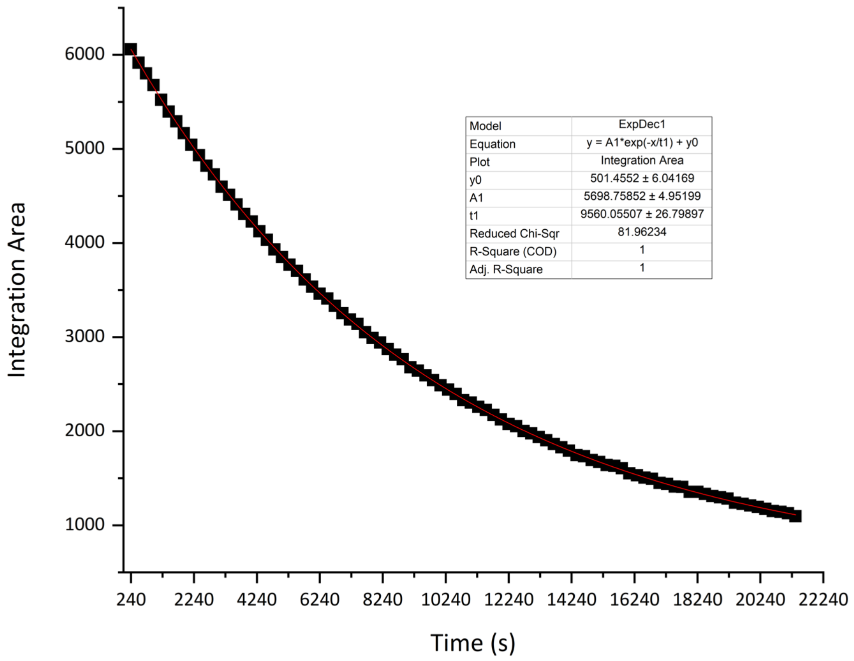

Figure 58. Thermal decay of **2** in DMSO after reaching the photo stationary state (λ_{ex} = 526 nm) at 65 °C. ^{1}HNMR spectra were collected every 240 seconds until (almost) full conversion to the trans isomer was observed.

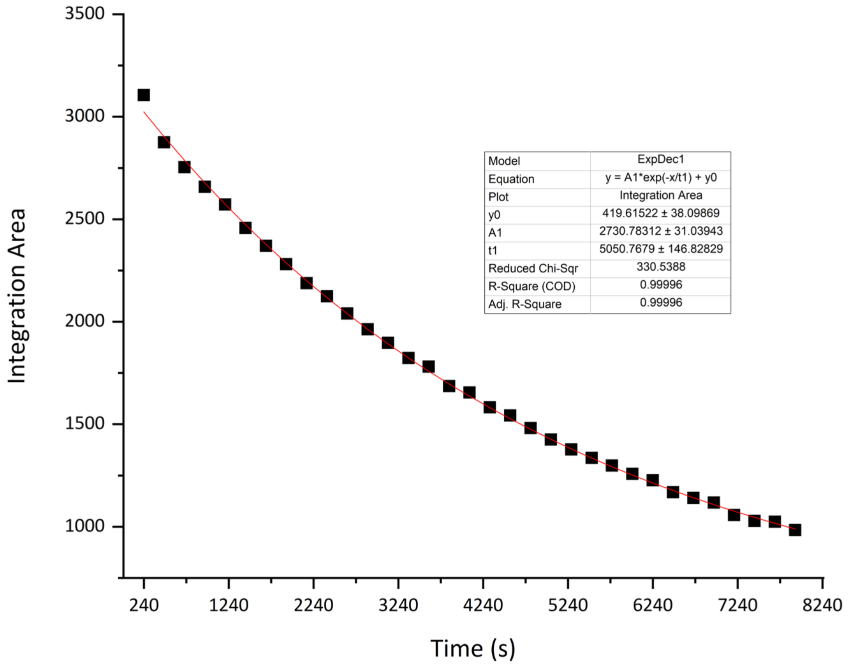

Figure 59. Thermal decay of **2** in DMSO after reaching the photo stationary state (λ_{ex} = 526 nm) at 70 °C. ^{1}HNMR spectra were collected every 240 seconds until (almost) full conversion to the trans isomer was observed.

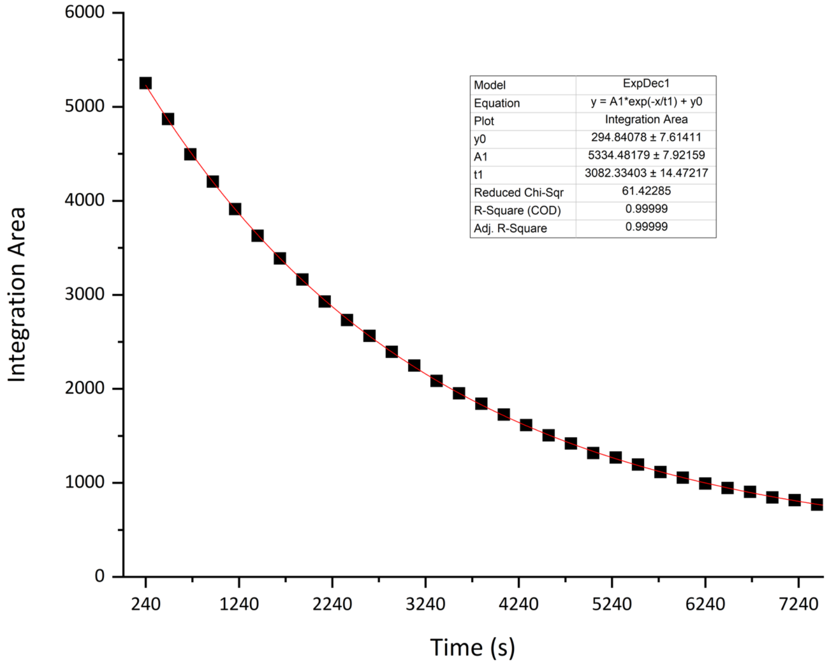

Figure 60. Thermal decay of **2** in DMSO after reaching the photo stationary state (λ_{ex} = 526 nm) at 75 °C. ^{1}HNMR spectra were collected every 240 seconds until (almost) full conversion to the trans isomer was observed.

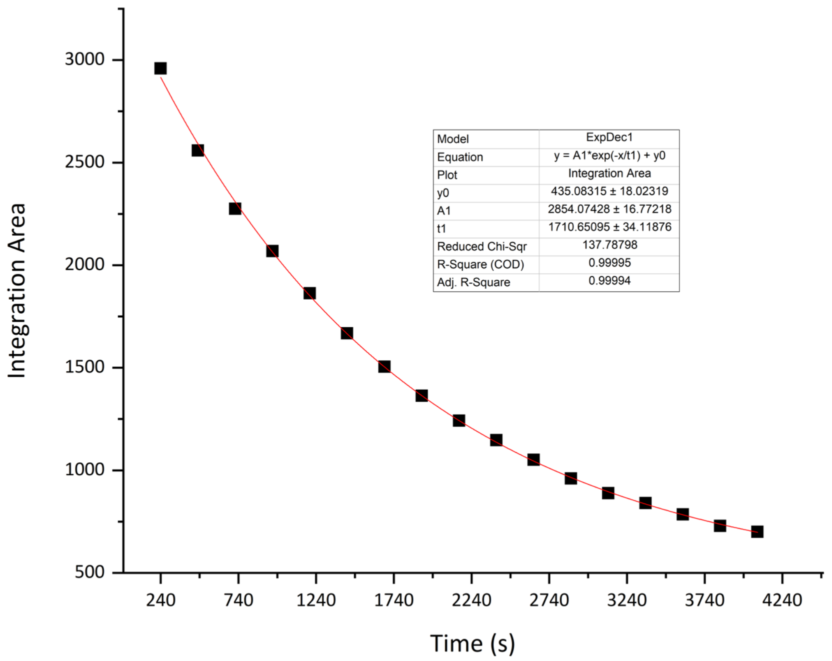

Figure 61. Thermal decay of **2** in DMSO after reaching the photo stationary state (λ_{ex} = 526 nm) at 80 °C. ^{1}HNMR spectra were collected every 240 seconds until (almost) full conversion to the trans isomer was observed.

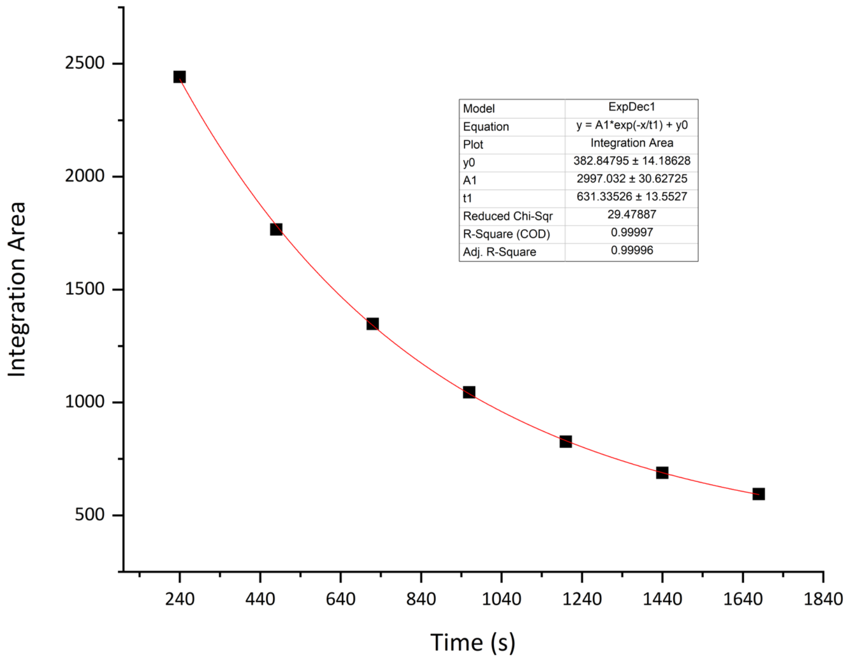

Figure 62. Thermal decay of **2** in DMSO after reaching the photo stationary state (λ_{ex} = 526 nm) at 90 °C. ^{1}HNMR spectra were collected every 240 seconds until (almost) full conversion to the trans isomer was observed.

Eyring plot for compound 2

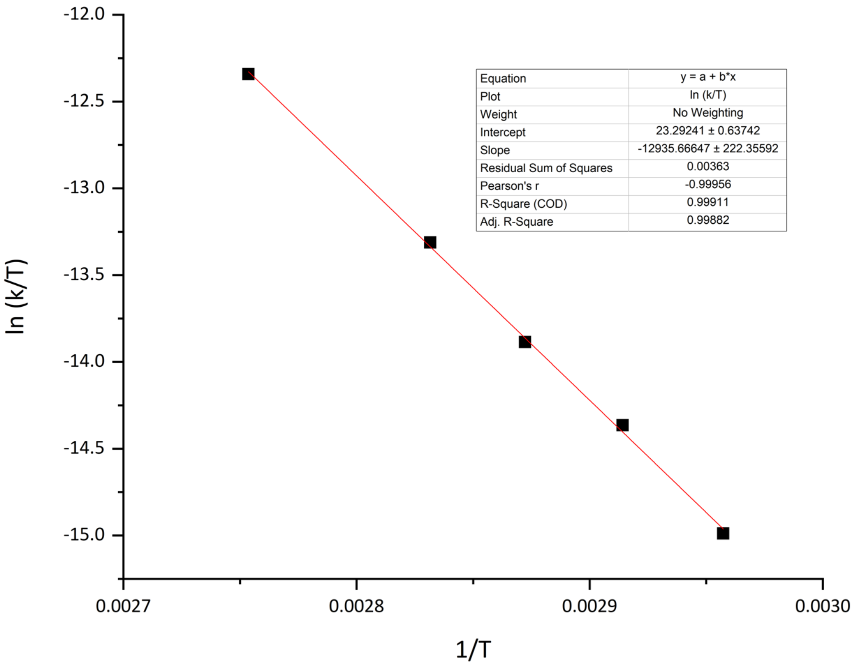

Figure 63. Eyring plot of ln (k/T) vs 1/T for compound **2**. Adjusted R^2 = 1.00.

Thermal decay plots for compound 3

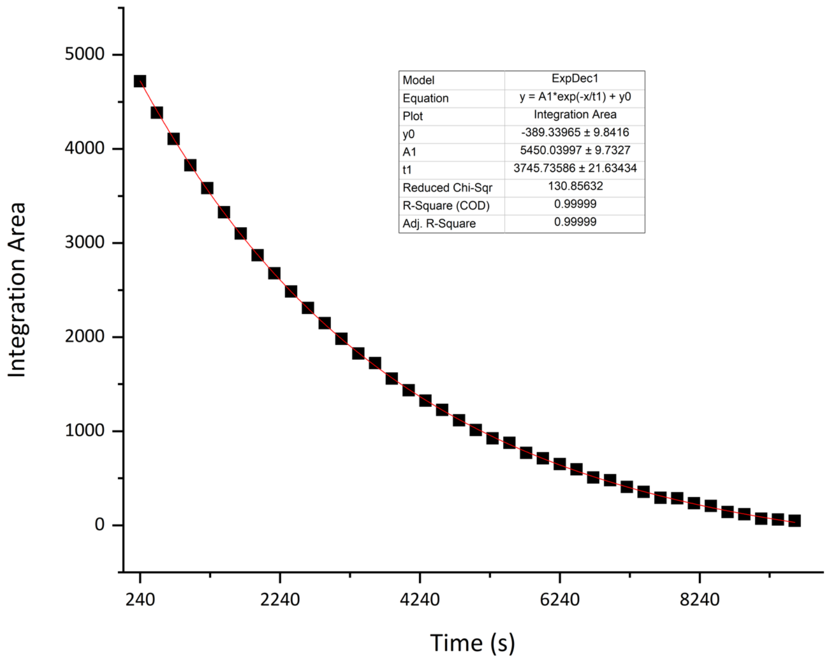

Figure 64. Thermal decay of **3** in DMSO at 80 °C after reaching the photo stationary state (λ_{ex} = 526 nm). ^{1}HNMR spectra were collected every 240 seconds until (almost) full conversion to the trans isomer was observed.

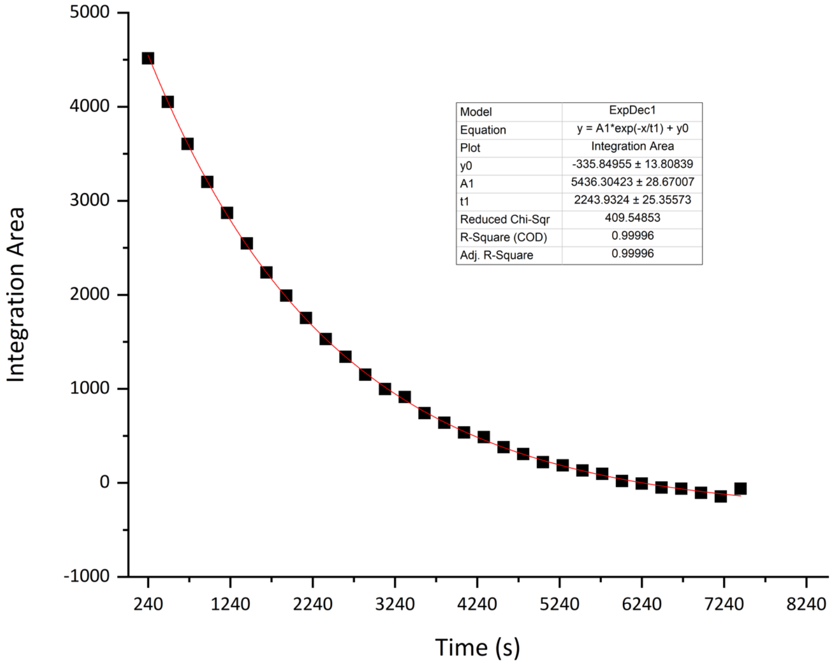

Figure 65. Thermal decay of **3** in DMSO at 85 °C after reaching the photo stationary state (λ_{ex} = 526 nm). ^{1}HNMR spectra were collected every 240 seconds until (almost) full conversion to the trans isomer was observed.

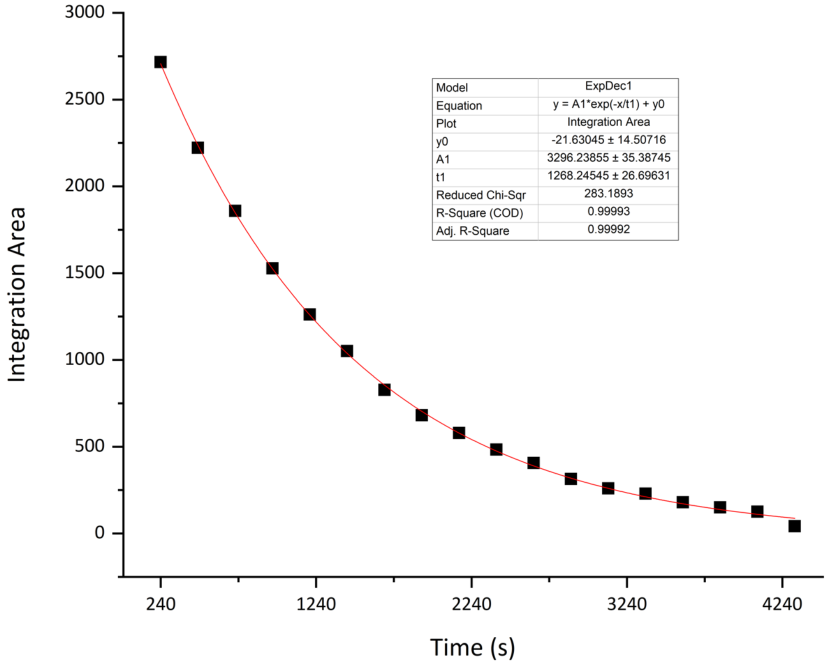

Figure 66. Thermal decay of **3** in DMSO at 90 °C after reaching the photo stationary state (λ_{ex} = 526 nm). ^{1}HNMR spectra were collected every 240 seconds until (almost) full conversion to the trans isomer was observed.

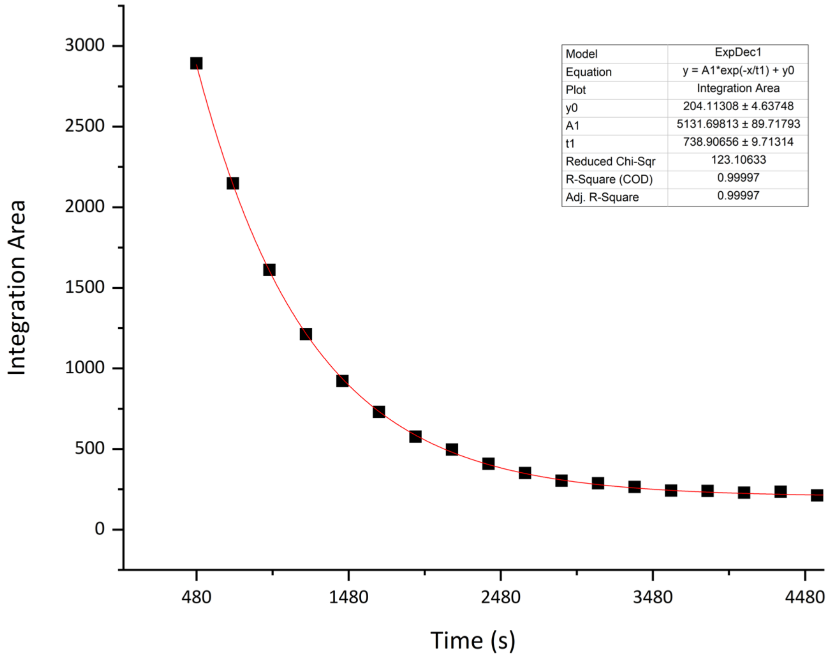

Figure 67. Thermal decay of **3** in DMSO at 95 °C after reaching the photo stationary state (λ_{ex} = 526 nm). ^{1}HNMR spectra were collected every 240 seconds until (almost) full conversion to the trans isomer was observed.

Eyring plot for compound 3

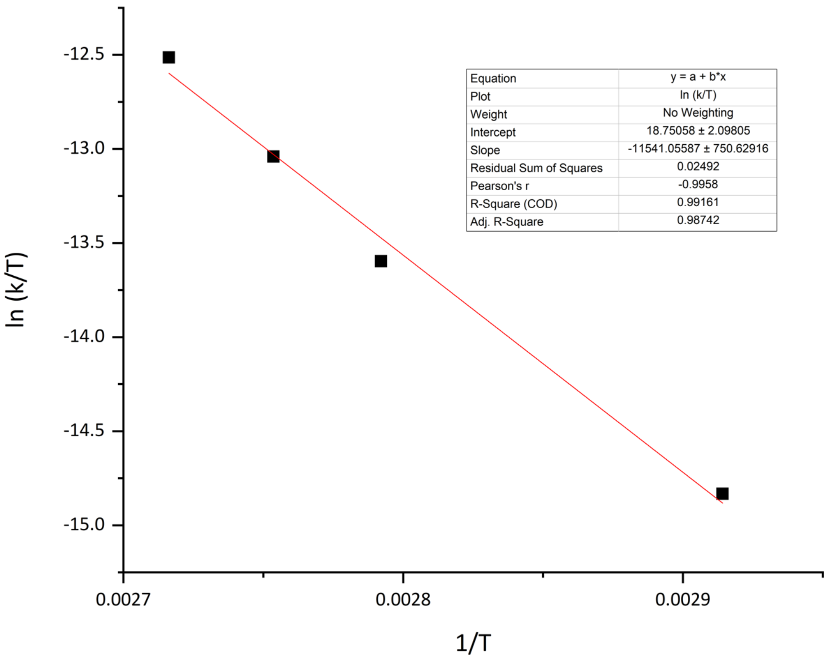

Figure 68. Eyring plot of ln (k/T) vs 1/T for compound **3**. Adjusted R^2 = 0.99.

Thermal decay plots for compound 4

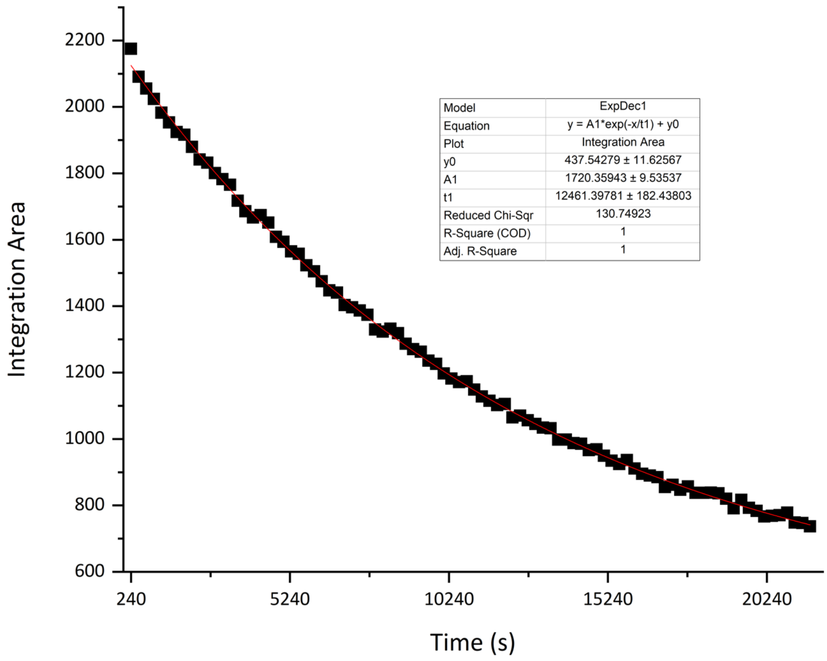

Figure 69. Thermal decay of **4** in DMSO at 70 °C after reaching the photo stationary state (λ_{ex} = 526 nm). ^{1}HNMR spectra were collected every 240 seconds until (almost) full conversion to the trans isomer was observed.

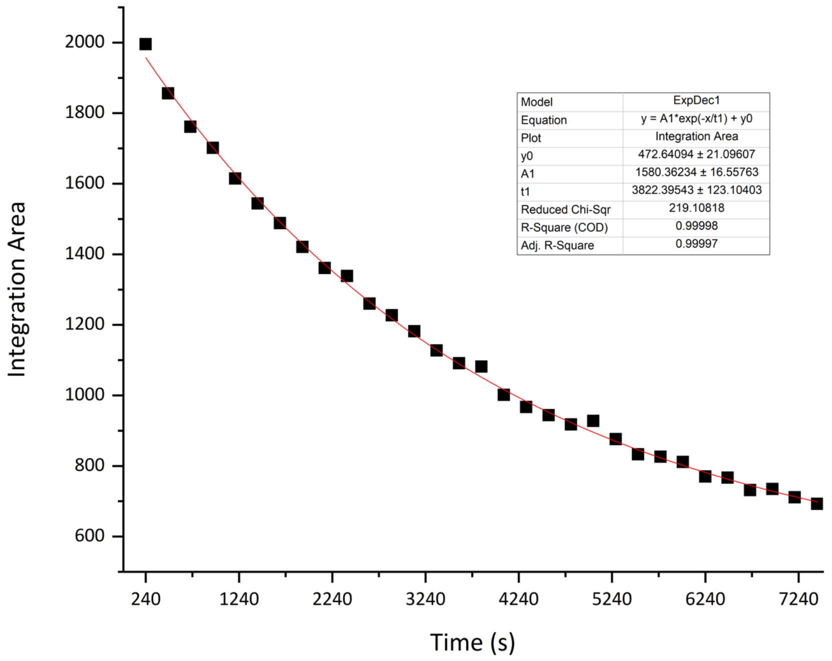

Figure 70. Thermal decay of **4** in DMSO at 80 °C after reaching the photo stationary state (λ_{ex} = 526 nm). ^{1}HNMR spectra were collected every 240 seconds until (almost) full conversion to the trans isomer was observed.

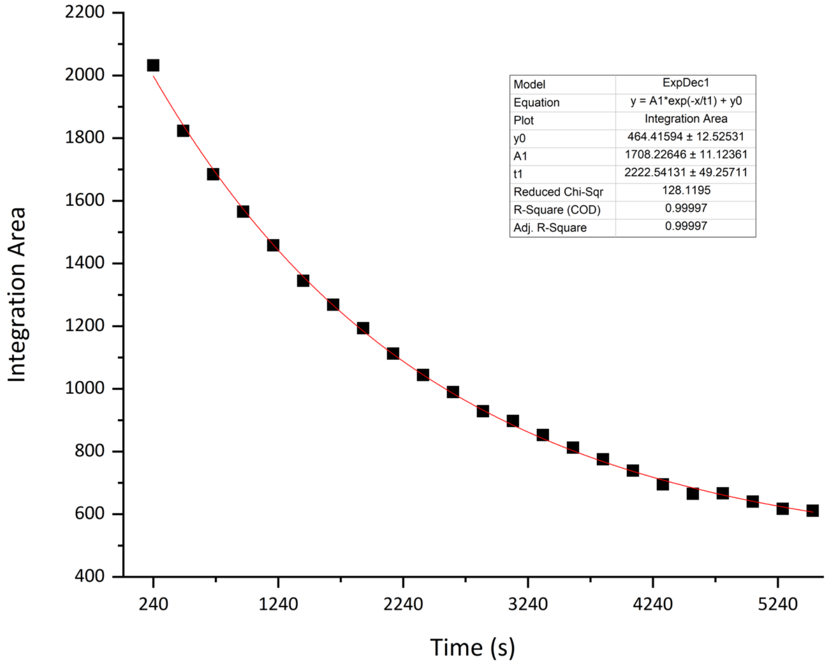

Figure 71. Thermal decay of **4** in DMSO at 85 °C after reaching the photo stationary state (λ_{ex} = 526 nm). ^{1}HNMR spectra were collected every 240 seconds until (almost) full conversion to the trans isomer was observed.

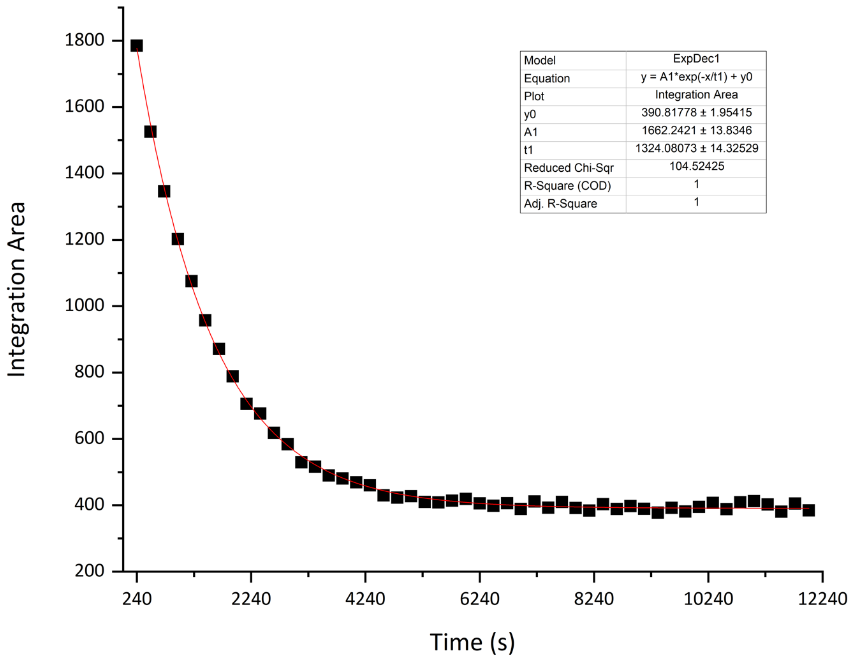

Figure 72. Thermal decay of **4** in DMSO at 90 °C after reaching the photo stationary state (λ_{ex} = 526 nm). ^{1}HNMR spectra were collected every 240 seconds until (almost) full conversion to the trans isomer was observed.

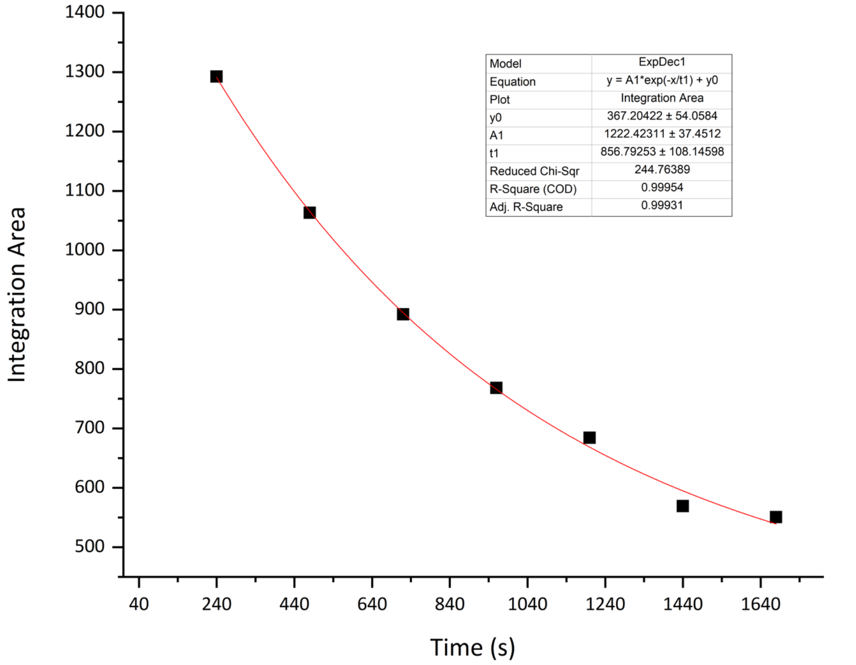

Figure 73. Thermal decay of **4** in DMSO at 95 °C after reaching the photo stationary state (λ_{ex} = 526 nm). ^{1}HNMR spectra were collected every 240 seconds until (almost) full conversion to the trans isomer was observed.

Eyring plot for compound 4

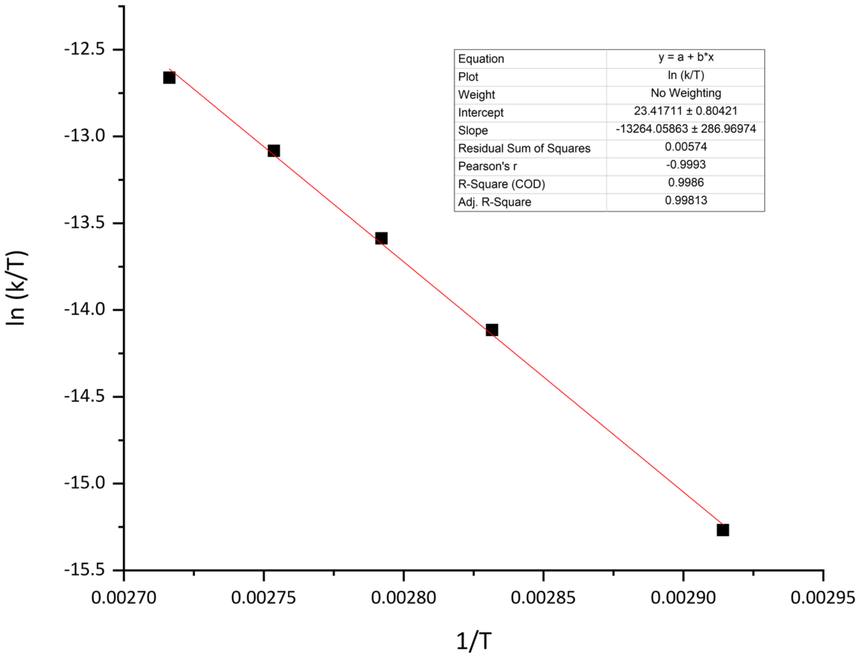

Figure 74. Eyring plot of ln (k/T) vs 1/T for compound **4**. Adjusted R^2 = 0.99.

Thermal decay plots for compound 5

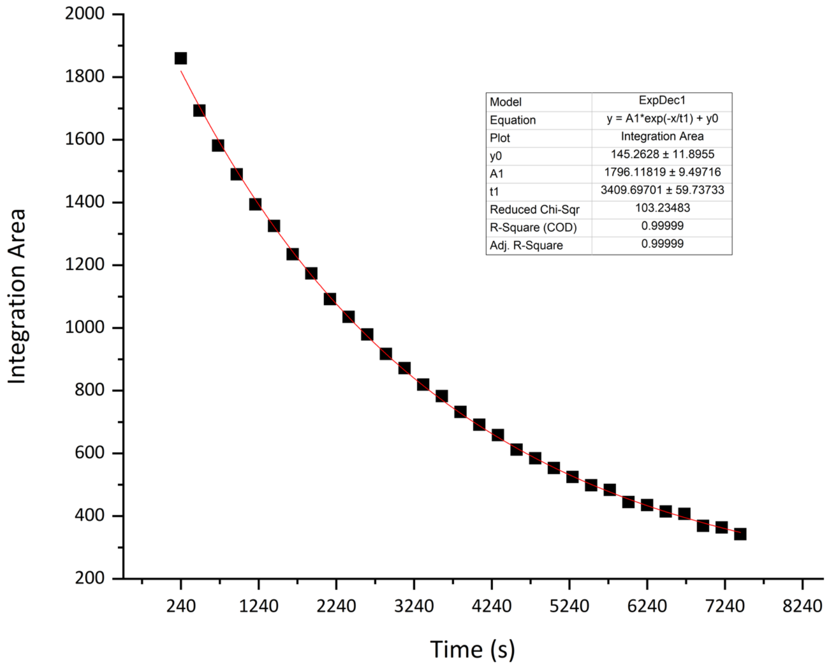

Figure 75. Thermal decay of **5** in DMSO at 70 °C after reaching the photo stationary state (λ_{ex} = 526 nm). ^{1}HNMR spectra were collected every 240 seconds until (almost) full conversion to the trans isomer was observed.

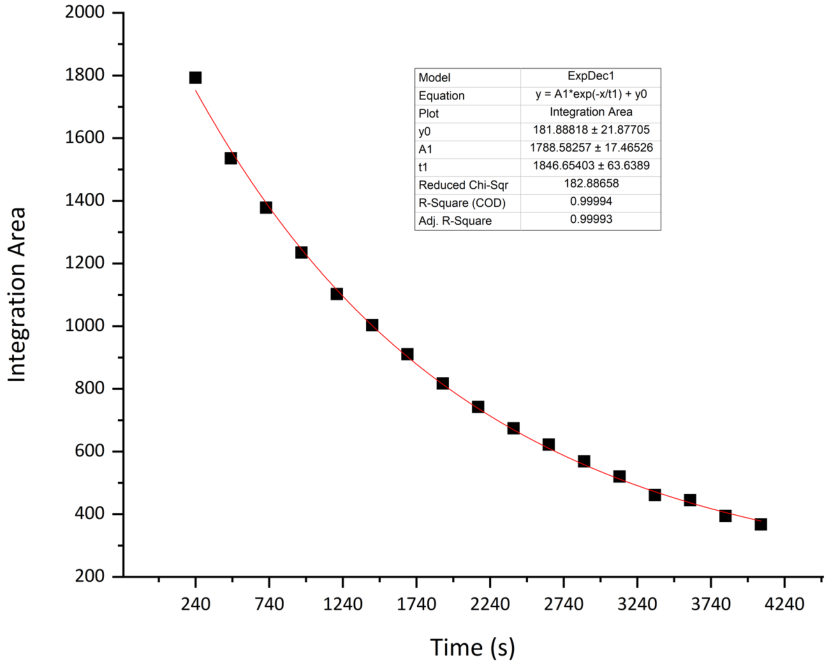

Figure 76. Thermal decay of **5** in DMSO at 75 °C after reaching the photo stationary state (λ_{ex} = 526 nm). ^{1}HNMR spectra were collected every 240 seconds until (almost) full conversion to the trans isomer was observed.

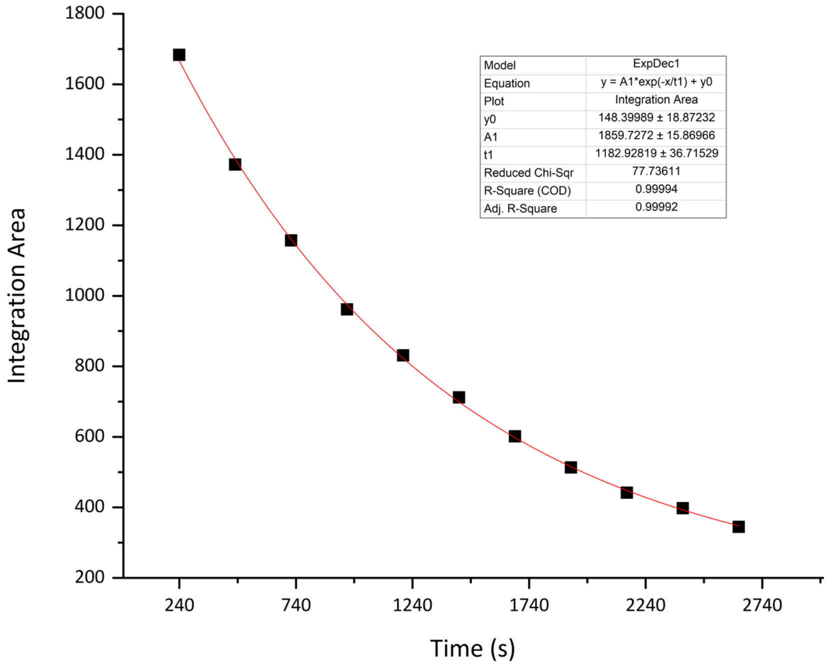

Figure 77. Thermal decay of **5** in DMSO at 80 °C after reaching the photo stationary state (λ_{ex} = 526 nm). ^{1}HNMR spectra were collected every 240 seconds until (almost) full conversion to the trans isomer was observed.

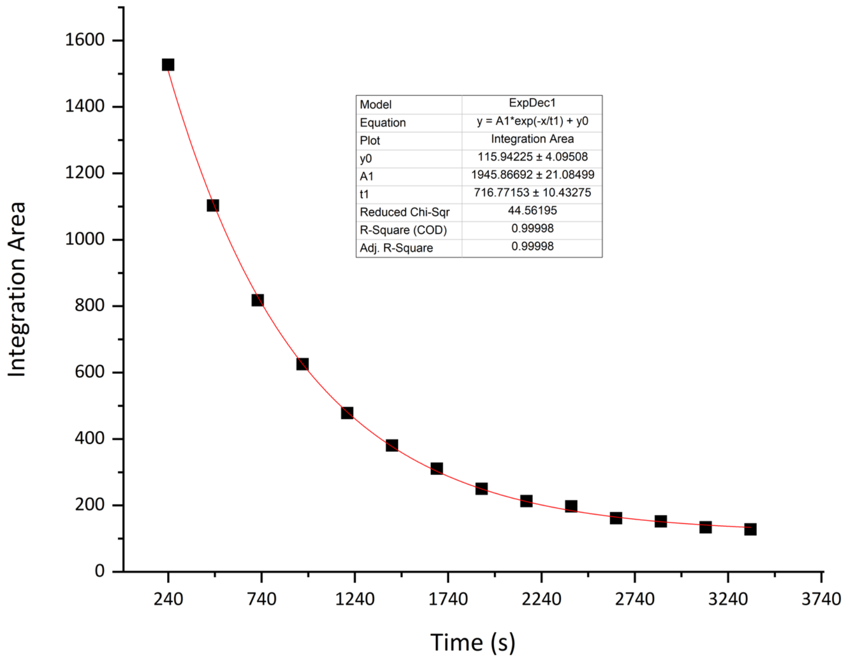

Figure 78. Thermal decay of **5** in DMSO at 85 °C after reaching the photo stationary state (λ_{ex} = 526 nm). ^{1}HNMR spectra were collected every 240 seconds until (almost) full conversion to the trans isomer was observed.

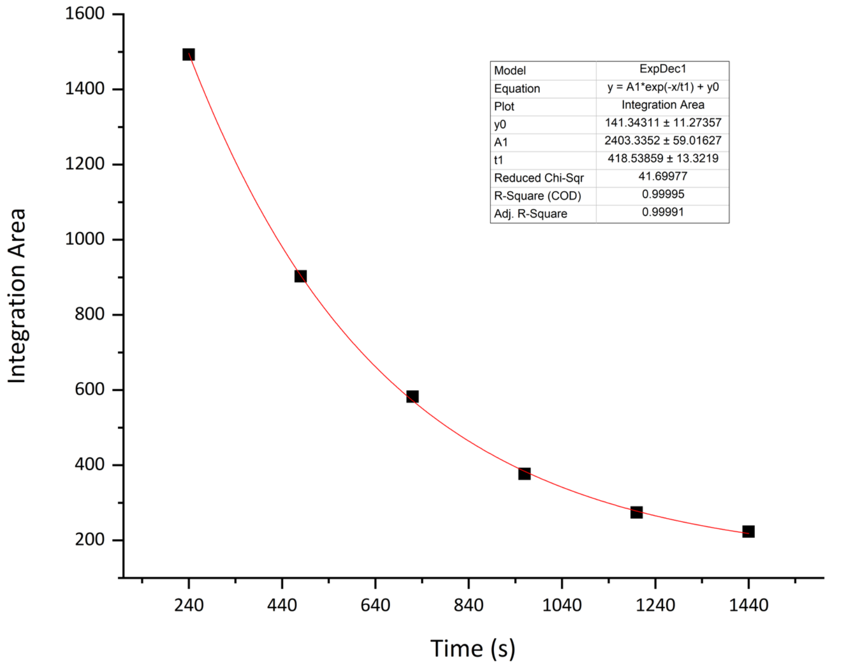

Figure 79. Thermal decay of **5** in DMSO at 90 °C after reaching the photo stationary state (λ_{ex} = 526 nm). ^{1}HNMR spectra were collected every 240 seconds until (almost) full conversion to the trans isomer was observed.

Eyring plot for compound 5

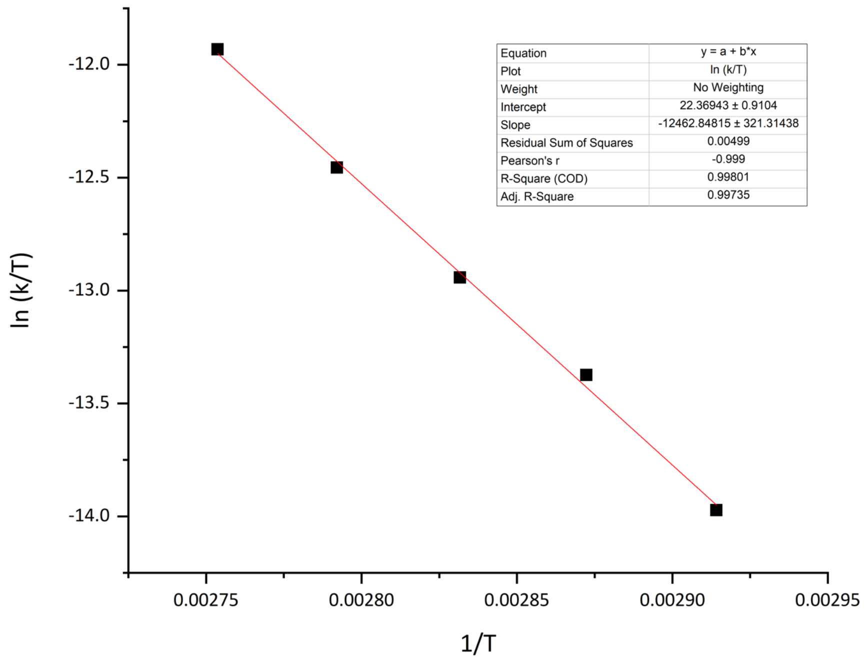

Figure 80. Eyring plot of ln (k/T) vs 1/T for compound **5**. Adjusted R^2 = 1.00.

Thermal decay plots for compound 6

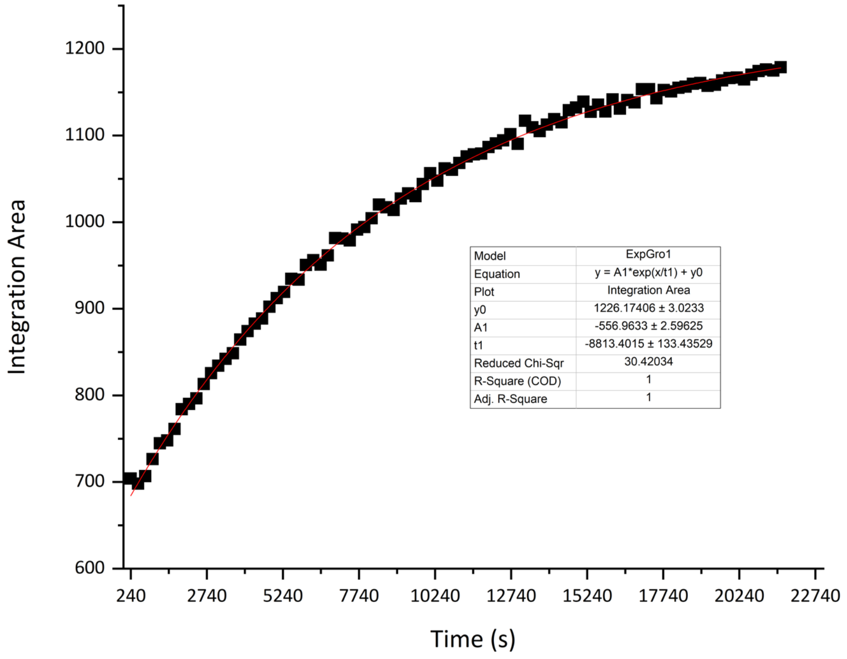

Figure 81. Thermal decay of **6** in DMSO at 70 °C after reaching the photo stationary state (λ_{ex} = 526 nm). ^{1}HNMR spectra were collected every 240 seconds until (almost) full conversion to the trans isomer was observed.

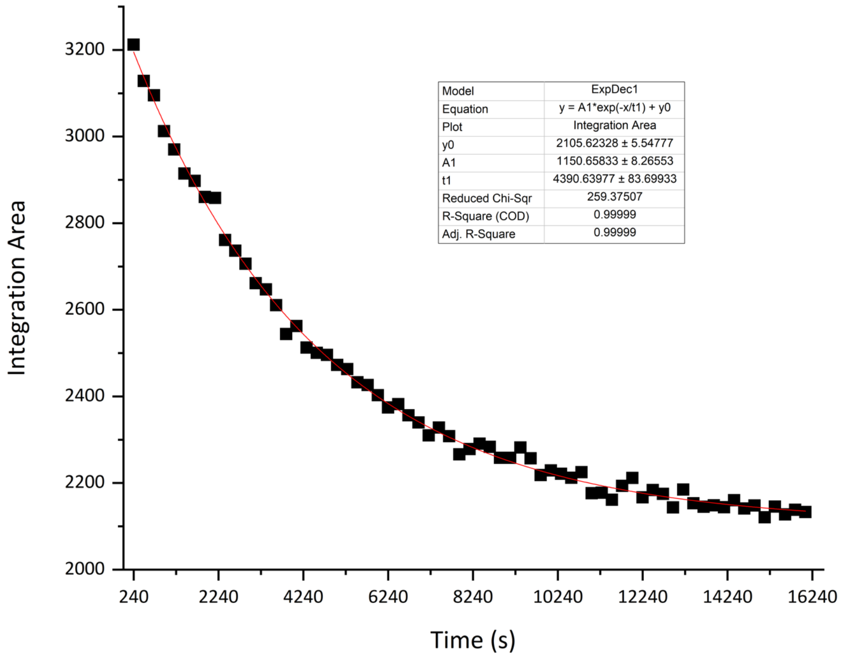

Figure 82. Thermal decay of **6** in DMSO at 75 °C after reaching the photo stationary state (λ_{ex} = 526 nm). ^{1}HNMR spectra were collected every 240 seconds until (almost) full conversion to the trans isomer was observed.

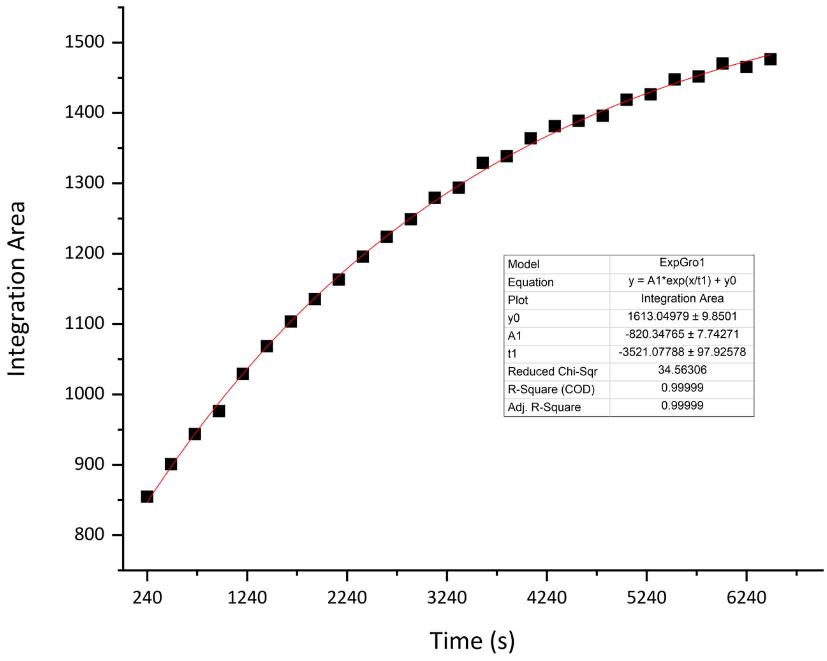

Figure 83. Thermal decay of **6** in DMSO at 80 °C after reaching the photo stationary state (λ_{ex} = 526 nm). ^{1}HNMR spectra were collected every 240 seconds until (almost) full conversion to the trans isomer was observed.

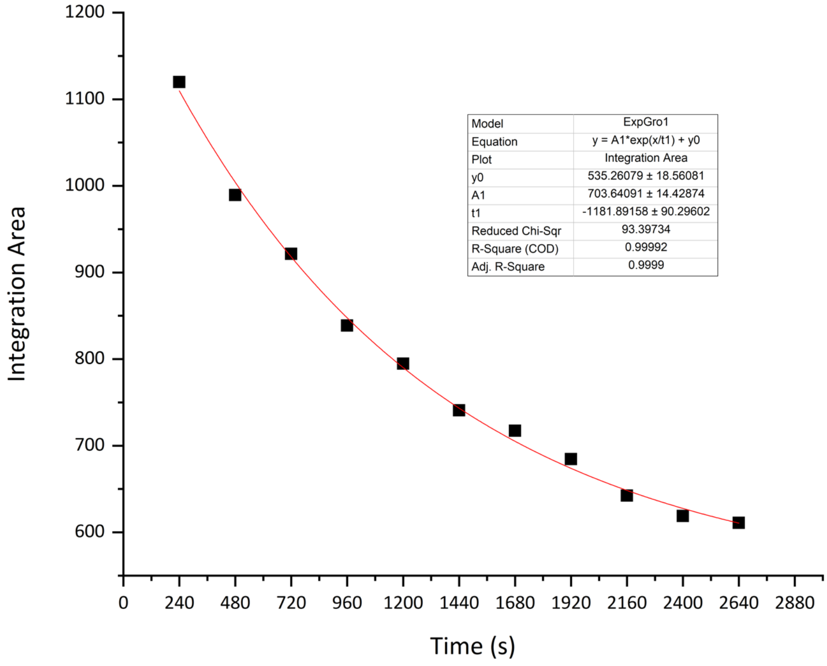

Figure 84. Thermal decay of **6** in DMSO at 85 °C after reaching the photo stationary state (λ_{ex} = 526 nm). ^{1}HNMR spectra were collected every 240 seconds until (almost) full conversion to the trans isomer was observed.

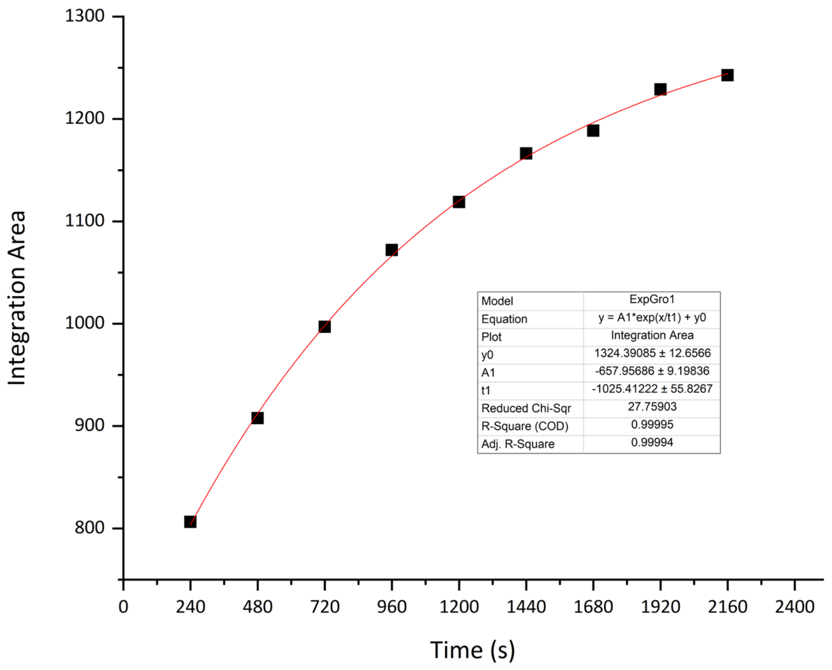

Figure 85. Thermal decay of **6** in DMSO at 90 °C after reaching the photo stationary state (λ_{ex} = 526 nm). ^{1}HNMR spectra were collected every 240 seconds until (almost) full conversion to the trans isomer was observed.

Eyring plot for compound 6

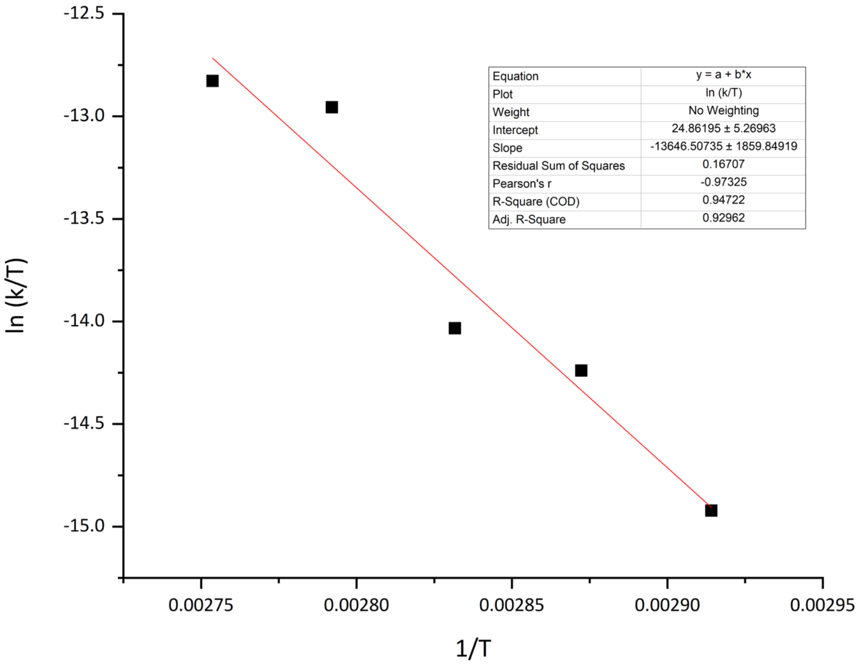

Figure 86. Eyring plot of ln (k/T) vs 1/T for compound **5**. Adjusted R^2 = 0.93.

Thermal decay plots for compound 7

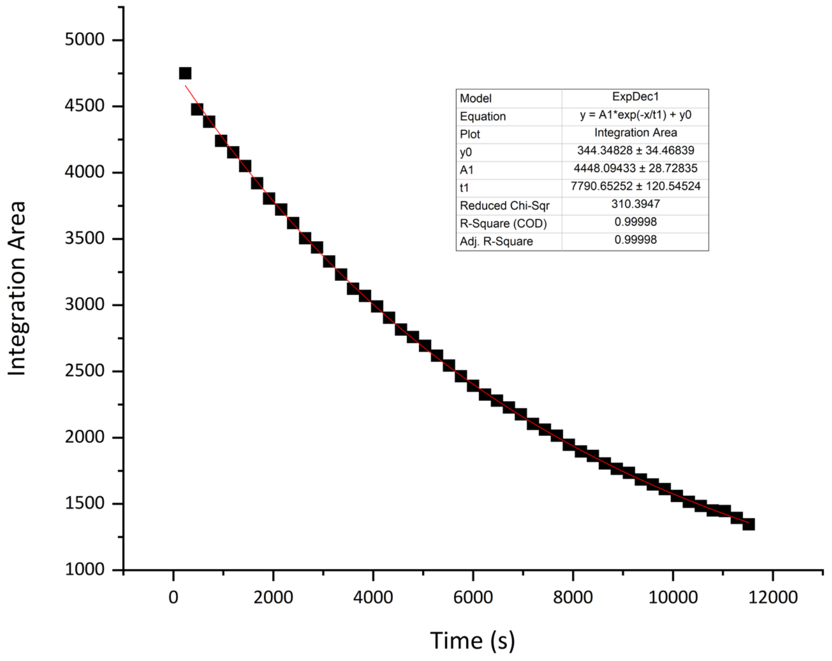

Figure 87. Thermal decay of **7** in DMSO at 70 °C after reaching the photo stationary state (λ_{ex} = 526 nm). ^{1}HNMR spectra were collected every 240 seconds until (almost) full conversion to the trans isomer was observed.

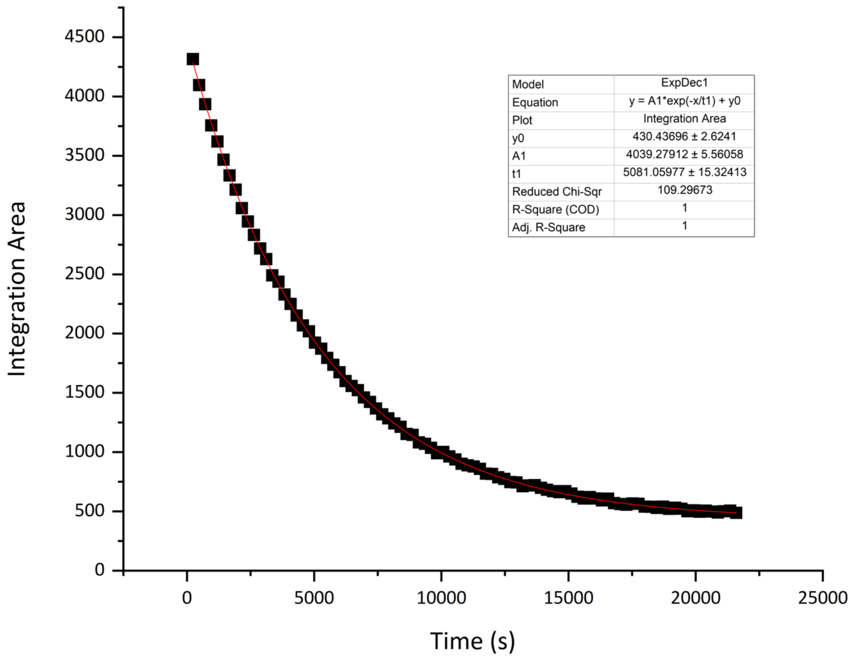

Figure 88. Thermal decay of **7** in DMSO at 75 °C after reaching the photo stationary state (λ_{ex} = 526 nm). ^{1}HNMR spectra were collected every 240 seconds until (almost) full conversion to the trans isomer was observed.

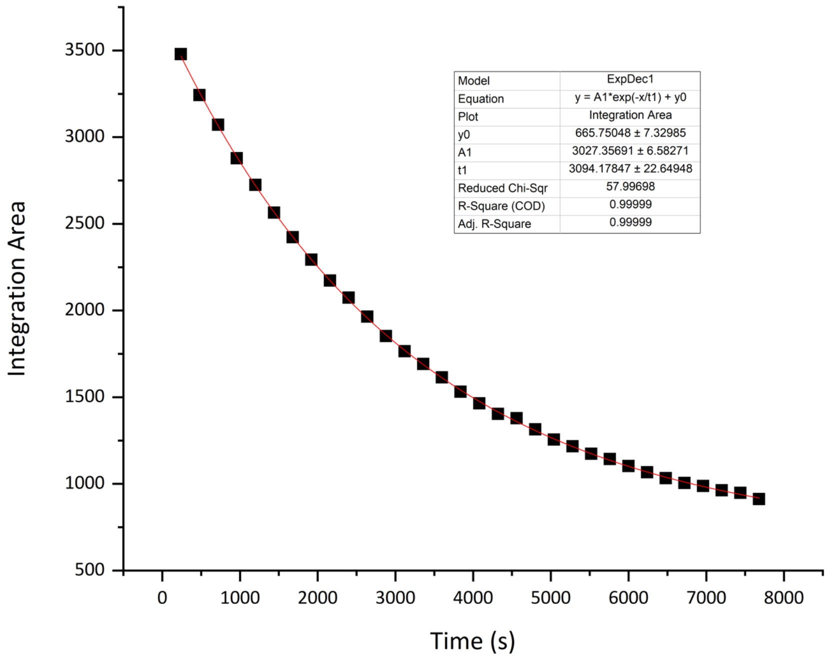

Figure 89. Thermal decay of **7** in DMSO at 80 °C after reaching the photo stationary state (λ_{ex} = 526 nm). ^{1}HNMR spectra were collected every 240 seconds until (almost) full conversion to the trans isomer was observed.

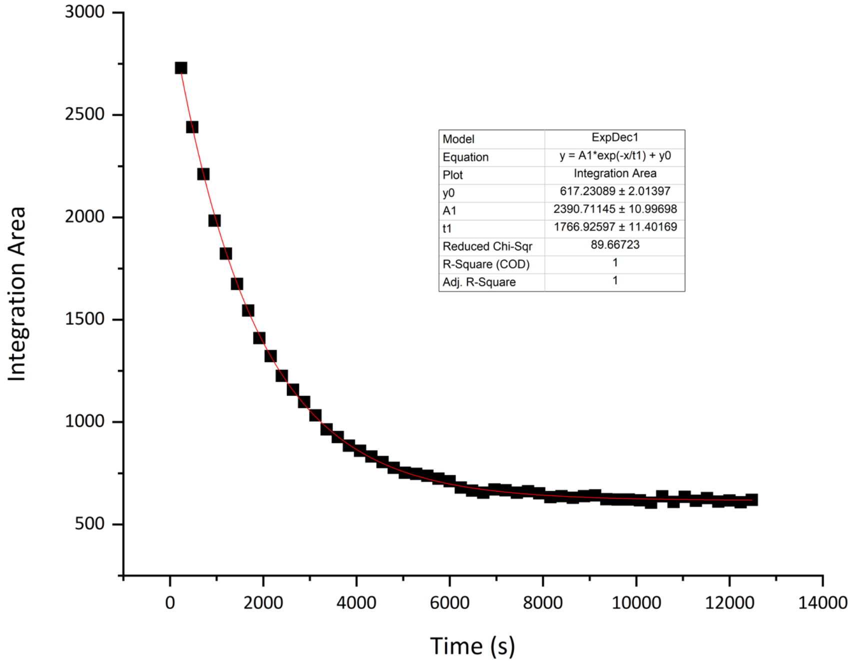

Figure 90. Thermal decay of **7** in DMSO at 85 °C after reaching the photo stationary state (λ_{ex} = 526 nm). ^{1}HNMR spectra were collected every 240 seconds until (almost) full conversion to the trans isomer was observed.

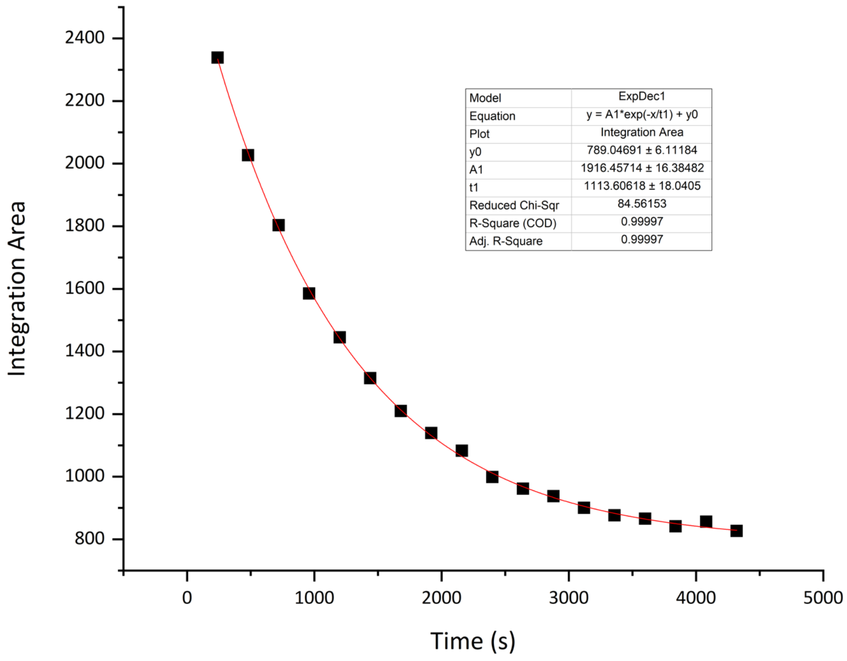

Figure 91. Thermal decay of **7** in DMSO at 90 °C after reaching the photo stationary state (λ_{ex} = 526 nm). ^{1}HNMR spectra were collected every 240 seconds until (almost) full conversion to the trans isomer was observed.

Eyring plot for compound 7

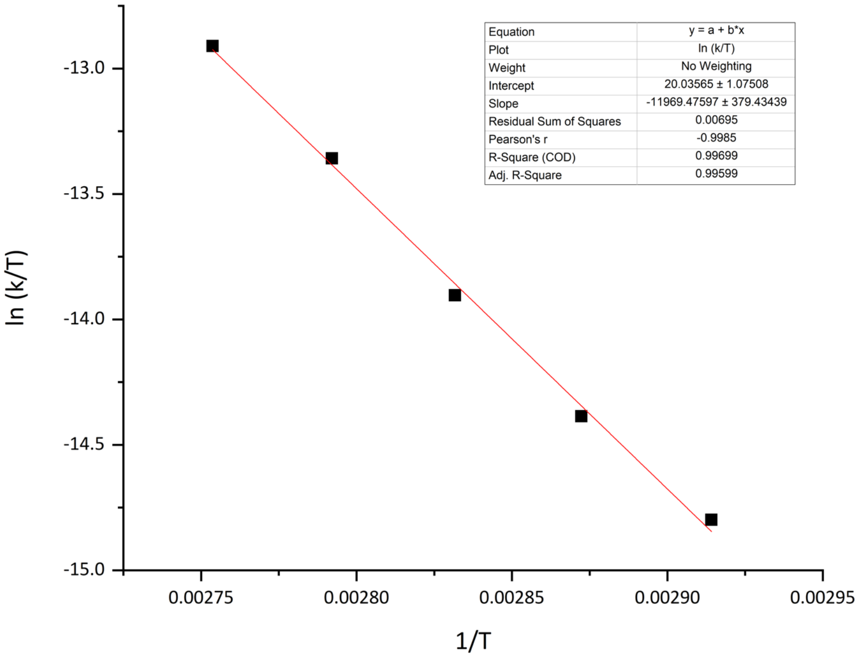

Figure 92. Eyring plot of ln (k/T) vs 1/T for compound **7**. Adjusted R^2 = 1.00.

Thermal decay plots for compound 8

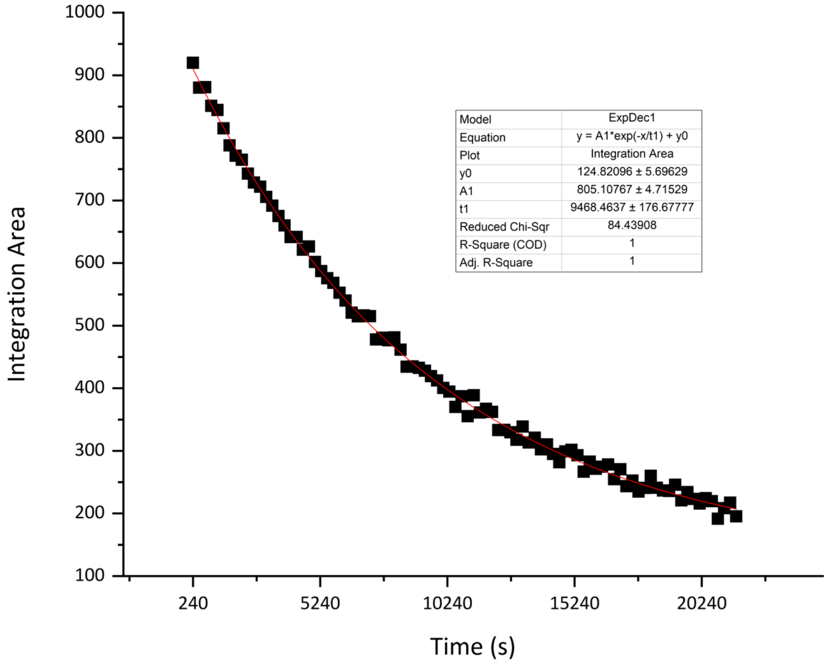

Figure 93. Thermal decay of **8** in DMSO at 70 °C after reaching the photo stationary state (λ_{ex} = 526 nm). ^{1}HNMR spectra were collected every 240 seconds until (almost) full conversion to the trans isomer was observed.

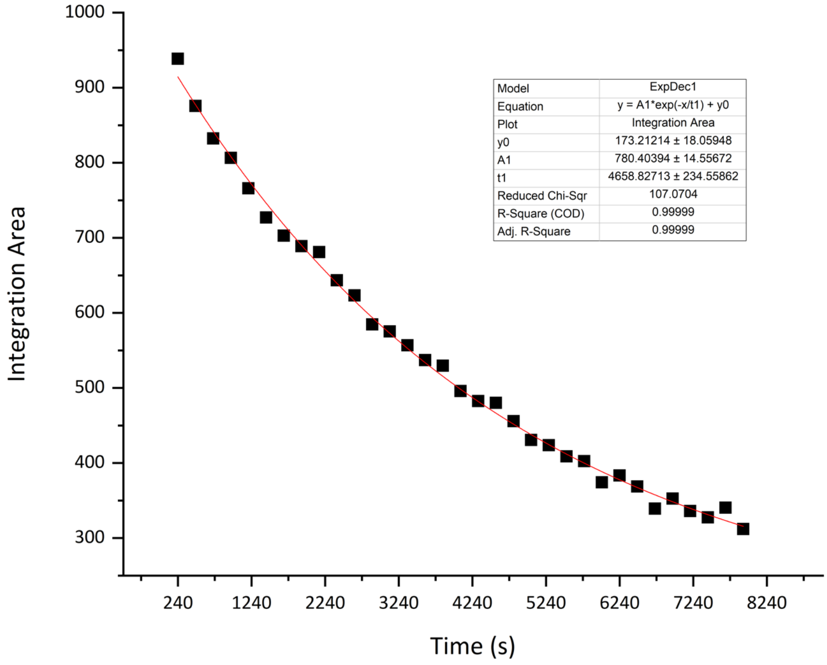

Figure 94. Thermal decay of **8** in DMSO at 75 °C after reaching the photo stationary state (λ_{ex} = 526 nm). ^{1}HNMR spectra were collected every 240 seconds until (almost) full conversion to the trans isomer was observed.

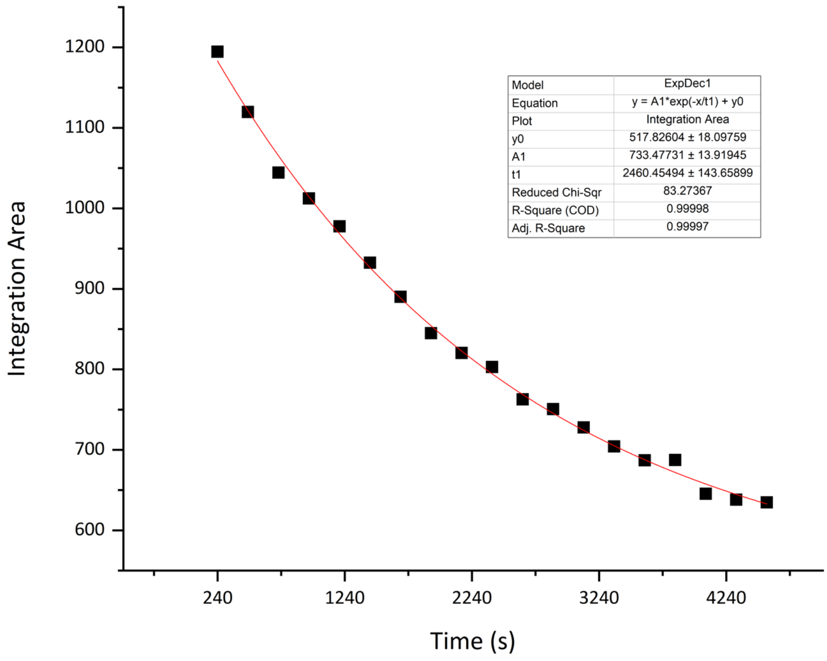

Figure 95. Thermal decay of **8** in DMSO at 80 °C after reaching the photo stationary state (λ_{ex} = 526 nm). ^{1}HNMR spectra were collected every 240 seconds until (almost) full conversion to the trans isomer was observed.

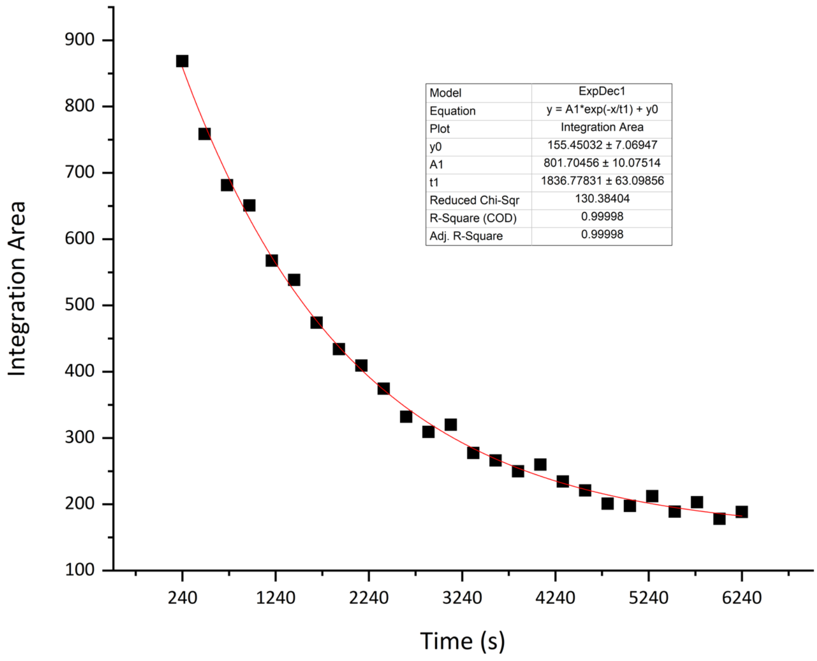

Figure 96. Thermal decay of **8** in DMSO at 85 °C after reaching the photo stationary state (λ_{ex} = 526 nm). ^{1}HNMR spectra were collected every 240 seconds until (almost) full conversion to the trans isomer was observed.

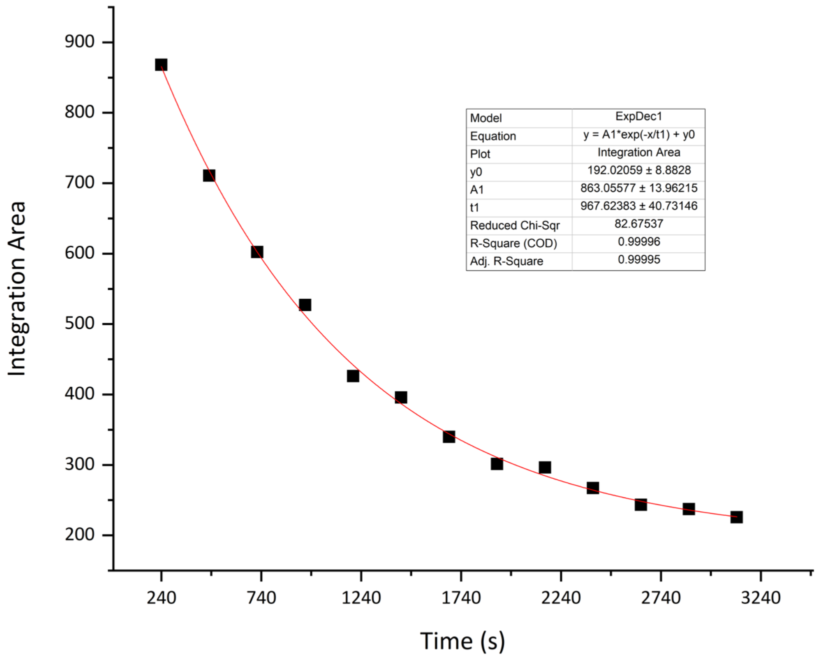

Figure 97. Thermal decay of **8** in DMSO at 90 °C after reaching the photo stationary state (λ_{ex} = 526 nm). ^{1}HNMR spectra were collected every 240 seconds until (almost) full conversion to the trans isomer was observed.

Eyring plot for compound 8

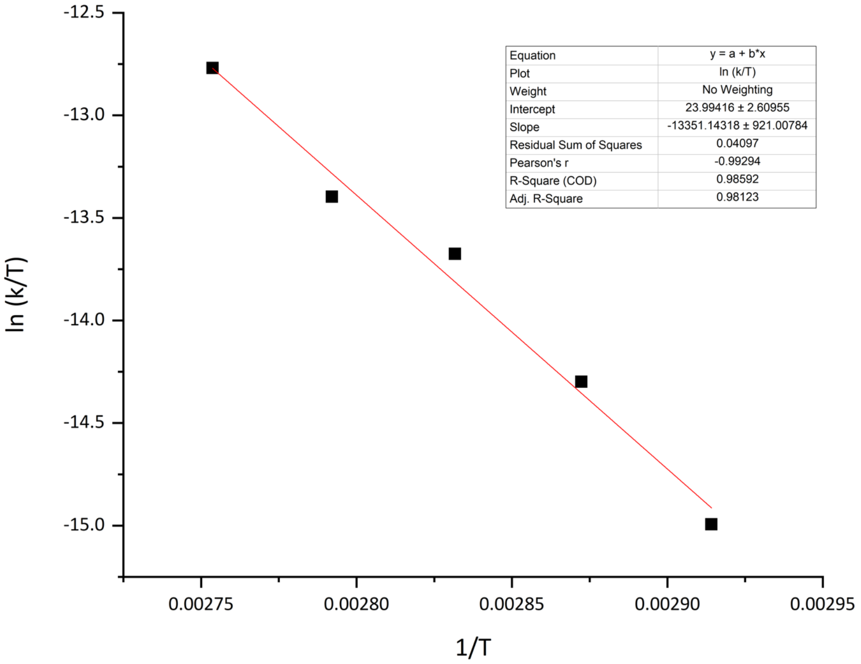

Figure 98. Eyring plot of ln (k/T) vs 1/T for compound **8**. Adjusted R^2 = 0.98.

Thermal decay plots for compound 9

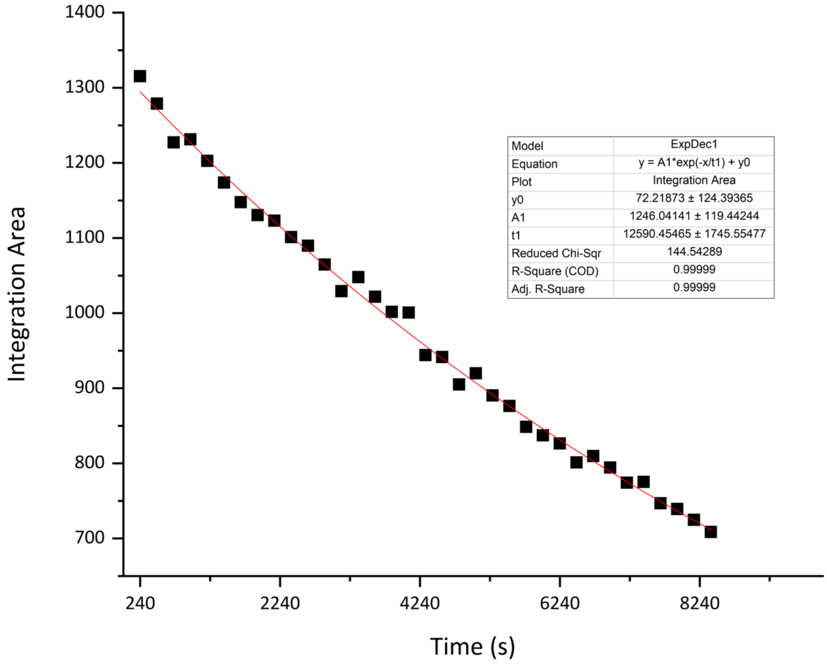

Figure 99. Thermal decay of **9** in DMSO at 50 °C after reaching the photo stationary state (λ_{ex} = 526 nm). ^{1}HNMR spectra were collected every 240 seconds until (almost) full conversion to the trans isomer was observed.

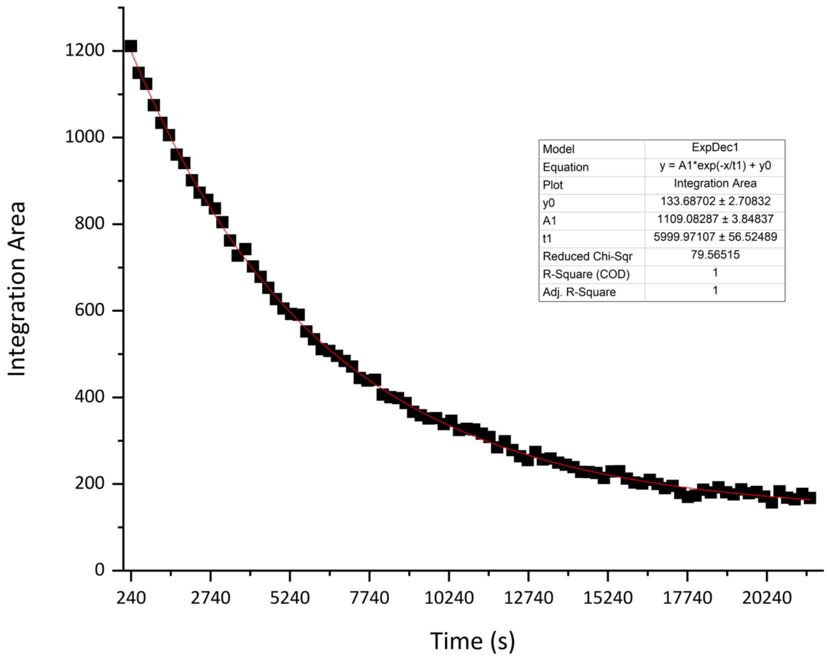

Figure 100. Thermal decay of **9** in DMSO at 55 °C after reaching the photo stationary state (λ_{ex} = 526 nm). ^{1}HNMR spectra were collected every 240 seconds until (almost) full conversion to the trans isomer was observed.

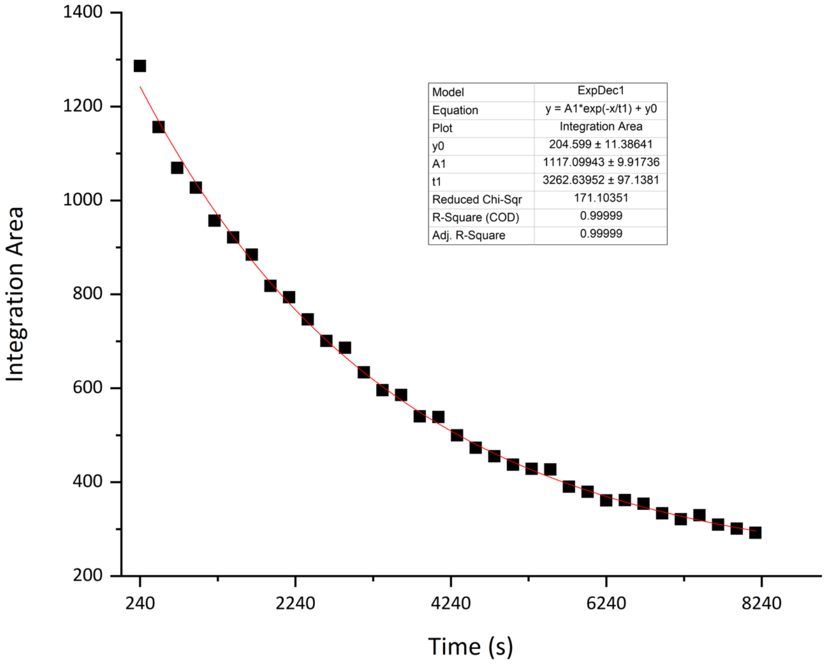

Figure 101. Thermal decay of **9** in DMSO at 60 °C after reaching the photo stationary state (λ_{ex} = 526 nm). ^{1}HNMR spectra were collected every 240 seconds until (almost) full conversion to the trans isomer was observed.

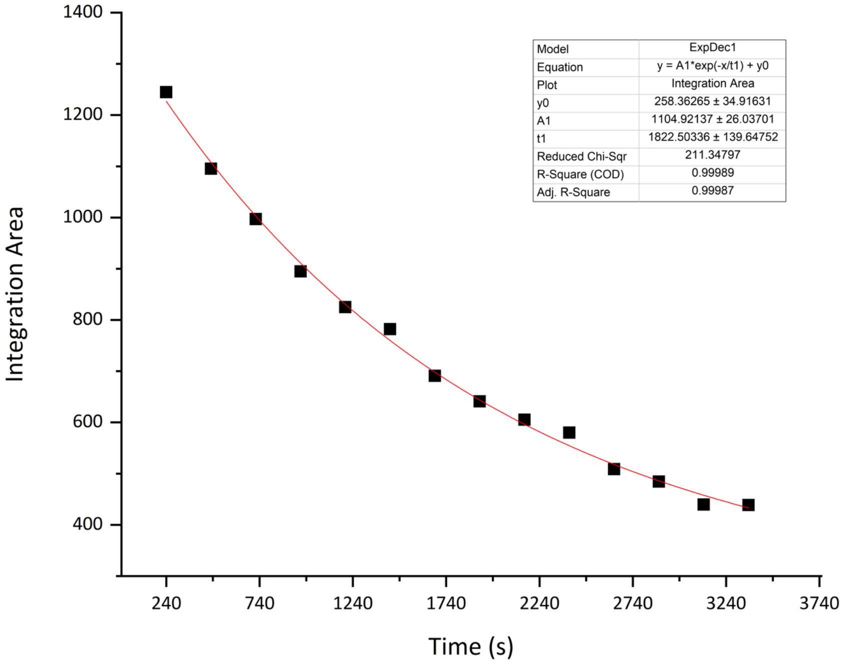

Figure 102. Thermal decay of **9** in DMSO at 65 °C after reaching the photo stationary state (λ_{ex} = 526 nm). ^{1}HNMR spectra were collected every 240 seconds until (almost) full conversion to the trans isomer was observed.

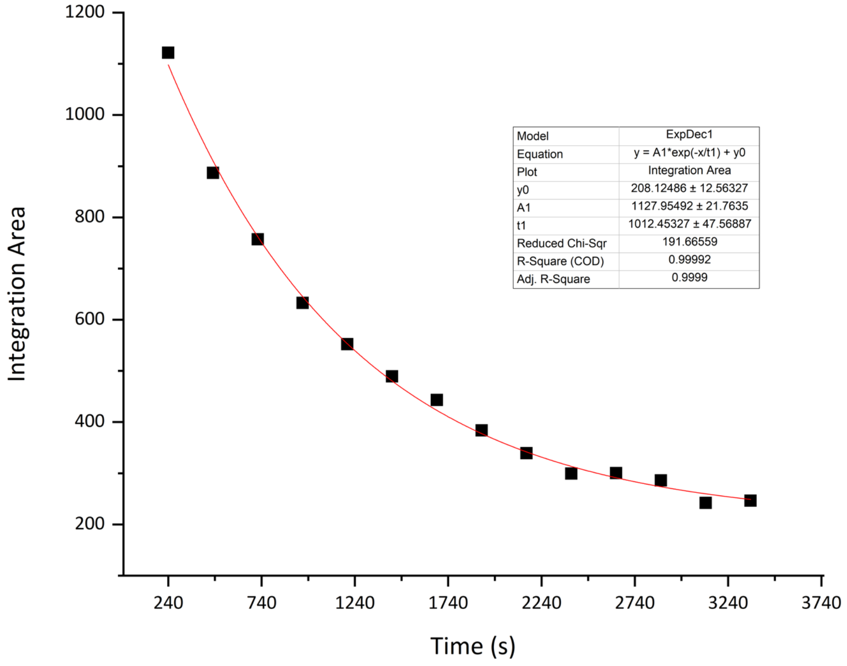

Figure 103. Thermal decay of **9** in DMSO at 70 °C after reaching the photo stationary state (λ_{ex} = 526 nm). ^{1}HNMR spectra were collected every 240 seconds until (almost) full conversion to the trans isomer was observed.

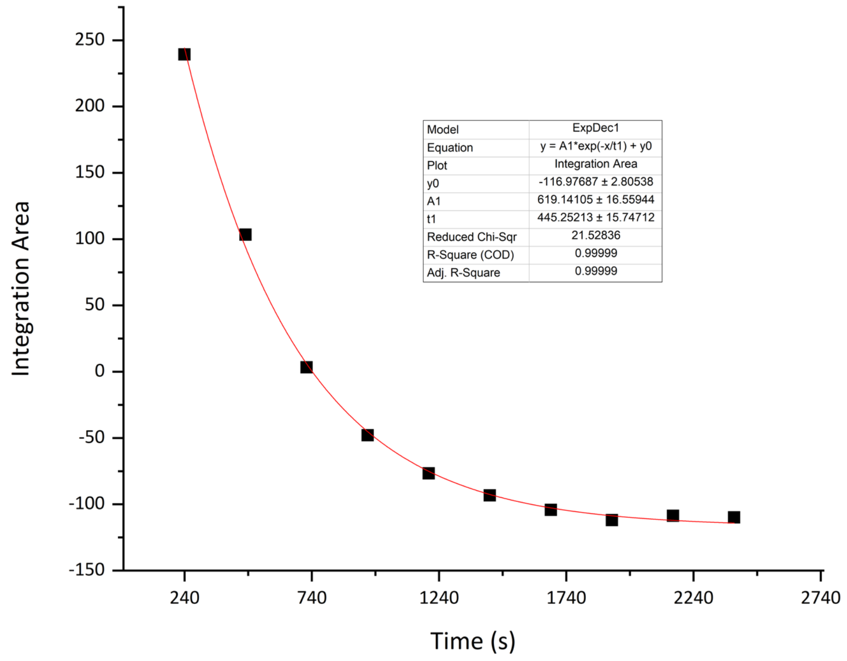

Figure 104. Thermal decay of **9** in DMSO at 75 °C after reaching the photo stationary state (λ_{ex} = 526 nm). ^{1}HNMR spectra were collected every 240 seconds until (almost) full conversion to the trans isomer was observed.

Eyring plot for compound 9

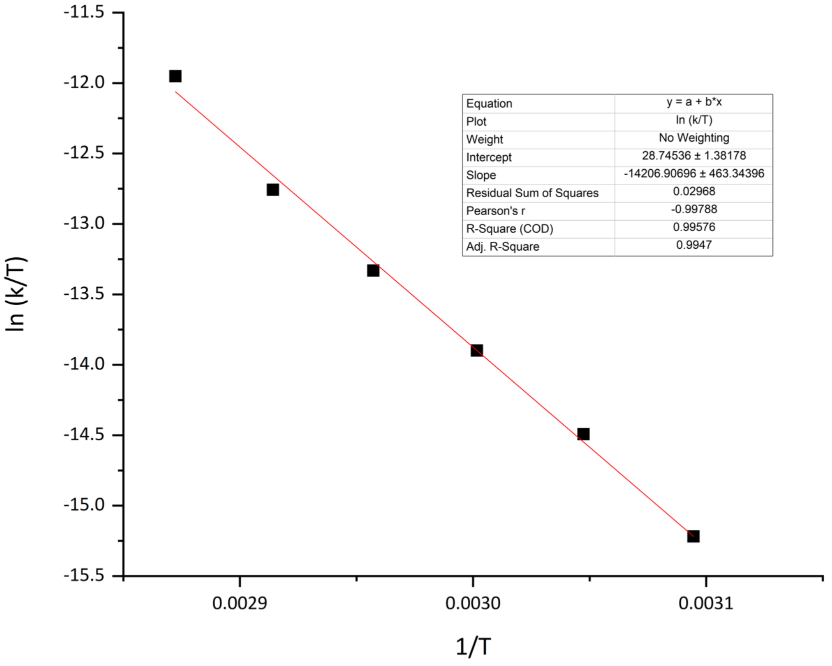

Figure 105. Eyring plot of ln (k/T) vs 1/T for compound **9**. Adjusted R^2 = 0.99.

Thermal decay plots for compound 10

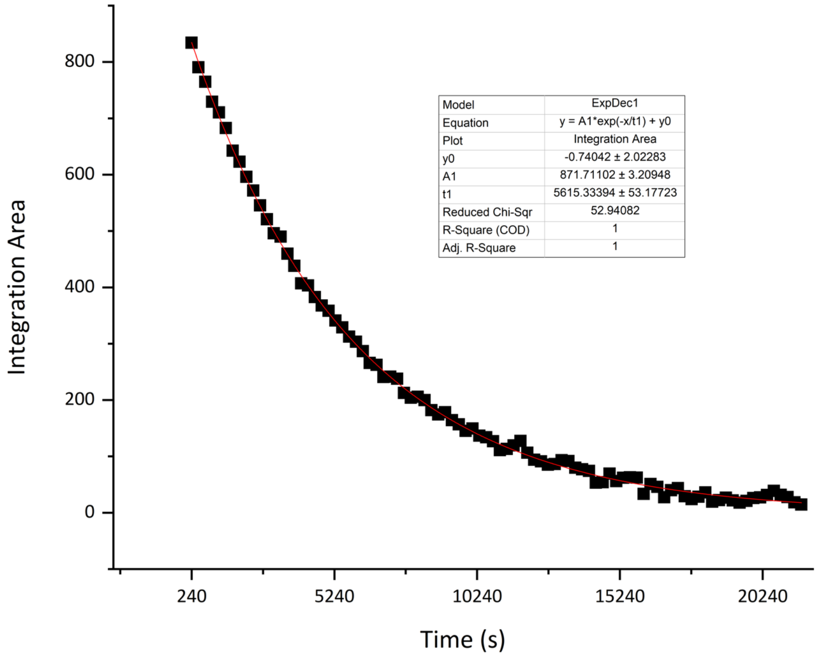

Figure 106. Thermal decay of **10** in DMSO at 25 °C after reaching the photo stationary state (λ_{ex} = 526 nm). ^{1}HNMR spectra were collected every 240 seconds until (almost) full conversion to the trans isomer was observed.

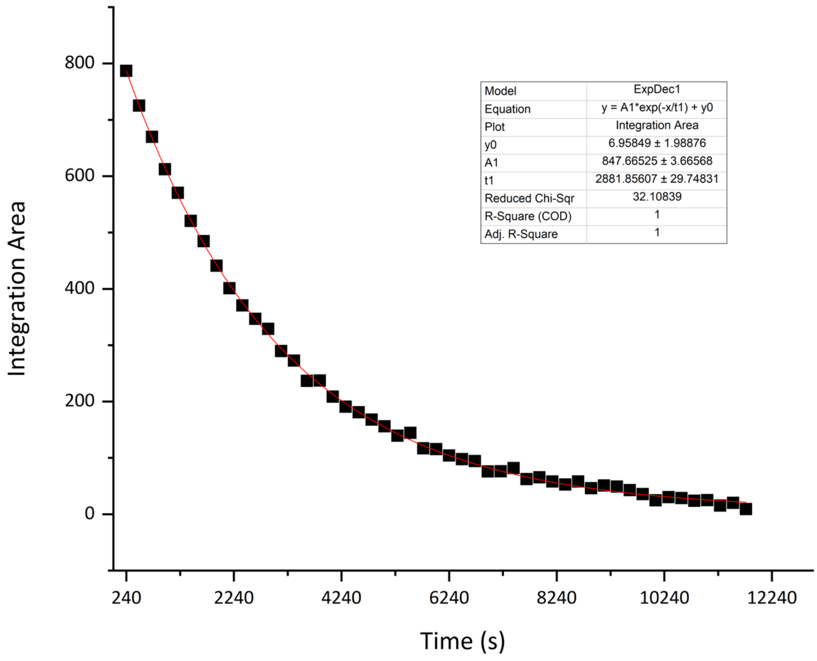

Figure 107. Thermal decay of **10** in DMSO at 30 °C after reaching the photo stationary state (λ_{ex} = 526 nm). ^{1}HNMR spectra were collected every 240 seconds until (almost) full conversion to the trans isomer was observed.

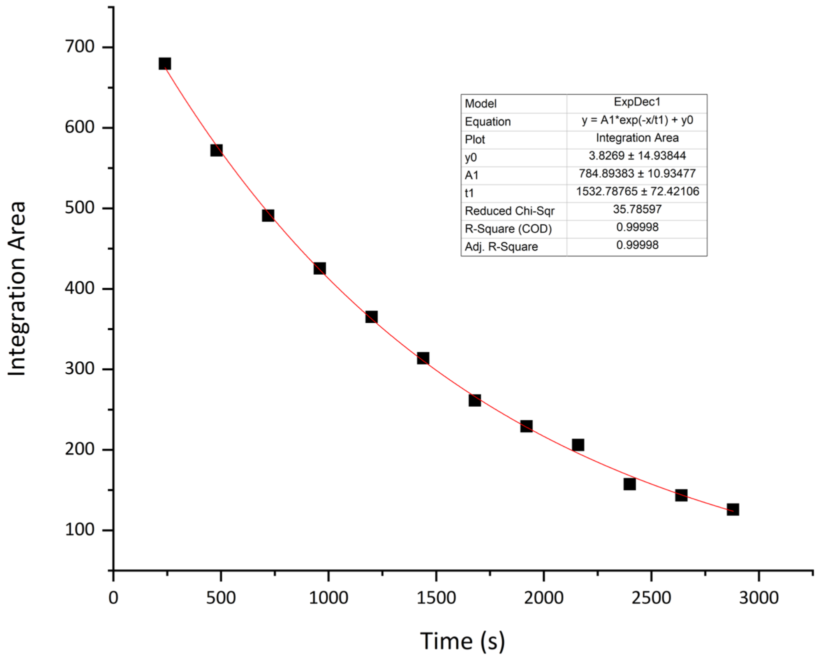

Figure 108. Thermal decay of **10** in DMSO at 35 °C after reaching the photo stationary state (λ_{ex} = 526 nm). ^{1}HNMR spectra were collected every 240 seconds until (almost) full conversion to the trans isomer was observed.

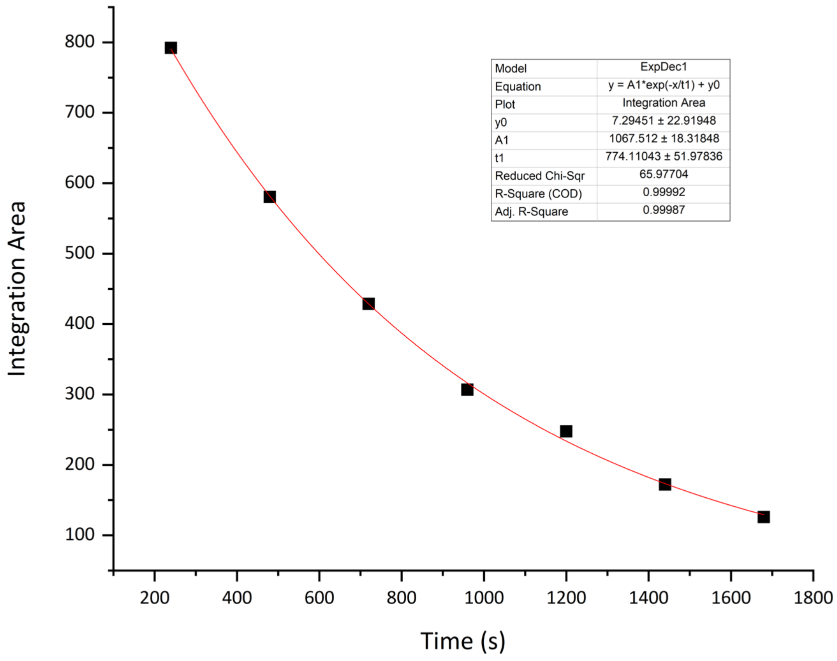

Figure 109. Thermal decay of **10** in DMSO at 40 °C after reaching the photo stationary state (λ_{ex} = 526 nm). ^{1}HNMR spectra were collected every 240 seconds until (almost) full conversion to the trans isomer was observed.

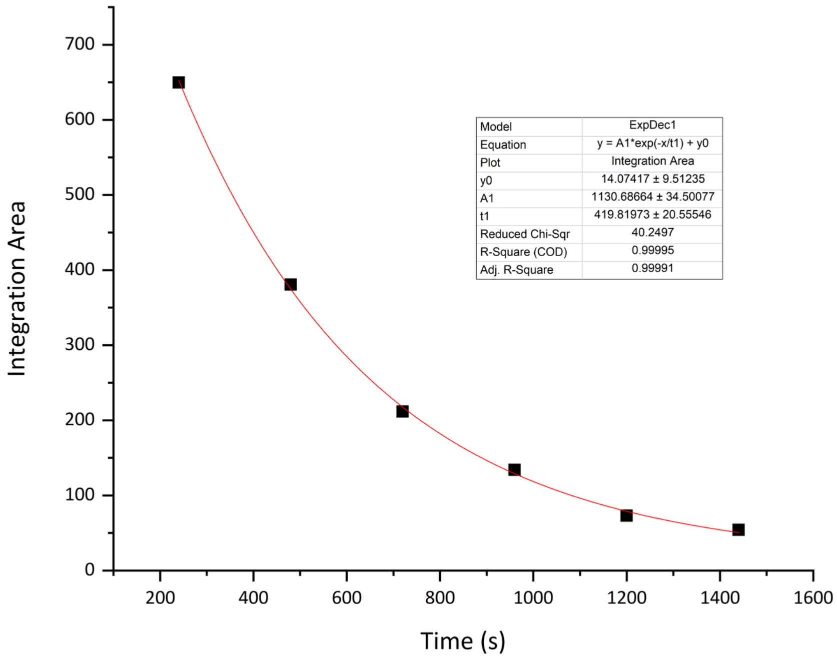

Figure 110. Thermal decay of **10** in DMSO at 45 °C after reaching the photo stationary state (λ_{ex} = 526 nm). ^{1}HNMR spectra were collected every 240 seconds until (almost) full conversion to the trans isomer was observed.

Eyring plot for compound 10

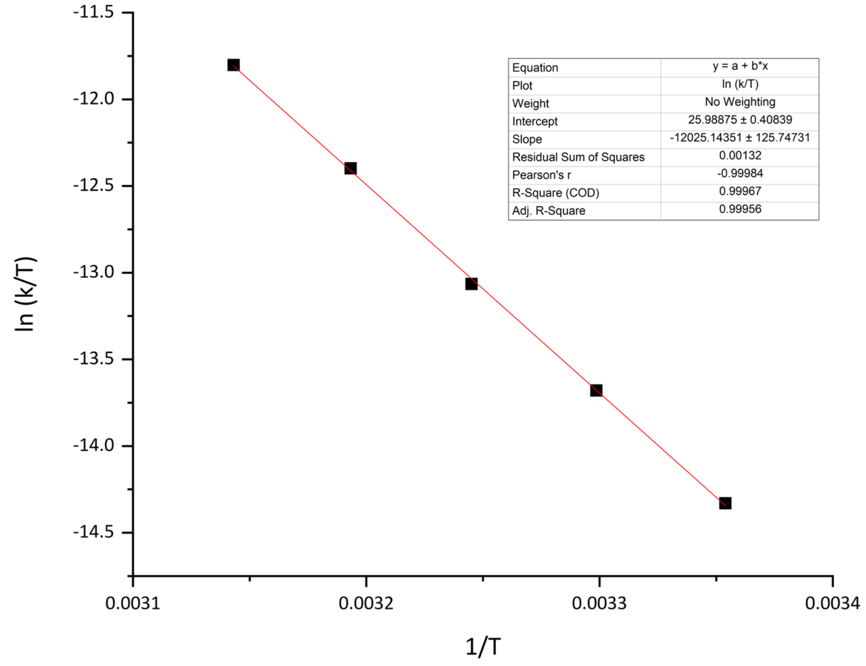

Figure 111. Eyring plot of ln (k/T) vs 1/T for compound **10**. Adjusted R^2 = 1.00.

Quantum Yield calculation

UV/vis evolution spectra were recorded using the setup described above with the 535R LED and the Chroma Narrow GreenMV532/20 filter (FWHM = 20 nm), the 420Z LED (FWHM = 14 nm), or 445B LED (FWHM = 18 nm). The photon flux was determined using standard ferrioxalate actinometry, which provided a value of of 5.76 · 10^{-6} mol · photons s^{-1} for the 535R LED and the Chroma Narrow GreenMV532/20 filter, 3.26 10^{-5} mol · photons s^{-1} for the 420Z LED, and 2.60 10^{-5} mol · photons s^{-1} for the 445B LED. Spectra were collected over 1000 seconds, exported and processed in SpectraGryph and OriginPro. Baseline corrections were carried out to correct for baseline drifting, after which the data was processed in QYMain (https://www.nature.com/articles/srep41145#Sec14) developed by Stranius & Börjesson.[1] Eq. 7 (d[A]/dt=I*QY(A→B)*beta/(Na*V)+I*QY(B→A)*betaB/(Na*V)+[A]*k(B→A) was used to the fit the data collected in the first 50 – 500 seconds using the following molar extinction coefficients and absorbance data at the irradiation wavelength (532 nm or 445 nm, respectively):

Compound	445 nm		532 nm	
	**ε *trans*** **(M^{-1} cm^{-1})**	**ε *cis*** **(M^{-1} cm^{-1})**	**ε *trans*** **(M^{-1} cm^{-1})**	**ε *cis*** **(M^{-1} cm^{-1})**
**1**	8775[a]	3903[a]	1715	2564
**2**	1022	1318	353	239
**3**	753	1568	243	89
**4**	811	1556	222	104
**5**	1489	2520	636	238
**6**	829	1535	243	68
**7**	1323	1577	312	151
**8**	573	1204	176	31
**9**	617	1288	199	59
**10**	768	1165	451	280

[a]For compound **1** the 420 nm LED of the same setup and ε(*trans*)=14796 and ε(*cis*)=2500 was used.

Absorbance at 532 nm for compound **1**

Time (s)	Absorbance
0	0.08014393
1	0.08056974
2	0.08046484
3	0.08035707
4	0.08124828
5	0.08107805
6	0.08059358
7	0.08184767
8	0.0809083
9	0.08169412
10	0.08250427
11	0.0823536
12	0.08314037
13	0.0830369
14	0.08298349
15	0.08419418
16	0.08372784
17	0.08359671
18	0.08396768
19	0.08395338
20	0.08405399
21	0.08445024
22	0.0843029
23	0.0842185
24	0.08539295
25	0.08506393
26	0.0846138
27	0.08505058
28	0.08581925
29	0.08589983
30	0.08587455
31	0.08515978
32	0.08515119
33	0.08522605
34	0.0855608
35	0.08582258
36	0.0859828
37	0.08617973
38	0.08638096
39	0.08660888
40	0.08618259
41	0.08634377
42	0.08626937
43	0.08623314
44	0.08641863
45	0.08662605
46	0.0870533
47	0.08744049
48	0.08763361
49	0.08761167
50	0.08728743
51	0.08725023
52	0.08764314
53	0.08821726
54	0.08789157
55	0.08772707
56	0.08800935
57	0.08837462
58	0.08805466
59	0.08809519
60	0.08826542
61	0.0883689
62	0.08808613
63	0.0885377

64	0.08856488
65	0.08862495
66	0.0884242
67	0.08876705
68	0.08873892
69	0.08865261
70	0.08896017
71	0.08887625
72	0.0890913
73	0.08907986
74	0.08910846
75	0.08924342
76	0.08918381
77	0.08935452
78	0.08934116
79	0.08906745
80	0.08916331
81	0.08911562
82	0.08939791
83	0.08913231
84	0.0896535
85	0.08956957
86	0.08949805
87	0.08979559
88	0.08959055
89	0.0895257
90	0.08975553
91	0.0895958
92	0.08989954
93	0.08986712
94	0.08971405
95	0.09000826
96	0.09011697
97	0.08977651
98	0.09011364
99	0.09019327
100	0.0902648

Absorbance at 420 nm for compound **1**

Time (s)	Absorbance
0	0.46009
0.5	0.44525
1	0.43185
1.5	0.41951
2	0.40744
2.5	0.39661
3	0.38675
3.5	0.37645
4	0.36667
4.5	0.35776
5	0.34934
5.5	0.34195
6	0.33356
6.5	0.32572
7	0.31947
7.5	0.31284
8	0.30601
8.5	0.29925
9	0.29242
9.5	0.28614
10	0.28014
10.5	0.27453
11	0.26973
11.5	0.26539
12	0.26125
12.5	0.25699
13	0.25319
13.5	0.24899

14	0.24544
14.5	0.24155
15	0.23799
15.5	0.23461
16	0.23159
16.5	0.22826
17	0.22547
17.5	0.22285
18	0.22031
18.5	0.21824
19	0.21579
19.5	0.21398
20	0.21128
20.5	0.20943
21	0.20764
21.5	0.20606
22	0.20425
22.5	0.20266
23	0.20109
23.5	0.19893
24	0.19789
24.5	0.19678
25	0.19527
25.5	0.19385
26	0.19258
26.5	0.19141
27	0.18942
27.5	0.1888
28	0.18816
28.5	0.18661
29	0.18597
29.5	0.18508
30	0.18472
30.5	0.18391
31	0.18327
31.5	0.1827
32	0.1816
32.5	0.18099
33	0.1804
33.5	0.17989
34	0.17939
34.5	0.17862
35	0.17815
35.5	0.17749
36	0.17622
36.5	0.17631
37	0.176
37.5	0.17567
38	0.17535
38.5	0.17509
39	0.17461
39.5	0.17447
40	0.17393
40.5	0.17359
41	0.17318
41.5	0.17346
42	0.17289
42.5	0.17265
43	0.17245
43.5	0.17201
44	0.17193
44.5	0.1715
45	0.17148
45.5	0.17167
46	0.17109
46.5	0.17098
47	0.17022
47.5	0.17007

48	0.17018
48.5	0.16995
49	0.16965
49.5	0.1703
50	0.16979
50.5	0.1697
51	0.16939
51.5	0.16899
52	0.16919
52.5	0.16917
53	0.1688
53.5	0.16915
54	0.16925
54.5	0.16826
55	0.16851
55.5	0.16855
56	0.16847

Absorbance at 532 nm for compound **2**

Time (s)	Absorbance
0	0.0712905
1	0.07138252
2	0.07124663
3	0.07119607
4	0.07107258
5	0.07097005
6	0.0709095
7	0.07108879
8	0.0708437
9	0.07119226
10	0.07115126
11	0.07111931
12	0.0710764
13	0.07108831
14	0.07100964
15	0.0710268
16	0.07117319
17	0.07098627
18	0.07079029
19	0.07088136
20	0.07081556
21	0.07079077
22	0.07067061
23	0.07082414
24	0.0705638
25	0.070714
26	0.07077407
27	0.07065249
28	0.07055426
29	0.07055568
30	0.07057143
31	0.07050848
32	0.07025481
33	0.07048702
34	0.07050896
35	0.07039928
36	0.07026958
37	0.07038164
38	0.07034779
39	0.0703206
40	0.07046557
41	0.07014561
42	0.07024384
43	0.07000875
44	0.07010412
45	0.07026195
46	0.0702405
47	0.07017947

48	0.0701766
49	0.06992054
50	0.0700016
51	0.06968689
52	0.06983471
53	0.06970072
54	0.06982613
55	0.06982422
56	0.06983328
57	0.06952334
58	0.06944847
59	0.06947946
60	0.06950807
61	0.06956434
62	0.06955194
63	0.06937123
64	0.06935072
65	0.06925345
66	0.06936121
67	0.06929254
68	0.06920195
69	0.06932259
70	0.06924057
71	0.06930066
72	0.0690465
73	0.06912184
74	0.06894112
75	0.0690732
76	0.0689907
77	0.06897449
78	0.06908322
79	0.06885291
80	0.06884289
81	0.06876851
82	0.06869746
83	0.06882572
84	0.06871176
85	0.0687604
86	0.06879473
87	0.0684948
88	0.06847286
89	0.06862354
90	0.0686655
91	0.06830692
92	0.06830024
93	0.0682826
94	0.06827021
95	0.06835461
96	0.06818008
97	0.06833219
98	0.068089
99	0.0682106
100	0.06832981

Absorbance at 445 nm for compound **2**

Time (s)	Absorbance
0	0.03168
0.5	0.0317
1	0.03152
1.5	0.03158
2	0.032
2.5	0.03158
3	0.03187
3.5	0.03205
4	0.03221
4.5	0.03223
5	0.03198
5.5	0.03209

6	0.03238
6.5	0.0321
7	0.03229
7.5	0.03203
8	0.03218
8.5	0.03221
9	0.03218
9.5	0.03241
10	0.03199
10.5	0.03258
11	0.03215
11.5	0.03229
12	0.03238
12.5	0.03242
13	0.03266
13.5	0.03254
14	0.03236
14.5	0.03255
15	0.03301
15.5	0.03309
16	0.03337
16.5	0.03323
17	0.03289
17.5	0.03279
18	0.03289
18.5	0.03315
19	0.03271
19.5	0.03302
20	0.03293
20.5	0.03314
21	0.03299
21.5	0.03281
22	0.03326
22.5	0.03313
23	0.03311
23.5	0.03328
24	0.03294
24.5	0.03294
25	0.03326
25.5	0.03316
26	0.03338
26.5	0.03322
27	0.03294
27.5	0.03352
28	0.03311
28.5	0.0333
29	0.03342
29.5	0.03347
30	0.03336
30.5	0.0336
31	0.03352
31.5	0.03383
32	0.03332
32.5	0.03363
33	0.03343
33.5	0.03371
34	0.03344
34.5	0.03348
35	0.03345
35.5	0.03344
36	0.03377
36.5	0.03358
37	0.03373
37.5	0.03411
38	0.03366
38.5	0.0339
39	0.03369
39.5	0.03363

40	0.03375
40.5	0.03379
41	0.03361
41.5	0.03376
42	0.03375
42.5	0.03384
43	0.03385
43.5	0.03411
44	0.03391
44.5	0.03403
45	0.03391
45.5	0.03405
46	0.03375
46.5	0.03393
47	0.03389
47.5	0.03386
48	0.03412
48.5	0.0339
49	0.03388
49.5	0.0344
50	0.03428
50.5	0.03383
51	0.03426
51.5	0.03427
52	0.03415
52.5	0.03411
53	0.03454
53.5	0.03414
54	0.03406
54.5	0.03399
55	0.03418
55.5	0.03441
56	0.03423
56.5	0.03413
57	0.03437
57.5	0.03398
58	0.03425
58.5	0.03409
59	0.03414
59.5	0.03425
60	0.03383
60.5	0.03399
61	0.03411
61.5	0.03427
62	0.03442
62.5	0.03454
63	0.03423
63.5	0.03415
64	0.03406
64.5	0.03428
65	0.03452
65.5	0.03424
66	0.03411
66.5	0.03445
67	0.0344
67.5	0.03443
68	0.03442
68.5	0.03465
69	0.03437
69.5	0.03421
70	0.0343
70.5	0.03397
71	0.03397
71.5	0.03429
72	0.03413
72.5	0.03431
73	0.03468
73.5	0.03473

74	0.03431
74.5	0.03452
75	0.03458
75.5	0.0341
76	0.03411
76.5	0.0344
77	0.03446
77.5	0.03424
78	0.03451
78.5	0.03459
79	0.03426
79.5	0.03401
80	0.03433
80.5	0.03396
81	0.03445
81.5	0.03458
82	0.03415
82.5	0.03406
83	0.03438
83.5	0.03453
84	0.03458
84.5	0.03414
85	0.03435
85.5	0.03451
86	0.03467
86.5	0.03464
87	0.0343
87.5	0.0346
88	0.03448
88.5	0.03495
89	0.03477
89.5	0.03453
90	0.0349
90.5	0.03486
91	0.03477
91.5	0.03463
92	0.03465
92.5	0.03489
93	0.03513
93.5	0.03491
94	0.03481
94.5	0.03459
95	0.03464
95.5	0.03468
96	0.03426
96.5	0.03469
97	0.03476
97.5	0.03466
98	0.03468
98.5	0.03454
99	0.03459
99.5	0.03475
100	0.03446
100.5	0.03455
101	0.03448
101.5	0.03474
102	0.0348
102.5	0.0349
103	0.0348
103.5	0.03462
104	0.03434
104.5	0.03466
105	0.03438
105.5	0.03455
106	0.03454
106.5	0.03464
107	0.03465
107.5	0.03463

108	0.03449
108.5	0.03495
109	0.03501
109.5	0.03462
110	0.03452
110.5	0.03498
111	0.03469
111.5	0.03454
112	0.03514
112.5	0.03469
113	0.03452
113.5	0.0346
114	0.03465
114.5	0.03502
115	0.03445
115.5	0.03476
116	0.03472
116.5	0.03471
117	0.03453
117.5	0.03454
118	0.0342
118.5	0.03455
119	0.03427
119.5	0.03484
120	0.03489
120.5	0.03484
121	0.03483
121.5	0.03483
122	0.03493
122.5	0.03488
123	0.03457
123.5	0.03465
124	0.0347
124.5	0.03459
125	0.03449
125.5	0.03483
126	0.03459
126.5	0.03493
127	0.03482
127.5	0.03465
128	0.03465
128.5	0.0349
129	0.03501
129.5	0.03495
130	0.035
130.5	0.03479
131	0.03483
131.5	0.03505
132	0.03521
132.5	0.03516
133	0.03468
133.5	0.03476
134	0.0348
134.5	0.03456
135	0.03477
135.5	0.03472
136	0.03466
136.5	0.03487
137	0.03501
137.5	0.03465
138	0.0347
138.5	0.03456
139	0.03474
139.5	0.03495
140	0.03453
140.5	0.03463
141	0.03478
141.5	0.03468

142	0.03472
142.5	0.03446
143	0.03457
143.5	0.03475
144	0.03508
144.5	0.03468
145	0.03464
145.5	0.03477
146	0.03471
146.5	0.03451
147	0.0344
147.5	0.03447
148	0.03451
148.5	0.03469
149	0.03472
149.5	0.0346
150	0.03467
150.5	0.0344
151	0.03448
151.5	0.03458
152	0.03467
152.5	0.03513
153	0.03474
153.5	0.03463
154	0.03445
154.5	0.03463
155	0.0345
155.5	0.03485
156	0.03471
156.5	0.03472
157	0.03436
157.5	0.03467
158	0.03487
158.5	0.03479
159	0.03459
159.5	0.03438
160	0.03457
160.5	0.03473
161	0.03486
161.5	0.03447
162	0.03453
162.5	0.03449
163	0.0346
163.5	0.03478
164	0.03444
164.5	0.03462
165	0.0346
165.5	0.03436
166	0.03436
166.5	0.03443
167	0.03448
167.5	0.03431
168	0.03439
168.5	0.03461
169	0.03461
169.5	0.03465
170	0.03488
170.5	0.03482
171	0.03454
171.5	0.03441
172	0.0347
172.5	0.03513
173	0.03468
173.5	0.03465
174	0.03474
174.5	0.03425
175	0.03471
175.5	0.03482

176	0.03468
176.5	0.03485
177	0.03515
177.5	0.03489
178	0.03465
178.5	0.03517
179	0.03505
179.5	0.0348
180	0.03483
180.5	0.03474
181	0.0345
181.5	0.03461
182	0.03489
182.5	0.03484
183	0.03447
183.5	0.03457
184	0.03465
184.5	0.03476
185	0.03456
185.5	0.0346
186	0.03475
186.5	0.03447
187	0.03418
187.5	0.03418
188	0.03428
188.5	0.03473
189	0.03495
189.5	0.03448
190	0.03473
190.5	0.03444
191	0.03438
191.5	0.0345
192	0.03456
192.5	0.03494
193	0.0349
193.5	0.03445
194	0.03436
194.5	0.03469
195	0.03458
195.5	0.03467
196	0.03464
196.5	0.03458
197	0.03465
197.5	0.03455
198	0.03461
198.5	0.03457
199	0.03479

Absorbance at 532 nm for compound **3**

Time (s)	Absorbance
0	0.04625082
1	0.04623413
2	0.0462656
3	0.04666853
4	0.04698181
5	0.04663468
6	0.04646539
7	0.04598904
8	0.04590988
9	0.04561138
10	0.04534197
11	0.04552031
12	0.04564094
13	0.04554414
14	0.04542256
15	0.04556989
16	0.04553127
17	0.04589844

18	0.04592657
19	0.04586649
20	0.04591751
21	0.04592228
22	0.0455656
23	0.04481363
24	0.046094413
25	0.046550749
26	0.04699278
27	0.046942231
28	0.04654407
29	0.046245573
30	0.046430591
31	0.045267579
32	0.046497817
33	0.046577933
34	0.04676533
35	0.045854568
36	0.04580641
37	0.0455122
38	0.045975209
39	0.045809746
40	0.045721536
41	0.04571772
42	0.04595756
43	0.046052456
44	0.045938969
45	0.04544019
46	0.04590226
47	0.045698164
48	0.045753482
49	0.045574193
50	0.045563695
51	0.046092988
52	0.046018604
53	0.045792106
54	0.045789247
55	0.04537678
56	0.04509306
57	0.045009609
58	0.045332904
59	0.045413975
60	0.04576826
61	0.045486452
62	0.045498845
63	0.045202255
64	0.045255184
65	0.045462133
66	0.045372968
67	0.045553212
68	0.045567515
69	0.045296666
70	0.045409676
71	0.045446873
72	0.045325275
73	0.045520305
74	0.04553795
75	0.044834615
76	0.044733045
77	0.044924737
78	0.044889928
79	0.045127867
80	0.045032497
81	0.045125483
82	0.045283321
83	0.045122149
84	0.044983861
85	0.04510307

86	0.04496956
87	0.044899942
88	0.044867519
89	0.044865128
90	0.044852729
91	0.044990067
92	0.044726368
93	0.04484034
94	0.045317654
95	0.044740204
96	0.045203688
97	0.045345302
98	0.045309063
99	0.045156479
100	0.04524803

Absorbance at 445 nm for compound **3**

Time (s)	Absorbance
0	0.01755
0.5	0.01824
1	0.01843
1.5	0.01818
2	0.01854
2.5	0.01907
3	0.01829
3.5	0.01869
4	0.01874
4.5	0.01838
5	0.01857
5.5	0.01912
6	0.01939
6.5	0.01946
7	0.01956
7.5	0.02021
8	0.01975
8.5	0.0191
9	0.01916
9.5	0.0195
10	0.0202
10.5	0.01991
11	0.02021
11.5	0.02037
12	0.02033
12.5	0.02093
13	0.02119
13.5	0.02134
14	0.02121
14.5	0.02185
15	0.02166
15.5	0.02164
16	0.0216
16.5	0.02125
17	0.02108
17.5	0.02186
18	0.0216
18.5	0.02185
19	0.0216
19.5	0.02204
20	0.02178
20.5	0.02238
21	0.02243
21.5	0.0224
22	0.02164
22.5	0.02162
23	0.02265
23.5	0.02214
24	0.0227
24.5	0.02174

25	0.02235
25.5	0.02217
26	0.02217
26.5	0.02242
27	0.02244
27.5	0.02226
28	0.02201
28.5	0.02231
29	0.02287
29.5	0.02271
30	0.02316
30.5	0.02288
31	0.0227
31.5	0.02303
32	0.02298
32.5	0.02283
33	0.0231
33.5	0.02324
34	0.02303
34.5	0.02313
35	0.0231
35.5	0.02301
36	0.02338
36.5	0.02298
37	0.02314
37.5	0.02324
38	0.02328
38.5	0.02317
39	0.02359
39.5	0.02343
40	0.02357
40.5	0.0236
41	0.02376
41.5	0.02336
42	0.02343
42.5	0.02337
43	0.02383
43.5	0.02372
44	0.0238
44.5	0.0232
45	0.02396
45.5	0.02398
46	0.02382
46.5	0.02384
47	0.02381
47.5	0.02388
48	0.02387
48.5	0.02397
49	0.02414
49.5	0.02441
50	0.02433

Absorbance at 532 nm for compound **4**

Time (s)	Absorbance
0	0.03371191
1	0.03353595
2	0.03370476
3	0.03370905
4	0.03347873
5	0.03344965
6	0.03351307
7	0.03352404
8	0.03347015
9	0.03361702
10	0.03353786
11	0.0333929
12	0.03345013
13	0.03343725

14	0.03346444
15	0.0333848
16	0.03339338
17	0.03351927
18	0.03332901
19	0.03343582
20	0.03343201
21	0.03342199
22	0.03334856
23	0.03333997
24	0.03317404
25	0.03338575
26	0.03341293
27	0.03331661
28	0.03341246
29	0.03333283
30	0.0332241
31	0.03321743
32	0.03324032
33	0.03326416
34	0.0330615
35	0.03308678
36	0.03314162
37	0.0332756
38	0.03305102
39	0.03327942
40	0.03321361
41	0.03313732
42	0.03308773
43	0.03322553
44	0.03302765
45	0.0331769
46	0.03300142
47	0.03314066
48	0.0330348
49	0.03321599
50	0.03307199
51	0.03308868
52	0.03310919
53	0.03286266
54	0.03300857
55	0.03300905
56	0.03306579
57	0.03307105
58	0.03280306
59	0.03294611
60	0.03293276
61	0.03297806
62	0.03299284
63	0.0328989
64	0.03303146
65	0.03287459
66	0.0329895
67	0.0328064
68	0.03287267
69	0.03272915
70	0.03291369
71	0.03283167
72	0.03281308
73	0.03291035
74	0.03276157
75	0.03260517
76	0.03282309
77	0.03268767
78	0.03288841
79	0.03290987
80	0.03274632
81	0.03292275

82	0.03266811
83	0.03264856
84	0.03268718
85	0.03273392
86	0.03253126
87	0.03264857
88	0.03262663
89	0.0325737
90	0.03269052
91	0.03250075
92	0.03262282
93	0.03262329
94	0.0326891
95	0.03249741
96	0.03249359
97	0.0325656
98	0.03248024
99	0.03249884
100	0.03244114

Absorbance at 445 nm for compound **4**

Time (s)	Absorbance
0	0.04487
0.5	0.04493
1	0.04476
1.5	0.04402
2	0.04505
2.5	0.04473
3	0.04477
3.5	0.04392
4	0.04499
4.5	0.04454
5	0.04444
5.5	0.04428
6	0.04414
6.5	0.04489
7	0.04575
7.5	0.04567
8	0.04514
8.5	0.0456
9	0.04463
9.5	0.04459
10	0.04437
10.5	0.04507
11	0.04472
11.5	0.04537
12	0.04525
12.5	0.04546
13	0.04505
13.5	0.04577
14	0.04515
14.5	0.04511
15	0.0443
15.5	0.04493
16	0.04495
16.5	0.04454
17	0.04477
17.5	0.04425
18	0.04502
18.5	0.04561
19	0.04515
19.5	0.04552
20	0.0456
20.5	0.0455
21	0.04547
21.5	0.04542
22	0.04553

22.5	0.04489
23	0.04516
23.5	0.04538
24	0.04549
24.5	0.04471
25	0.04564
25.5	0.04545
26	0.04565
26.5	0.04573
27	0.0456
27.5	0.04506
28	0.04584
28.5	0.04577
29	0.04546
29.5	0.04533
30	0.04547
30.5	0.04534
31	0.04473
31.5	0.04526
32	0.04546
32.5	0.04499
33	0.0449
33.5	0.04539
34	0.04541
34.5	0.04527
35	0.04503
35.5	0.04515
36	0.04541
36.5	0.04591
37	0.04491
37.5	0.04479
38	0.04535
38.5	0.04553
39	0.04516
39.5	0.04541
40	0.04473
40.5	0.04534
41	0.04571
41.5	0.04526
42	0.04535
42.5	0.04617
43	0.04569
43.5	0.04541
44	0.04569
44.5	0.0459
45	0.04592
45.5	0.04576
46	0.04543
46.5	0.04557
47	0.04547
47.5	0.04535
48	0.04543
48.5	0.0454
49	0.04561
49.5	0.04466
50	0.04555
50.5	0.0455
51	0.04518
51.5	0.04597
52	0.04587
52.5	0.04572
53	0.04551
53.5	0.04543
54	0.04543
54.5	0.04564
55	0.04531
55.5	0.04498
56	0.04557

56.5	0.04514
57	0.04542
57.5	0.04602
58	0.04524
58.5	0.04488
59	0.04519
59.5	0.04549
60	0.04588
60.5	0.04535
61	0.04542
61.5	0.04607
62	0.04582
62.5	0.04602
63	0.0458
63.5	0.04525
64	0.04556
64.5	0.04491
65	0.04609
65.5	0.0459
66	0.04583
66.5	0.04603
67	0.04543
67.5	0.04538
68	0.04547
68.5	0.04553
69	0.04532
69.5	0.04545
70	0.04516
70.5	0.04523
71	0.04507
71.5	0.04536
72	0.04546
72.5	0.04546
73	0.04573
73.5	0.04499
74	0.04562
74.5	0.04533
75	0.04594
75.5	0.04579
76	0.04569
76.5	0.04534
77	0.04533
77.5	0.04531
78	0.04525
78.5	0.04585
79	0.04562
79.5	0.0454
80	0.04523
80.5	0.04519
81	0.04477
81.5	0.04503
82	0.04521
82.5	0.04536
83	0.04585
83.5	0.0455
84	0.04533
84.5	0.04568
85	0.04596
85.5	0.04625
86	0.04572
86.5	0.04544
87	0.04578
87.5	0.04543
88	0.04553
88.5	0.046
89	0.04604
89.5	0.04536
90	0.04539

90.5	0.04494
91	0.04519
91.5	0.04571
92	0.04553
92.5	0.04515
93	0.04502
93.5	0.04532
94	0.04598
94.5	0.04612
95	0.046
95.5	0.0456
96	0.04541
96.5	0.04578
97	0.04563
97.5	0.04562
98	0.04538
98.5	0.04543
99	0.04577
99.5	0.04567
100	0.04604
100.5	0.04562
101	0.04595
101.5	0.04549
102	0.04545
102.5	0.04622
103	0.04595
103.5	0.04537
104	0.04589
104.5	0.04555
105	0.04581
105.5	0.04584
106	0.04544
106.5	0.04572
107	0.0453
107.5	0.04585
108	0.04632
108.5	0.04546
109	0.04552
109.5	0.0451
110	0.0457
110.5	0.04607
111	0.04584
111.5	0.04559
112	0.04516
112.5	0.04543
113	0.04595
113.5	0.04531
114	0.04554
114.5	0.04594
115	0.0457
115.5	0.046
116	0.04618
116.5	0.0458
117	0.0456
117.5	0.04584
118	0.04612
118.5	0.0461
119	0.04585
119.5	0.04591
120	0.04573
120.5	0.04566
121	0.04575
121.5	0.04528
122	0.04577
122.5	0.04637
123	0.04652
123.5	0.04627
124	0.04602

124.5	0.04591
125	0.04587
125.5	0.0462
126	0.04554
126.5	0.04582
127	0.04592
127.5	0.04577
128	0.04617
128.5	0.04555
129	0.04572
129.5	0.0457
130	0.04594
130.5	0.04517
131	0.04622
131.5	0.0462
132	0.04573
132.5	0.04497
133	0.0458
133.5	0.04629
134	0.04603
134.5	0.04599
135	0.04567
135.5	0.0458
136	0.04567
136.5	0.04566
137	0.04569
137.5	0.0461
138	0.04631
138.5	0.04624
139	0.04607
139.5	0.04601
140	0.04563
140.5	0.04571
141	0.04564
141.5	0.0461
142	0.04594
142.5	0.04675
143	0.04582
143.5	0.04588
144	0.04614
144.5	0.04604
145	0.0461
145.5	0.04581
146	0.04601
146.5	0.04577
147	0.046
147.5	0.04574
148	0.04629
148.5	0.04648
149	0.04581
149.5	0.04575
150	0.04527
150.5	0.04591
151	0.04611
151.5	0.04582
152	0.04577
152.5	0.0462
153	0.04571
153.5	0.04597
154	0.04599
154.5	0.04602
155	0.04582
155.5	0.04593
156	0.04601
156.5	0.04612
157	0.04596
157.5	0.04561
158	0.0453

158.5	0.04596
159	0.04547
159.5	0.04612
160	0.04577
160.5	0.04598
161	0.04587
161.5	0.04559
162	0.04582
162.5	0.04584
163	0.04532
163.5	0.04569
164	0.04608
164.5	0.046
165	0.04667
165.5	0.04638
166	0.04565
166.5	0.04583
167	0.04669
167.5	0.04602
168	0.04606
168.5	0.04591
169	0.04582
169.5	0.04613
170	0.04605
170.5	0.04569
171	0.04593
171.5	0.04544
172	0.04651
172.5	0.04595
173	0.04559
173.5	0.04584
174	0.04552
174.5	0.04592
175	0.0461
175.5	0.0462
176	0.04615
176.5	0.04597
177	0.04573
177.5	0.04569
178	0.04533
178.5	0.04589
179	0.04545
179.5	0.04558
180	0.04553
180.5	0.04585
181	0.0457
181.5	0.04616
182	0.04569
182.5	0.04604
183	0.04584
183.5	0.04534
184	0.04612
184.5	0.04596
185	0.04596
185.5	0.04613
186	0.04608
186.5	0.04601
187	0.04554
187.5	0.04615
188	0.04651
188.5	0.04647
189	0.04646
189.5	0.04524
190	0.04583
190.5	0.04585
191	0.04701
191.5	0.04718
192	0.04644

192.5	0.04678
193	0.04646
193.5	0.04652
194	0.04624
194.5	0.04602
195	0.04673
195.5	0.04584
196	0.04579
196.5	0.04558
197	0.04567
197.5	0.04659
198	0.04552
198.5	0.04516
199	0.04548
199.5	0.0461
200	0.04552
200.5	0.04602
201	0.04582
201.5	0.04589
202	0.04583
202.5	0.04637
203	0.04661
203.5	0.04651
204	0.04609
204.5	0.04602
205	0.0453
205.5	0.04539
206	0.04613
206.5	0.04605
207	0.04539
207.5	0.04585
208	0.04597
208.5	0.04592
209	0.04581
209.5	0.04572
210	0.04631
210.5	0.04605
211	0.04655
211.5	0.04576
212	0.04603
212.5	0.04622
213	0.04607
213.5	0.04576
214	0.04555
214.5	0.04588
215	0.04538
215.5	0.0455
216	0.04521
216.5	0.04573
217	0.04545
217.5	0.04551
218	0.04599
218.5	0.04596
219	0.04568
219.5	0.0459
220	0.04616
220.5	0.04598
221	0.04605
221.5	0.04588
222	0.04647
222.5	0.04576
223	0.04613
223.5	0.04632
224	0.04623
224.5	0.04591
225	0.04561
225.5	0.0462
226	0.04589

226.5	0.04573
227	0.04606
227.5	0.04568
228	0.04591
228.5	0.04546
229	0.0461
229.5	0.04566
230	0.04565
230.5	0.04599
231	0.04539
231.5	0.04574
232	0.04586
232.5	0.04549
233	0.04537
233.5	0.04526
234	0.04541
234.5	0.04532
235	0.04611
235.5	0.04585
236	0.0459
236.5	0.04601
237	0.04584
237.5	0.04622
238	0.04602
238.5	0.04601
239	0.04581
239.5	0.04586
240	0.04566
240.5	0.04594
241	0.04614
241.5	0.04582
242	0.04595
242.5	0.04584
243	0.04596
243.5	0.0458
244	0.04525
244.5	0.04598
245	0.04597
245.5	0.0464
246	0.04626
246.5	0.04626
247	0.04632
247.5	0.04644
248	0.04634
248.5	0.04612
249	0.04591
249.5	0.0456
250	0.0459
250.5	0.04553
251	0.04497
251.5	0.04529
252	0.04543
252.5	0.04543
253	0.04588
253.5	0.04591
254	0.04591
254.5	0.04579
255	0.04572
255.5	0.04611
256	0.0463
256.5	0.04624
257	0.04659
257.5	0.04624
258	0.04651
258.5	0.04617
259	0.04619
259.5	0.04654
260	0.04595

260.5	0.04624
261	0.04616
261.5	0.04572
262	0.04587
262.5	0.04569
263	0.0458
263.5	0.04612
264	0.0462
264.5	0.04607
265	0.04624
265.5	0.04674
266	0.04666
266.5	0.04652
267	0.0463
267.5	0.04554
268	0.04574
268.5	0.0457
269	0.04604
269.5	0.04632
270	0.04613
270.5	0.04594
271	0.0454
271.5	0.04561
272	0.04582
272.5	0.04547
273	0.04513
273.5	0.04589
274	0.04573
274.5	0.04591
275	0.04581
275.5	0.04588
276	0.04566
276.5	0.04568
277	0.04534
277.5	0.04548
278	0.04625
278.5	0.04558
279	0.04602
279.5	0.04614
280	0.04613
280.5	0.04622
281	0.04585
281.5	0.04542
282	0.04591
282.5	0.04601
283	0.04615
283.5	0.04562
284	0.04571
284.5	0.04606
285	0.04593
285.5	0.04576
286	0.04605
286.5	0.04575
287	0.04554
287.5	0.04603
288	0.04538
288.5	0.04533
289	0.04571
289.5	0.04554
290	0.04582
290.5	0.04598
291	0.04616
291.5	0.04571
292	0.04593
292.5	0.04583
293	0.04578
293.5	0.04615
294	0.0455

294.5	0.04595
295	0.04585
295.5	0.04614
296	0.04629
296.5	0.04598
297	0.04572
297.5	0.04593
298	0.04585
298.5	0.04581
299	0.04575
299.5	0.0458
300	0.04638
300.5	0.04601
301	0.04561
301.5	0.04525
302	0.04599
302.5	0.04566
303	0.04582
303.5	0.04607
304	0.04585
304.5	0.04573
305	0.04584
305.5	0.04608
306	0.04634
306.5	0.0458
307	0.0455
307.5	0.04572
308	0.04583
308.5	0.04589
309	0.04533
309.5	0.04576
310	0.04612
310.5	0.04604
311	0.04585
311.5	0.04581
312	0.04613
312.5	0.0458
313	0.04575
313.5	0.04593
314	0.04533
314.5	0.04514
315	0.04573
315.5	0.04599
316	0.04525
316.5	0.0452
317	0.04513
317.5	0.04528
318	0.04547
318.5	0.0457
319	0.04601
319.5	0.04586
320	0.04537
320.5	0.04563
321	0.04572
321.5	0.04605
322	0.04577
322.5	0.04587
323	0.04544
323.5	0.04568
324	0.04564
324.5	0.04574
325	0.04537
325.5	0.04547
326	0.04571
326.5	0.04587
327	0.04604
327.5	0.04602
328	0.04557

328.5	0.04565
329	0.04576
329.5	0.04602
330	0.04588
330.5	0.04549
331	0.04567
331.5	0.04605
332	0.04548
332.5	0.04601
333	0.04546
333.5	0.04625
334	0.04576
334.5	0.04554
335	0.04578
335.5	0.04507
336	0.04567
336.5	0.04604
337	0.04594
337.5	0.04573
338	0.04579
338.5	0.04564
339	0.04564
339.5	0.04553
340	0.0463
340.5	0.04603
341	0.04645
341.5	0.04567
342	0.04584
342.5	0.04553
343	0.04585
343.5	0.04618
344	0.04625
344.5	0.04592
345	0.04575
345.5	0.04608
346	0.04582
346.5	0.04592
347	0.04559
347.5	0.04567
348	0.04605
348.5	0.04681
349	0.04562
349.5	0.04638
350	0.04608
350.5	0.04561
351	0.04596
351.5	0.04601
352	0.04523
352.5	0.046
353	0.0451
353.5	0.04504
354	0.04553
354.5	0.04547
355	0.0455
355.5	0.04573
356	0.04573
356.5	0.04598
357	0.04556
357.5	0.04569
358	0.04584
358.5	0.04528
359	0.0453
359.5	0.04554
360	0.04609
360.5	0.04624
361	0.04553
361.5	0.04552
362	0.04531

362.5	0.04567
363	0.04555
363.5	0.04447
364	0.0456
364.5	0.04583
365	0.04596
365.5	0.04584
366	0.0457
366.5	0.04555
367	0.04511
367.5	0.04556
368	0.04538
368.5	0.04504
369	0.04543
369.5	0.04491
370	0.04553
370.5	0.04593
371	0.04634
371.5	0.04621
372	0.04591
372.5	0.04619
373	0.04572
373.5	0.04552
374	0.04546
374.5	0.04581
375	0.04605
375.5	0.04587
376	0.04624
376.5	0.04614
377	0.04582
377.5	0.04624
378	0.04577
378.5	0.04616
379	0.0458
379.5	0.04585
380	0.04557
380.5	0.04558
381	0.04539
381.5	0.04566
382	0.04566
382.5	0.04563
383	0.04575
383.5	0.04595
384	0.04582
384.5	0.04595
385	0.04662
385.5	0.04641
386	0.04585
386.5	0.04647
387	0.04634
387.5	0.04621
388	0.04561
388.5	0.04548
389	0.04574
389.5	0.04578
390	0.04598
390.5	0.04583
391	0.04599
391.5	0.04587
392	0.04584
392.5	0.04572
393	0.04571
393.5	0.04522
394	0.04526
394.5	0.0457
395	0.04622
395.5	0.04609

Absorbance at 532 nm for compound **5**

Time (s)	Absorbance
0	0.054464813
1	0.054437635
2	0.054404737
3	0.054451465
4	0.054440495
5	0.054302215
6	0.054170612
7	0.054245468
8	0.054227829
9	0.054221151
10	0.054074291
11	0.054098132
12	0.054139619
13	0.054019923
14	0.054082395
15	0.05403853
16	0.053856847
17	0.053951265
18	0.053942207
19	0.053754807
20	0.053851123
21	0.05371475
22	0.0538559
23	0.053825377
24	0.0537076
25	0.053809639
26	0.05380344
27	0.053622727
28	0.053572658
29	0.053556439
30	0.053636552
31	0.053605556
32	0.053609369
33	0.053584095
34	0.053464414
35	0.053336622
36	0.053362847
37	0.053363797
38	0.053277492
39	0.053285594
40	0.053291317
41	0.053304198
42	0.053085801
43	0.053133012
44	0.053065781
45	0.052980427
46	0.053102014
47	0.053153041
48	0.053039078
49	0.052919386
50	0.053244111
51	0.053187849
52	0.05289602
53	0.052922249
54	0.052971366
55	0.052953716
56	0.052930834
57	0.052793982
58	0.052788259
59	0.052785875
60	0.052893639
61	0.052769184
62	0.052603725
63	0.052915574
64	0.052768704
65	0.052632334

66	0.052377228
67	0.052441599
68	0.052687647
69	0.05245924
70	0.052544122
71	0.052511218
72	0.05238247
73	0.052615645
74	0.052509303
75	0.052492138
76	0.05250168
77	0.052510742
78	0.052324296
79	0.05227852
80	0.052460192
81	0.05224991
82	0.052368166
83	0.05223846
84	0.052051069
85	0.052259447
86	0.052155974
87	0.052267553
88	0.052250859
89	0.052241801
90	0.052054401
91	0.052115436
92	0.052094463
93	0.052005772
94	0.052079676
95	0.052015305
96	0.05206728
97	0.051935674
98	0.051999094
99	0.051980495
100	0.05197191

Absorbance at 445 nm for compound **5**

Time (s)	Absorbance
0	0.09474
0.5	0.0956
1	0.0953
1.5	0.09529
2	0.09497
2.5	0.09485
3	0.09437
3.5	0.09525
4	0.09368
4.5	0.09452
5	0.09344
5.5	0.09423
6	0.09282
6.5	0.09291
7	0.09317
7.5	0.09341
8	0.09311
8.5	0.09322
9	0.09376
9.5	0.09296
10	0.0926
10.5	0.09235
11	0.09307
11.5	0.09318
12	0.09282
12.5	0.09245
13	0.09248
13.5	0.09235
14	0.09203
14.5	0.09238

15	0.09199
15.5	0.09127
16	0.09102
16.5	0.09117
17	0.0914
17.5	0.09154
18	0.09184
18.5	0.09207
19	0.09151
19.5	0.09068
20	0.09038
20.5	0.0904
21	0.09056
21.5	0.09087
22	0.09035
22.5	0.08999
23	0.09072
23.5	0.09017
24	0.09016
24.5	0.0895
25	0.09001
25.5	0.08945
26	0.0893
26.5	0.08914
27	0.0895
27.5	0.0894
28	0.08872
28.5	0.08858
29	0.08895
29.5	0.08933
30	0.08909
30.5	0.0889
31	0.0889
31.5	0.08862
32	0.08852
32.5	0.08797
33	0.08848
33.5	0.08805
34	0.08868
34.5	0.08859
35	0.08825
35.5	0.08843
36	0.08792
36.5	0.08875
37	0.08799
37.5	0.08706
38	0.08789
38.5	0.08764
39	0.08759
39.5	0.08763
40	0.08703
40.5	0.0872
41	0.08712
41.5	0.08714
42	0.08756
42.5	0.08721
43	0.08699
43.5	0.08791
44	0.08744
44.5	0.08758
45	0.08673
45.5	0.08688
46	0.08664
46.5	0.0863
47	0.08618
47.5	0.08645
48	0.08623
48.5	0.08707

49	0.08594
49.5	0.08661
50	0.08618
50.5	0.08634
51	0.08596
51.5	0.08618
52	0.08569
52.5	0.08607
53	0.08616
53.5	0.08642
54	0.08629
54.5	0.08665
55	0.08635
55.5	0.08572
56	0.08556
56.5	0.08522
57	0.08561
57.5	0.08511
58	0.0858
58.5	0.08566
59	0.08601
59.5	0.08581
60	0.08596
60.5	0.08538
61	0.08545
61.5	0.08593
62	0.08449
62.5	0.08415
63	0.08422
63.5	0.0844
64	0.08375
64.5	0.08492
65	0.08479
65.5	0.08449
66	0.08502
66.5	0.08481
67	0.08485
67.5	0.08481
68	0.08475
68.5	0.08454
69	0.08514
69.5	0.08481
70	0.08512
70.5	0.08476
71	0.08465
71.5	0.08481
72	0.08461
72.5	0.08412
73	0.08421
73.5	0.08418
74	0.08398
74.5	0.08405
75	0.08418
75.5	0.08366
76	0.08309
76.5	0.08434
77	0.08427
77.5	0.08408
78	0.0843
78.5	0.08468
79	0.08386
79.5	0.08395
80	0.08339
80.5	0.08426
81	0.08435
81.5	0.08415
82	0.08485
82.5	0.0842

83	0.08436
83.5	0.08462
84	0.08412
84.5	0.08432
85	0.08392
85.5	0.08455
86	0.08404
86.5	0.08366
87	0.08395
87.5	0.08414
88	0.0841
88.5	0.08404
89	0.08428
89.5	0.08426
90	0.08433
90.5	0.08371
91	0.0837
91.5	0.08367
92	0.08419
92.5	0.08446
93	0.08396
93.5	0.08441
94	0.08434
94.5	0.08409
95	0.08432
95.5	0.08442
96	0.08409
96.5	0.0841
97	0.08398
97.5	0.08401
98	0.08364
98.5	0.08404
99	0.08386
99.5	0.0836
100	0.08382
100.5	0.08386
101	0.08356
101.5	0.08337
102	0.08349
102.5	0.08392
103	0.08353
103.5	0.08373
104	0.08427
104.5	0.0839
105	0.08369
105.5	0.08389
106	0.08372
106.5	0.08396
107	0.08384
107.5	0.08363
108	0.08381
108.5	0.08411
109	0.08395
109.5	0.08459
110	0.08443
110.5	0.08423
111	0.08372
111.5	0.08346
112	0.08349
112.5	0.08345
113	0.0841
113.5	0.08371
114	0.08348
114.5	0.08428
115	0.08368
115.5	0.0838
116	0.08329
116.5	0.08376

117	0.08404
117.5	0.0838
118	0.08329
118.5	0.08393
119	0.08293
119.5	0.08319
120	0.08368
120.5	0.08364
121	0.0832
121.5	0.08399
122	0.08363
122.5	0.08432
123	0.08381
123.5	0.08386
124	0.084
124.5	0.08386
125	0.08397
125.5	0.08376
126	0.08395
126.5	0.08394
127	0.08401
127.5	0.08382
128	0.08352
128.5	0.08358
129	0.08381
129.5	0.08352
130	0.08354
130.5	0.08364
131	0.08333
131.5	0.08326
132	0.08366
132.5	0.08367
133	0.08353
133.5	0.08335
134	0.08357
134.5	0.08333
135	0.08392
135.5	0.08369
136	0.08377
136.5	0.08331
137	0.08371
137.5	0.08375
138	0.08357
138.5	0.08304
139	0.08345
139.5	0.08299
140	0.08343
140.5	0.08362
141	0.084
141.5	0.08368
142	0.08388
142.5	0.08353
143	0.08399
143.5	0.08382
144	0.08385
144.5	0.08386
145	0.08406
145.5	0.08367
146	0.08352
146.5	0.08328
147	0.08321
147.5	0.08294
148	0.08268
148.5	0.08384
149	0.08381
149.5	0.08346
150	0.08406
150.5	0.08346

151	0.08366
151.5	0.08326
152	0.08375
152.5	0.08366
153	0.08367
153.5	0.08396
154	0.08358
154.5	0.08359
155	0.08346
155.5	0.08404
156	0.08443
156.5	0.08308
157	0.08375
157.5	0.0839
158	0.08363
158.5	0.08427
159	0.08415
159.5	0.08375
160	0.084
160.5	0.08387
161	0.0837
161.5	0.08378
162	0.08439
162.5	0.08418
163	0.08399
163.5	0.08347
164	0.08386
164.5	0.08387
165	0.08361
165.5	0.08377
166	0.08347
166.5	0.08365
167	0.08372
167.5	0.08398
168	0.08413
168.5	0.08333
169	0.08383
169.5	0.08347
170	0.08355
170.5	0.08367
171	0.0837
171.5	0.0835
172	0.08407
172.5	0.08369
173	0.08361
173.5	0.08336
174	0.08361
174.5	0.08394
175	0.08341
175.5	0.08357
176	0.08329
176.5	0.08355
177	0.08342
177.5	0.08339
178	0.08365
178.5	0.08345
179	0.08349
179.5	0.08343
180	0.08357
180.5	0.08367
181	0.08318
181.5	0.08416
182	0.08327
182.5	0.08321
183	0.083
183.5	0.08299
184	0.0833
184.5	0.08301

185	0.08285
185.5	0.08382
186	0.08326
186.5	0.08348
187	0.0836
187.5	0.08357
188	0.08363
188.5	0.08356
189	0.08367
189.5	0.08365
190	0.08333
190.5	0.08363
191	0.08386
191.5	0.08353
192	0.08327
192.5	0.0839
193	0.08342
193.5	0.08379
194	0.08349
194.5	0.08383
195	0.0836
195.5	0.08401
196	0.08365
196.5	0.08353
197	0.08346
197.5	0.08389
198	0.08387
198.5	0.08387
199	0.08354
199.5	0.08337
200	0.08286
200.5	0.08331
201	0.08335
201.5	0.0834
202	0.0836
202.5	0.08422
203	0.0835
203.5	0.08344
204	0.08376
204.5	0.08388
205	0.08378
205.5	0.08355
206	0.08368
206.5	0.08344
207	0.08363
207.5	0.08328
208	0.08318
208.5	0.0835
209	0.08356
209.5	0.08321
210	0.08352
210.5	0.08371
211	0.08354
211.5	0.08326
212	0.0836
212.5	0.08311
213	0.0839
213.5	0.08408
214	0.08414
214.5	0.08394
215	0.08341
215.5	0.08391
216	0.08444
216.5	0.08392
217	0.0837
217.5	0.08344
218	0.08353
218.5	0.08369

219	0.08368
219.5	0.08373
220	0.08322
220.5	0.08307
221	0.08376
221.5	0.084
222	0.08322
222.5	0.08289
223	0.0829
223.5	0.08355
224	0.0836
224.5	0.08339
225	0.08304
225.5	0.08287
226	0.08355
226.5	0.08367
227	0.08313
227.5	0.08353
228	0.08342
228.5	0.08332
229	0.08333
229.5	0.08379
230	0.08334
230.5	0.08297
231	0.08311
231.5	0.08336
232	0.08386
232.5	0.08398
233	0.08335
233.5	0.08271
234	0.08335
234.5	0.08304
235	0.08313
235.5	0.08381
236	0.08375
236.5	0.08383
237	0.08349
237.5	0.08363
238	0.0836
238.5	0.08285
239	0.0833
239.5	0.08352
240	0.0835
240.5	0.0833
241	0.08378
241.5	0.08327
242	0.08417
242.5	0.08417
243	0.08315
243.5	0.0825
244	0.08285
244.5	0.08333
245	0.08297
245.5	0.08394
246	0.08347
246.5	0.08306
247	0.08341
247.5	0.08304
248	0.08368
248.5	0.08323
249	0.08336
249.5	0.08292
250	0.08302
250.5	0.08317
251	0.0835
251.5	0.08322
252	0.08355
252.5	0.0829

253	0.08311
253.5	0.08367
254	0.0829
254.5	0.08354
255	0.08361
255.5	0.0834
256	0.08336
256.5	0.08305
257	0.08317
257.5	0.08293
258	0.08327
258.5	0.0836
259	0.0835
259.5	0.08409
260	0.08384
260.5	0.08408
261	0.08392
261.5	0.08377
262	0.08381
262.5	0.08423
263	0.08363
263.5	0.08414
264	0.08382
264.5	0.08346
265	0.0831
265.5	0.08297
266	0.08354
266.5	0.08346
267	0.08376
267.5	0.08338
268	0.08364
268.5	0.08366
269	0.08361
269.5	0.08358
270	0.08422
270.5	0.08314
271	0.08401
271.5	0.08358
272	0.0838
272.5	0.08325
273	0.08368
273.5	0.08348
274	0.08331
274.5	0.08336
275	0.08312
275.5	0.08375
276	0.08403
276.5	0.0834
277	0.08444
277.5	0.08378
278	0.08403
278.5	0.08368
279	0.08352
279.5	0.08376
280	0.08331
280.5	0.08329
281	0.0827
281.5	0.0832
282	0.08336
282.5	0.08316
283	0.08302
283.5	0.0838
284	0.08344
284.5	0.08346
285	0.08345
285.5	0.08357
286	0.08325
286.5	0.08365

287	0.08357
287.5	0.0832
288	0.08314
288.5	0.08394
289	0.08342
289.5	0.08292
290	0.0835
290.5	0.08385
291	0.08293
291.5	0.08292
292	0.08298
292.5	0.0832
293	0.08289
293.5	0.08352
294	0.08268
294.5	0.08316
295	0.08304
295.5	0.08308
296	0.08313
296.5	0.08429
297	0.0833
297.5	0.08332
298	0.08317
298.5	0.08357
299	0.08294
299.5	0.08292
300	0.0829
300.5	0.08391
301	0.0836
301.5	0.08333
302	0.0837
302.5	0.08326
303	0.08381
303.5	0.0835
304	0.0832
304.5	0.08346
305	0.0832
305.5	0.08425
306	0.08352
306.5	0.08355
307	0.083
307.5	0.08298
308	0.08365
308.5	0.08363
309	0.08321
309.5	0.08323
310	0.08348
310.5	0.08238
311	0.083
311.5	0.08345
312	0.08366
312.5	0.08368
313	0.08319
313.5	0.0831
314	0.08301
314.5	0.08397
315	0.08402
315.5	0.08365
316	0.08327
316.5	0.08395
317	0.08383
317.5	0.08379
318	0.08348
318.5	0.08349
319	0.08343
319.5	0.08319
320	0.08263
320.5	0.08323

321	0.08304
321.5	0.08291
322	0.08342
322.5	0.08434
323	0.08376
323.5	0.08326
324	0.08346
324.5	0.0834
325	0.0839
325.5	0.08336
326	0.08348
326.5	0.08356
327	0.08343
327.5	0.08352
328	0.08281
328.5	0.08376
329	0.08346
329.5	0.08399
330	0.08329
330.5	0.08311
331	0.08357
331.5	0.08359
332	0.08354
332.5	0.08365
333	0.08331
333.5	0.08353
334	0.08338
334.5	0.08353
335	0.08384
335.5	0.08312
336	0.08378
336.5	0.08364
337	0.08407
337.5	0.08343
338	0.0835
338.5	0.08348
339	0.08369
339.5	0.08375
340	0.08316
340.5	0.0839
341	0.08397
341.5	0.08349
342	0.08348
342.5	0.08338
343	0.08328
343.5	0.08281
344	0.08294
344.5	0.08362
345	0.08385
345.5	0.08346
346	0.08383
346.5	0.08381
347	0.08351
347.5	0.08374
348	0.08313
348.5	0.08327
349	0.08391
349.5	0.0833
350	0.08321
350.5	0.083
351	0.0828
351.5	0.08355
352	0.08325
352.5	0.08349
353	0.08317
353.5	0.08339
354	0.08361
354.5	0.0831

355	0.08362
355.5	0.08331
356	0.08332
356.5	0.0829
357	0.08322
357.5	0.08304
358	0.0832
358.5	0.08333
359	0.0828
359.5	0.08304
360	0.0828
360.5	0.08293
361	0.08316
361.5	0.08303
362	0.0836
362.5	0.08356
363	0.08303
363.5	0.08327
364	0.08325
364.5	0.08334
365	0.08401
365.5	0.08336
366	0.08338
366.5	0.08336
367	0.08346
367.5	0.08342
368	0.08368
368.5	0.08388
369	0.08345
369.5	0.08327
370	0.08333
370.5	0.0832
371	0.08355
371.5	0.0839
372	0.08308
372.5	0.08332
373	0.08333
373.5	0.08315
374	0.08347
374.5	0.08356
375	0.08327
375.5	0.08367
376	0.08326
376.5	0.08425
377	0.08359
377.5	0.08386
378	0.08379
378.5	0.08383
379	0.08435
379.5	0.08328
380	0.08371
380.5	0.08331
381	0.08342
381.5	0.08321
382	0.08324
382.5	0.08375
383	0.0842
383.5	0.08358
384	0.08364
384.5	0.08385
385	0.08367
385.5	0.08362
386	0.08326
386.5	0.08313
387	0.08342
387.5	0.08397
388	0.08368
388.5	0.08404

389	0.08397
389.5	0.0838
390	0.08357
390.5	0.08376

Absorbance at 532 nm for compound **6**

Time (s)	Absorbance
0	0.05204964
1	0.052050595
2	0.052180764
3	0.052155494
4	0.052237507
5	0.052086351
6	0.052036281
7	0.052093985
8	0.052241327
9	0.051992894
10	0.052085399
11	0.052162174
12	0.052167894
13	0.051991466
14	0.051817419
15	0.051844594
16	0.051900868
17	0.051908489
18	0.051921843
19	0.051705365
20	0.051702974
21	0.051831244
22	0.051896096
23	0.051676748
24	0.051380154
25	0.051181788
26	0.050989155
27	0.050975327
28	0.050772195
29	0.05087662
30	0.050873282
31	0.050972461
32	0.050946232
33	0.050940995
34	0.05062962
35	0.050635814
36	0.050934312
37	0.051109314
38	0.051070694
39	0.051092629
40	0.051022053
41	0.051064019
42	0.051084996
43	0.051064971
44	0.051045898
45	0.051093577
46	0.050768848
47	0.050878522
48	0.05085707
49	0.050817492
50	0.05072689
51	0.050728326
52	0.050772192
53	0.050786497
54	0.050775056
55	0.050686356
56	0.050704478
57	0.05071354
58	0.050725463
59	0.050715928

60	0.050596238
61	0.050556656
62	0.050724987
63	0.050726418
64	0.050613403
65	0.050625803
66	0.050678255
67	0.050594808
68	0.050234799
69	0.050214769
70	0.050345896
71	0.050373559
72	0.050271034
73	0.050395008
74	0.050551415
75	0.050375459
76	0.050217148
77	0.050118926
78	0.05021906
79	0.050470351
80	0.050244812
81	0.050280095
82	0.050008778
83	0.050193309
84	0.050014019
85	0.049983974
86	0.050074103
87	0.050173755
88	0.049909113
89	0.050023078
90	0.049849514
91	0.050062183
92	0.049886707
93	0.050006392
94	0.049990177
95	0.04990339
96	0.049864296
97	0.049789906
98	0.049825195
99	0.049930093
100	0.04979658

Absorbance at 532 nm for compound **7**

Time (s)	Absorbance
0	0.07526064
1	0.07523012
2	0.07504606
3	0.0749712
4	0.07482958
5	0.07498026
6	0.07464457
7	0.07477903
8	0.07450056
9	0.07492781
10	0.07448244
11	0.07469797
12	0.0747056
13	0.07454634
14	0.07464123
15	0.07460642
16	0.07465172
17	0.0743103
18	0.07448149
19	0.07473755
20	0.07414055
21	0.07422543
22	0.07414865

23	0.07398844
24	0.0741477
25	0.07425404
26	0.07414341
27	0.07427263
28	0.07411957
29	0.0740943
30	0.07403183
31	0.0741334
32	0.0737524
33	0.07397842
34	0.0738225
35	0.07385015
36	0.07386971
37	0.07383633
38	0.07350016
39	0.07383204
40	0.07353306
41	0.07359362
42	0.07343197
43	0.07338285
44	0.07339334
45	0.07328033
46	0.07342768
47	0.07329273
48	0.07322598
49	0.07325459
50	0.07285929
51	0.07313347
52	0.07311058
53	0.07303858
54	0.07305479
55	0.07309055
56	0.07302094
57	0.07301569
58	0.07285786
59	0.072855
60	0.07273436
61	0.07300138
62	0.0725193
63	0.07266188
64	0.07269049
65	0.07269382
66	0.07262611
67	0.07268572
68	0.07236814
69	0.07266712
70	0.0722928
71	0.07245111
72	0.0723691
73	0.07224131
74	0.07226467
75	0.07180977
76	0.07195139
77	0.07193661
78	0.07215977
79	0.07202339
80	0.07222414
81	0.07190657
82	0.07199955
83	0.07203674
84	0.0717206
85	0.07179689
86	0.07188034
87	0.07180929
88	0.07215786
89	0.07163143
90	0.07166529

91	0.07137871
92	0.07169533
93	0.07150269
94	0.07147598
95	0.07172823
96	0.07159281
97	0.07148409
98	0.071527
99	0.07162809
100	0.07150888

Absorbance at 445 nm for compound **7**

Time (s)	Absorbance
0	0.031987
0.5	0.032241
1	0.031879
1.5	0.031385
2	0.031055
2.5	0.032201
3	0.032727
3.5	0.031084
4	0.031161
4.5	0.030987
5	0.031459
5.5	0.0311
6	0.031211
6.5	0.030903
7	0.030904
7.5	0.030906
8	0.030989
8.5	0.03141
9	0.031591
9.5	0.032369
10	0.03162
10.5	0.030744
11	0.031156
11.5	0.031323
12	0.031253
12.5	0.03046
13	0.03086
13.5	0.031295
14	0.032275
14.5	0.031559
15	0.032019
15.5	0.031577
16	0.031524
16.5	0.030769
17	0.030635
17.5	0.029915
18	0.029922
18.5	0.03099
19	0.031137
19.5	0.030436
20	0.030938
20.5	0.030978
21	0.032126
21.5	0.03153
22	0.030591
22.5	0.031029
23	0.032095
23.5	0.031917
24	0.031485
24.5	0.031549
25	0.032128
25.5	0.031537
26	0.030894
26.5	0.03158

27	0.030752
27.5	0.031042
28	0.0312
28.5	0.031371
29	0.031385
29.5	0.031425
30	0.030705
30.5	0.031051
31	0.031523
31.5	0.031231
32	0.0311
32.5	0.031201
33	0.031145
33.5	0.030855
34	0.030896
34.5	0.031193
35	0.030922
35.5	0.030688
36	0.031028
36.5	0.031285
37	0.031041
37.5	0.030768
38	0.031168
38.5	0.031603
39	0.030934
39.5	0.031225
40	0.032258
40.5	0.032298
41	0.032236
41.5	0.032386
42	0.031669
42.5	0.031826
43	0.03158
43.5	0.03176
44	0.031927
44.5	0.0321
45	0.031712
45.5	0.031773
46	0.031614
46.5	0.031407
47	0.031362
47.5	0.030914
48	0.030847
48.5	0.031001
49	0.030944
49.5	0.030922
50	0.030942
50.5	0.030987
51	0.030662
51.5	0.031311
52	0.031466
52.5	0.031307
53	0.031125
53.5	0.031068
54	0.031089
54.5	0.031125
55	0.030718
55.5	0.030527
56	0.030688
56.5	0.030249
57	0.030858
57.5	0.030772
58	0.030839
58.5	0.030972
59	0.030632
59.5	0.030509
60	0.030599
60.5	0.031034

61	0.030645
61.5	0.03027
62	0.030355
62.5	0.030541
63	0.030633
63.5	0.030717
64	0.03097
64.5	0.030408
65	0.030492
65.5	0.030558
66	0.030527
66.5	0.030197
67	0.030396
67.5	0.03045
68	0.030087
68.5	0.030267
69	0.030137
69.5	0.030233
70	0.030089
70.5	0.029923
71	0.030022
71.5	0.030075
72	0.030349
72.5	0.030166
73	0.030401
73.5	0.030549
74	0.030241
74.5	0.030311
75	0.030244
75.5	0.030073
76	0.030037
76.5	0.029961
77	0.029991
77.5	0.030143
78	0.030649
78.5	0.030315
79	0.030777
79.5	0.030925
80	0.03049
80.5	0.030676
81	0.030507
81.5	0.030187
82	0.030186
82.5	0.030471
83	0.029857
83.5	0.030881
84	0.029846
84.5	0.029608
85	0.029113
85.5	0.029311
86	0.02964
86.5	0.029109
87	0.029473
87.5	0.029775
88	0.030446
88.5	0.03011
89	0.029614
89.5	0.029599
90	0.029176
90.5	0.029173
91	0.029312
91.5	0.030228
92	0.029956
92.5	0.029263
93	0.02986
93.5	0.029615
94	0.029495
94.5	0.029761

95	0.029481
95.5	0.029543
96	0.029735
96.5	0.029474
97	0.029385
97.5	0.029863
98	0.029378
98.5	0.029048
99	0.028916
99.5	0.029162
100	0.029131
100.5	0.028916
101	0.029388
101.5	0.029164
102	0.029221
102.5	0.029548
103	0.029168
103.5	0.029083
104	0.028811
104.5	0.029057
105	0.02936
105.5	0.029245
106	0.028985
106.5	0.028923
107	0.02928
107.5	0.029044
108	0.029028
108.5	0.028797
109	0.028946
109.5	0.029002
110	0.028642
110.5	0.028901
111	0.028459
111.5	0.028393
112	0.02819
112.5	0.028485
113	0.028063
113.5	0.028229
114	0.028493
114.5	0.028672
115	0.028808
115.5	0.02856
116	0.028898
116.5	0.028726
117	0.029073
117.5	0.028407
118	0.02853
118.5	0.028099
119	0.027765
119.5	0.028121
120	0.02831
120.5	0.028063
121	0.028023
121.5	0.02819
122	0.027909
122.5	0.028278
123	0.028164
123.5	0.027833
124	0.027936
124.5	0.027784
125	0.027792
125.5	0.027704
126	0.027603
126.5	0.027687
127	0.027461
127.5	0.02749
128	0.027874
128.5	0.027421

129	0.027544
129.5	0.02813
130	0.027834
130.5	0.027583
131	0.027715
131.5	0.027257
132	0.027284
132.5	0.026986
133	0.027465
133.5	0.027221
134	0.026876
134.5	0.027179
135	0.02738
135.5	0.027483
136	0.027401
136.5	0.027463
137	0.027595
137.5	0.027403
138	0.026958
138.5	0.02712
139	0.027179
139.5	0.027128
140	0.027244
140.5	0.0274
141	0.027295
141.5	0.026639
142	0.02725
142.5	0.027532
143	0.027438
143.5	0.026873
144	0.027276
144.5	0.026863
145	0.027154
145.5	0.027014
146	0.027116
146.5	0.027001
147	0.027045
147.5	0.026754
148	0.027042
148.5	0.026376
149	0.026565
149.5	0.026302
150	0.026598
150.5	0.026299
151	0.026224
151.5	0.026308
152	0.026324
152.5	0.026321
153	0.025949
153.5	0.026115
154	0.025967
154.5	0.026159
155	0.025668
155.5	0.0259
156	0.025898
156.5	0.026043
157	0.026036
157.5	0.02621
158	0.02614
158.5	0.026313
159	0.025935
159.5	0.025631
160	0.025749
160.5	0.025746
161	0.025891
161.5	0.025456
162	0.025735
162.5	0.025738

163	0.025635
163.5	0.02559
164	0.025796
164.5	0.025654
165	0.025591
165.5	0.025347
166	0.025489
166.5	0.025187
167	0.025317
167.5	0.025353
168	0.025221
168.5	0.024935
169	0.025234
169.5	0.02533
170	0.025161
170.5	0.025414
171	0.025179
171.5	0.025169
172	0.024893
172.5	0.025078
173	0.025005
173.5	0.024813
174	0.024898
174.5	0.024941
175	0.024444
175.5	0.0249
176	0.024979
176.5	0.024718
177	0.025126
177.5	0.024517
178	0.02506
178.5	0.024802
179	0.024921
179.5	0.025021
180	0.024811
180.5	0.024606
181	0.024667
181.5	0.024812
182	0.024534
182.5	0.024629
183	0.024454
183.5	0.02452
184	0.02463
184.5	0.023888
185	0.024035
185.5	0.024225
186	0.023891
186.5	0.023906
187	0.024279
187.5	0.024235
188	0.02399
188.5	0.023607
189	0.023896
189.5	0.023937
190	0.023898
190.5	0.023815
191	0.023433
191.5	0.023544
192	0.023762
192.5	0.023942
193	0.023751
193.5	0.023625
194	0.02376
194.5	0.02355
195	0.023491
195.5	0.023557
196	0.023448
196.5	0.02336

197	0.023259
197.5	0.023513
198	0.023107
198.5	0.023293
199	0.02326
199.5	0.023008
200	0.022931
200.5	0.023296
201	0.022966
201.5	0.02306
202	0.023135
202.5	0.022974
203	0.022786
203.5	0.023259
204	0.022858
204.5	0.022594
205	0.023018
205.5	0.022704
206	0.022919
206.5	0.022205
207	0.022552
207.5	0.022579
208	0.022507
208.5	0.022873
209	0.022728
209.5	0.022531
210	0.022333
210.5	0.022835
211	0.02276
211.5	0.022245
212	0.022029
212.5	0.022469
213	0.022646
213.5	0.022345
214	0.022566
214.5	0.022111
215	0.021648
215.5	0.022057
216	0.021931
216.5	0.022051
217	0.022101
217.5	0.021873
218	0.021936
218.5	0.022007
219	0.02103
219.5	0.021601
220	0.021842
220.5	0.021755
221	0.0218
221.5	0.021498
222	0.021528
222.5	0.02188
223	0.022298
223.5	0.022062
224	0.021843
224.5	0.02137
225	0.021422
225.5	0.02124
226	0.020744
226.5	0.021045
227	0.021414
227.5	0.021302
228	0.021139
228.5	0.021023
229	0.021008
229.5	0.020779
230	0.021131
230.5	0.021181

231	0.021075
231.5	0.021212
232	0.02084
232.5	0.020969
233	0.021386
233.5	0.020877
234	0.021223
234.5	0.021101
235	0.020795
235.5	0.020881
236	0.020475
236.5	0.020746
237	0.020646
237.5	0.020579
238	0.020676
238.5	0.020346
239	0.020615
239.5	0.020642
240	0.020325
240.5	0.020688
241	0.020568
241.5	0.020037
242	0.01976
242.5	0.019915
243	0.019924
243.5	0.020156
244	0.020125
244.5	0.02007
245	0.019661
245.5	0.01986
246	0.019741
246.5	0.019615
247	0.020106
247.5	0.019483
248	0.019228
248.5	0.019405
249	0.019358
249.5	0.01958
250	0.019609
250.5	0.019717
251	0.01949
251.5	0.019145
252	0.019417
252.5	0.019303
253	0.019023
253.5	0.019543
254	0.01903
254.5	0.019121
255	0.019325
255.5	0.01924
256	0.019002
256.5	0.019293
257	0.019273
257.5	0.01905
258	0.01915
258.5	0.019112
259	0.018706
259.5	0.018942
260	0.019518
260.5	0.019027
261	0.019184
261.5	0.019363
262	0.018699
262.5	0.018833
263	0.01862
263.5	0.018677
264	0.01857
264.5	0.018385

265	0.018552
265.5	0.018237
266	0.018388
266.5	0.018753
267	0.018717
267.5	0.018564
268	0.018708
268.5	0.018637
269	0.018727
269.5	0.0183
270	0.018357
270.5	0.017715
271	0.017512
271.5	0.017866
272	0.017911
272.5	0.017789
273	0.018087
273.5	0.018044
274	0.017869
274.5	0.018255
275	0.018351
275.5	0.017778
276	0.018025
276.5	0.017834
277	0.017953
277.5	0.017601
278	0.017203
278.5	0.017273
279	0.017566
279.5	0.017554
280	0.017409
280.5	0.01786
281	0.017672
281.5	0.017478
282	0.01764
282.5	0.017476
283	0.017515
283.5	0.016968
284	0.017077
284.5	0.016998
285	0.017039
285.5	0.017166
286	0.01703
286.5	0.01711
287	0.017393
287.5	0.017019
288	0.016674
288.5	0.016364
289	0.017492
289.5	0.01693
290	0.01689
290.5	0.016906
291	0.016794
291.5	0.016993
292	0.016762
292.5	0.017194
293	0.016958
293.5	0.016553
294	0.016644
294.5	0.016829
295	0.016693
295.5	0.016613
296	0.016628
296.5	0.016571
297	0.01632
297.5	0.016239
298	0.016103
298.5	0.01628

299	0.016394
299.5	0.016332
300	0.015998
300.5	0.016091
301	0.016556
301.5	0.016304
302	0.016174
302.5	0.016425
303	0.016198
303.5	0.016118
304	0.016088
304.5	0.016125
305	0.016
305.5	0.015889
306	0.016281
306.5	0.016366
307	0.015968
307.5	0.015781
308	0.015845
308.5	0.015789
309	0.015735
309.5	0.016173
310	0.015827
310.5	0.015028
311	0.015427
311.5	0.015424
312	0.015101
312.5	0.015528
313	0.015371
313.5	0.015403
314	0.015577
314.5	0.015598
315	0.015033
315.5	0.014986
316	0.014829
316.5	0.014835
317	0.015115
317.5	0.015088
318	0.01503
318.5	0.015093
319	0.014783
319.5	0.014746
320	0.015027
320.5	0.014995
321	0.014755
321.5	0.014586
322	0.014869
322.5	0.014716
323	0.014987
323.5	0.015082
324	0.015143
324.5	0.014707
325	0.01436
325.5	0.014968
326	0.014965
326.5	0.014909
327	0.014395
327.5	0.01441
328	0.01461
328.5	0.014435
329	0.014354
329.5	0.014039
330	0.014069
330.5	0.014392
331	0.014558
331.5	0.014857
332	0.014504
332.5	0.014583

333	0.014458
333.5	0.014296
334	0.014257
334.5	0.014464
335	0.014198
335.5	0.014208
336	0.014005
336.5	0.013991
337	0.014164
337.5	0.014599
338	0.014555
338.5	0.014255
339	0.01442
339.5	0.014533
340	0.01421
340.5	0.013906
341	0.013933
341.5	0.013902
342	0.014033
342.5	0.013899
343	0.01396
343.5	0.013956
344	0.013949
344.5	0.01415
345	0.013978
345.5	0.013915
346	0.013507
346.5	0.013689
347	0.013753
347.5	0.013567
348	0.013654
348.5	0.01335
349	0.013739
349.5	0.013674
350	0.013442
350.5	0.014438
351	0.014216
351.5	0.014579
352	0.014366
352.5	0.014261
353	0.013822
353.5	0.013747
354	0.013829
354.5	0.014143
355	0.013962
355.5	0.013796
356	0.014057
356.5	0.013814
357	0.013308
357.5	0.013393
358	0.013279
358.5	0.013262
359	0.013414
359.5	0.013302
360	0.013184
360.5	0.013131
361	0.013083
361.5	0.01305
362	0.01322
362.5	0.013078
363	0.012914
363.5	0.013126
364	0.012919
364.5	0.013161
365	0.012992
365.5	0.012572
366	0.012465
366.5	0.012561

367	0.012348
367.5	0.012631
368	0.012801
368.5	0.01252
369	0.012742
369.5	0.012669
370	0.012883
370.5	0.013508
371	0.013179
371.5	0.01295
372	0.012834
372.5	0.012601
373	0.012558
373.5	0.012853
374	0.012806
374.5	0.013103
375	0.012574
375.5	0.013116
376	0.012736
376.5	0.0122
377	0.012593
377.5	0.012702
378	0.012564
378.5	0.012692
379	0.012643
379.5	0.012498
380	0.01274
380.5	0.012723
381	0.012796
381.5	0.012798
382	0.012622
382.5	0.012647
383	0.012563
383.5	0.012503
384	0.012546
384.5	0.01265
385	0.012363
385.5	0.012586
386	0.012321
386.5	0.012125
387	0.011858
387.5	0.012112
388	0.012206
388.5	0.012084
389	0.011995
389.5	0.01257
390	0.012742
390.5	0.012414
391	0.012108
391.5	0.012382
392	0.012358
392.5	0.012405
393	0.012444
393.5	0.012453
394	0.012425
394.5	0.012383
395	0.012509
395.5	0.012766
396	0.012387
396.5	0.01182
397	0.011938
397.5	0.01207
398	0.01206
398.5	0.0121
399	0.012557
399.5	0.012258
400	0.012184

Absorbance at 532 nm for compound **8**

Time (s)	Absorbance
0	0.019730188
1	0.019542204
2	0.019969882
3	0.019742364
4	0.019879753
5	0.019648474
6	0.019838089
7	0.019777615
8	0.019801874
9	0.020007503
10	0.019970104
11	0.019844382
12	0.019775336
13	0.019843715
14	0.020039565
15	0.019665676
16	0.019857062
17	0.019524342
18	0.019495193
19	0.019536793
20	0.019587386
21	0.019347069
22	0.019559539
23	0.019808798
24	0.019463697
25	0.019510003
26	0.019508557
27	0.019494893
28	0.019390211
29	0.01924265
30	0.019196443
31	0.019171642
32	0.019245172
33	0.019515834
34	0.019315142
35	0.019247429
36	0.019409639
37	0.01917272
38	0.019056083
39	0.019188109
40	0.018834377
41	0.018819662
42	0.019314263
43	0.019273724
44	0.019018313
45	0.019023984
46	0.019076403
47	0.018872649
48	0.018999116
49	0.019215659
50	0.019098218
51	0.019345762
52	0.019042837
53	0.019046993
54	0.018943818
55	0.019363305
56	0.01905418
57	0.019110195
58	0.019499443
59	0.019237026
60	0.019250774
61	0.019246148
62	0.019255686
63	0.019137486
64	0.019413788
65	0.019145009

66	0.019329173
67	0.019181353
68	0.019102214
69	0.019373895
70	0.019319979
71	0.019314364
72	0.01924773
73	0.019112168
74	0.019286861
75	0.019122102
76	0.019266952
77	0.019375503
78	0.019152072
79	0.019346866
80	0.01932048
81	0.019421006
82	0.019347188
83	0.019475398
84	0.019144494
85	0.019429594
86	0.019207702
87	0.019364522
88	0.019082654
89	0.019356113
90	0.019047109
91	0.018998146
92	0.019206535
93	0.019165041
94	0.018959073
95	0.018978983
96	0.018961055
97	0.019050514
98	0.019308017
99	0.019171623
100	0.018976551

Absorbance at 532 nm for compound **9**

Time (s)	Absorbance
0	0.033824926
1	0.033830646
2	0.033874035
3	0.033977032
4	0.033755776
5	0.033942221
6	0.033757214
7	0.03383541
8	0.033824917
9	0.033540729
10	0.033680436
11	0.03370333
12	0.033693317
13	0.033636098
14	0.033840178
15	0.033878804
16	0.033783437
17	0.033821105
18	0.033922197
19	0.033870696
20	0.03395796
21	0.033942224
22	0.033974173
23	0.033978466
24	0.034058097
25	0.034050463
26	0.033957483
27	0.034174445
28	0.034121518

29	0.034003253
30	0.033986089
31	0.033965585
32	0.034144402
33	0.034119127
34	0.034167292
35	0.034026143
36	0.034100537
37	0.034045697
38	0.03405047
39	0.034254554
40	0.034032343
41	0.034144402
42	0.034017082
43	0.034135337
44	0.034170151
45	0.03413916
46	0.034275534
47	0.033997535
48	0.034243583
49	0.03423071
50	0.034177777
51	0.034307956
52	0.034268376
53	0.034187312
54	0.034081931
55	0.03410435
56	0.034125802
57	0.034200194
58	0.034222603
59	0.034124849
60	0.034282688
61	0.034305097
62	0.034264562
63	0.034157752
64	0.034236912
65	0.034077647
66	0.034227846
67	0.034229755
68	0.034091476
69	0.034162998
70	0.034135346
71	0.034139637
72	0.034160134
73	0.034117221
74	0.034058572
75	0.034162999
76	0.034158709
77	0.03414869
78	0.034192082
79	0.034100529
80	0.034072872
81	0.034044269
82	0.034114836
83	0.034116748
84	0.034092422
85	0.034054756
86	0.034019466
87	0.033956526
88	0.033888342
89	0.033881664
90	0.033783433
91	0.033960816
92	0.033883571
93	0.033971788
94	0.03389835
95	0.033961768
96	0.034005167

97	0.03389072
98	0.03404427
99	0.033858301
100	0.033917431
101	0.033928397
102	0.033817766
103	0.033872127
104	0.033828737
105	0.033824439
106	0.033999447
107	0.033862592
108	0.033877376
109	0.033750539
110	0.033774375
111	0.033843996
112	0.033846383
113	0.033944605
114	0.033810142
115	0.03370285
116	0.033856394
117	0.033936025
118	0.033680439
119	0.033805366
120	0.033742903
121	0.033674241
122	0.033682351
123	0.033701895
124	0.033728595
125	0.033623218
126	0.033744331
127	0.033734802
128	0.033401013
129	0.0337863
130	0.033655641
131	0.033555512
132	0.033654216
133	0.033496382
134	0.033535006
135	0.033670907
136	0.033589841
137	0.033690931
138	0.033477305
139	0.033514502
140	0.03360987
141	0.033638951
142	0.03360891
143	0.033656599
144	0.033380507
145	0.033564094
146	0.033474443
147	0.03348303
148	0.03351545
149	0.033484455
150	0.033530232
151	0.033441065
152	0.033592223
153	0.033447743
154	0.033457752
155	0.033508301
156	0.033426284
157	0.033482553
158	0.033497809
159	0.033356669
160	0.033316608
161	0.033207894
162	0.03336954
163	0.0334425
164	0.033401011

165	0.033232684
166	0.033286099
167	0.033284186
168	0.033364772
169	0.033246999
170	0.033374783
171	0.033214095
172	0.03320742
173	0.033163552
174	0.033246043
175	0.033241275
176	0.033295154
177	0.033260824
178	0.033379078
179	0.033335681
180	0.033279899
181	0.033207898
182	0.033250807
183	0.033142092
184	0.033150677
185	0.033323765
186	0.033127312
187	0.03315687
188	0.03327942
189	0.033140183
190	0.033169748
191	0.033181671
192	0.033075332
193	0.033107285
194	0.033143997
195	0.03302336
196	0.03300619
197	0.033030985
198	0.033097747
199	0.03299856
200	0.033051489
201	0.033204559
202	0.033093931
203	0.033234596
204	0.03305674
205	0.033071993
206	0.033035281
207	0.033081534
208	0.032924178
209	0.033144476
210	0.033007625
211	0.032944207
212	0.032916543
213	0.032983776
214	0.03290129
215	0.032791611
216	0.032859329
217	0.032993316
218	0.032866959
219	0.032815456
220	0.032894136
221	0.032629015
222	0.032798765
223	0.03286982
224	0.033007623
225	0.032854557
226	0.032921792
227	0.032897471
228	0.032755373
229	0.032700537
230	0.03272915
231	0.032835488
232	0.032759668

233	0.032803061
234	0.032543178
235	0.032769677
236	0.032593726
237	0.032667161
238	0.032843109
239	0.032629489
240	0.032750604
241	0.032644272
242	0.03262949
243	0.032778263
244	0.032536505
245	0.032565119
246	0.032594683
247	0.032672879
248	0.032545085
249	0.03263521
250	0.032774451
251	0.032612797
252	0.032487393
253	0.032544615
254	0.032463553
255	0.03250361
256	0.032420155
257	0.032672406
258	0.03250408
259	0.032545086
260	0.032517436
261	0.032543187
262	0.032630916
263	0.032469748
264	0.032484532
265	0.032466413
266	0.032480718
267	0.032465939
268	0.032415393
269	0.032444002
270	0.032352444
271	0.032343867
272	0.032533169
273	0.032315734
274	0.032386298
275	0.032368179
276	0.032387733
277	0.032228943
278	0.032361029
279	0.032324795
280	0.032319543
281	0.032293794
282	0.032256606
283	0.032328131
284	0.032356261
285	0.032279494
286	0.032285689
287	0.032367225
288	0.032330036
289	0.032238003
290	0.032261369
291	0.032250405
292	0.032277106
293	0.032447339
294	0.032172677
295	0.032145502
296	0.032185558
297	0.032162186
298	0.032307621
299	0.032053949
300	0.032243731

301	0.032207009
302	0.032179836
303	0.032212261
304	0.032218936
305	0.032028198
306	0.032030585
307	0.031954284
308	0.032070639
309	0.032155512
310	0.032114032
311	0.03204298
312	0.031927109
313	0.032146934
314	0.032069204
315	0.032039646
316	0.031950476
317	0.032010557
318	0.031891818
319	0.032019615
320	0.031980518
321	0.031953808
322	0.031957151
323	0.032000063
324	0.031953337
325	0.031873226
326	0.031999106
327	0.03186321
328	0.032021041
329	0.031978605
330	0.031901835
331	0.03187895
332	0.031829831
333	0.031919
334	0.03199196
335	0.031762602
336	0.031756878
337	0.031720642
338	0.031763075
339	0.031785016
340	0.031907561
341	0.031861786
342	0.031766895
343	0.031699653
344	0.031818872
345	0.03179741
346	0.031825543
347	0.031723021
348	0.031663894
349	0.031777861
350	0.031682492
351	0.031714914
352	0.03170061
353	0.031688214
354	0.031604766
355	0.031664847
356	0.031648155
357	0.031802656
358	0.03174543
359	0.031653876
360	0.031408308
361	0.031602857
362	0.031554694
363	0.031779768
364	0.031678682
365	0.031630036
366	0.031625269
367	0.031561849
368	0.031584264

369	0.031635283
370	0.031641006
371	0.031489846
372	0.031414505
373	0.031664844
374	0.031489847
375	0.03158569
376	0.031507967
377	0.031452176
378	0.031539438
379	0.031561848
380	0.031501766
381	0.031489852
382	0.031391141
383	0.031452175
384	0.031490807
385	0.031501768
386	0.031494136
387	0.031374451
388	0.031363011
389	0.031358239
390	0.031309604
391	0.031441214
392	0.03145695
393	0.03131437
394	0.031467916
395	0.031472204
396	0.031384949
397	0.031438825
398	0.031380656
399	0.031271938
400	0.03130388
401	0.03127146
402	0.031382565
403	0.031393524
404	0.031179903
405	0.031215671
406	0.031226639
407	0.031326767
408	0.031201362
409	0.031432155
410	0.031367306
411	0.03131723
412	0.031217578
413	0.031232837
414	0.031289098
415	0.031282904
416	0.031321051
417	0.031172274
418	0.031170845
419	0.031276226
420	0.03128719
421	0.031186577
422	0.031130313
423	0.031100272
424	0.031154158
425	0.031172757
426	0.031103615
427	0.031102185
428	0.031082627
429	0.031060698
430	0.031147001
431	0.031076907
432	0.03099012
433	0.031164167
434	0.031131266
435	0.031128404
436	0.031067371

437	0.031020637
438	0.031031134
439	0.031024454
440	0.031068799
441	0.030969141
442	0.031083583
443	0.030874248
444	0.031059266
445	0.030921464
446	0.030865197
447	0.030872825
448	0.03091097
449	0.030886177
450	0.03097677
451	0.031060219
452	0.030792233
453	0.030831812
454	0.030843261
455	0.030857091
456	0.030833246
457	0.030797962
458	0.030798911
459	0.030771255
460	0.030807976
461	0.030761718
462	0.030920029
463	0.030889985
464	0.030706879
465	0.030853275
466	0.030898567
467	0.030750749
468	0.030818459
469	0.030941012
470	0.030764103
471	0.030883312
472	0.030748843
473	0.030738354
474	0.030893806
475	0.030745503
476	0.030784127
477	0.030824188
478	0.030659673
479	0.030602455
480	0.030737878
481	0.030817504
482	0.030728812
483	0.030652525
484	0.030685422
485	0.030744553
486	0.030631067
487	0.030649189
488	0.030707358
489	0.030768872
490	0.030641079
491	0.030555253
492	0.030610562
493	0.03066635
494	0.030654904
495	0.030627252
496	0.030551432
497	0.030585766
498	0.030589576
499	0.030649182
500	0.030592442

Absorbance at 445 nm for compound **9**

Time (s)	Absorbance
0	0.02717

0.5	0.02728
1	0.02776
1.5	0.02732
2	0.02685
2.5	0.02749
3	0.02668
3.5	0.02695
4	0.0264
4.5	0.02623
5	0.02631
5.5	0.02629
6	0.02714
6.5	0.02596
7	0.0251
7.5	0.02515
8	0.02532
8.5	0.02621
9	0.02575
9.5	0.02623
10	0.02574
10.5	0.02517
11	0.02504
11.5	0.02516
12	0.02478
12.5	0.02494
13	0.02499
13.5	0.0253
14	0.02489
14.5	0.02496
15	0.02503
15.5	0.02494
16	0.0251
16.5	0.02462
17	0.02513
17.5	0.02524
18	0.02434
18.5	0.02525
19	0.02496
19.5	0.02492
20	0.02483
20.5	0.02515
21	0.02503
21.5	0.02434
22	0.02445
22.5	0.02416
23	0.02475
23.5	0.0242
24	0.02438
24.5	0.02424
25	0.02401
25.5	0.02423
26	0.02396
26.5	0.02373
27	0.02413
27.5	0.02409
28	0.02419
28.5	0.0238
29	0.02412
29.5	0.02376
30	0.02386
30.5	0.02365
31	0.02314
31.5	0.02381
32	0.02348
32.5	0.02382
33	0.02355
33.5	0.02357
34	0.0235

34.5	0.02318
35	0.02305
35.5	0.02302
36	0.0235
36.5	0.02306
37	0.02295
37.5	0.02289
38	0.02323
38.5	0.02276
39	0.02285
39.5	0.02245
40	0.0228
40.5	0.02275
41	0.02279
41.5	0.02294
42	0.02293
42.5	0.02236
43	0.02278
43.5	0.02242
44	0.02221
44.5	0.02207
45	0.02256
45.5	0.02211
46	0.02256
46.5	0.02223
47	0.02204
47.5	0.02192
48	0.02208
48.5	0.02204
49	0.02184
49.5	0.02191
50	0.02191
50.5	0.0219
51	0.02219
51.5	0.02178
52	0.02203
52.5	0.02157
53	0.02143
53.5	0.02162
54	0.0217
54.5	0.02155
55	0.02176
55.5	0.02159
56	0.02172
56.5	0.02122
57	0.02159
57.5	0.02176
58	0.02133
58.5	0.02158
59	0.0213
59.5	0.02173
60	0.02112
60.5	0.02112
61	0.02132
61.5	0.02106
62	0.02114
62.5	0.02155
63	0.02106
63.5	0.021
64	0.02102
64.5	0.02089
65	0.02067
65.5	0.02063
66	0.02073
66.5	0.02073
67	0.02064
67.5	0.0207
68	0.02053

68.5	0.02077
69	0.02058
69.5	0.02063
70	0.02045
70.5	0.0205
71	0.02059
71.5	0.02065
72	0.02097
72.5	0.02045
73	0.02042
73.5	0.02032
74	0.02062
74.5	0.02029
75	0.02083
75.5	0.02018
76	0.02031
76.5	0.01978
77	0.0208
77.5	0.02022
78	0.0203
78.5	0.02037
79	0.02072
79.5	0.02049
80	0.02035
80.5	0.02019
81	0.02028
81.5	0.02011
82	0.02024
82.5	0.02046
83	0.02018
83.5	0.02021
84	0.01996
84.5	0.02023
85	0.02006
85.5	0.02025
86	0.02012
86.5	0.02017
87	0.01974
87.5	0.01924
88	0.01929
88.5	0.01939
89	0.01975
89.5	0.01943
90	0.01999
90.5	0.01945
91	0.01993
91.5	0.01995
92	0.01966
92.5	0.01919
93	0.01955
93.5	0.01963
94	0.01937
94.5	0.01942
95	0.01941
95.5	0.01909
96	0.01945
96.5	0.01936
97	0.01945
97.5	0.01927
98	0.01949
98.5	0.01935
99	0.01942
99.5	0.01922
100	0.01942
100.5	0.01913
101	0.01921
101.5	0.01937
102	0.01956

102.5	0.01916
103	0.01932
103.5	0.01917
104	0.01878
104.5	0.01867
105	0.01896
105.5	0.01898
106	0.01902
106.5	0.01902
107	0.01936
107.5	0.01902
108	0.01884
108.5	0.01902
109	0.01896
109.5	0.01875
110	0.01859
110.5	0.0189
111	0.01874
111.5	0.0186
112	0.01885
112.5	0.01881
113	0.01881
113.5	0.01881
114	0.01876
114.5	0.01844
115	0.01835
115.5	0.01859
116	0.01822
116.5	0.01854
117	0.01866
117.5	0.01847
118	0.0184
118.5	0.01842
119	0.01852
119.5	0.01816
120	0.01836
120.5	0.01833
121	0.01852
121.5	0.01857
122	0.01823
122.5	0.01855
123	0.01861
123.5	0.01834
124	0.01838
124.5	0.01833
125	0.01819
125.5	0.01845
126	0.01816
126.5	0.01826
127	0.0188
127.5	0.01848
128	0.0184
128.5	0.01848
129	0.0184
129.5	0.0184
130	0.01839
130.5	0.01819
131	0.01832
131.5	0.01819
132	0.01808
132.5	0.01807
133	0.01809
133.5	0.01815
134	0.01827
134.5	0.01863
135	0.01816
135.5	0.01829
136	0.01825

136.5	0.01829
137	0.01807
137.5	0.01827
138	0.01836
138.5	0.01857
139	0.0183
139.5	0.01818
140	0.01851
140.5	0.01848
141	0.01821
141.5	0.01803
142	0.01824
142.5	0.01808
143	0.01801
143.5	0.01833
144	0.01799
144.5	0.01796
145	0.01796
145.5	0.01839
146	0.01792
146.5	0.01783
147	0.01835
147.5	0.01794
148	0.01786
148.5	0.0178
149	0.01788
149.5	0.01777
150	0.01795
150.5	0.01766
151	0.01798
151.5	0.01789
152	0.01757
152.5	0.01766
153	0.01817
153.5	0.01794
154	0.01791
154.5	0.01785
155	0.01752
155.5	0.01789
156	0.01777
156.5	0.01794
157	0.01786
157.5	0.01817
158	0.01773
158.5	0.01756
159	0.01773
159.5	0.01775
160	0.01786
160.5	0.01775
161	0.01767
161.5	0.01773
162	0.01766
162.5	0.01779
163	0.0176
163.5	0.01775
164	0.01766
164.5	0.01777
165	0.01791
165.5	0.01792
166	0.01772
166.5	0.01795
167	0.01766
167.5	0.01776
168	0.01785
168.5	0.01744
169	0.01757
169.5	0.01766
170	0.01759

170.5	0.01744
171	0.01781
171.5	0.0178
172	0.01794
172.5	0.0178
173	0.01778
173.5	0.01768
174	0.01783
174.5	0.01772
175	0.01804
175.5	0.01791
176	0.01786
176.5	0.01802
177	0.01775
177.5	0.01783
178	0.01813
178.5	0.01812
179	0.01795
179.5	0.01769
180	0.01781
180.5	0.01773
181	0.01776
181.5	0.01795
182	0.01763
182.5	0.01731
183	0.01785
183.5	0.01777
184	0.01752
184.5	0.01744
185	0.01761
185.5	0.01772
186	0.01778
186.5	0.01776
187	0.01763
187.5	0.01769
188	0.01748
188.5	0.01736
189	0.01754
189.5	0.01764
190	0.0175
190.5	0.01765
191	0.01746
191.5	0.01745
192	0.01748
192.5	0.01745
193	0.01724
193.5	0.0176
194	0.01736
194.5	0.01726
195	0.01737
195.5	0.01764
196	0.01761
196.5	0.01725
197	0.01716
197.5	0.01738
198	0.01772
198.5	0.01777
199	0.01747
199.5	0.01735
200	0.01726
200.5	0.01721
201	0.01732
201.5	0.0174
202	0.01739
202.5	0.01693
203	0.0173
203.5	0.01722
204	0.01741

204.5	0.01737

Absorbance at 532 nm for compound **10**

Time (s)	Absorbance
0	0.06122515
1	0.061625685
2	0.06145939
3	0.06188368
4	0.0617987
5	0.061839946
6	0.061775458
7	0.061910778
8	0.061696524
9	0.0616927
10	0.061644962
11	0.062055917
12	0.061773609
13	0.061681962
14	0.061609061
15	0.061734903
16	0.061908696
17	0.061802546
18	0.061720683
19	0.06201593
20	0.061750309
21	0.06183991
22	0.062104153
23	0.061721273
24	0.061996395
25	0.061826501
26	0.061938134
27	0.06209343
28	0.061843207
29	0.061814635
30	0.061947092
31	0.061810624
32	0.061997094
33	0.061984411
34	0.062308998
35	0.061801227
36	0.061827637
37	0.06223234
38	0.061802386
39	0.06217333
40	0.061875859
41	0.062104698
42	0.061830298
43	0.061957495
44	0.062222745
45	0.062030212
46	0.062012453
47	0.062211531
48	0.061878565
49	0.062115892
50	0.061916765
51	0.061968938
52	0.062071587
53	0.062115688
54	0.061879695
55	0.061985704
56	0.062102151
57	0.062126806
58	0.062024016
59	0.061989987
60	0.06205798
61	0.061898346
62	0.062028814
63	0.062152035

64	0.062055196
65	0.062153659
66	0.062168141
67	0.062091949
68	0.061961587
69	0.061842469
70	0.062028444
71	0.061857429
72	0.06204202
73	0.062233758
74	0.062033726
75	0.062143089
76	0.062129443
77	0.062251782
78	0.061949976
79	0.061864793
80	0.062051208
81	0.062136957
82	0.062214946
83	0.062343535
84	0.062356901
85	0.061911241
86	0.06226065
87	0.062383594
88	0.062278703
89	0.061854835
90	0.062153541
91	0.061825377
92	0.062212145
93	0.062201507
94	0.06194421
95	0.06187457
96	0.061836173
97	0.062165164
98	0.062021569
99	0.062027697
100	0.062091654

Absorbance at 445 nm for compound **10**

Time (s)	Absorbance
0	0.034969
0.5	0.034576
1	0.035342
1.5	0.03613
2	0.036115
2.5	0.035937
3	0.034955
3.5	0.036042
4	0.036447
4.5	0.036669
5	0.03651
5.5	0.035743
6	0.035669
6.5	0.035446
7	0.035504
7.5	0.035676
8	0.035515
8.5	0.036123
9	0.03656
9.5	0.036337
10	0.036144
10.5	0.035339
11	0.03519
11.5	0.034987
12	0.036313
12.5	0.037036
13	0.03663

13.5	0.036073
14	0.036901
14.5	0.036928
15	0.036997
15.5	0.03661
16	0.03646
16.5	0.036396
17	0.036618
17.5	0.036346
18	0.036418
18.5	0.036583
19	0.036713
19.5	0.037194
20	0.036538
20.5	0.037067
21	0.036755
21.5	0.036676
22	0.036484
22.5	0.036533
23	0.036656
23.5	0.036955
24	0.036517
24.5	0.03644
25	0.036732
25.5	0.037114
26	0.037004
26.5	0.03711
27	0.037476
27.5	0.037418
28	0.037107
28.5	0.036721
29	0.037492
29.5	0.037179
30	0.037425
30.5	0.037039
31	0.037423
31.5	0.037007
32	0.0376
32.5	0.037211
33	0.036921
33.5	0.038082
34	0.037529
34.5	0.038192
35	0.038043
35.5	0.03846
36	0.038217
36.5	0.037895
37	0.037638
37.5	0.037936
38	0.037272
38.5	0.038363
39	0.038043
39.5	0.038102
40	0.038466
40.5	0.037922
41	0.037764
41.5	0.037396
42	0.038091
42.5	0.037555
43	0.038366
43.5	0.038272
44	0.037794
44.5	0.038087
45	0.037933
45.5	0.037849
46	0.03792
46.5	0.038318
47	0.038479

47.5	0.038327
48	0.038091
48.5	0.038043
49	0.03791
49.5	0.038129
50	0.038311
50.5	0.038695
51	0.038104
51.5	0.038114
52	0.037597
52.5	0.038118
53	0.0386
53.5	0.038826
54	0.038764
54.5	0.038568
55	0.038692
55.5	0.038373
56	0.038907
56.5	0.038863
57	0.038785
57.5	0.038691
58	0.038374
58.5	0.038461
59	0.038883
59.5	0.038738
60	0.038671
60.5	0.038666
61	0.038629
61.5	0.038571
62	0.03867
62.5	0.038439
63	0.038435
63.5	0.039093
64	0.03867
64.5	0.038405
65	0.039284
65.5	0.038006
66	0.038486
66.5	0.038612
67	0.038548
67.5	0.03894
68	0.038442
68.5	0.038995
69	0.038776
69.5	0.038871
70	0.038864
70.5	0.038664
71	0.038772
71.5	0.03902
72	0.039316
72.5	0.039213
73	0.039051
73.5	0.038722
74	0.03901
74.5	0.039083
75	0.039021
75.5	0.039103
76	0.03955
76.5	0.03968
77	0.039529
77.5	0.038646
78	0.038986
78.5	0.039125
79	0.039329
79.5	0.038844
80	0.039006
80.5	0.039151
81	0.039266

81.5	0.039621
82	0.039452
82.5	0.039612
83	0.039571
83.5	0.039572
84	0.039646
84.5	0.039631
85	0.039146
85.5	0.038711
86	0.038681
86.5	0.039428
87	0.039466
87.5	0.039555
88	0.039699
88.5	0.03974
89	0.039229
89.5	0.039265
90	0.039821
90.5	0.039429
91	0.039715
91.5	0.039923
92	0.039701
92.5	0.039685
93	0.03942
93.5	0.038859
94	0.038892
94.5	0.039299
95	0.039924
95.5	0.03976
96	0.03987
96.5	0.039952
97	0.039706
97.5	0.039448
98	0.039564
98.5	0.039567
99	0.039279
99.5	0.03993
100	0.039636
100.5	0.039194
101	0.039595
101.5	0.039633
102	0.039399
102.5	0.039505
103	0.039689
103.5	0.039854
104	0.039723
104.5	0.03921
105	0.039548
105.5	0.039461
106	0.039785
106.5	0.039761
107	0.039707
107.5	0.039488
108	0.039053
108.5	0.039604
109	0.039872
109.5	0.039807
110	0.040346
110.5	0.040045
111	0.040214
111.5	0.04075
112	0.04016
112.5	0.040433
113	0.040175
113.5	0.039798
114	0.040743
114.5	0.039397
115	0.039562

115.5	0.040131
116	0.040469
116.5	0.039835
117	0.040428
117.5	0.040666
118	0.04068
118.5	0.039954
119	0.040332
119.5	0.040567
120	0.040668
120.5	0.040242
121	0.04047
121.5	0.040312
122	0.040964
122.5	0.040482
123	0.04044
123.5	0.040477
124	0.040327
124.5	0.040604
125	0.040446
125.5	0.04038
126	0.040468
126.5	0.040472
127	0.040827
127.5	0.041068
128	0.04035
128.5	0.040488
129	0.040429
129.5	0.039855
130	0.040319
130.5	0.040439
131	0.040413
131.5	0.040765
132	0.040672
132.5	0.040512
133	0.040333
133.5	0.040737
134	0.041044
134.5	0.040553
135	0.040923
135.5	0.040215
136	0.040407

Emission spectra 420, 445 & 535 LEDs & transmission spectrum band pass filter MV532/20

<u>**Ch. 3: LED 420Z**</u>
Output power (1000um NA 0.5 L=1m): 130 [mW]
Output power (1000um NA 0.63 L=1m): 190 [mW]
Emission Spectrum:

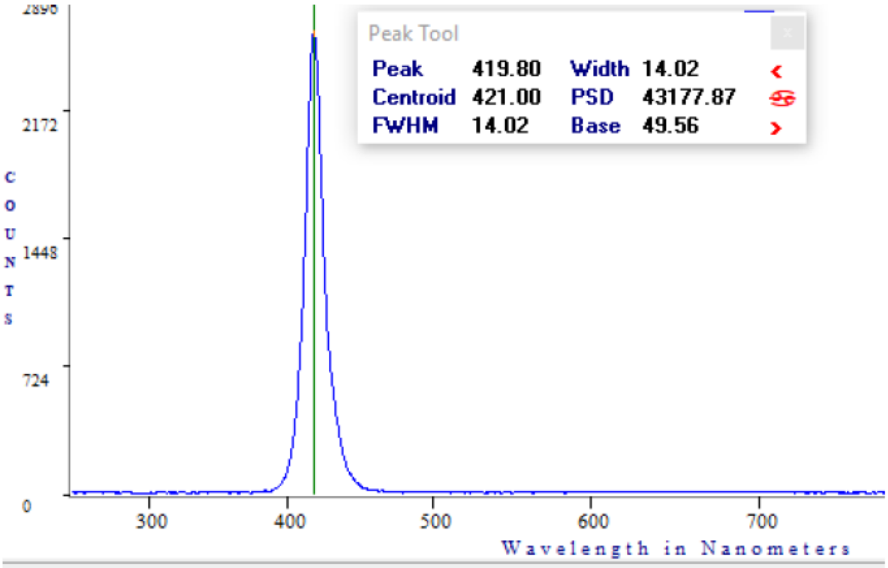

<u>**Ch. 4: LED 445B**</u>
Output power (1000um NA 0.5 L=1m): 65 [mW]
Output power (1000um NA 0.63 L=1m): 85 [mW]
Emission Spectrum:

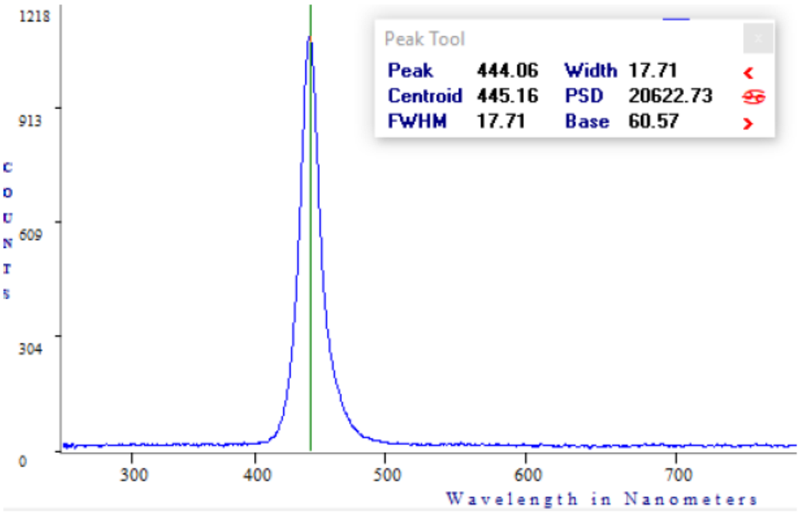

__Ch. 5: LED 535R__

Output power (1000um NA 0.5 L=1m): 115 [mW]

Output power (1000um NA 0.63 L=1m): 160 [mW]

Emission Spectrum:

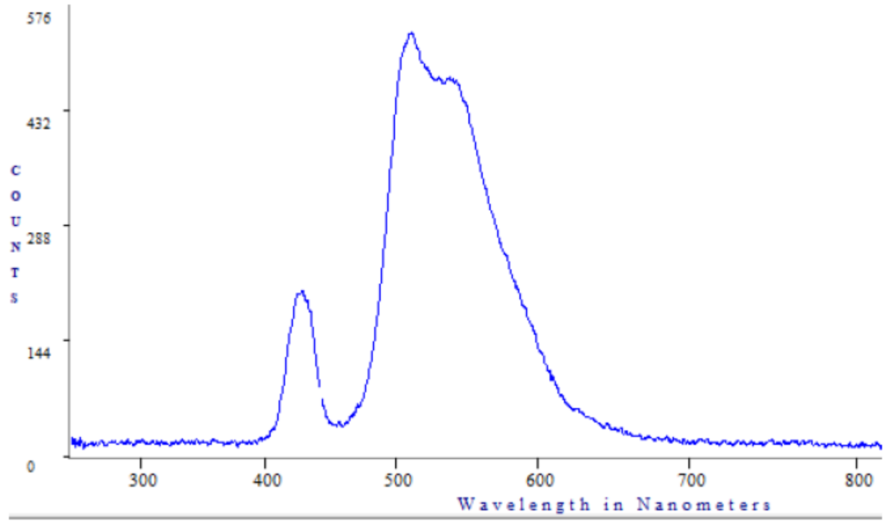

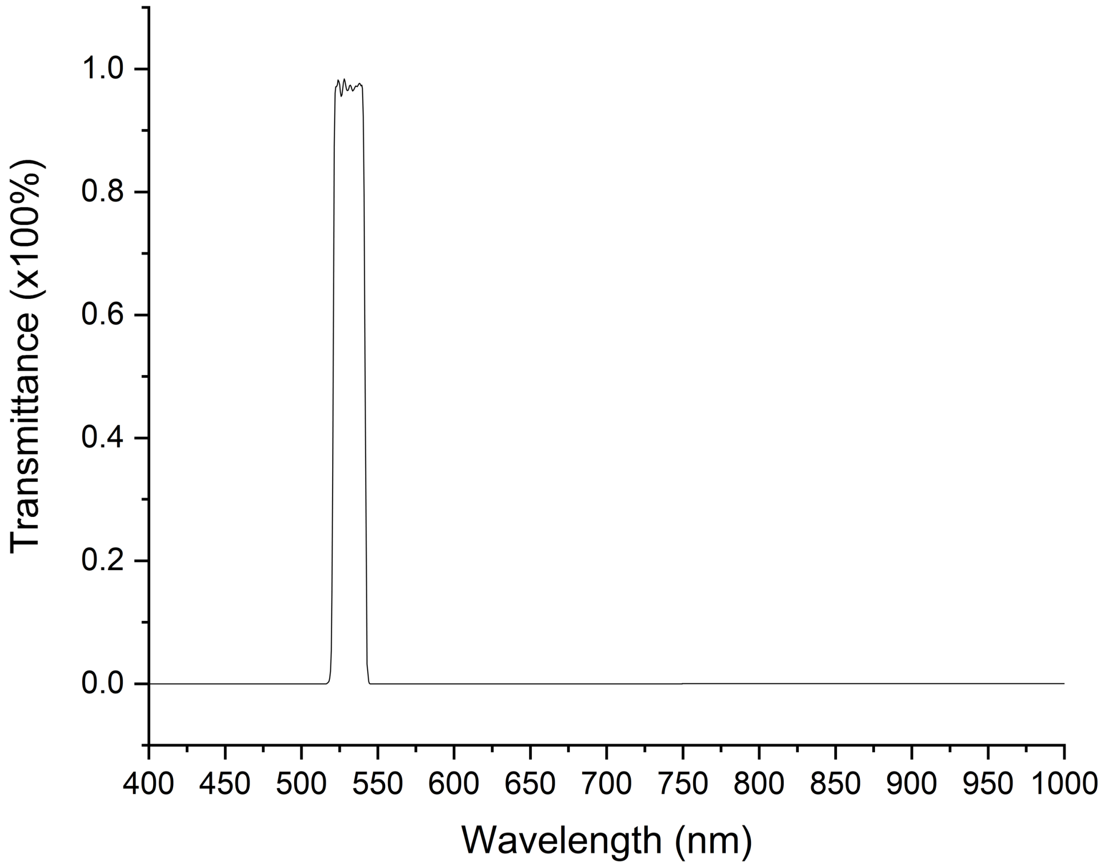

Transmission spectrum of Chroma Narrow GreenMV532/20 band pass filter.

NMR spectra (^{1}H NMR, ^{13}C APT, ^{19}F NMR) and FT-IR spectra 1 – 10

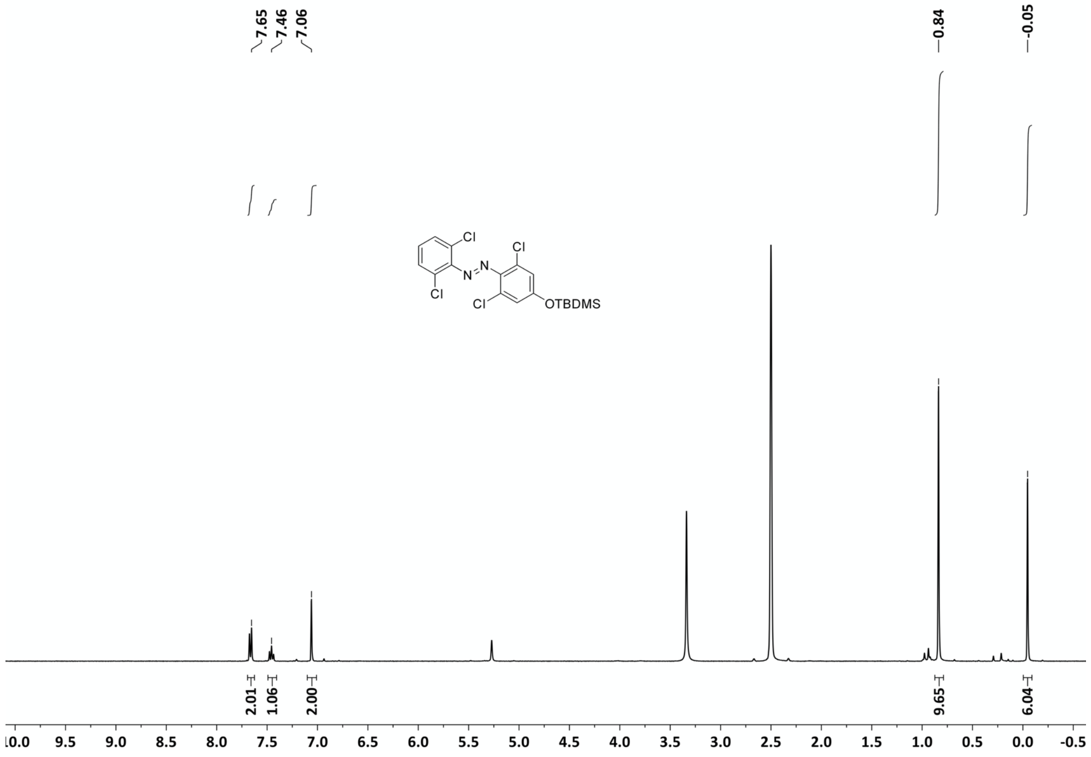

Figure 112. ^{1}HNMR spectrum of 2,2',4-(*O*-tert-butyldimethylsilyl)-6,6'-tetra-chloroazobenzene.

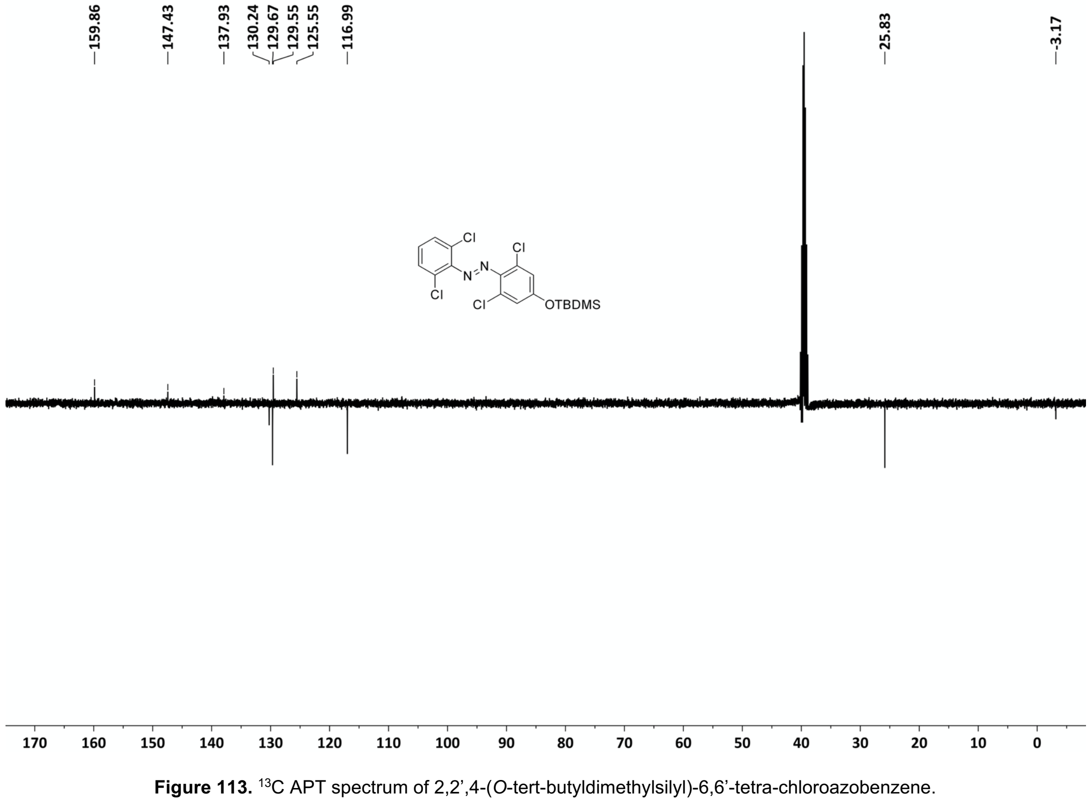

Figure 113. ^{13}C APT spectrum of 2,2',4-(*O*-tert-butyldimethylsilyl)-6,6'-tetra-chloroazobenzene.

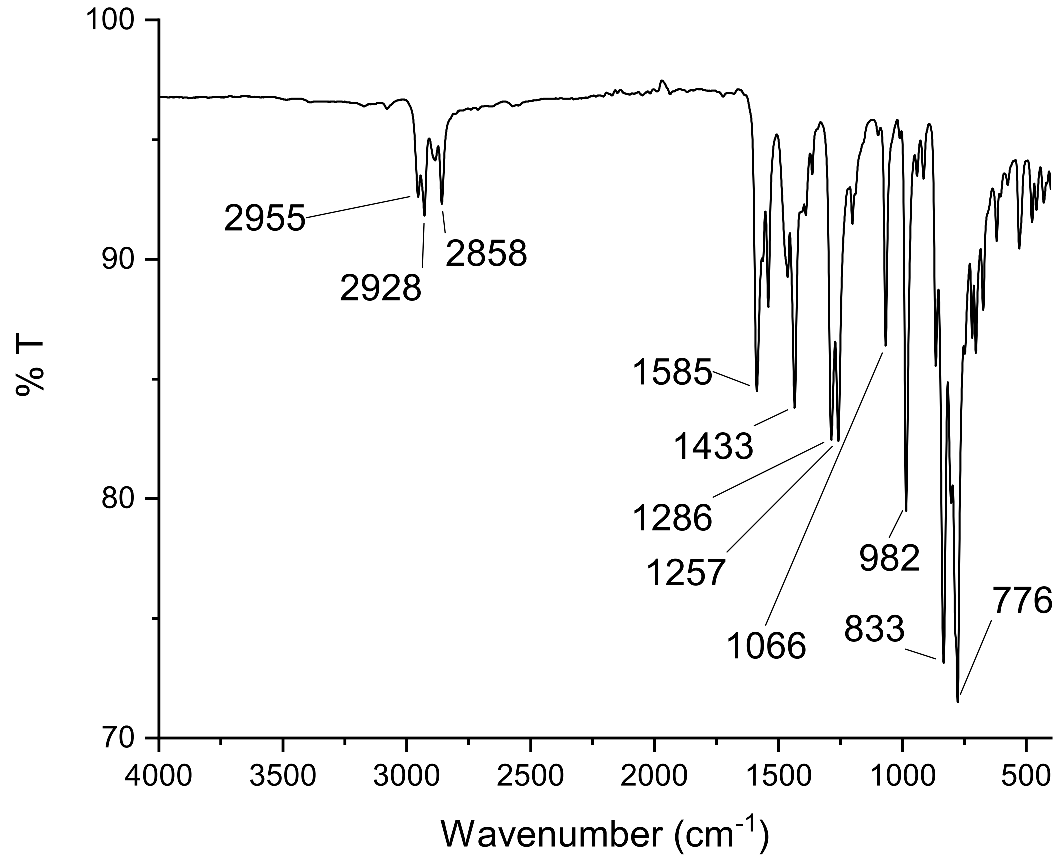

Figure 114. FT-IR spectrum of 2,2',4-(*O*-tert-butyldimethylsilyl)-6,6'-tetra-chloroazobenzene.

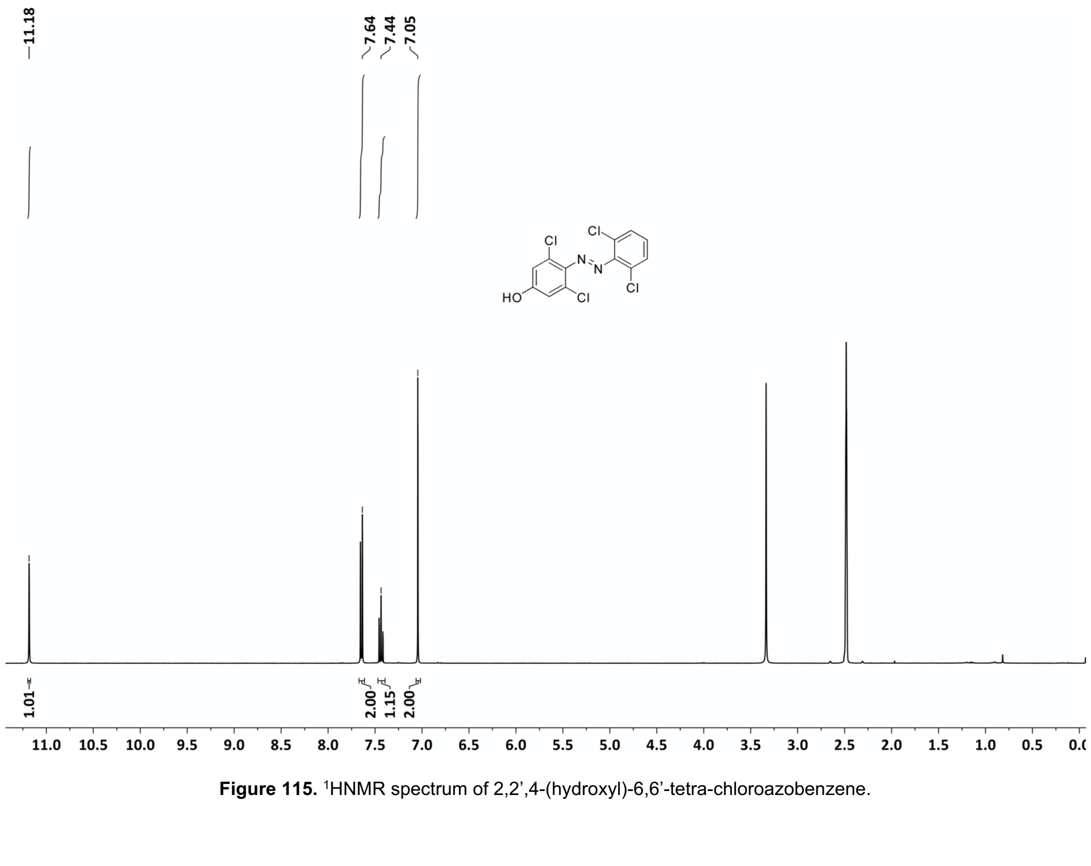

Figure 115. ^{1}HNMR spectrum of 2,2',4-(hydroxyl)-6,6'-tetra-chloroazobenzene.

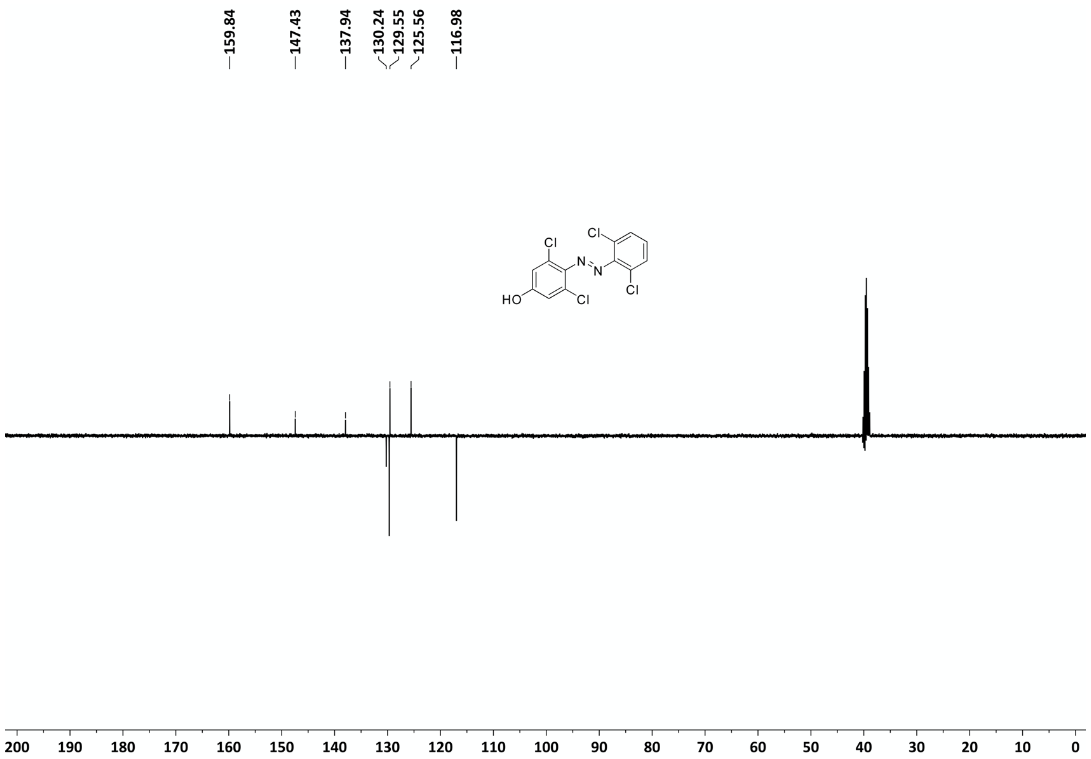

Figure 116. ^{13}C APT spectrum of2,2',4-(hydroxyl)-6,6'-tetra-chloroazobenzene.

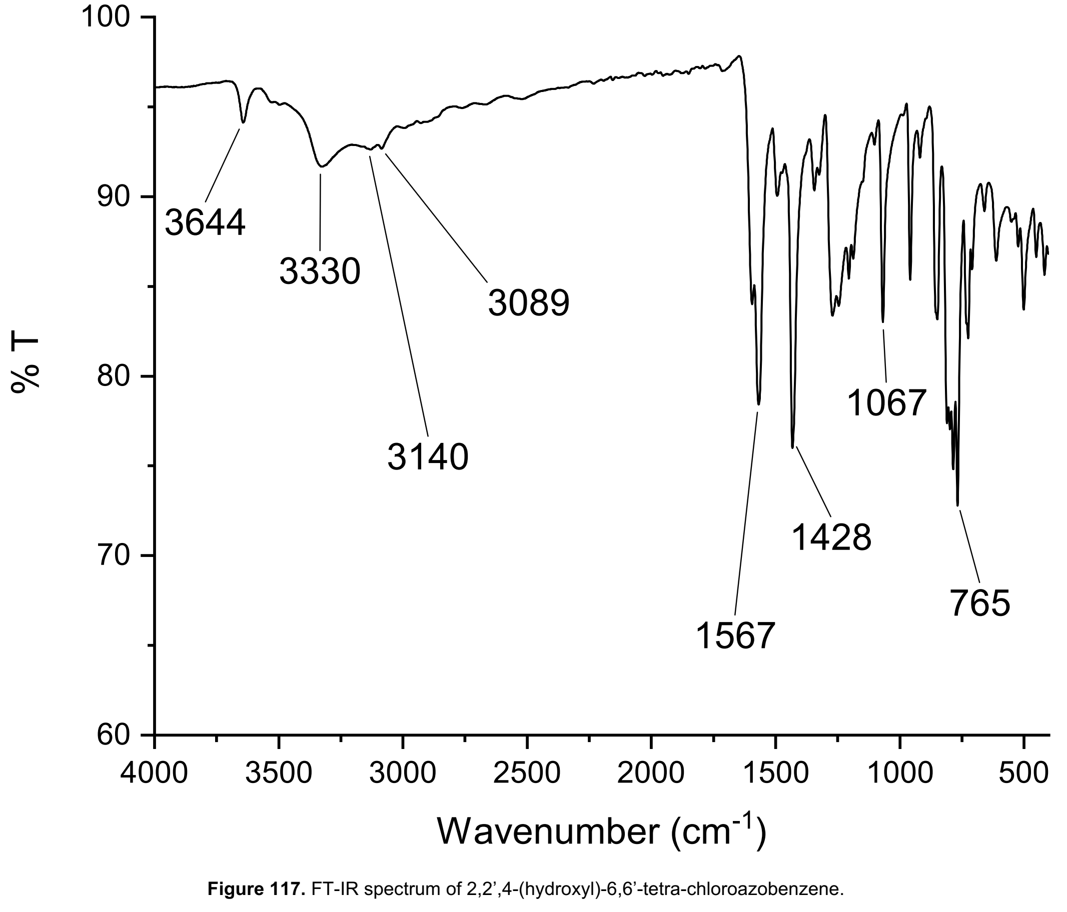

Figure 117. FT-IR spectrum of 2,2',4-(hydroxyl)-6,6'-tetra-chloroazobenzene.

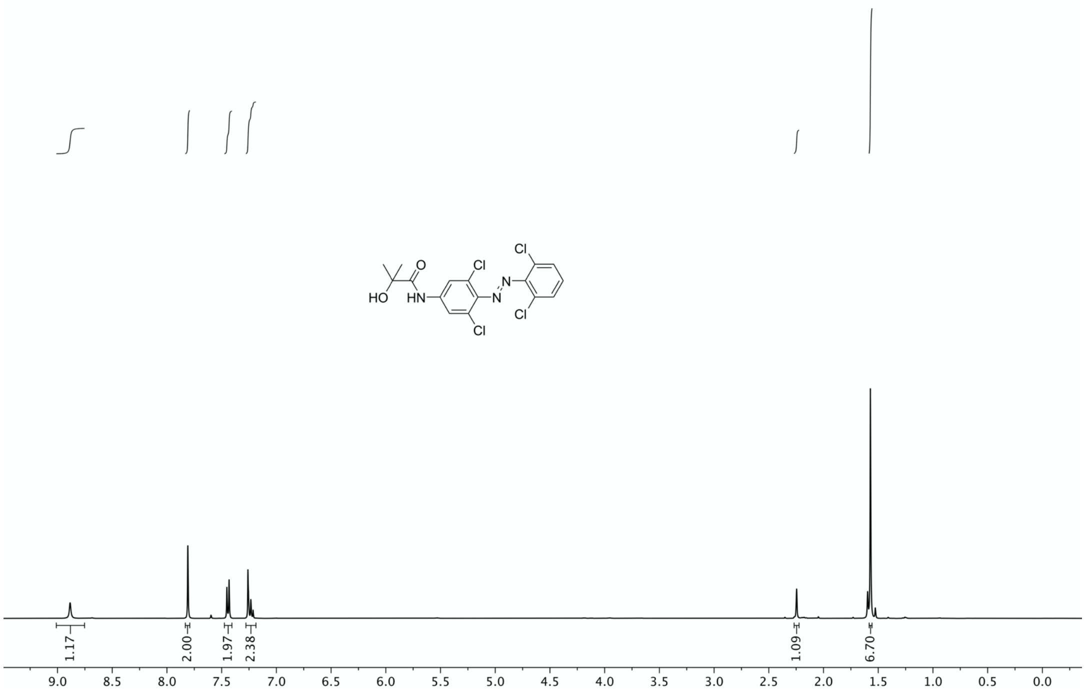

Figure 118. ^{1}HNMR spectrum of 4-(2-hydroxy-2-methylpropanamide)-2,2',6,6'-tetra-chloroazobenzene.

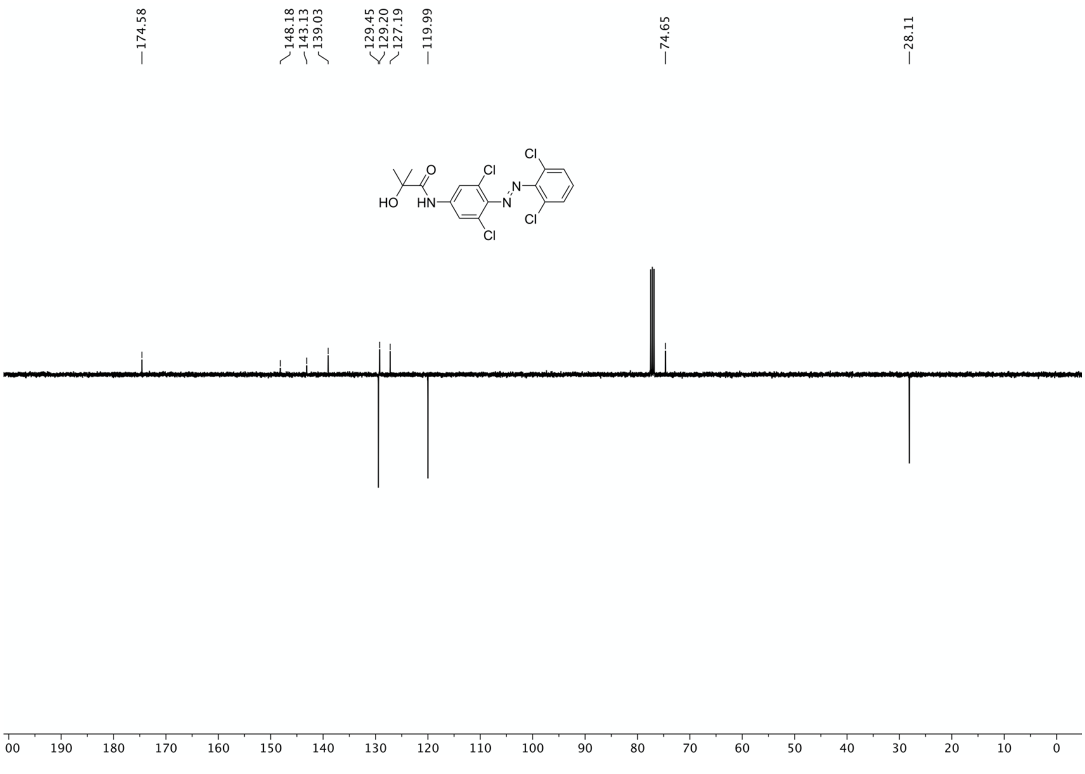

Figure 119. ^{13}C APT spectrum of 4-(2-hydroxy-2-methylpropanamide)-2,2',6,6'-tetra-chloroazobenzene.

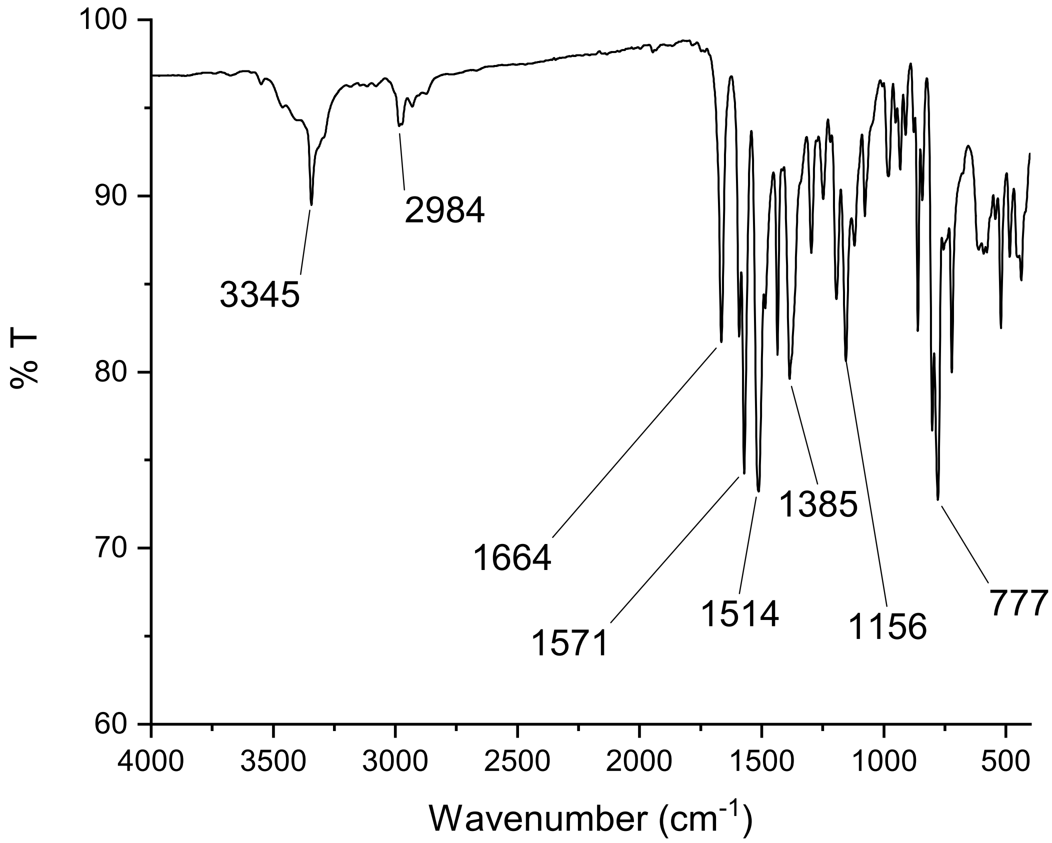

Figure 120. FT-IR spectrum of 4-(2-hydroxy-2-methylpropanamide)-2,2',6,6'-tetra-chloroazobenzene.

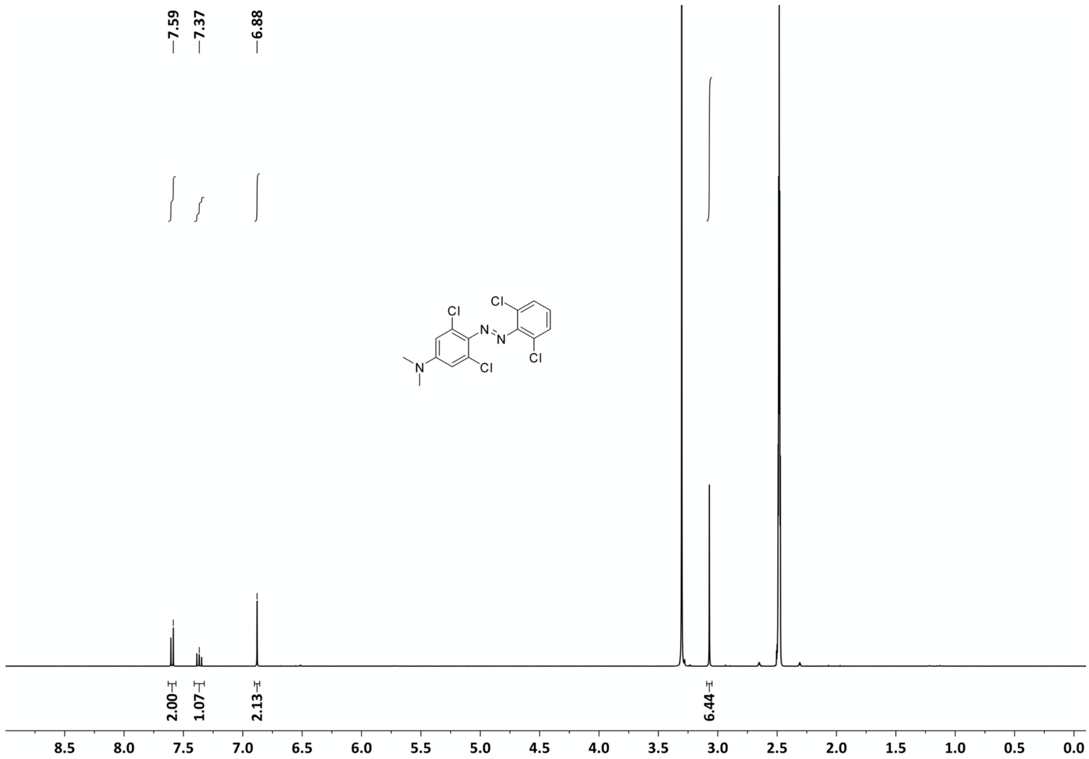

Figure 121. ^{1}HNMR spectrum of **1**.

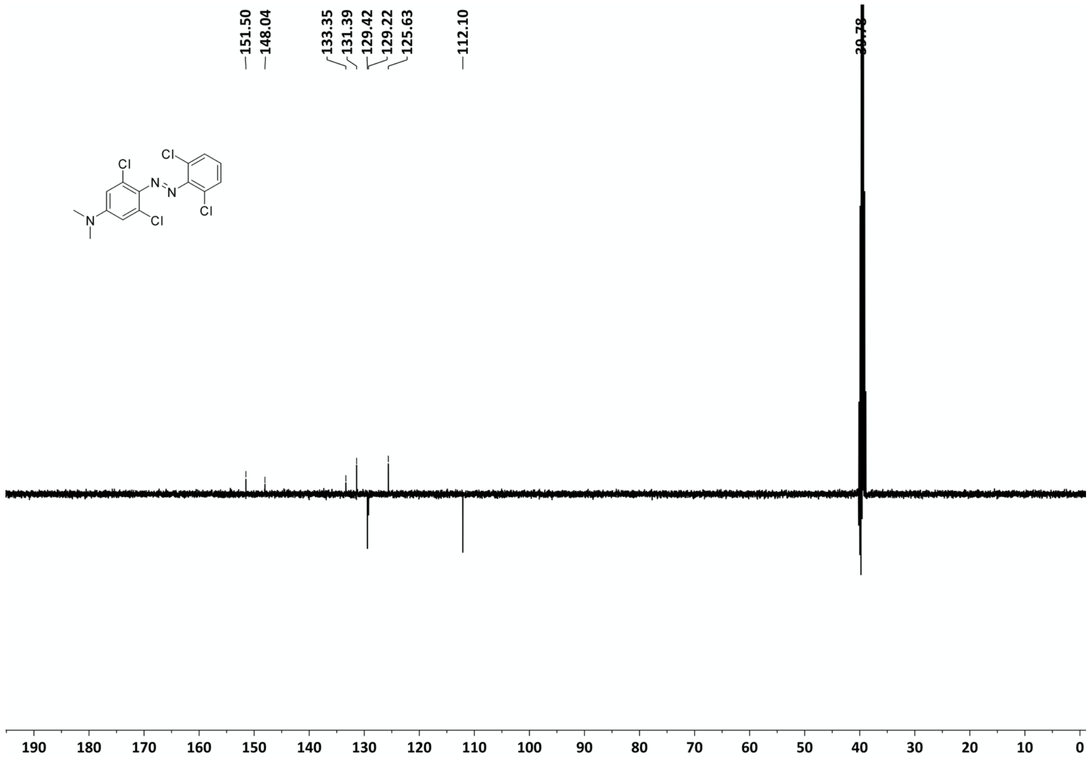

Figure 122. ^{13}C APT spectrum of **1**.

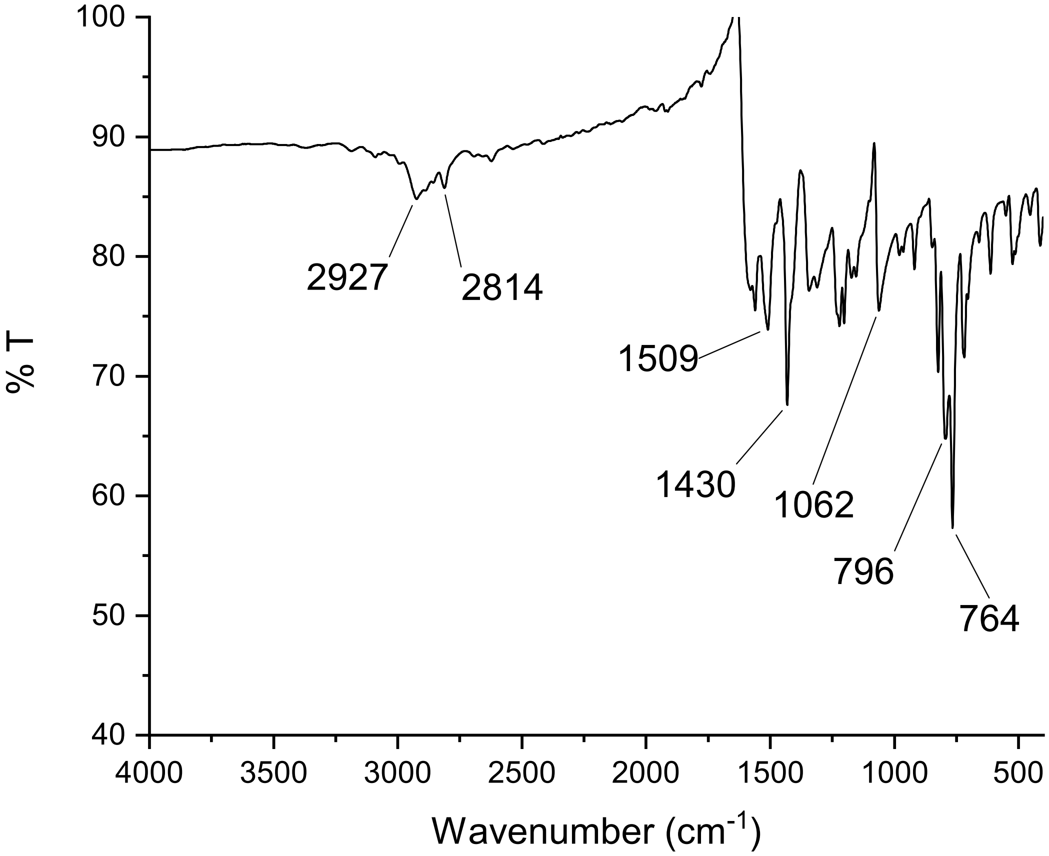

Figure 123. FT-IR spectrum of **1**.

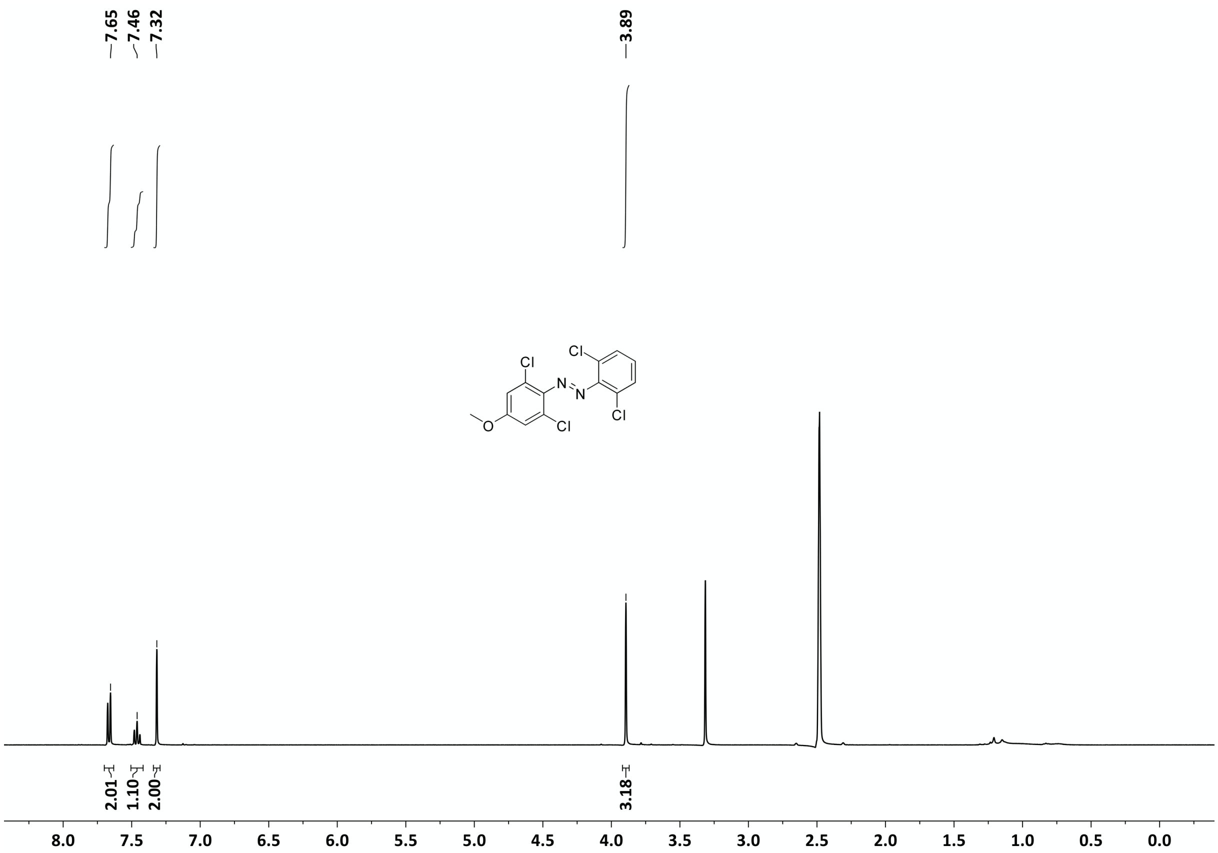

Figure 124. ^{1}HNMR spectrum of **2**.

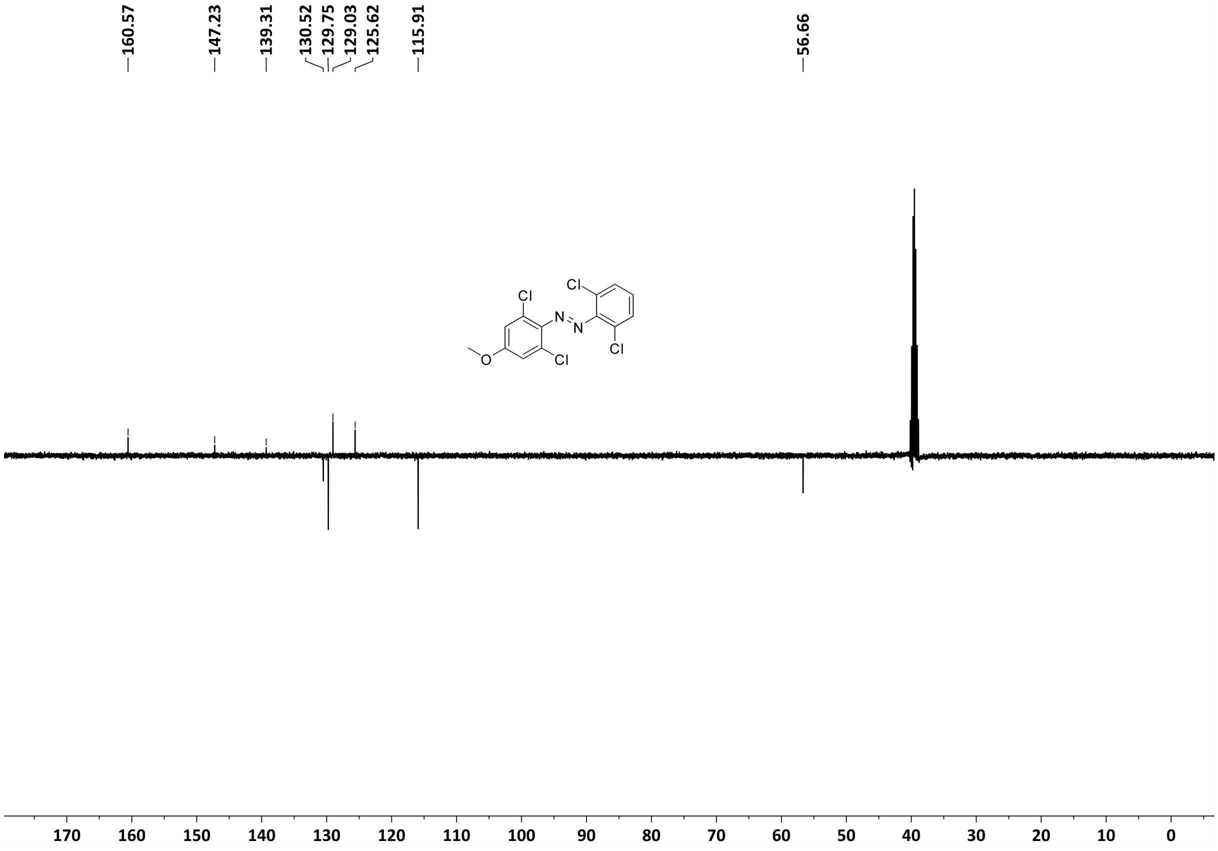

Figure 125. ^{13}C APT spectrum of **2**.

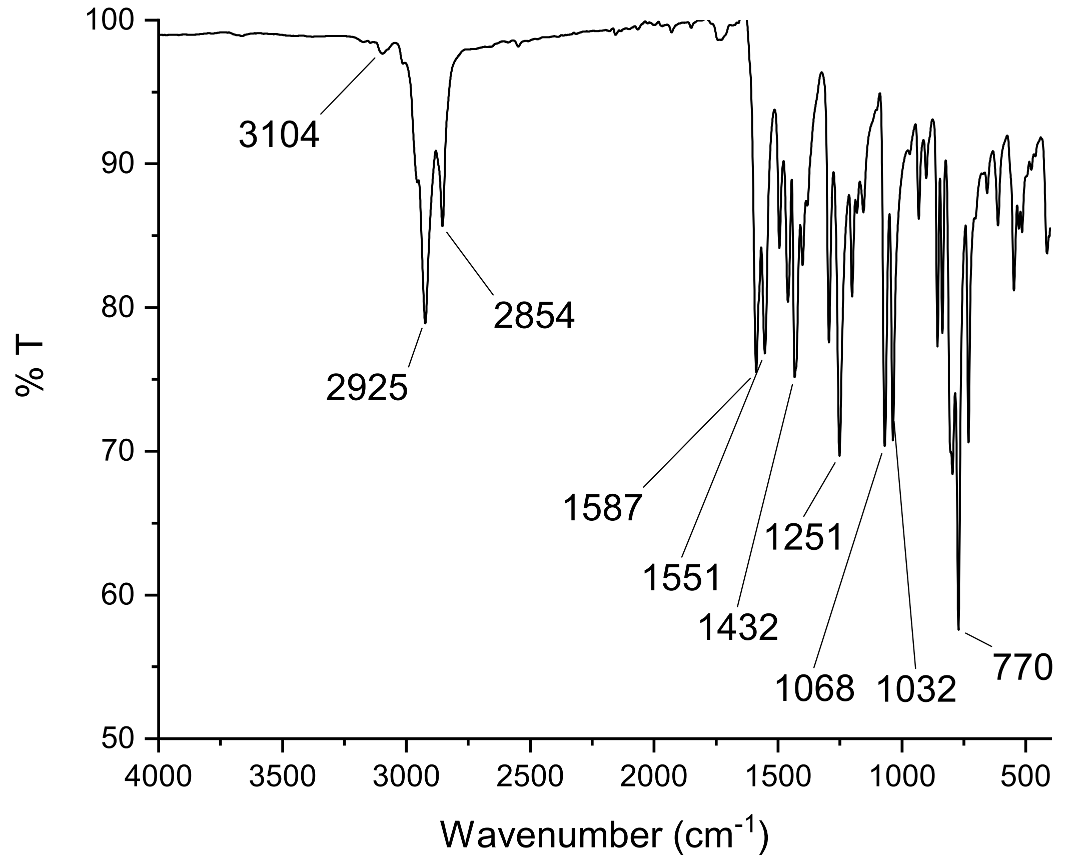

Figure 126. FT-IR spectrum of **2.**

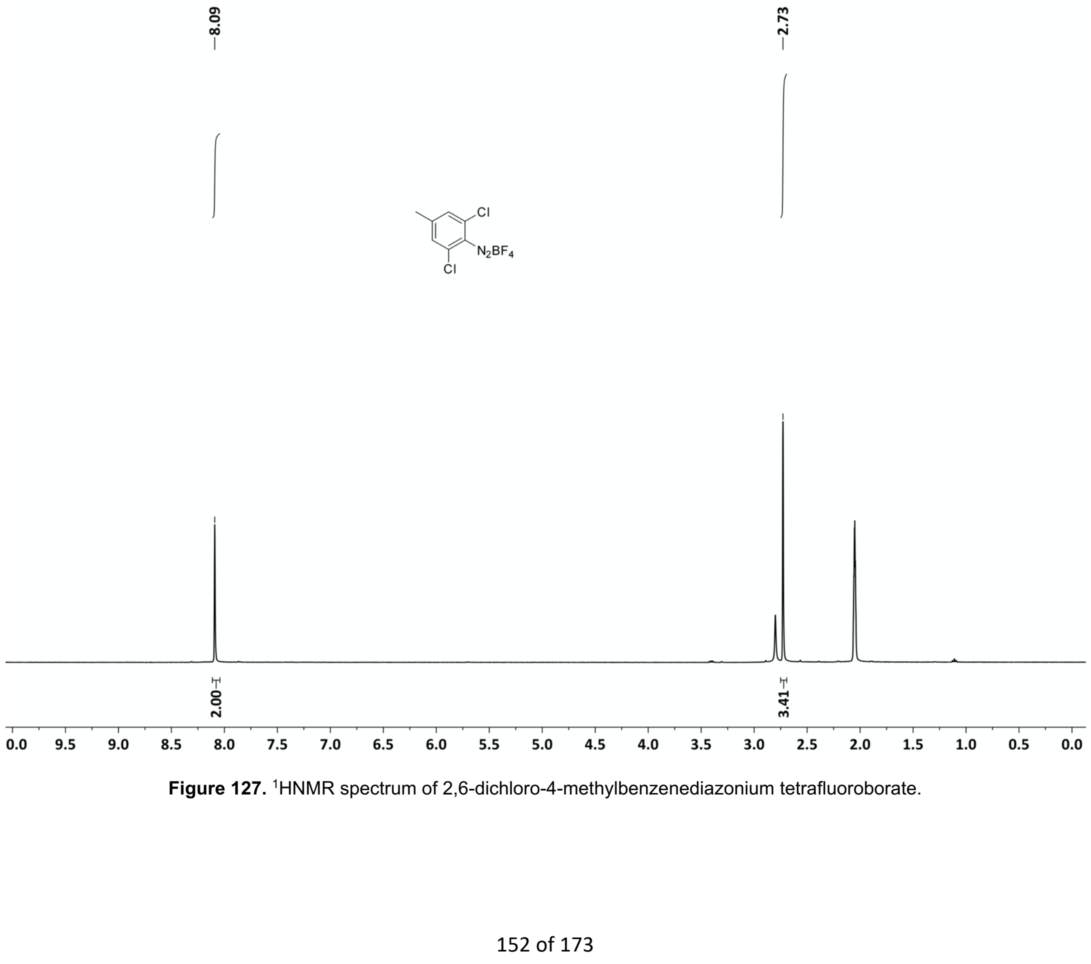

Figure 127. ^{1}HNMR spectrum of 2,6-dichloro-4-methylbenzenediazonium tetrafluoroborate.

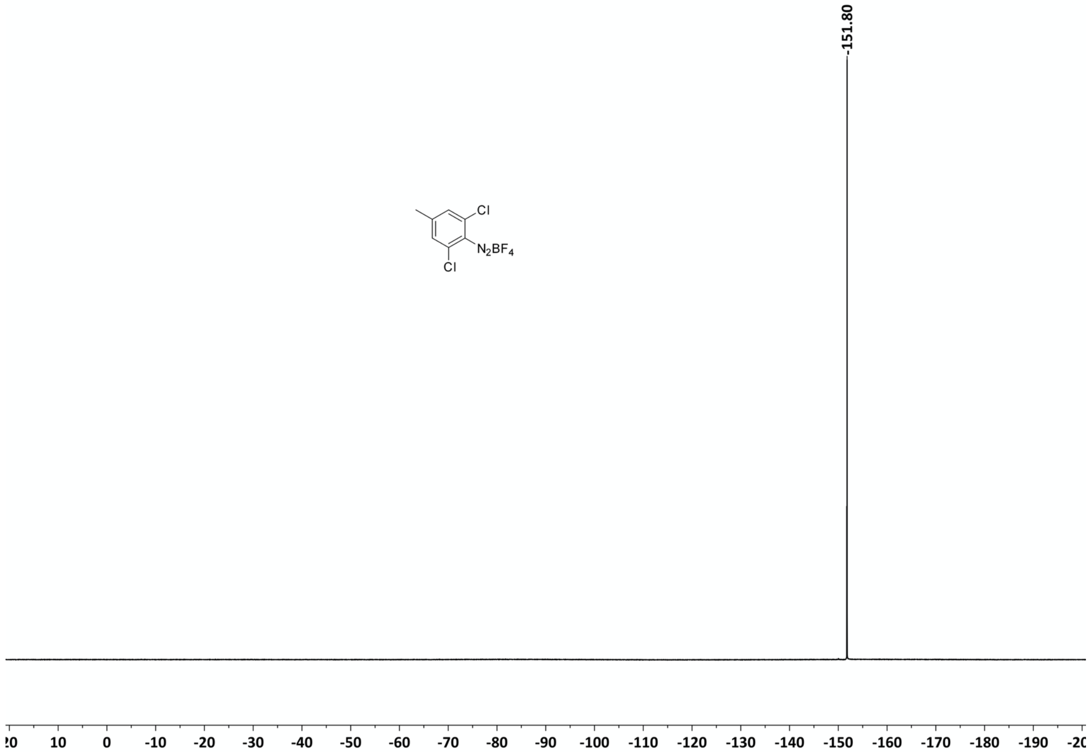

Figure 128. ^{19}F NMR spectrum of 2,6-dichloro-4-methylbenzenediazonium tetrafluoroborate.

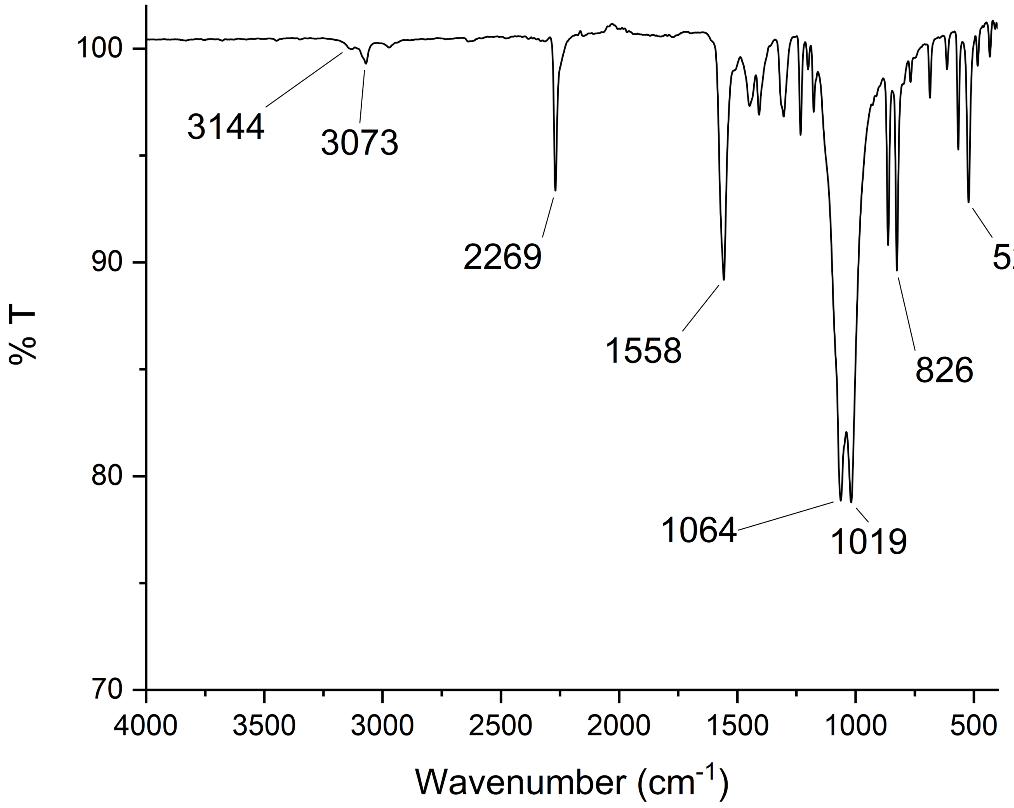

Figure 129. FT-IR of 2,6-dichloro-4-methylbenzenediazonium tetrafluoroborate.

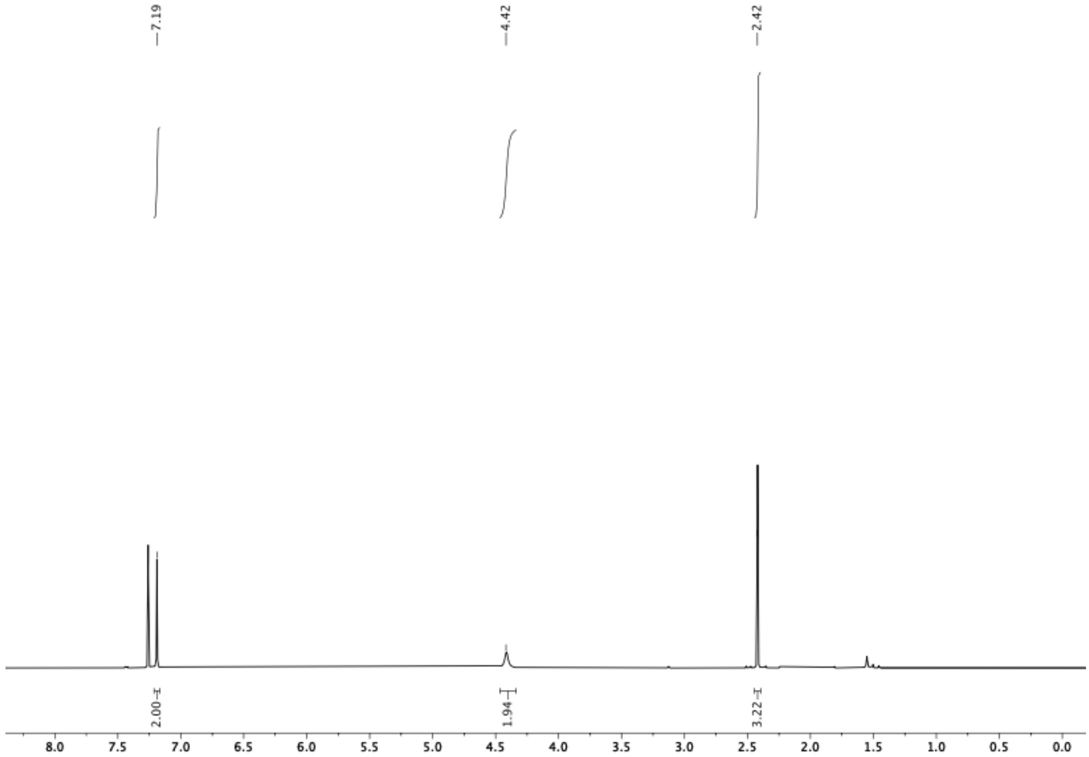

Figure 130. ^{1}HNMR spectrum of 2,6-dichloro-4-(methylthio)aniline.

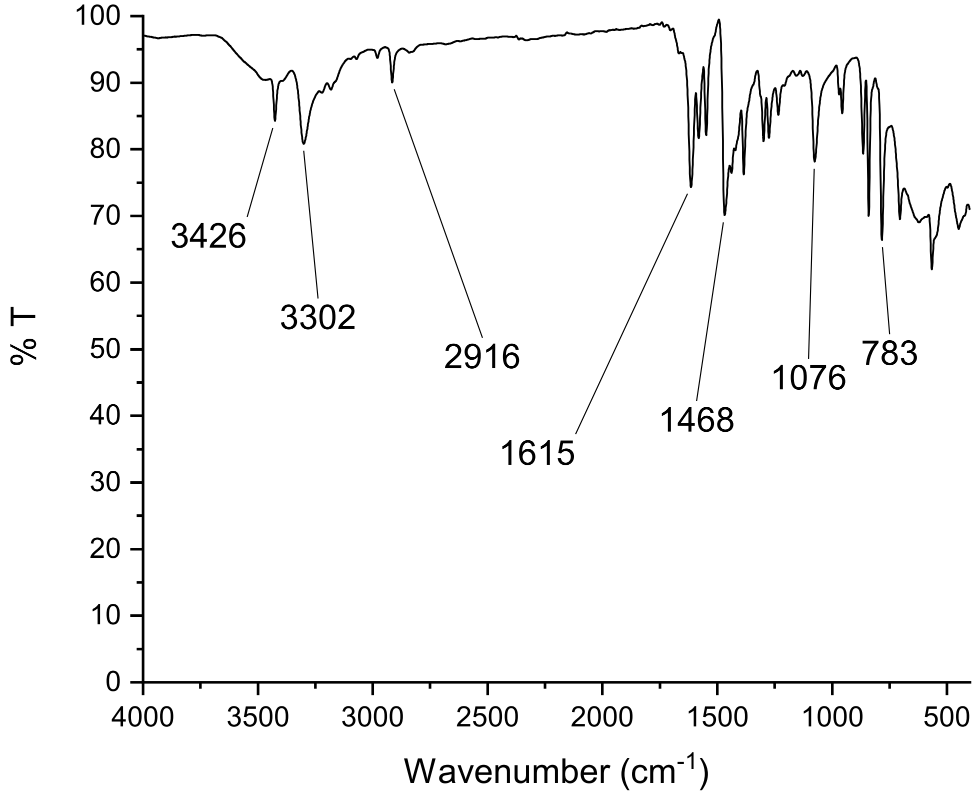

Figure 131. FT-IR spectrum of 2,6-dichloro-4-(methylthio)aniline.

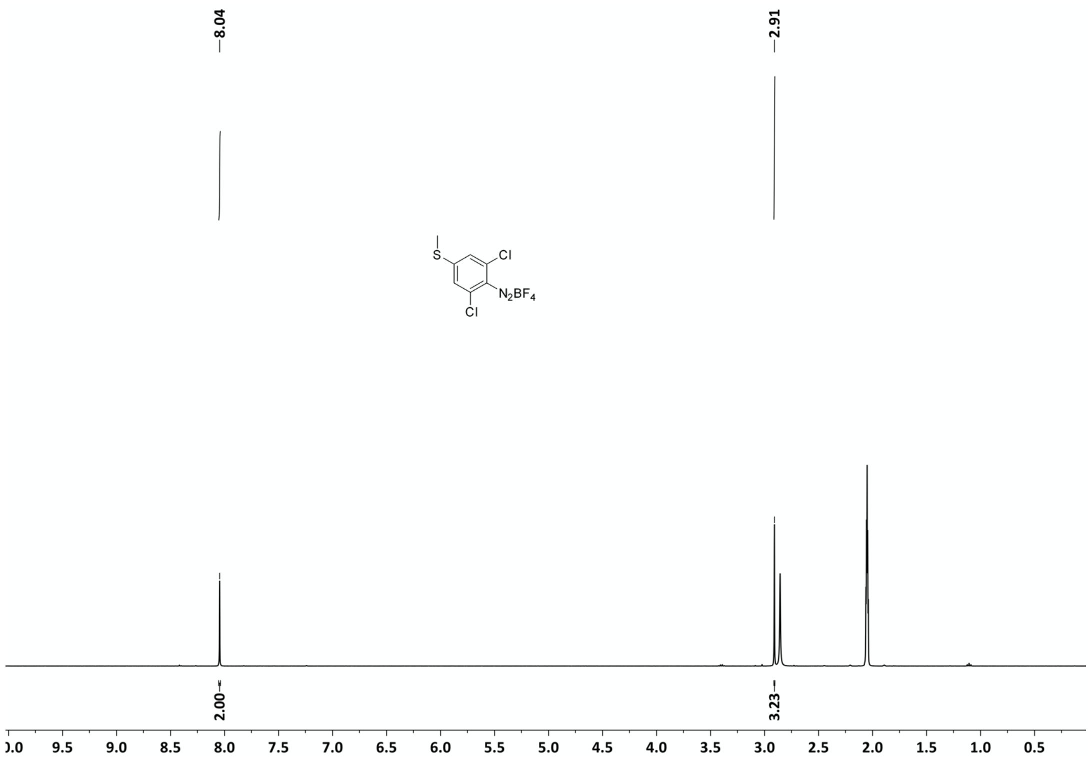

Figure 132. ^{1}HNMR spectrum of 2,6-dichloro-4-(methylthio)benzenediazonium tetrafluoroborate.

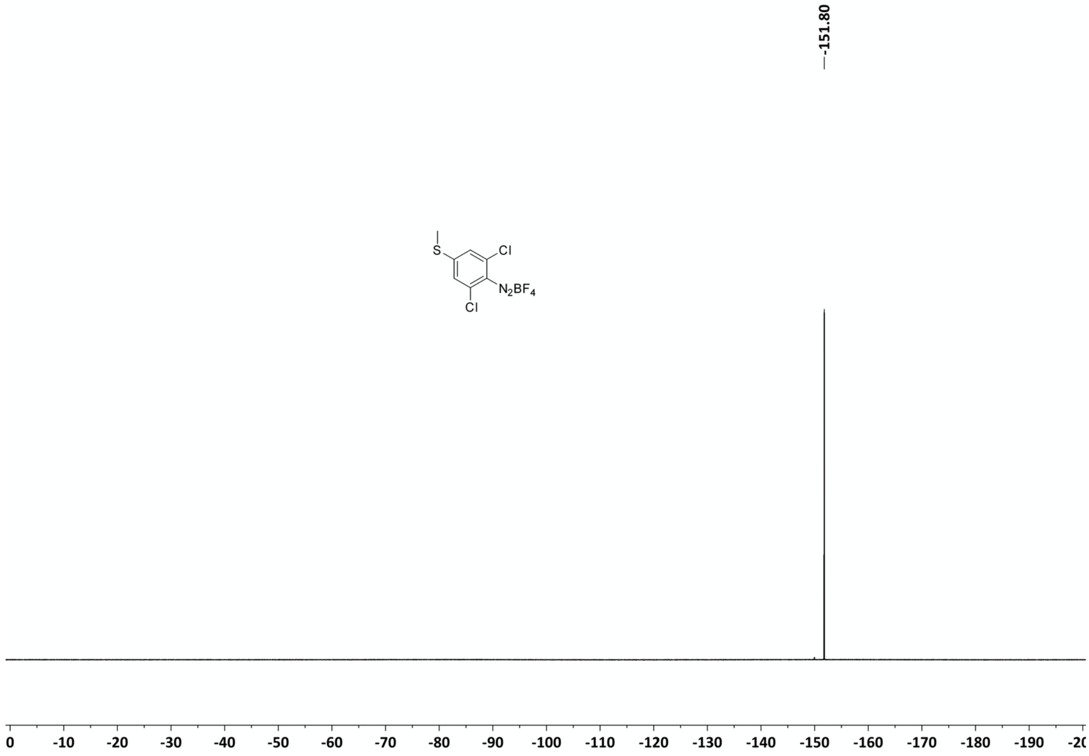

Figure 133.^{19}F NMRspectrum of 2,6-dichloro-4-(methylthio)benzenediazonium tetrafluoroborate.

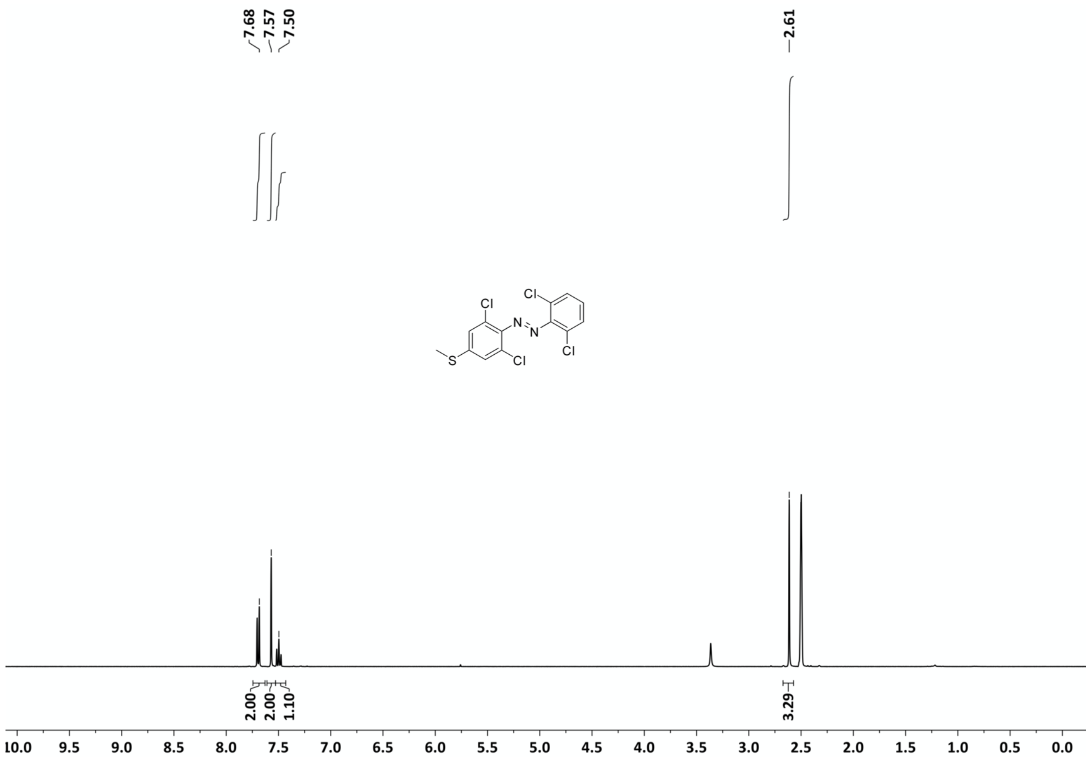

Figure 134. ^{1}HNMR spectrum of **5**.

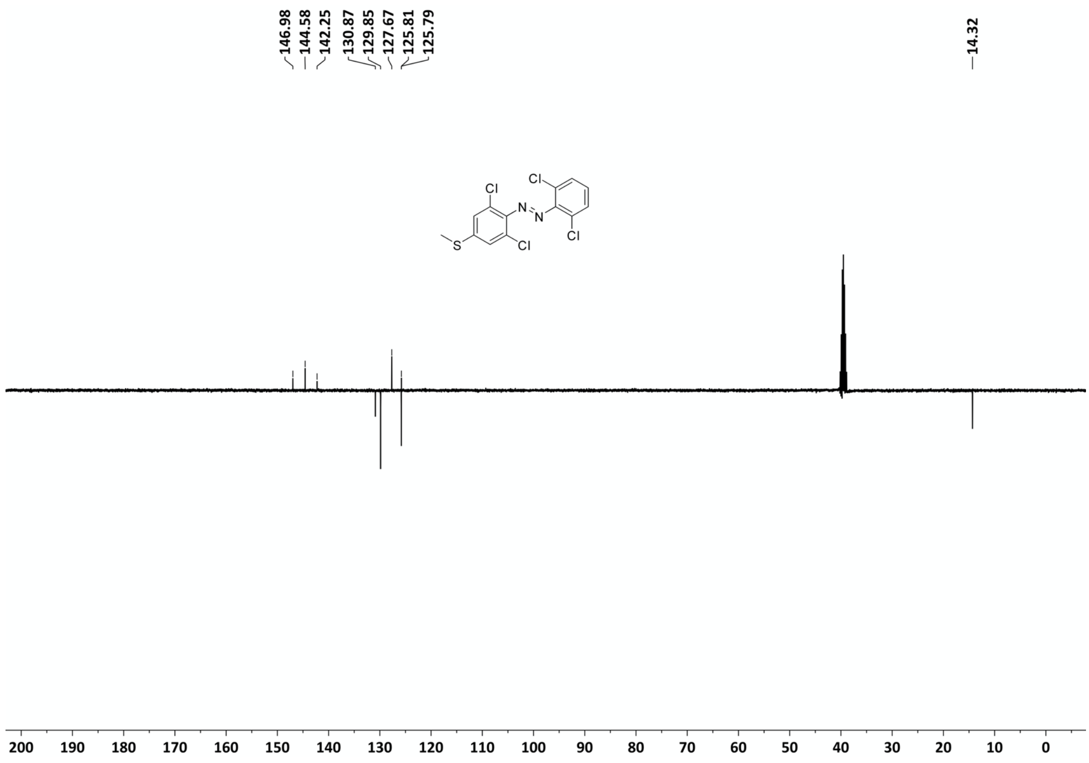

Figure 135. ^{13}C APT spectrum of 5.

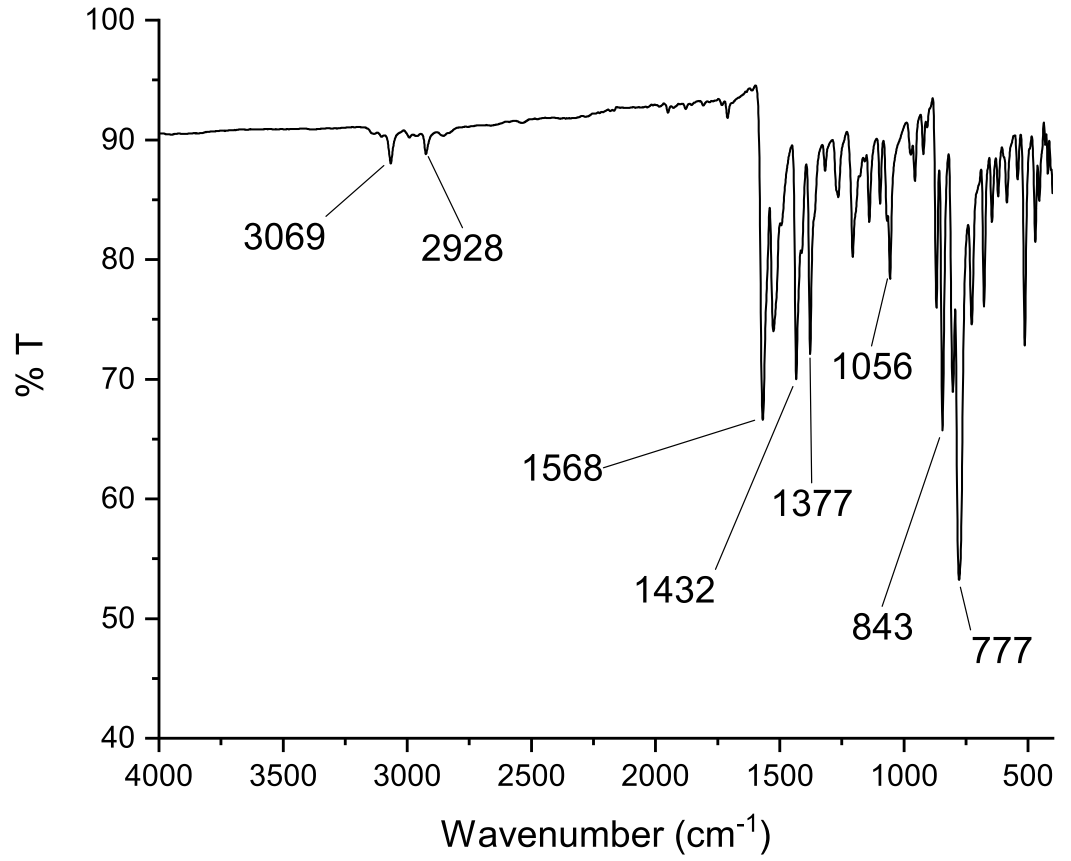

Figure 136. FT-IR spectrum of 5.

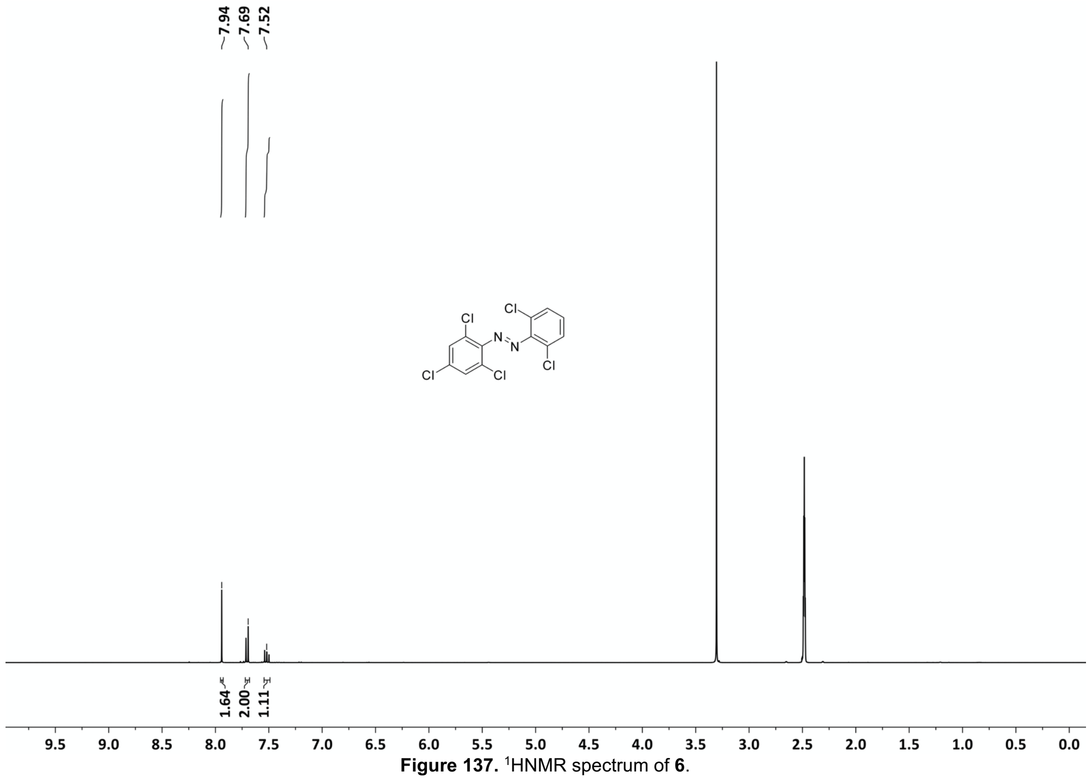

Figure 137. ^{1}HNMR spectrum of **6**.

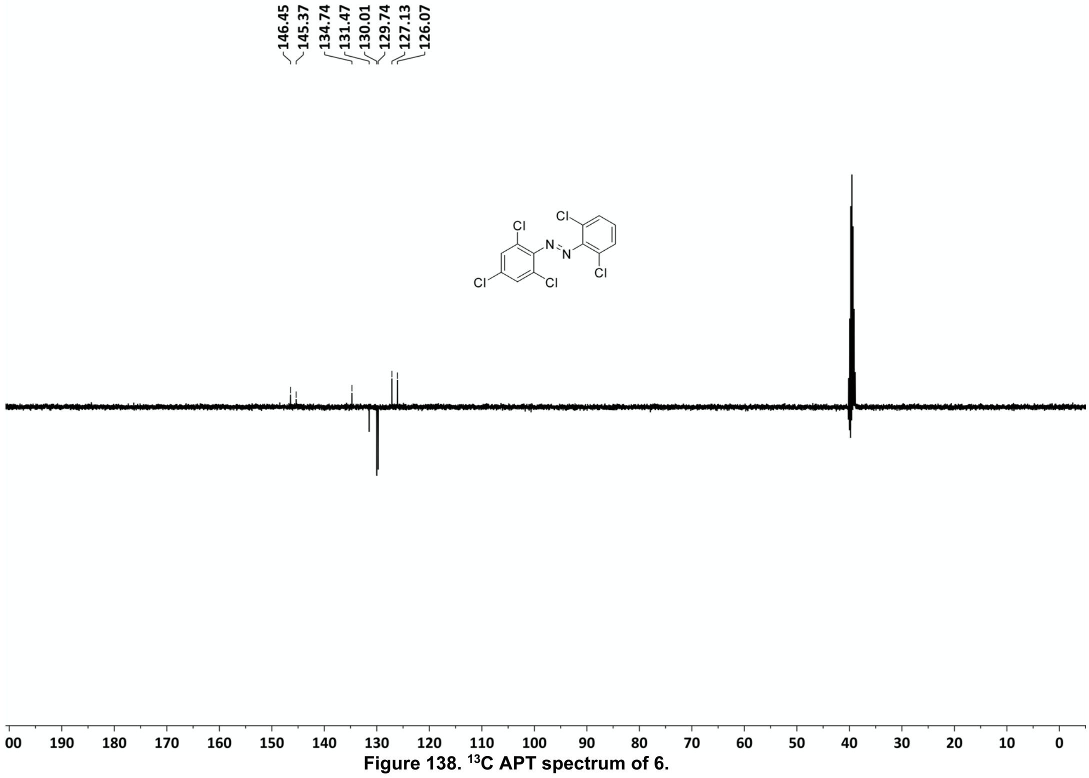

Figure 138. ^{13}C APT spectrum of 6.

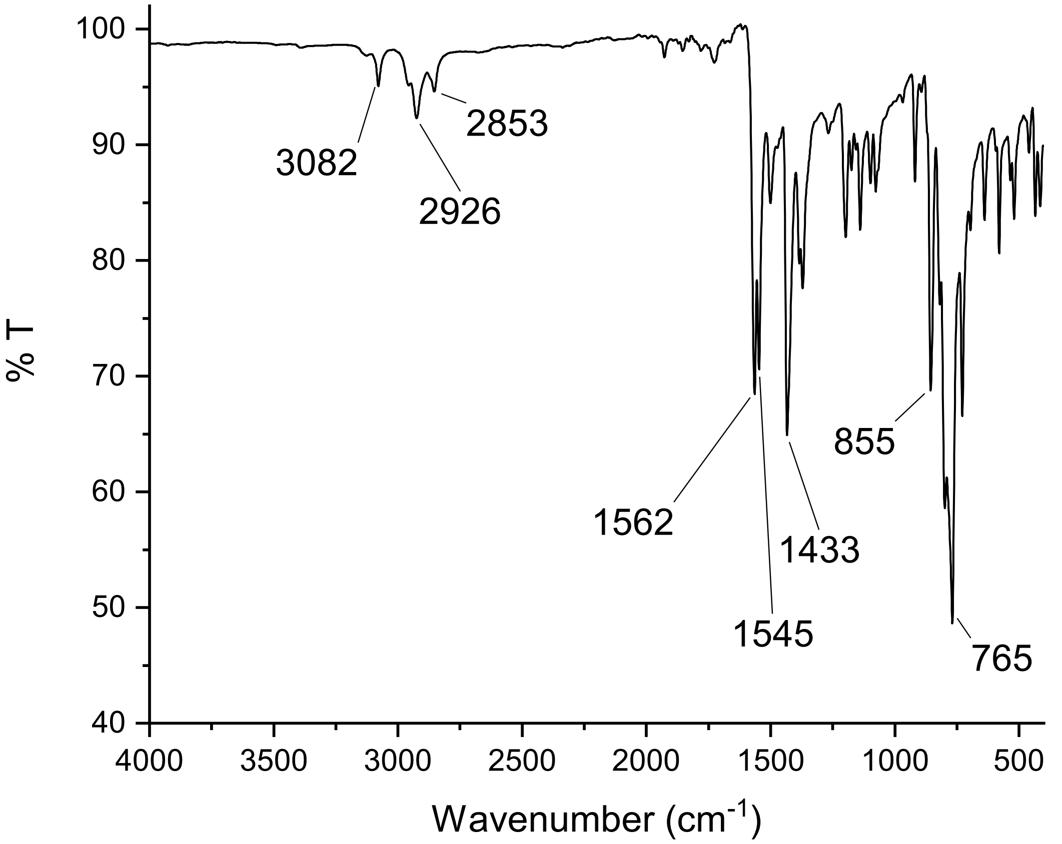

Figure 139. FT-IR spectrum of 6.

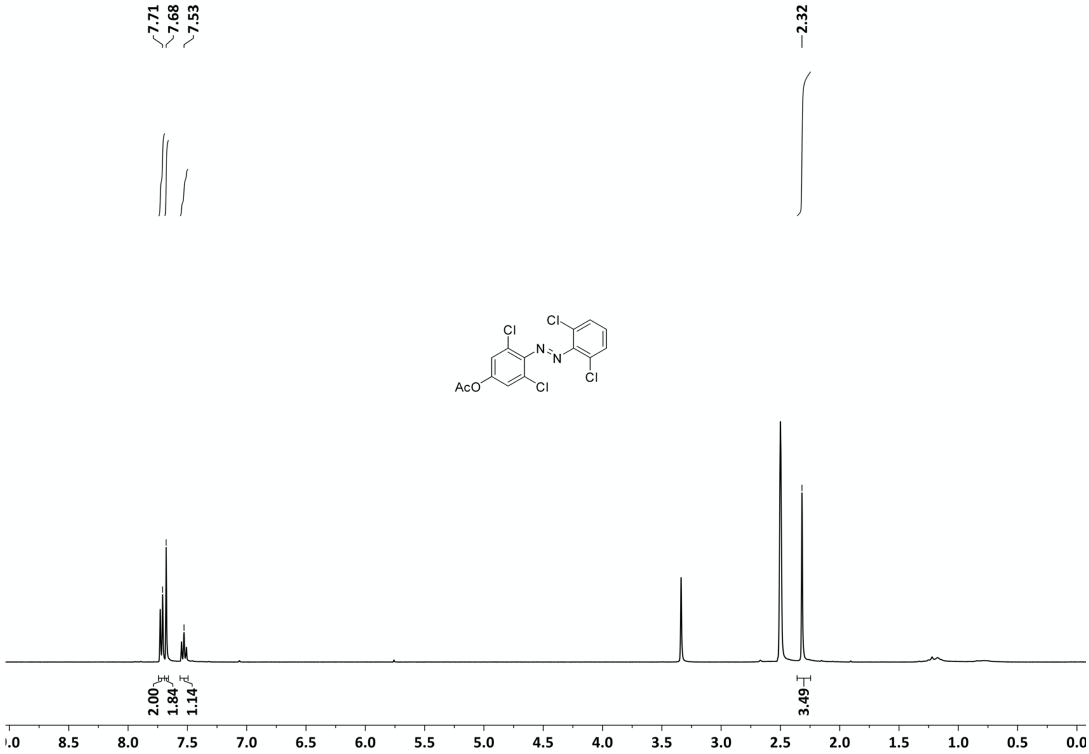

Figure 140. ^{1}HNMR spectrum of **7**.

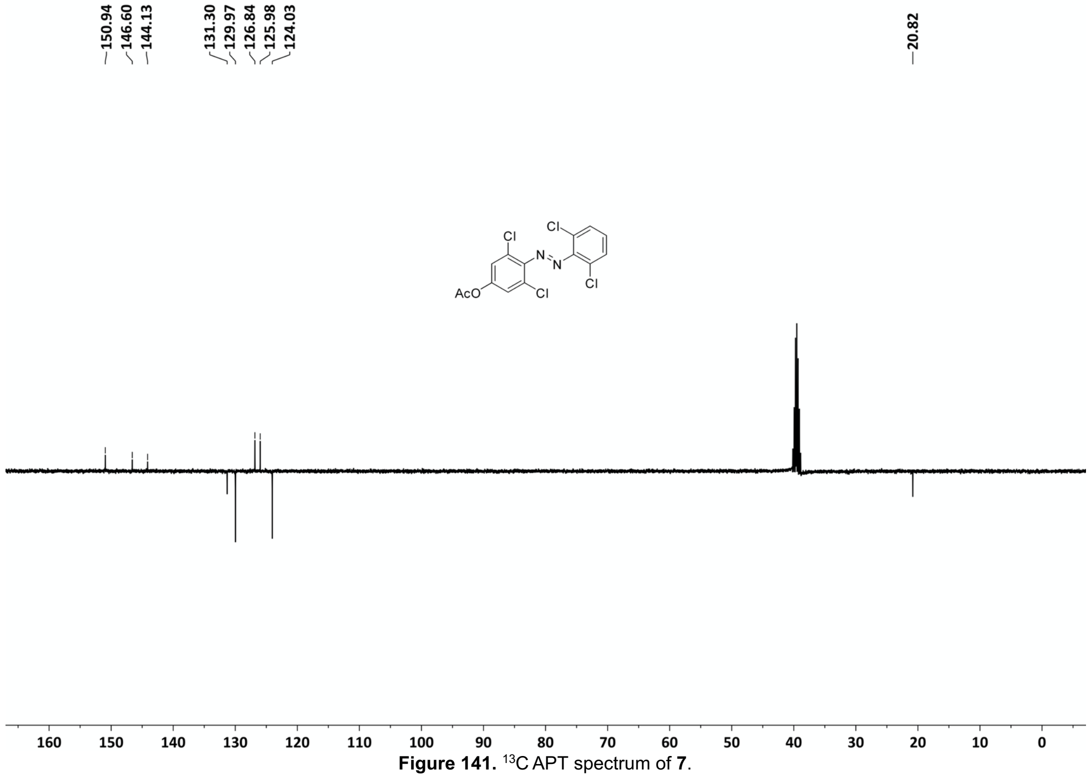

Figure 141. ^{13}C APT spectrum of **7**.

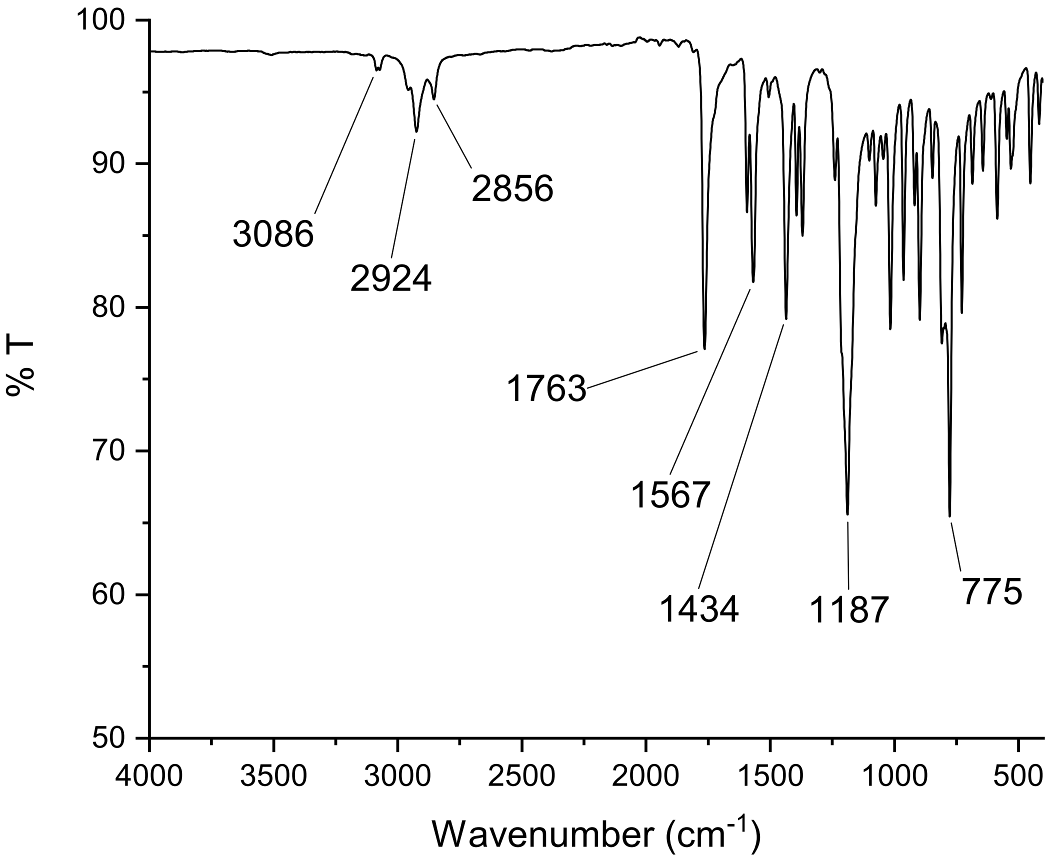

Figure 142. FT-IR spectrum of **7**.

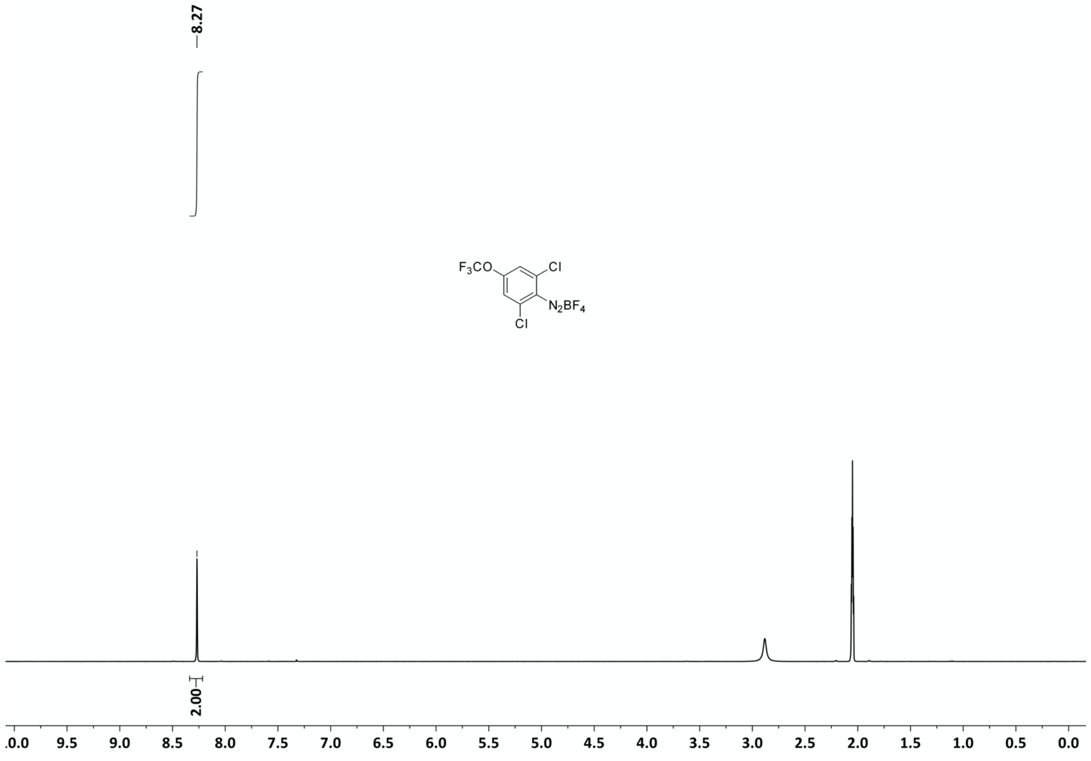

Figure 143. ^{1}HNMR spectrum of 2,6-dichloro-4-(trifluoromethoxy)benzenediazonium tetrafluoroborate.

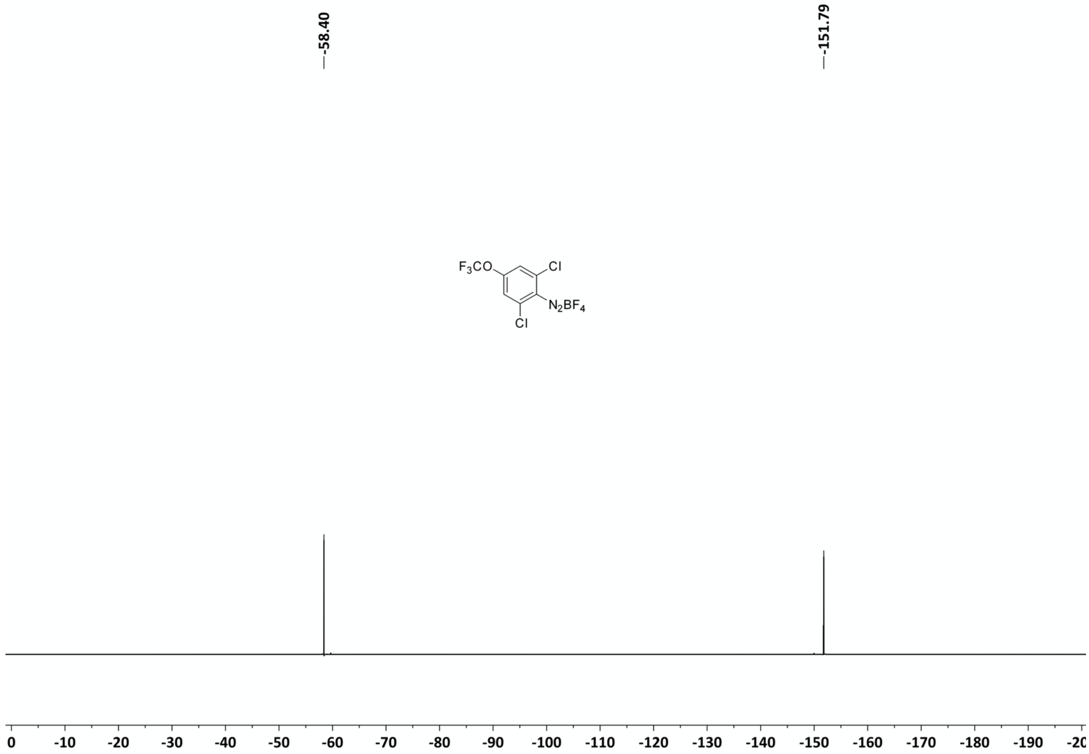

Figure 144. ^{19}F NMR spectrum of 2,6-dichloro-4-(trifluoromethoxy)benzenediazonium tetrafluoroborate.

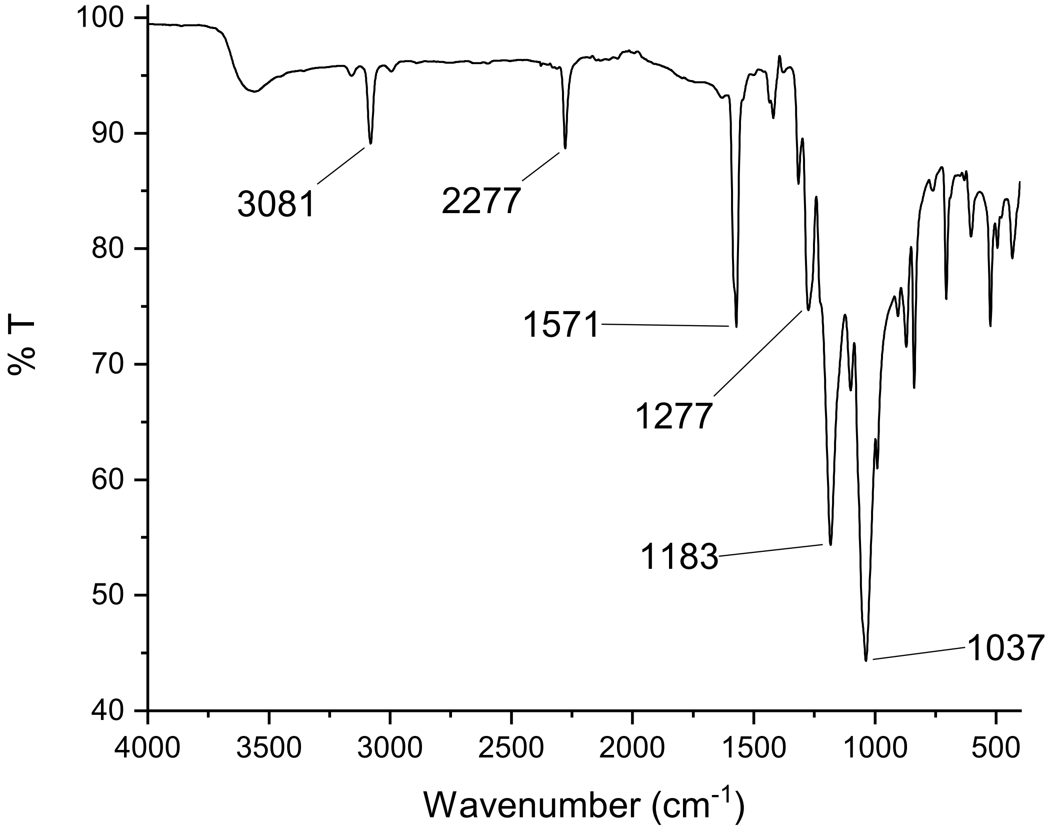

Figure 145. FT-IR of 2,6-dichloro-4-(trifluoromethoxy)benzenediazonium tetrafluoroborate.

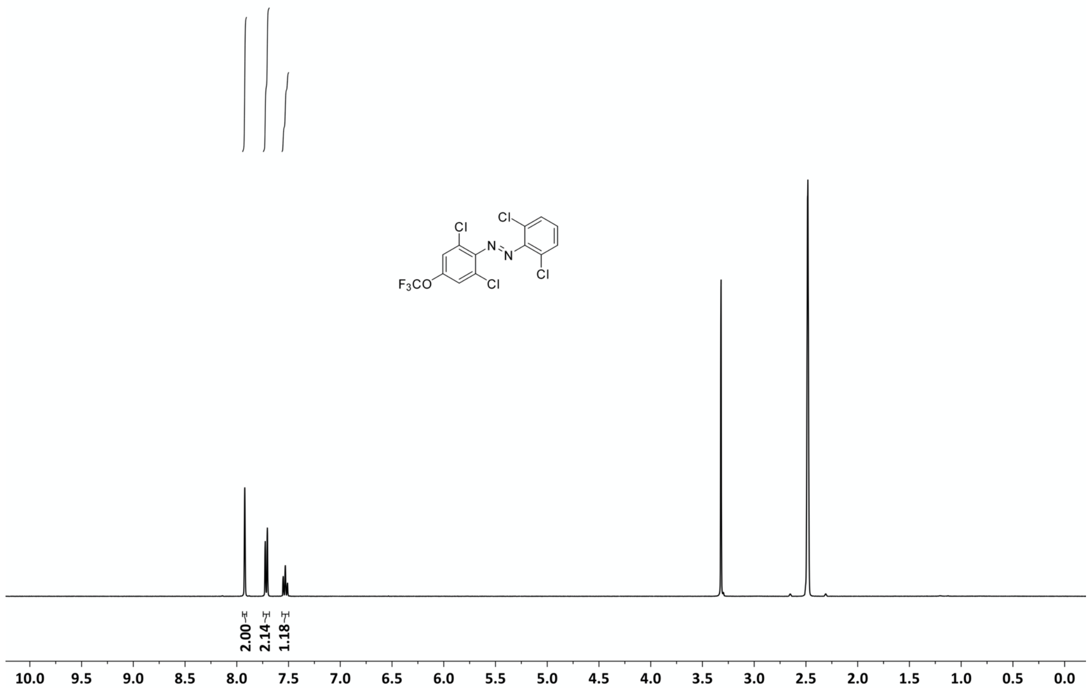

Figure 146. ^{1}HNMR spectrum of 8.

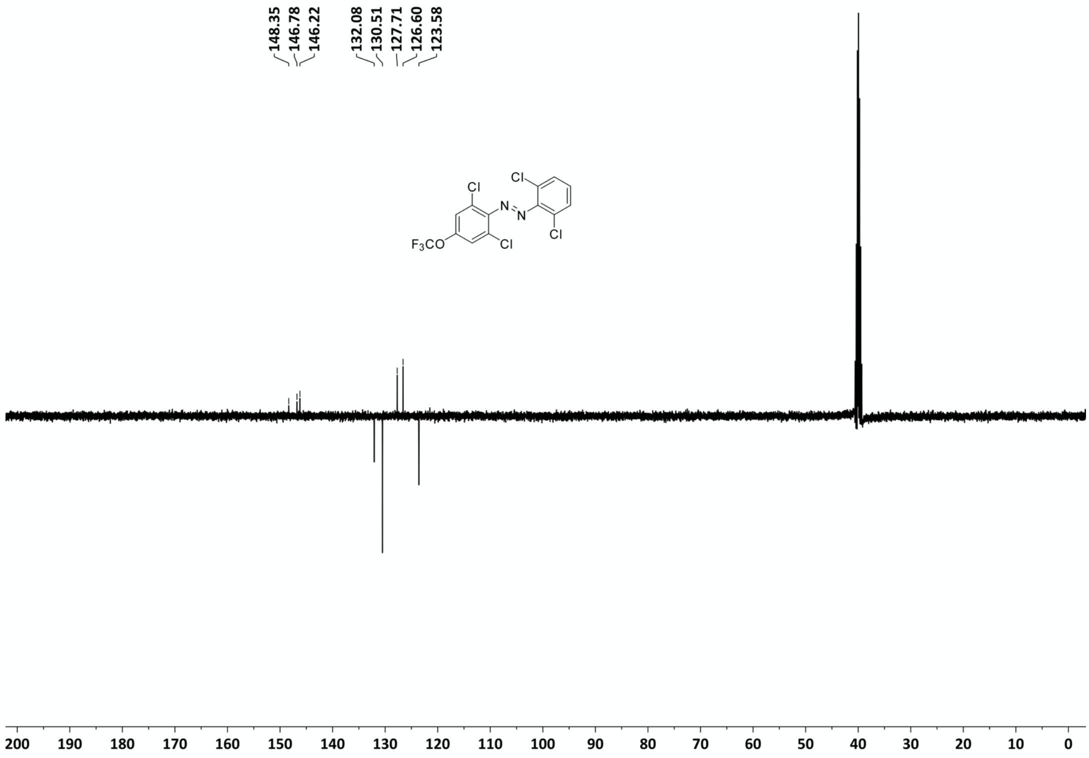

Figure 147. ^{13}C APT spectrum of **8**.

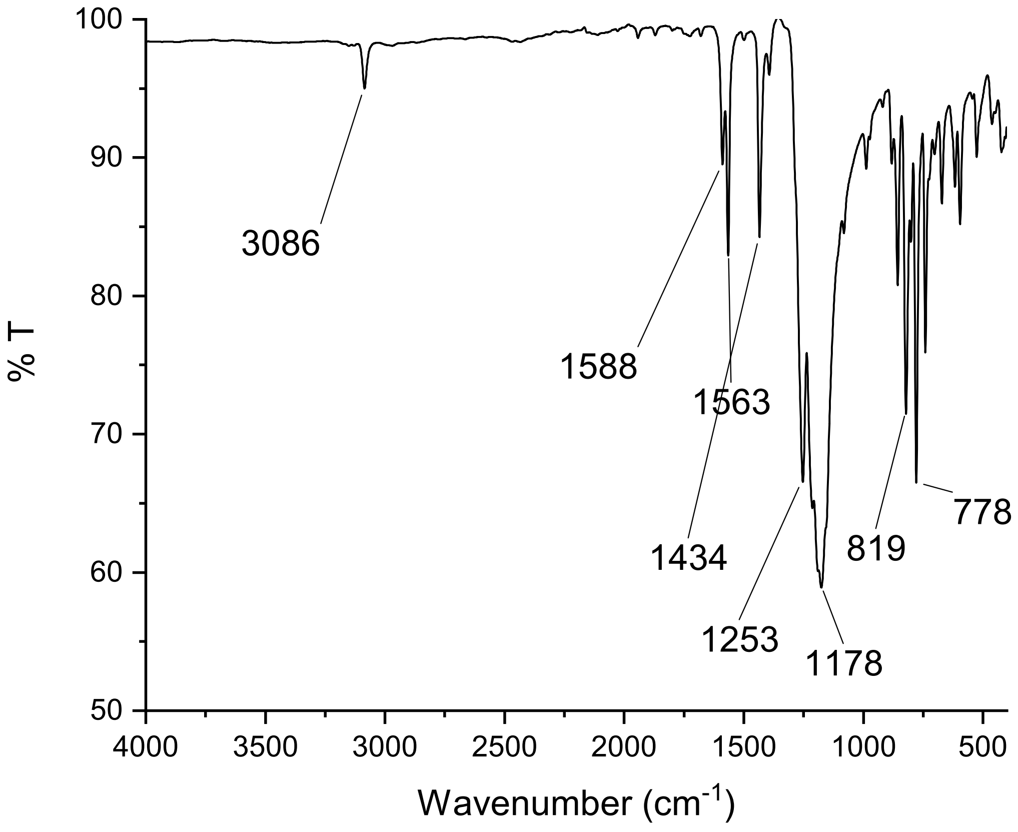

Figure 148. FT-IR spectrum of **8**.

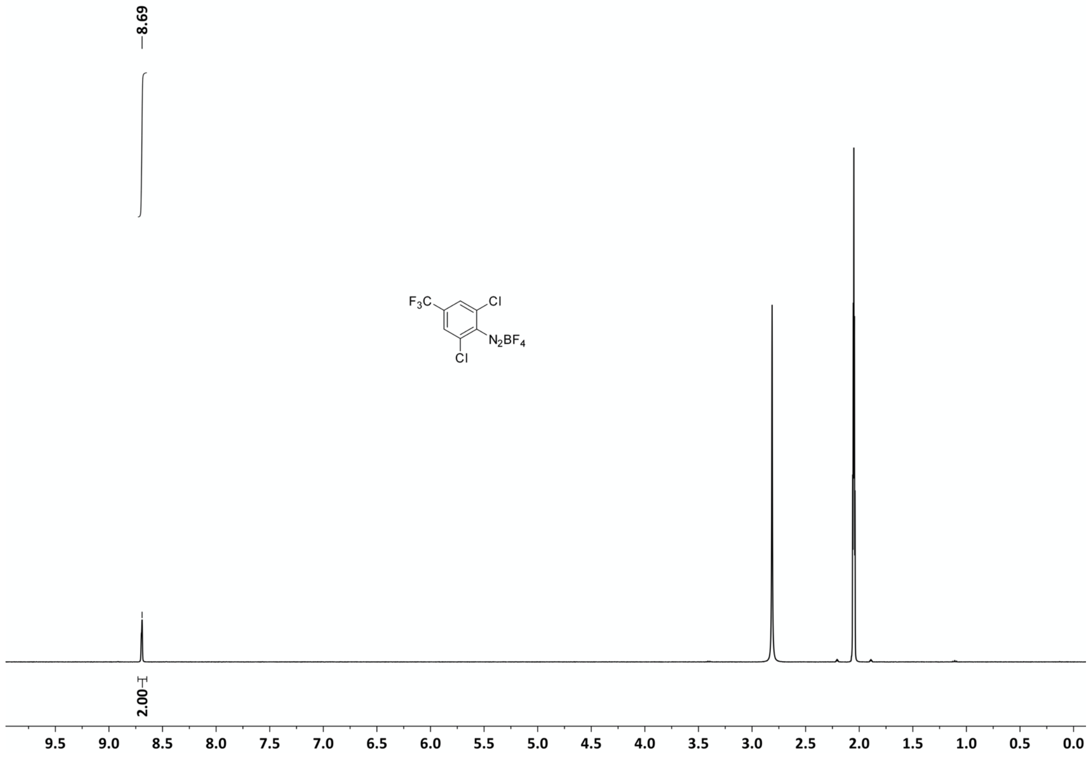

Figure 149. ^{1}HNMR spectrum of 2,6-dichloro-4-(trifluoromethyl)benzenediazonium tetrafluoroborate.

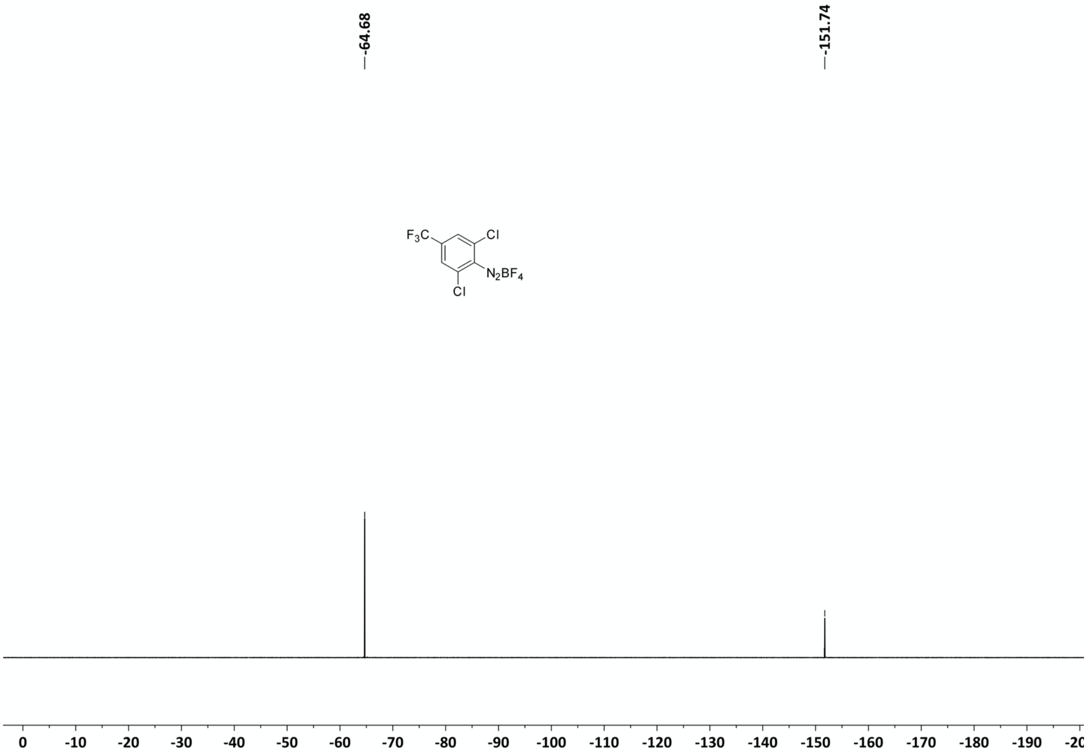

Figure 150. ^{19}F NMR of 2,6-dichloro-4-(trifluoromethyl)benzenediazonium tetrafluoroborate.

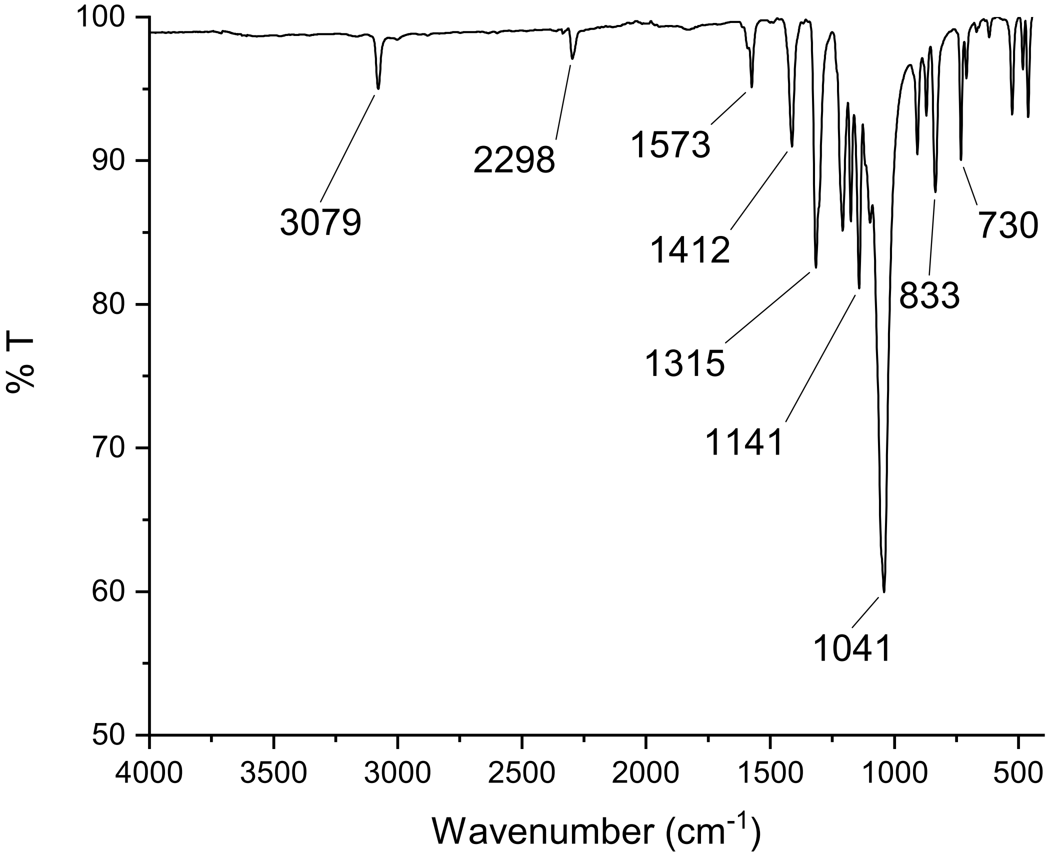

Figure 151. FT-IR of of 2,6-dichloro-4-(trifluoromethyl)benzenediazonium tetrafluoroborate.

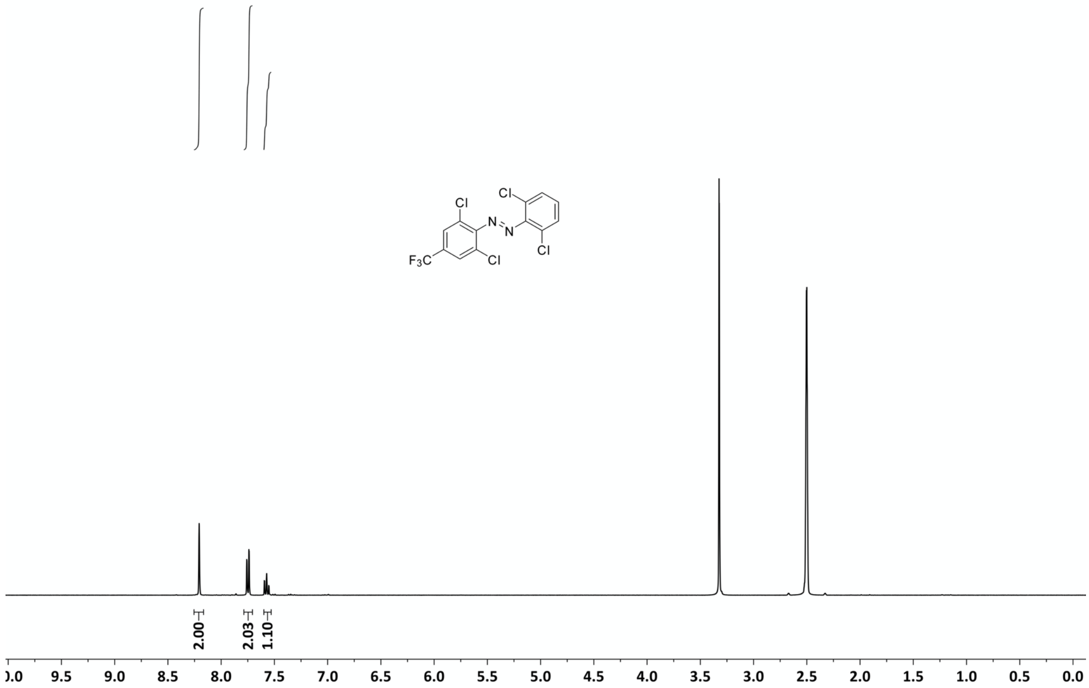

Figure 152. ^{1}HNMR spectrum of **9**.

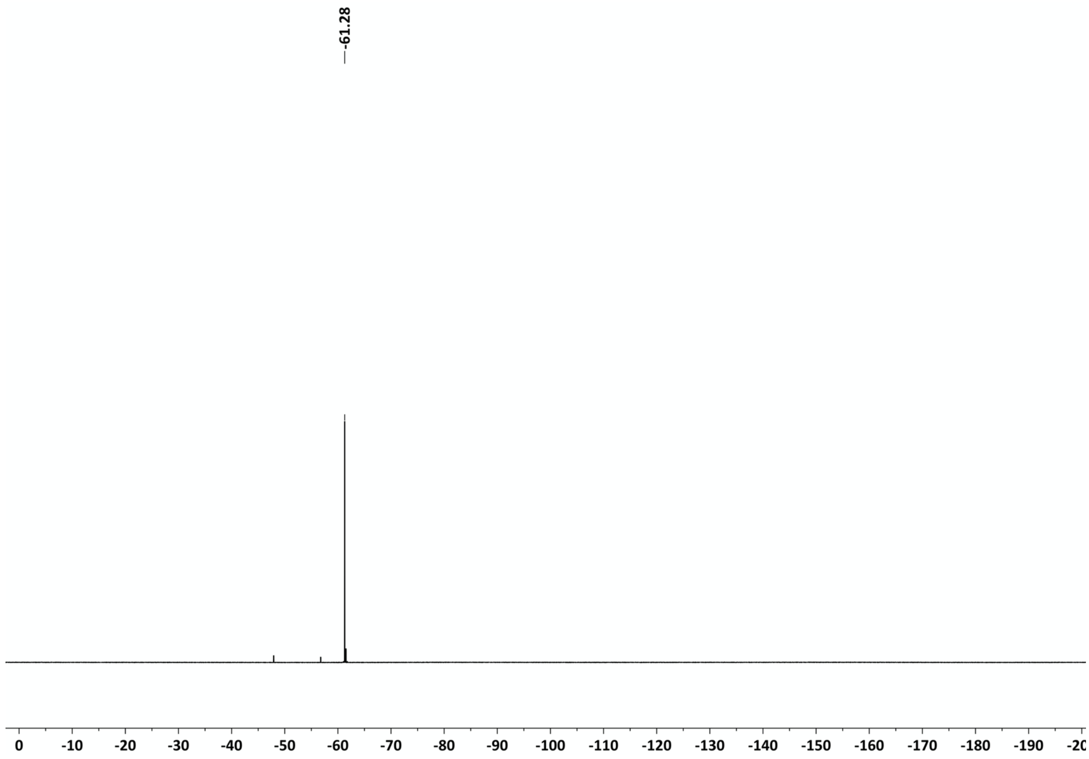

Figure 153. ^{19}F NMR spectrum of **9**.

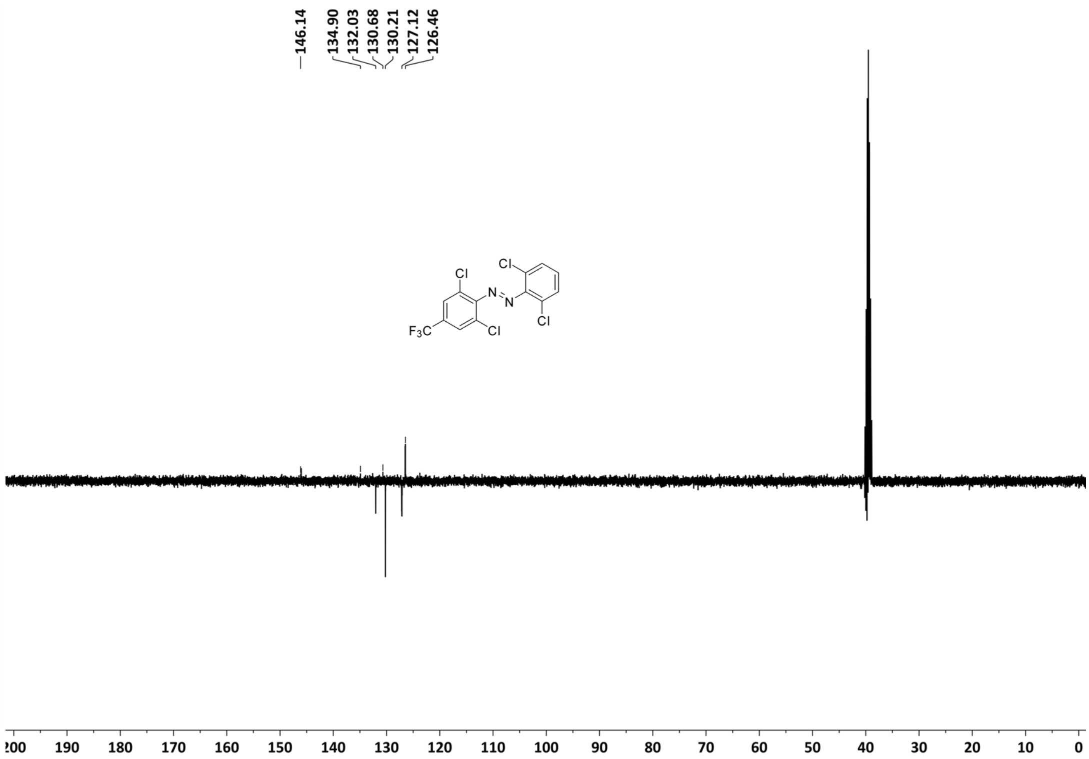

Figure 154. ^{13}C APT spectrum of **9**.

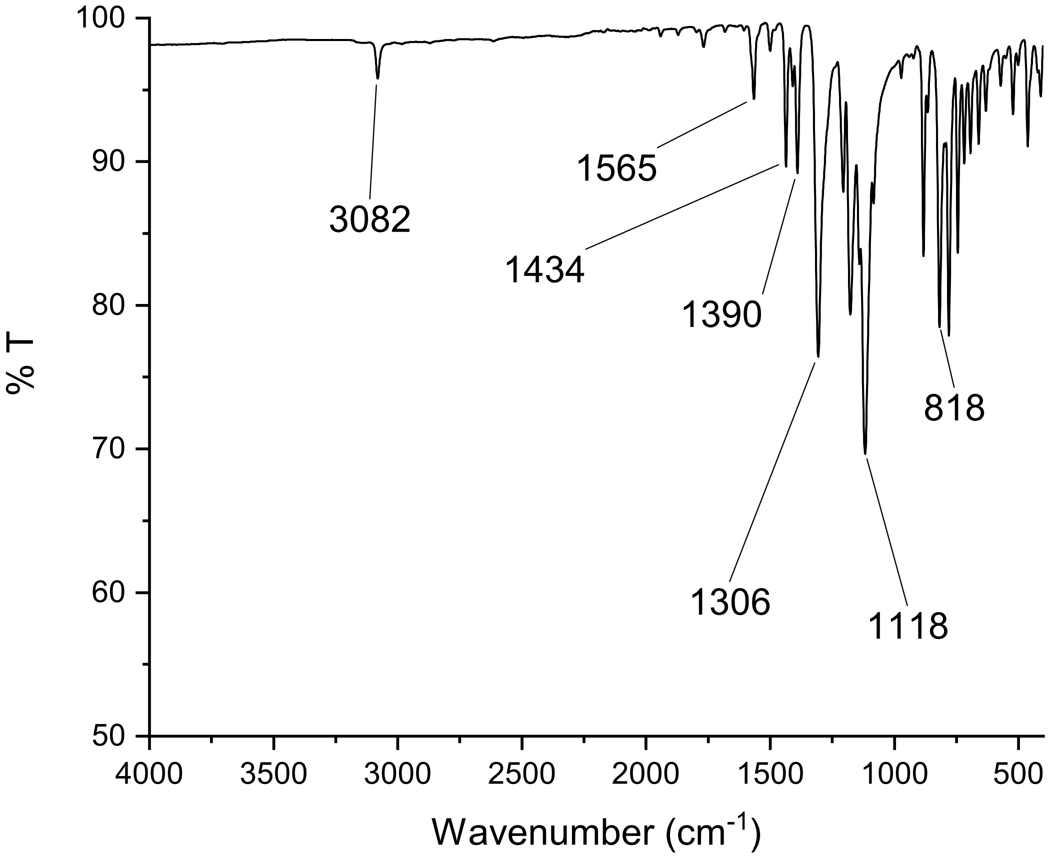

Figure 155. FT-IR spectrum of **9**.

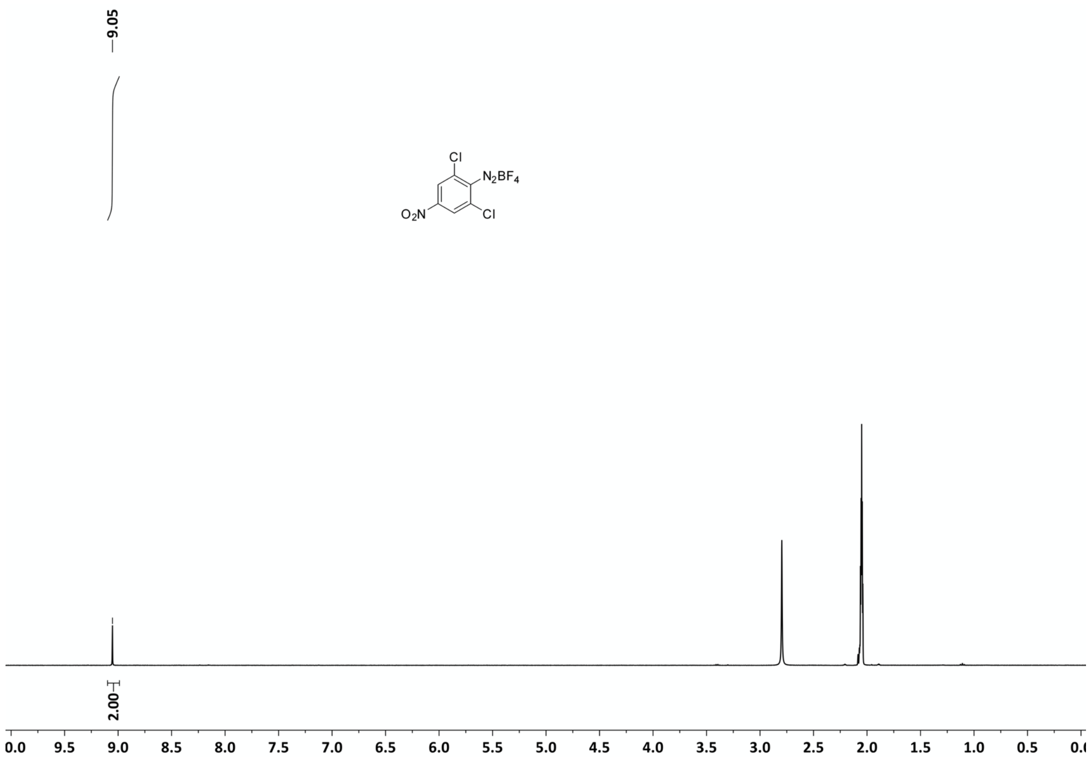

Figure 156. ^{1}HNMR spectrum of 2,6-dichloro-4-(nitro)benzenediazonium tetrafluoroborate.

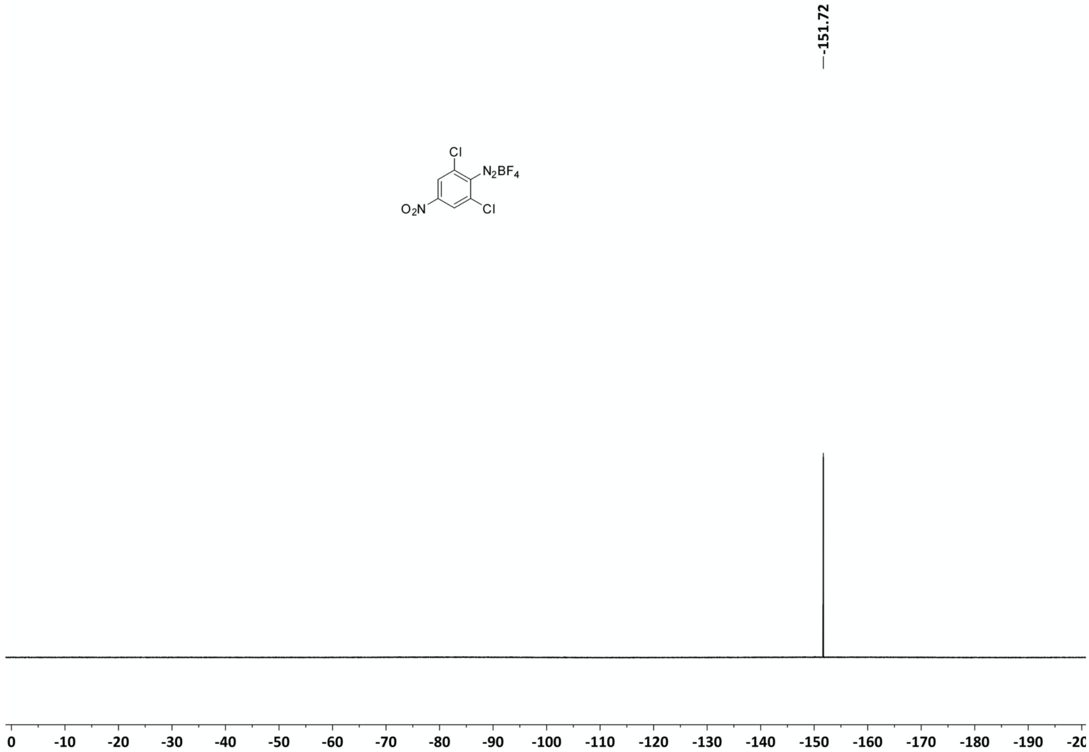

Figure 157. ^{19}F NMR of 2,6-dichloro-4-(nitro)benzenediazonium tetrafluoroborate.

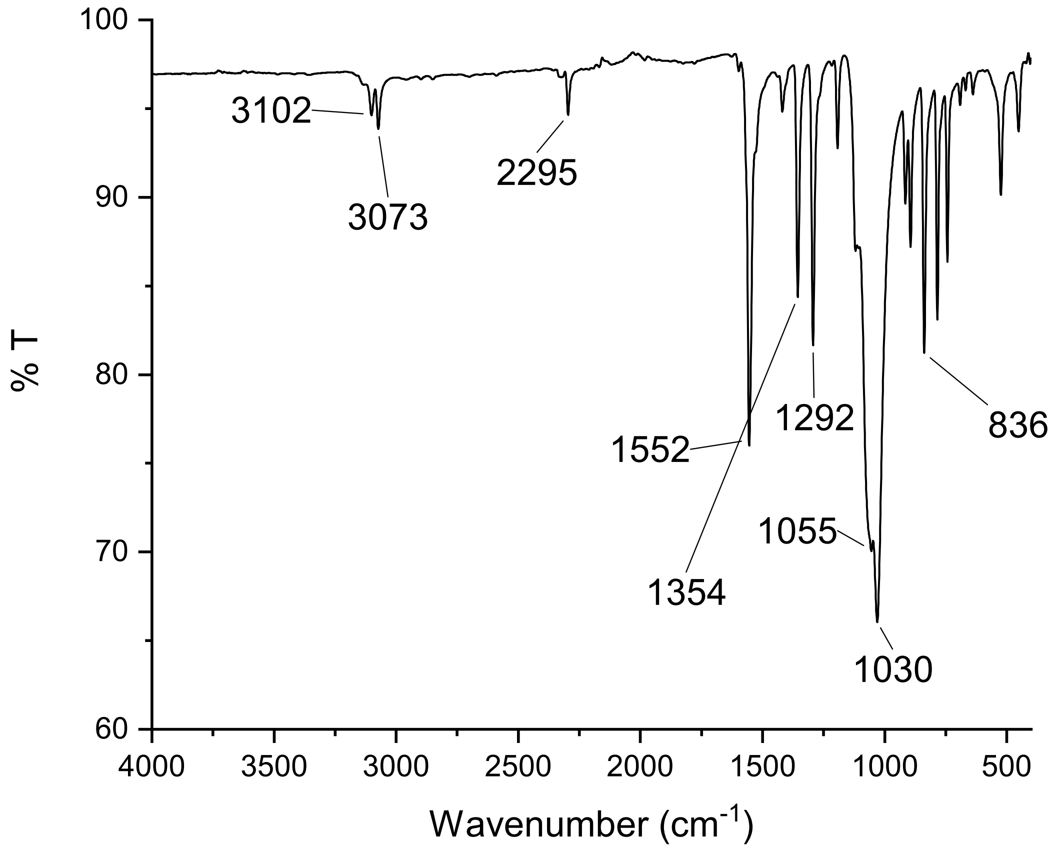

Figure 158. FT-IR of 2,6-dichloro-4-(nitro)benzenediazonium tetrafluoroborate.

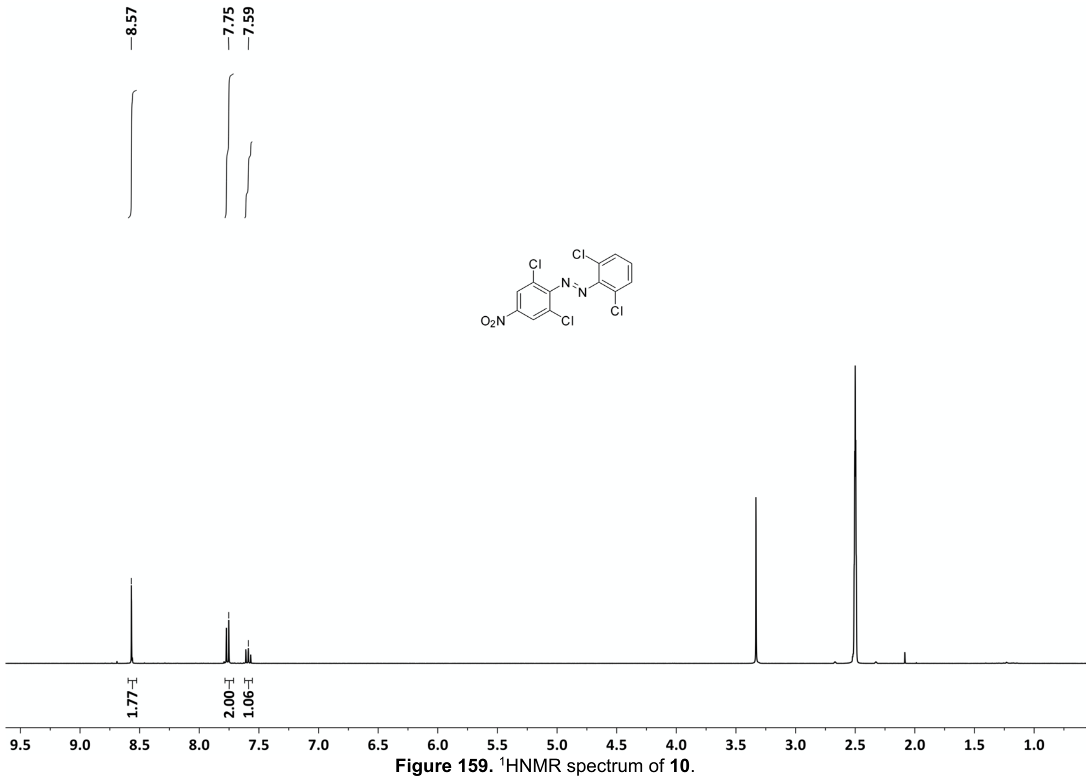

Figure 159. ^{1}HNMR spectrum of **10**.

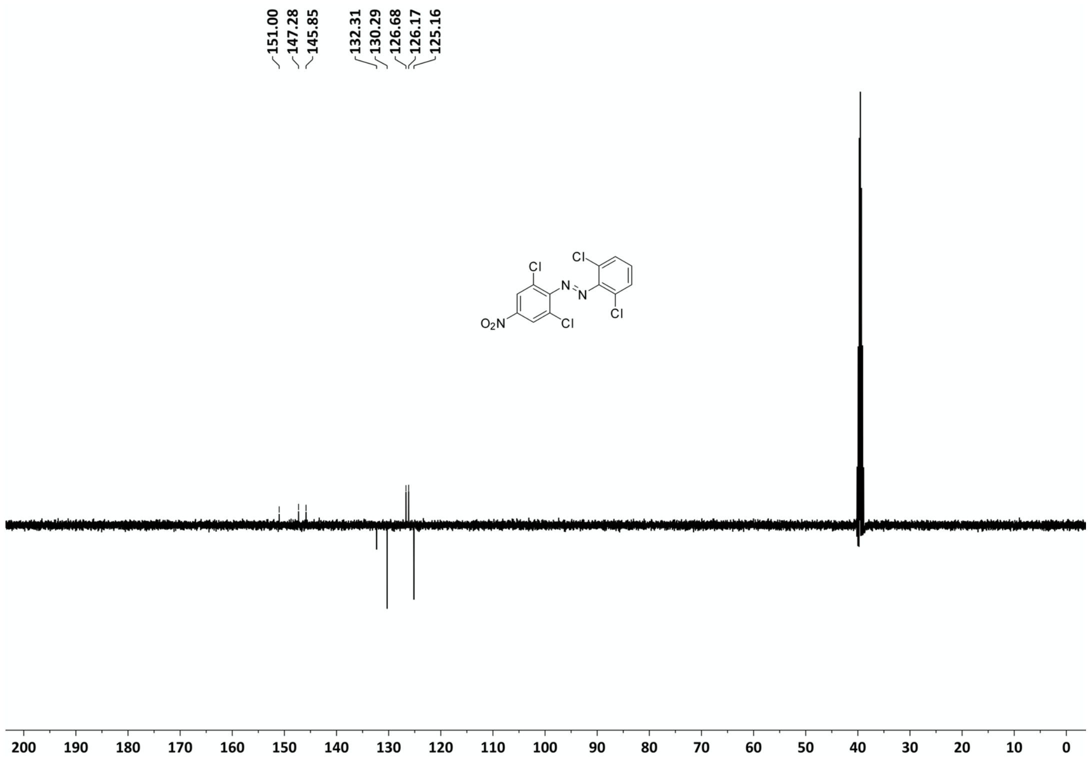

Figure 160. ^{13}C APT spectrum of **10**.

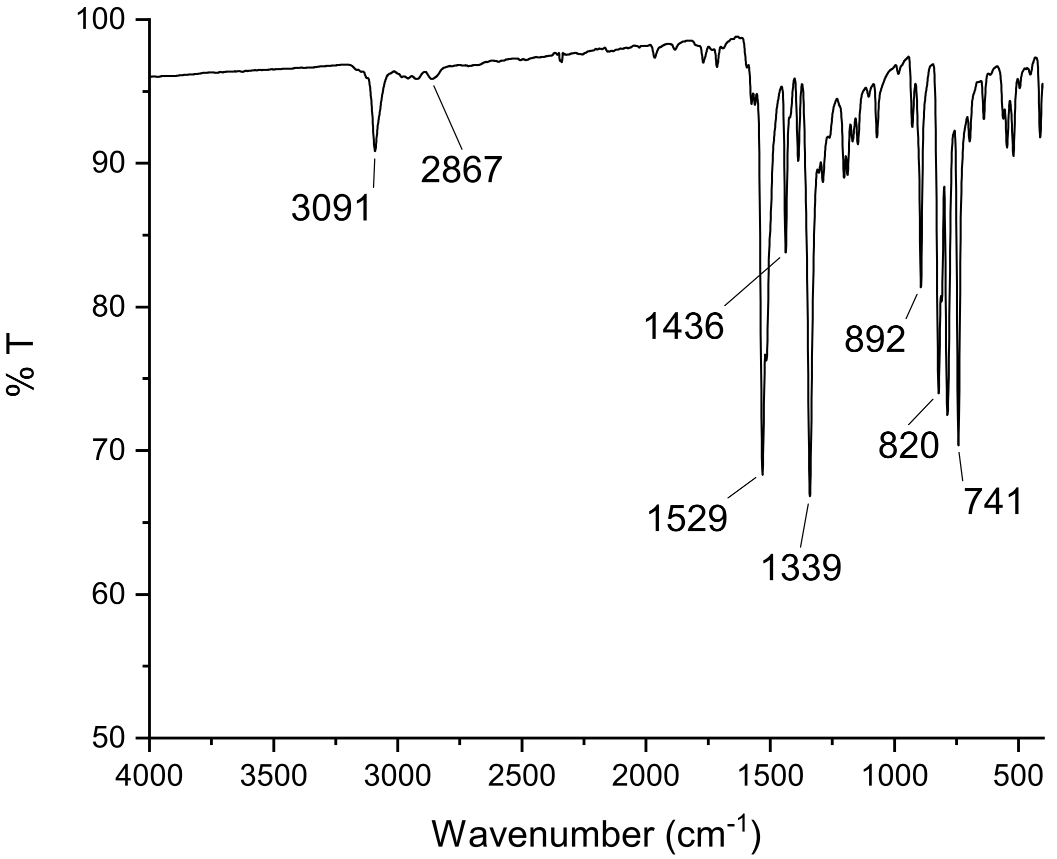

Figure 161. FT-IR spectrum of **10**.

Theoretical calculations

Computational details. To perform our simulations, we use the Gaussian16 program.[5] The *ab initio* simulations consist in: i) DFT geometry optimizations of the S_0 structures in their *E* and *Z* forms; ii) calculations of the thermal transition states, considering the well-known inversion and rotation pathways, the latter being determined in the broken-symmetry (BS) approach; iii) calculations of the vibrational frequencies on the structures optimized in the first two steps; iv) subsequent vertical TD-DFT calculations of the singlet states on the ground-state structures. We apply default procedures, integration grids, algorithms and parameters, except for tighten energy (typically 10^{-10} *a.u.*) convergence threshold and the use of the *ultrafine* integration DFT grid at all steps (including CPKS). All calculations are performed with the PBE0 exchange-correlation functional, that is free of empirical parameters.[6] The optimization and vibrational calculations use the 6-311+G(d) atomic basis set and account for dispersion effect at the $D3^{BJ}$ level, whereas the vertical TD-DFT calculations rely on the 6-311++G(2d,p) basis set. During all steps,[7] a modelling of bulk solvent effects (DMSO) through the Polarizable Continuum Model (PCM).[8] For the TD-DFT part, the transition energies have been obtained using the LR+cLR approach,[9] an approach that accounts for both linear-response and state-specific solvation effects. Our LR+cLR TD-DFT total energies are therefore obtained as:

$$E^{LR+cLR} = E^{LR} + E^{cLR} - E^{\omega 0}$$

In which $E^{\omega 0}$ is the "gas-phase like" total excites-state energy obtained with solvated orbitals. When subtracting the ground-state energy from that LR+cLR energy, one obtains the corresponding transition energy. The TD-DFT results have been corrected by CC2 gas-phase calculations used to shift the TD-DFT response, so that we simply used:

$$\Delta E^{CC2\text{-}corr} = \Delta E^{CC2\text{-}gas} + \Delta E^{TD\text{-}LR+cLR} - \Delta E^{TD\text{-}gas}$$

in which one can approximate $\Delta E^{TD\text{-}gas}$ as $\Delta E^{\omega 0}$. The CC2 calculations were made with Turbomole.[10]

Additional results for 4.

The optimized structures of **1** are represented in **Fig. 162**. Due to the strong steric effect of the chlorine atoms, the *E* isomer is non planar (C_2 point group) with dihedral angles of 5.4° and 48.8° for the f^{CNNC} and f^{NNCC} dihedral angles, respectively. In the *Z* form, these angles are 4.8° and 58.7° respectively (see **Fig. 162**). The *Z* form is less stable by 6.29 kcal.mol^{-1} on the free energy scale than the *E* form.

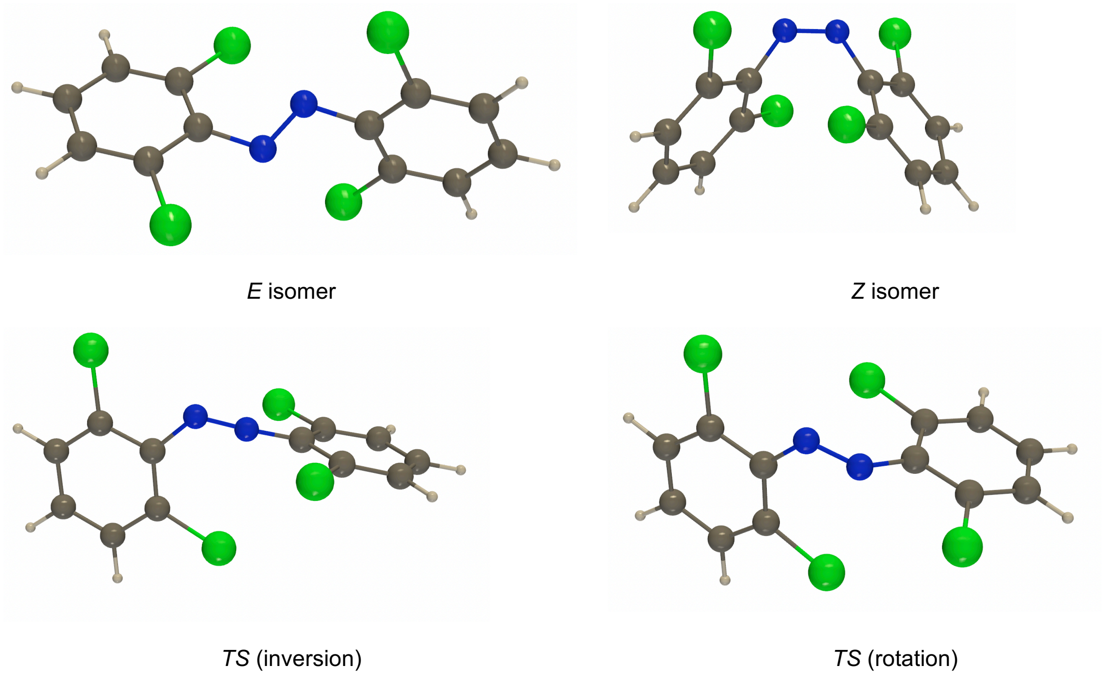

Figure 162. Representation of the different *minimal* structures for compound **4** (R=H)

We have located both the inversion and rotation transition states, the former using standard (restricted) DFT, whereas the second that implies rotation around a double bond was modelled using BS-DFT, with a spin density showing the expected topology (see **Fig. 163**). The rotation and inversion transition states present frequencies of 389*i* cm^{-1} and 435*i* cm^{-1}, respectively, with the expected atom displacements. The relative free energies with respect to the *Z* (*E*) form are 71.3 (97.6) kJ.mol^{-1} and 101.4 (127.7) kJ.mol^{-1} for the rotation and inversion, respectively, indicating that the thermal back reaction should occur through a rotational process.

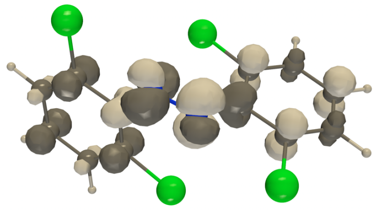

Figure 163. Representation of the spin density for the BS-DFT solution of the *TS* (rotation). Representation threshold 10^{-2} au.

The TD-DFT main transitions computed for **4** in its *E* form are 495 nm (*f*=0.053) and 303 nm (*f*=0.409), which correspond to the 457 and 284 nm absorption experimentally. For the lowest transition, the CC2-corrected value is 466 nm, very close from the experimental value. The states are represented in **Fig 164**. Due to the strong non-planarity of the system, the usual clear n-π* and π-π* character are lost, especially for the second transition. In the *Z* form, the main determined TD-DFT excitations are 464 nm (*f*=0.034) and 292 nm (*f*=0.194), which correspond to the measured 441 nm and 279 nm absorption experimentally. For the lowest transition, the CC2-corrected value is 434 nm. The relative hypsochromic shifts are qualitatively (but only qualitatively) reproduced.

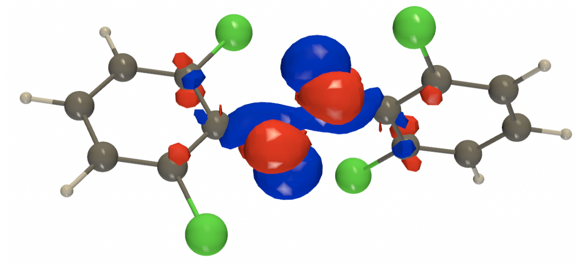

495 nm

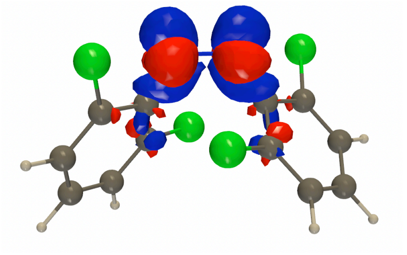

464 nm

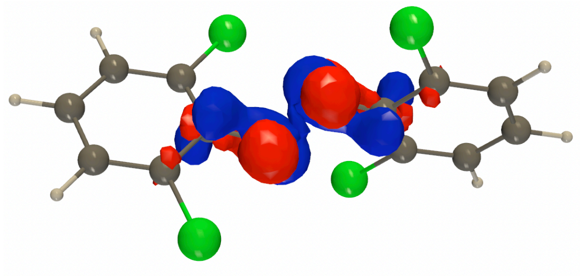

303 nm

4 (*E*)

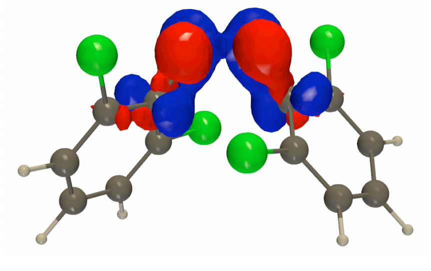

292 nm

4 (*Z*)

Figure 164. Representation of the density difference plot for the main transitions in **4**. The blue and red lobes indicate regions of decrease and increase of density upon excitation, respectively. Representation threshold 4 10^{-3} au.

Localization of the lowest transition.

The computed vertical transition energies are listed in **Table 1**. As can be seen, the CC2-corrected values tend to be closed from their experimental counterpart than the original TD-DFT results, with mean absolute error of 35 and 11 nm, respectively. In all cases, theory foresees that the *Z* form absorbs at more blue-shifted wavelengths than the *E* form, which is the expected trend. The difference between the absorption wavelengths of the two isomers is exaggerated by theory, but the trends are globally correct.

Table S1. Comparison between the computed vertical excitation energies and the experimentally measured λ_{max}, for the lowest-lying transition. All values are given in nm.

		TD-DFT		CC2-corr		Exp.	
Entry	R	*E*	*Z*	*E*	*Z*	*E*	*Z*
**1**	NMe_2	509	496	497	461	*400*[&]	483
**2**	OMe	503	471	476	438	463	452
**3**	Me	498	466	469	435	461	443
**4**	H	495	464	466	434	457	441
**5**	SMe	511	480	482	445	465	452
**6**	Cl	501	467	470	436	456	441
**7**	OAc	498	465	468	433	444	442
**8**	OCF_3	521	465	471	434	461	441
**9**	CF_3	500	467	469	437	459	444
**10**	NO_2	522	486	482	449	467	448

[a] Probably not significant given the strong band overlap.

In the *E* series, the four most redshifted absorption are computed for Entries **1** (497 nm), **5** and **10** (482 nm) and **2** (476 nm). For the former, one cannot compare to experiment because the experimental n-π^* transition is buried under the very intense π - π^* peak, but Entries **2** and **10** are also significantly redshifted experimentally. In the *E* form, the absorption appears in the same spectral regions for all dyes, but for **1** (483 nm) and theory reproduces this effect.

References

[1] G. Bringmann, D. Menche, J. Mühlbacher, M. Reichert, N. Saito, S. S. Pfeiffer, B. H. Lipshutz, *Org. Lett.* **2002**, *4*, 2833–2836.

[2] M. J. Hansen, M. M. Lerch, W. Szymanski, B. L. Feringa, *Angew. Chem. Int. Ed.* **2016**, *55*, 13514–13518.

[3] M. Mizuno, M. Yamano, *Org. Lett.* **2005**, *7*, 3629-3631.

[4] M. Wegener, M. J. Hansen, A. J. M. Driessen, W. Szymanski, B. L. Feringa, *J. Am. Chem. Soc.* **2017**, *139*, 17979–17986.

[5] M. J. Frisch *et al.* Gaussian 16 Revision A.03, 2016, Gaussian Inc. Wallingford CT.

[6] C. Adamo, V. Barone, *J. Chem. Phys.* **1999**, *110*, 6158–6170.

[7] S. Grimme, S. Ehrlich, L. Goergik, *J. Comput. Chem.* **2011**, *32*, 1456–1465.

[8] J. Tomasi, B. Mennucci, R. Cammi, *Chem. Rev.* **2005**, *105*, 2999–3094.

[9] P. M. Vérité, C. A. Guido, D. Jacquemin, *Phys. Chem. Chem. Phys.* **2019**, *21*, 2307–2317.

[10] Turbomole 7.4.1. 2019 a development of University of Karlsruhe and Forschungszentrum Karlsruhe GmbH, 1989-2007, TURBOMOLE GmbH, since 2007; available from http://www.turbomole.com
